# Radical Cross Coupling and Enantioselective Protonation through Asymmetric Photoredox Catalysis

**DOI:** 10.1002/advs.202307773

**Published:** 2024-01-17

**Authors:** Manman Kong, Zhuoxi Wang, Xu Ban, Xiaowei Zhao, Yanli Yin, Junmin Zhang, Zhiyong Jiang

**Affiliations:** ^1^ International Joint Research Center for Molecular Science College of Chemistry and Environmental Engineering College of Physics and Optoelectronic Engineering Shenzhen University Shenzhen 518060 P. R. China; ^2^ School of Chemistry and Chemical Engineering Pingyuan Laboratory Henan Normal University Xinxiang Henan 453007 P. R. China; ^3^ Key Laboratory of Natural Medicine and Immuno‐Engineering of Henan Province Henan University Kaifeng Henan 475004 P. R. China

**Keywords:** asymmetric catalysis, azaarenes, enantioselective protonation, photoredox catalysis, radical coupling

## Abstract

An unprecedented enantioselective protonation reaction enabled by photoredox catalytic radical coupling is developed. Under cooperative dicynopyrazine‐derived chromophore (DPZ) as a photosensitizer and a chiral phosphoric acid catalyst, and Hantzsch ester as a sacrificial reductant, the transformations between α‐substituted enones and cyanoazaarenes or 2‐(chloromethyl)azaaren‐1‐iums can proceed a tandem reduction, radical coupling, and enantioselective protonation process efficiently. Two classes of pharmaceutically important enantioenriched azaarene variants, which contain a synthetically versatile ketone‐substituted tertiary carbon stereocenter at the *β*‐ or *γ*‐position of the azaarenes, are synthesized with high yields and ees.

## Graphical abstract

The first radical coupling and enantioselective protonation is developed. Under cooperative photoredox and chiral hydrogen‐bonding catalysis, the transformations of α‐substituted enones with cyanoazaarenes or 2‐(chloromethyl)azaaren‐1‐iums could render two classes of valuable azaarene variants with high yields and ees, which contain a synthetically useful ketone‐substituted tertiary carbon stereocenter at the *β*‐ or *γ*‐position of the azaarenes.

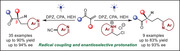

## 1 Introduction

Enantioselective protonation has long been recognized as a convenient method to assemble tertiary carbon stereocenters.^[^ ^1^ ^]^ However, its applications in organic synthesis are still rather limited, due to the formidable enantiocontrol originating from the small volume and rapid motility of the proton. In addition, the labile stereocenters are prone to be deprotonated during the reaction when in the presence of acidic or basic conditions, further compromising the enantioselectivity. As such, for classical manifolds where the key anion intermediates are produced via ground‐state ionic‐type pathways, viable substrates are restricted to the highly reactive carbonyl compounds that enable the transformations to occur under mild reaction conditions.^[^ ^2^, ^3^, ^4^, ^5^, ^6^, ^7^, ^8^, ^9^ ^]^ Meanwhile, the stereocenters of the products cannot possess the strongly electron‐withdrawing functional groups. Such an elusive dilemma has thus inspired constant and diligent exploration of chemists, and photoredox catalysis^[^ ^10^, ^11^ ^]^ has recently been revealed as a promising platform to solve this problem once and for all. The first example is reported in 2017 via a sequential single‐electron reduction and enantioselective protonation, where the labile α‐ketone tertiary alcohols could be directly synthesized from 1,2‐diketones with high enantioselectivities.^[^ ^12^ ^]^ More importantly, this success demonstrates the feasibility of stereocontrol of chiral hydrogen‐bonding (H‐bonding) catalysts for such an extremely reactive platform,^[^ ^13^, ^14^, ^15^, ^16^, ^17^, ^18^, ^19^ ^]^ even if the strong racemic background transformation exists in the reaction. Subsequently, several primary tools of photoredox catalysis have been applied for accomplishing enantioselective protonation, such as reductive dehalogenation,^[^ ^20^, ^21^ ^]^ hydrogen atom transfer,^[^ ^22^ ^]^ radical addition,^[^ ^23^, ^24^ ^]^ and reduction‐radical addition.^[^ ^25^ ^]^ These impressive works not only presented versatile photoredox catalytic platforms for devising novel and significant enantioselective protonation reactions, but also disclosed the robust capability of photocatalytic enantioselective protonation to the convenient preparation of enantioenriched imine‐containing azaarene derivatives from the readily accessible azaarene‐based feedstocks.^[^ ^21^, ^22^, ^23^, ^24^, ^25^, ^26^, ^27^ ^]^ Central to the success is the high reactivity of radical species, leading to the feasibility of these substrates albeit featuring rather low reactivity. In this regard, the exploration of distinct enantioselective protonation patterns enabled by other photocatalytic approaches constitutes a highly desirable task, which will enrich the toolkit of organic synthesis, and considerably promote the advance of pharmaceuticals and material industry, given the ubiquity of azaarenes in natural products, drugs, catalysts, ligands, and functional materials.

**Scheme 1:**
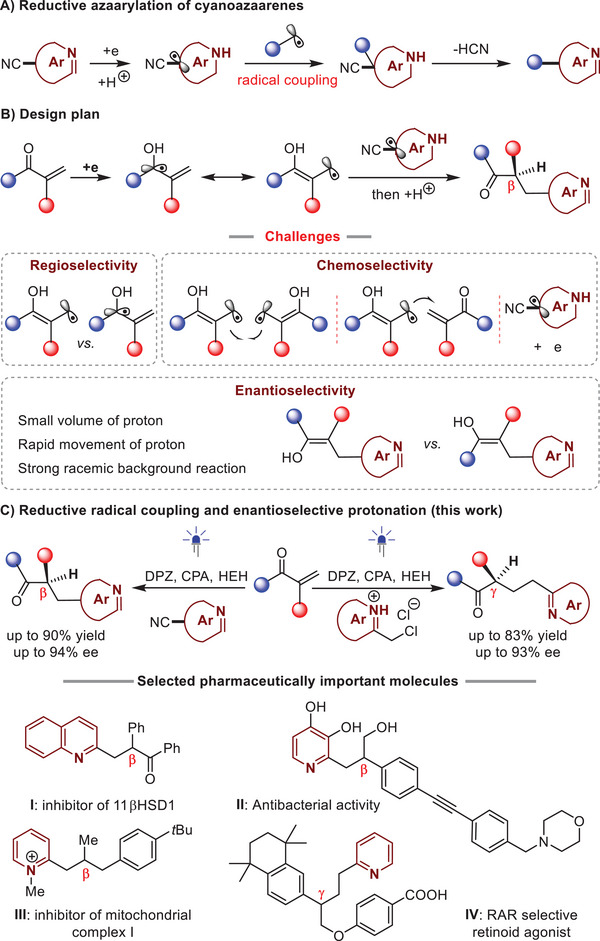
Outline of this work.

Among the fundamental bond‐formation modes of photocatalysis, radical coupling has been widely employed due to the substantially high reactivity arising from the near‐zero activation energy.^[^ ^27^ ^]^ In his context, a wide array of efficient photoredox catalytic reactions using commercially available cyanoazaarenes as feedstocks have been developed (**Scheme** **1**A).^[^ ^26^, ^27^, ^28^, ^29^, ^30^, ^31^, ^32^ ^]^ In addition to good functional group tolerance, such a reductive azaarylation features precise regioselectivity, and represents a powerful tool for the synthesis of valuable azaarene variants. Hence, we were intrigued to explore the viability of a tandem of radical coupling and enantioselective protonation of cyanoazaarenes. We considered that α‐substituted enones should be possible reaction partners, given their ability to ketyl radicals via single‐electron reduction and the less steric terminal carbons relative to the hydroxyl carbon of the resulting radical anions (Scheme 1B).^[^ ^25^, ^33^ ^]^ Importantly, it will provide a modular and facile approach to the valuable azaarene derivatives that contain tertiary carbon stereocenters *β* to azaarenes, where the ketone moiety can be readily modified. In this tentative scenario, since the ketyl radical is also prone to react with the azaaryl radial anion^[^ ^34^ ^]^ and the lifetime of the primary alkyl radical is extremely short, the regioselectivity between ketyls and α‐enolate radicals still poses a formidable challenge. Moreover, given that the two distinct radical species are generated by two separate photoredox catalytic cycles, they will be present individually in different solvent cages, leading to a fairly easy and rapid homocoupling. At the same time, the radical yielded from the enone can conveniently undergo addition to another enone. These adverse effects on chemoselectivity can considerably worsen the yield of the desired product. There are many huge challenges for the enantioselectivity, including those acknowledged issues stemming from proton,^[^ ^1^ ^]^ the difficulty in the precise formation of *Z*‐ or *E*‐enolate intermediates,^[^ ^1^, ^2^, ^3^, ^4^, ^5^, ^6^, ^7^, ^8^, ^9^ ^]^ and the irrepressible racemic background reactions originating from the rather high reactivity of the radical coupling.^[^ ^13^, ^14^, ^15^, ^16^, ^17^, ^18^, ^19^ ^]^

Nevertheless, given the importance of contributing novel enantioselective protonations and the potential of the enantioenriched products in drug discovery, we performed the study. Herein, we report the success of this attractive scenario that is photoredox catalytic radical coupling and enantioselective protonation of α‐substituted enones with cyanoazaarenes by using a transition metal‐free dual catalyst system involving DPZ^[^ ^17^ ^]^ as a photosensitizer and a chiral phosphoric acid (CPA) catalyst and Hantzsch ester (HEH) as the sacrificial reductant (Scheme 1C). Notably, this catalysis platform also allows 2‐(chloromethyl)azaaren‐1‐ium chlorides as the feasible partners of enones. Two series of azaarene derivatives that contain *β*‐ or *γ*‐ketone‐functionalized tertiary carbon stereocenters were obtained in high yields and ees, which are either bioactive (e.g., molecule **I**)^[^ ^35^ ^]^ or the key precursors of many pharmaceutically important compounds (e.g., molecules **II–IV**).^[^ ^36^, ^37^, ^38^ ^]^

## 2 Results and Discussion

The study was commenced by selecting 1,2‐diphenylprop‐2‐en‐1‐one (**1a**) and isoquinoline‐1‐carbonitrile (**2a**) as the model substrates. We first attempted **HEH‐1** as the terminal reductant and 0.5 mol% DPZ as the photoredox catalyst. It was found that the desired product **3a** could be obtained in 47% yield, suggesting the viability of the method and the existence of racemic background reaction for the enantioselective manifold. Nevertheless, we evaluated a series of CPAs, tertiary amines as the reductants, and other reaction parameters.^[^ ^39^ ^]^ To our delight, when at 10 °C with irradiation by a 3 W blue LED for 64 h in the presence of 0.5 mol% DPZ, 20 mol% SPINOL‐CPA (**C1**), 1.2 equiv. of **HEH‐1**, 1.0 equiv. of LiH_2_PO_4_ as an additive, and diethyl ether (Et_2_O) as solvent, **3a** was isolated in 78% yield with 92% ee (entry 1, **Table** **1**). When **C2** or **C15** instead of **C1** was used, ee of **3a** was decreased tremendously (entries 2−3), supporting that the substituents on the 6,6′‐aromatic rings of SPINOL are crucial for the enantiocontrol. Notably, the enantioselectivity can also be influenced by the reductant, as both **HEH‐2** and **HEH‐3** led to **3a** with poorer ee values (entries 4−5). We then examined the effect of photosensitizers (entries 6−7). As a result, 4CzlPN produced **3a** in 48% yield with 65% ee (entry 6). Meanwhile, Ru(III) complex could render **3a** in excellent yield, but the enantioselectivity was deteriorated tremendously (entry 7), revealing the importance of the current dual catalyst system for concurrently accomplishing the satisfactory yield and ee. The subsequent evaluation for the solvent effect disclosed that both CH_2_Cl_2_ and toluene are not suitable for achieving the high enantioselectivity (entries 8−9).

**Table 1:** Optimization of the Reaction Conditions.

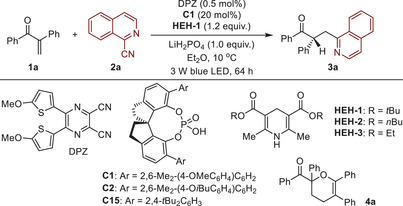			
Entry^a)^	Variation from the standard conditions	Yield [%]^b)^	Ee [%]^c)^
1	None	78	92
2	**C2** instead of **C1**	57	65
3	**C15** instead of **C1**	39	4
4	**HEH‐2** instead of **HEH‐1**	82	50
5	**HEH‐3** instead of **HEH‐1**	83	61
6	4CzIPN instead of DPZ	48	65
7	[Ru(bpz)_3_][PF_6_]_2_ instead of DPZ	88	29
8	CH_2_Cl_2_ instead of Et_2_O	77	13
9	Toluene instead of Et_2_O	82	22
10	No **C1**	60	N.A.
11	No LiH_2_PO_4_	60	82
12	No DPZ	27	17
13	No light	N.R.	N.A.
14	Under air	0^d^ ^)^	N.A.

^a)^ Reaction conditions: **1a** (0.2 mmol), **2a** (0.1 mmol), DPZ (5.0 × 10^−4^ mmol), **C1** (0.02 mmol), **HEH‐1** (0.12 mmol), and LiH_2_PO_4_ (0.1 mmol) in degassed Et_2_O and at 10 °C;

^b)^ Yield of isolated product;

^c)^ Ee was determined by HPLC analysis;

^d)^ **1a** was transformed to **4a** completely. N.A. = not applicable. N.R. = no reaction.

Finally, a series of control experiments were performed to assess the role of the key reaction factors to the transformation. First, when without chiral catalyst **C1**, racemic **3a** was achieved in 60% yield (entry 10), proving that such a photoredox catalytic reaction can occur smoothly in the absence of chiral H‐bonding catalyst. LiH_2_PO_4_ was detected to slightly improve reactivity and enantioselectivity (entry 11). The transformation was also tested in the absence of DPZ, and **3a** was obtained in 27% yield with 17% ee (entry 12). We speculated that it might stem from the ability of HEH to be activated by the applied light source and the different enantiocontrol manners between photocatalysis and the direct photoactivation (vide infra). No reaction in dark supports the indispensability of photons for the transformation to occur (entry 13). The transformation was subsequently examined under air (entry 14). Despite no **3a** achieved, **4a** that is directly derived from **1a** through an acid‐catalyzed cycloaddition reaction was isolated in 87% yield, revealing another important challenge for the chemoselectivity of the desired photocatalytic transformation.

With optimized conditions in hand, the substrate scope of this reductive coupling protocol was explored (**Table** **2**). Isoquinoline‐1‐carbonitrile (**2a**) was first tested to react with diverse α‐substituted enones, and the corresponding adducts **3a**–**i** could be obtained in 55% to 90% yields with 90% to 94% ees. It was found that the introduction of distinct electron‐withdrawing or electron‐donating groups on the aromatic ring of 1‐aryl of enones (i.e., **3b**–**f**) usually presented excellent enantioselectivity. The replacement of simple 1‐aryls by fused aromatic rings could also provide products (e.g., **3g**) with satisfactory results. Importantly, in addition to α‐aryl, the enones featuring α‐benzyl or alkyl groups are compatible with the reaction conditions, leading to **3h**–**i** in 65% and 55% yield, with 91% and 90% ee respectively, Cyclic ketone‐derived exocyclic activated olefins were then examined, and **3j** as a representative product was obtained in 67% yield with 90% ee. Subsequently, the reactions between diphenylprop‐2‐en‐1‐one (**1a**) and diverse isoquinoline‐1‐carbonitriles that contain various substituents on the isoquinoline ring were carried out. Gratifyingly, products **3k**–**s** were attained in 48% to 88% yields with 88% to 93% ees. Other cyanoazaarenes were then tested, and quinoline‐2‐carbonitriles and isonicotinonitriles were demonstrated to be compatible well, resulting in products **3t**‐**‐zj** in high yields and ees. Among these examples, isonicotinonitrile (**2l**) was selected to react with a variety of α‐substituted enones, and all satisfactory results further supported the versatility of this photoredox catalytic strategy. It is worth mentioning that this method is effective for direct‐preparation of the pharmaceutically important molecules (e.g., molecule **I**) in an enantioselective fashion (i.e., product **3t**). Moreover, the enantioenriched ketone‐containing tertiary carbon moieties could be readily assembled onto the structure of bioactive compounds; as an example, the corresponding product **3s** containing fasudil, an important cardiovascular drug, was obtained in 81% yield with 91% ee.

**Table 2:** Reactions between α‐substituted enones and cyanoazaarenes.^a)^

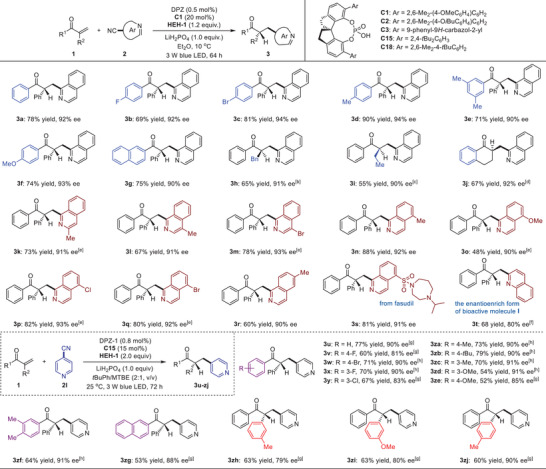

^a)^ The reaction was performed on a 0.1 mmol scale;

^b)^ C18 instead of C1;

^c)^ At −10 °C.

^d)^ C2 instead of C1;

^e)^ C15 instead of C1;

^f)^ C3 instead of C1, 5 mL MTBE;

^g)^ HEH‐1 (2.0 equiv.), tBuPh (4.0 mL), MTBE (2.0 mL);

^h)^ 1a:2a = 2.5:1, HEH‐1 (2.5 equiv.).

Inspired by the success, we were intrigued to challenge reductive coupling of α‐substituted enones with azaarene‐substituted methyl halides as another kind of oxidative azaarene‐containing substrates, therefore providing a modular and expedient enantioselective protonation approach to assemble *γ*‐stereocenters for azaarenes. In addition to the chemoselectivity originating from the different reactivity of the azaaryl α‐alkyl radicals compared to the cyano α‐radicals, the formidable challenges also involve the enantioselectivity, since these halides are quite unstable and the feedstocks are only commercial‐available in the forms of their salts within organic acids. Accordingly, the catalytic effects of the chiral H‐bonding catalysts may be susceptible to significant effects. Likely for this reason, no examples of asymmetric photocatalytic reactions of these entities have been reported.

At the beginning, we selected **1a** and 2‐(chloromethyl)quinolin‐1‐ium chloride **5a** as the model substrates, and evaluated the reaction by using 1.0 mol% DPZ and 1.0 equiv. of **HEH‐1**, and importantly, adding stoichiometric amount of inorganic or organic bases to basify **5a** as the neutral quinoline form. However, although numerous attempts were conducted, trace amount of product **6a** was obtained, and the ee of **6a** was rather low when diverse CPAs as chiral catalysts were used. The dilemma prompted us to directly explore the reaction without extra bases, given that the existence of proton can act as an H‐bonding donor. To our delight, **6a** was finally achieved in 77% yield with 90% ee when employing our developed another dicyanopyrazine chromophore as the photosensitizer (i.e., TDPZ),^[^ ^40^, ^41^ ^]^ 20 mol% CPA **C19**, 2.0 equiv. of **HEH‐6** as the terminal reductant, the mixed bromobenzene/CH_2_Cl_2_ in a 5:1 ratio as the solvent, and at 10 °C (**Table** **3**). The substrate scope was subsequently examined, and various ketone‐substituted tertiary carbon stereocenters were therefore successfully forged at the *γ*‐position of quinolines and pyridines. Notably, in addition to linear α‐enones (**6a**‐**‐h**), exocyclic enones were tolerant, and adduct **6i** as a representative was obtained in 61% yield with 78% ee.

**Table 3:** Reactions between α‐substituted enones and 2‐(chloromethyl)azaaren‐1‐ium chlorides.^a)^

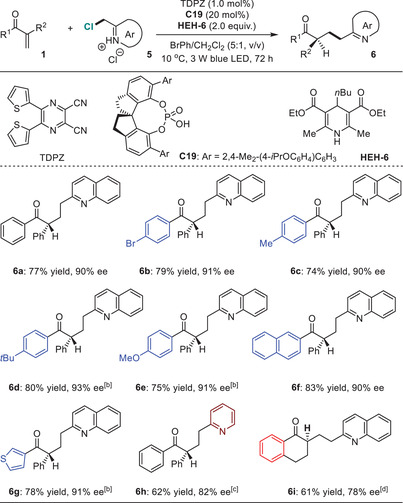

^a)^ The reaction was performed on a 0.1 mmol scale;

^b)^ At 0 °C;

^c)^ DPZ instead of TDPZ, at −15 °C, ethyl acetate (5.0 mL);

^d)^ DPZ instead of TDPZ, C2 instead of C19, HEH‐6 (1.2 equiv.), at −10 °C, CHCl_3_ (5.0 mL).

**Scheme 2:**
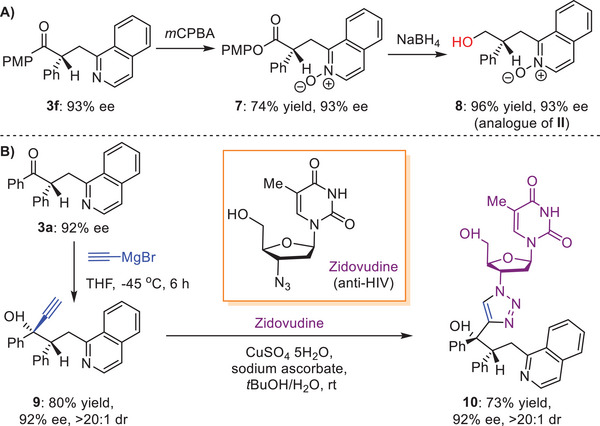
Synthetic applications.

**Scheme 3:**
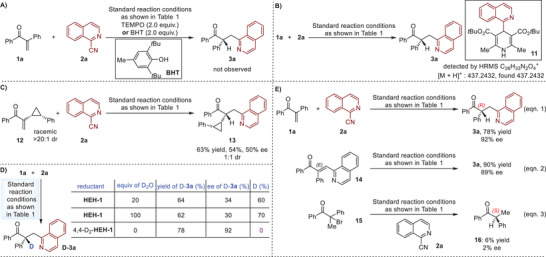
Mechanistic studies.

Although the synthetic utility of this method has been disclosed by the direct synthesis of the enantioenriched inhibitor of 11*β*HSD1 (i.e., **I**, Scheme 1C and **3t**, Table 2) and drug derivatives (e.g., **3s**, Table 2), several simple stream‐down transformations of the products were carried out by modifying the ketone moiety, to further demonstrate its practicability. Baeyer–Villiger oxidation of **3f** was first performed by using *m*CPBA, leading to ester **7** in 74% yield (**Scheme** **2**A). By treatment of NaBH_4_, reduction of **7** occurred, offering product **8** in 96% yield without any erosion of ee. In this regard, the method has established a convenient asymmetric catalytic approach to access the valuable compounds with the attractive antibacterial activity (e.g., **II**, Scheme 1C). To render more complex molecules, **3a** was used to first react with ethynylmagnesium bromide at −45 °C, and after 6 h, the corresponding product **9** containing a *β*‐tertiary carbon stereocenter and a remoter *γ*‐quaternary carbon stereocenter was achieved in 80% yield with 92% ee and >20:1 dr (Scheme 2B). The acetylenyl of **9** was found to readily react with azide of the anti‐HIV drugZidovudine in the presence of CuSO_4_ and sodium ascorbate, resulting in the attractive Zidovudine derivative **10** in 73% yield with 92% ee and >20:1 dr.

Subsequently, investigations on the plausible mechanism were commenced (**Scheme** **3**). We first attempted the model reaction of **1a** with **2a** in the presence of 2.0 equiv. of TEMPO (Scheme 3a). We found that **1a** was completely converted to the six‐membered ring side product **4a** (see Table 1 for the structure), and no **3a** was detected. Similar result was obtained when using another common radical scavenger BHT. Accordingly, a radical process should operate in the process. We then analyzed the reaction mixture of the transformation between **1a** and **2a** using high resolution mass spectrometer, and adduct **11** that is derived from **2a** and **HEH‐1** was recorded, supporting the formation of the corresponding isoquinolyl radical (Scheme 3B).^[^ ^39^ ^]^ To clarify the most possible reaction pattern of the newly formed C(sp^3^)─C(sp^3^) bond, that is proposed as electrophilic azaaryl radical's addition to the enones or coupling with the α‐enolate radicals resulting from the enones, radical clock experiment using enone‐derived racemic cyclopropane **12** (>20:1 dr) with **2a** was conducted (Scheme 3C). It was found that the corresponding adduct **13** was obtained in 63% yield with 54% and 50% ee with 1:1 dr. The retained cyclopropane group excluded the possibility of the formation of α‐ketone radicals via a radical addition to enones. Next, the transformation of **1a** with **2a** was performed in the presence of D_2_O or employing 4,4‐D_2_‐**HEH‐2** as the reductant (Scheme 3D). The outcomes suggested that protonation accounts for the C−H bonds of the tertiary carbon stereocenters. Furthermore, to answer the plausible sequential order between radical coupling and enantioselective protonation, olefin **14** was synthesized and subjected to the standard reaction conditions (Equation (2), Scheme 3E). It was found that product **3a** was obtained in 90% yield with 89% ee, which is fully similar to the result of the reaction of **1a** with **2a** (entry 1, Table 1 and Equation (1), Scheme 3E). In addition, when under the current reaction conditions where **2a** is useful for create a similar acid–base environment, bromide **15** could render product **16** in 6% yield with 2% ee (Equation (3), Scheme 3E). Notably, the absolute configuration of the stereocenter in **16** is *S*, but that of **3a** is *R*. In this context, radical coupling might occur prior to enantioselective protonation.

With the aforementioned information in hand, we subsequently conducted Stern–Volmer experiments to investigate the rational photoredox catalytic cycle.^[^ ^39^ ^]^ As a result, no measureable fluorescence quenching of *DPZ by 1,2‐diphenylprop‐2‐en‐1‐one (**1a**) or isoquinoline‐1‐carbonitrile (**2a**) in the absence and presence of chiral acid **C1** was observed. Notably, when without DPZ, the reaction is still workable (entry 12, Table 1). As such, we measured UV–vis absorption spectra of **1a**, **2a**, and **HEH‐1**, revealing that **HEH‐1** can be directly activated by the applied LED. It is noteworthy that less than 2.0 equiv. of **HEH‐1** is sufficient to provide electrons for the transformation (Table 1). Accordingly, the photoredox catalysis engaged by DPZ should be triggered by the single‐electron oxidation of **HEH‐1^•^** ^[^ ^42^ ^]^ radical generated from the reduction of **2a** (*E*_p_ = −1.18 V vs saturated calomel electrode (SCE) in CH_3_CN)^[^ ^39^ ^]^ by the photoactivated **HEH‐1** (i.e., *HEH‐1, *E* ^t^(S*/S^•−^) = −2.28 V vs the SCE in CH_3_CN)^[^ ^43^ ^]^ as the Stern–Volmer experiment results revealed a clear fluorescence quench of **HEH‐1** with **2a** but not **1a** (*E*_p_ = −1.07 V vs SCE in CH_3_CN).^[^ ^39^ ^]^ In this context, we conjectured that **C1** should participate in the reduction of **1a** through H‐bonding interaction, which is indispensable for achieving the high enantioselectivity by providing an efficient enantioface differentiation and avoiding the racemic background reaction. In other words, the reduction of **1a** via proton‐coupled electron transfer (PCET) by DPZ^•−^ (*E*_red_^1/2^ = −1.08 V vs SCE in CH_3_CN) should be engaged by chiral catalyst **C1**.

**Scheme 4:**
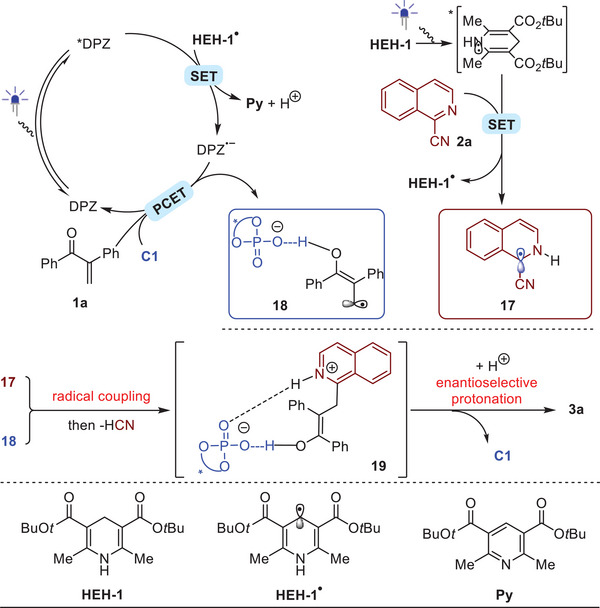
The proposed mechanism.

On the basis of these results, the plausible mechanism of this visible light‐initiated photoredox catalytic reductive coupling of cyanoazaarenes with α‐substituted enones is proposed. As shown in **Scheme** **4**, with the reaction between **1a** and **2a** as a representative, **2a** is first reduced by *HEH‐1, thereby leading to **HEH‐1^•^** and radical **17**. The reductive quenching of *DPZ subsequently occurs with **HEH‐1•** as the reductant. Finally, PCET between DPZ^•−^ and **C1**‐activated **1a** results in a radical anion that interacts with **C1** through H‐bonding (i.e., **18**). After cross coupling of two radical species that are **17** and **18** and then the removal of HCN, the key intermediate **19** participated by molecule **C1** is generated, which is crucial to the success of achieving excellent enantioselectivity.

## 3 Conclusion

In summary, we have developed the first enantioselective protonation enabled by photoredox catalytic radical coupling. When under a visible light‐induced dual catalyst system involving a photosensitizer and a chiral phosphoric acid and employing Hantzsch ester as the terminal reductant, the reaction of α‐substituted enones with various cyanoazaarenes could work with high yields and ees. The robustness of the current transition‐metal free catalytic system was further demonstrated the viability of 2‐(chloromethyl)azaaren‐1‐ium chlorides as the reaction partner of α‐substituted enones to undergo the attractive radical coupling and enantioselective protonation process. All reactions are important regarding that they open the first and fruitful synthetic avenue for two classes of pharmaceutically important enantioenriched azaarene variants, which contain a synthetically useful ketone‐substituted tertiary carbon stereocenter at the *β*‐ or *γ*‐position of the azaarenes. It is worth mentioning that this work represents the first example of assembling two such remote stereocenters (i.e., *β* and *γ*) for azaarenes via enantioselective protonation. We anticipate that the current achievements, involving the verified viability of cooperating the greatly reactive radical coupling with enantioselective protonation, will motivate the increasing pursuit for more kinds of important and challenging asymmetric photocatalytic reactions, thereby facilitating the rapid advancement in the pharmaceutical industry.

## Conflict of Interest

The authors declare no conflict of interest.

## Supporting information



Supporting Information

Supporting Information

Supporting Information

## Data Availability

The data that support the findings of this study are available from the corresponding author upon reasonable request.
