# Supplementary material for: Radical Cross Coupling and Enantioselective Protonation through Asymmetric Photoredox Catalysis
Source: Adv Sci (Weinh). 2024 Jan 17;11(12):2307773. doi: 10.1002/advs.202307773 (PMC10966550; doi:10.1002/advs.202307773)
Supplement: Supplementary file 1 — Supporting Information [file ADVS-11-2307773-s001.pdf]

ADVANCED SCIENCE
Open Access

Supporting Information

for *Adv. Sci.*, DOI 10.1002/advs.202307773

Radical Cross Coupling and Enantioselective Protonation through Asymmetric Photoredox Catalysis

Manman Kong, Zhuoxi Wang, Xu Ban, Xiaowei Zhao, Yanli Yin, Junmin Zhang and Zhiyong Jiang**

Supporting Information

Radical Cross Coupling and Enantioselective Protonation through Asymmetric Photoredox Catalysis

Manman Kong,[a,c] Zhuoxi Wang,[b] Xu Ban,[c] Xiaowei Zhao,[b] Yanli Yin,[c] Junmin Zhang,*[a] and Zhiyong Jiang*[a,b,c]

[a] International Joint Research Center for Molecular Science, College of Chemistry and Environmental Engineering, College of Physics and Optoelectronic Engineering, Shenzhen University, Shenzhen, 518060, P. R. China

[b] Key Laboratory of Natural Medicine and Immuno-Engineering of Henan Province, Henan University, Kaifeng, Henan 475004, P. R. China

[c] School of Chemistry and Chemical Engineering, Pingyuan Laboratory Henan Normal University, Xinxiang, Henan 453007, P. R. China

Email: chmjzy@henu.edu.cn

Table of Contents

1. General information **S3-S4**

2. Optimization of reaction conditions **S5-S8**

3. General experimental procedures **S9-S11**

4. Mechanism studies **S12-S22**

5. Synthetic applications **S23-S24**

6. Determination of the absolute configurations **S25-S38**

7. Characterization of adducts **S39-S78**

8. Copies of NMR spectra **S79-S134**

9. Unsuccessful reactions **S135**

1. General information

Experiments involving moisture and/or air sensitive components were performed under a positive pressure of argon in oven-dried glassware equipped with a rubber septum inlet. Dried solvents and liquid reagents were transferred by oven-dried syringes or hypodermic syringe cooled to ambient temperature in a desiccator. Reactions mixtures were stirred in 25 mL Schlenk tube with Teflon-coated magnetic stirring bars unless otherwise stated. Moisture in non-volatile reagents/compounds was removed in high *vacuo* by means of an oil pump and subsequent purging with nitrogen. Solvents were removed *in vacuo* under ~30 mmHg and heated with a water bath at 30–35 ºC using rotary evaporator with aspirator. The condenser was cooled with running water at 0 ºC.

All experiments were monitored by analytical thin layer chromatography (TLC). TLC was performed on pre-coated plates, 60 F_{254}. After elution, plate was visualized under UV illumination at 254 nm for UV active material. Further visualization was achieved by staining phosphomolybdic acid ethanol solution. For those using the aqueous stains, the TLC plates were heated on a hot plate.

Columns for flash chromatography (FC) contained *silica gel* 200–300 mesh. Columns were packed as slurry of *silica gel* in petroleum ether and equilibrated solution using the appropriate solvent system. The elution was assisted by applying pressure of about 2.0 atm with an air pump.

Instrumentations

Proton nuclear magnetic resonance (^{1}H NMR) and carbon NMR (^{13}C NMR) were recorded in $CDCl_3$ otherwise stated. Chemical shifts are reported in parts per million (ppm), using the residual solvent signal as an internal standard: $CDCl_3$ (^{1}H NMR: δ 7.26, singlet; ^{13}C NMR: δ 77.0, triplet). Multiplicities were given as: *s* (singlet), *d* (doublet), *t* (triplet), *q* (quartet), *quintet*, *m* (multiplets), *dd* (doublet of doublets), *dt* (doublet of triplets), and *br* (broad). Coupling constants (*J*) were recorded in hertz (Hz). The number of proton atoms (*n*) for a given resonance was indicated by *n*H. The number of carbon atoms (*n*) for a given resonance was indicated by *n*C. HRMS (Analyzer: TOF) was reported in units of mass of charge ratio (m/z). Mass samples were dissolved in CH_3CN (HPLC Grade) unless otherwise stated. Optical rotations were recorded on a polarimeter with a sodium lamp of wavelength 589 nm and reported as follows; $[\alpha]_{\lambda}^{T°C}$ (*c* = g/100 mL, solvent). Melting points were determined on a melting point apparatus.

Enantiomeric excesses were determined by chiral High Performance Liquid Chromatography (HPLC) analysis. UV detection was monitored at 254 nm and 210 nm at the same time. HPLC samples were dissolved in HPLC grade isopropanol (IPA) unless otherwise stated.

Materials

All commercial reagents were purchased with the highest purity grade. They were used without further purification unless specified. All solvents used, mainly petroleum ether (PE) and ethyl acetate (EtOAc) were distilled. Anhydrous dichloromethane (DCM), CH_3CN were freshly distilled from CaH_2 and stored under N_2 atmosphere. THF, Et_2O, *t*BuPh, mesitylene, and toluene were freshly distilled from sodium/benzophenone before use. All compounds synthesized were stored in a −80 °C freezer and light-sensitive compounds were protected with aluminium foil.

2. Optimization of reaction conditions

Table S1. Optimization of Reaction Conditions-(1)[a]

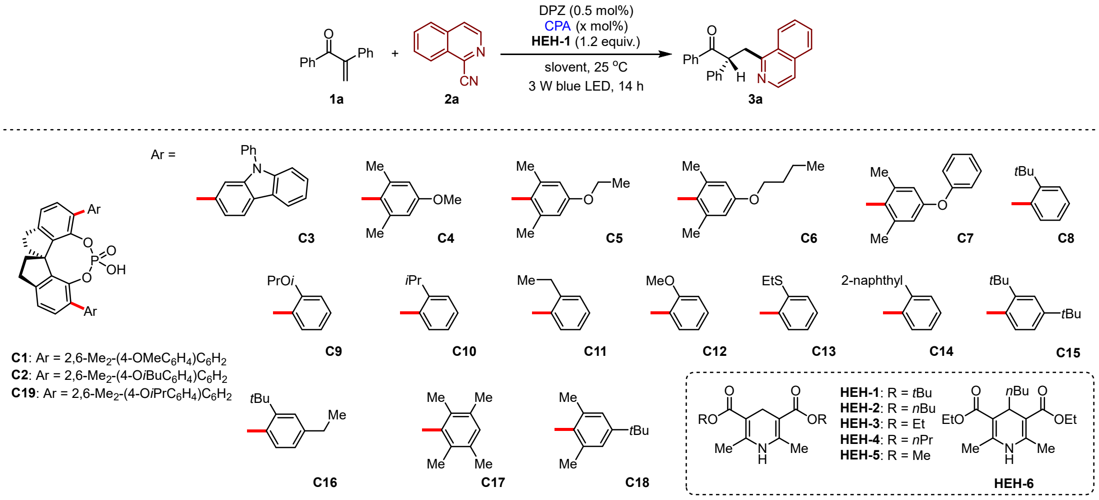

entry	CPA (10 mol%)	solvent (mL)	ee (%)[b]
1	**C1**	CH_2Cl_2 (0.3)	79
2	**C1**	toluene (0.3)	82
3	**C1**	Et_2O (0.3)	84
4	**C4**	Et_2O (0.3)	25
5	**C5**	Et_2O (0.3)	32
6	**C6**	Et_2O (0.3)	16
7	**C7**	Et_2O (0.3)	8
8	**C8**	Et_2O (0.3)	20
9	**C9**	Et_2O (0.3)	-10
10	**C10**	Et_2O (0.3)	2
11	**C11**	Et_2O (0.3)	-27
12	**C13**	Et_2O (0.3)	-20
13	**C14**	Et_2O (0.3)	38
14	**C15**	Et_2O (0.3)	11
15	**C16**	Et_2O (0.3)	-5
16	**C17**	Et_2O (0.3)	10

[a]Reaction conditions: **1a** (0.02 mmol), **2a** (0.01 mmol). LED distance = 3 cm. Note: a series of BINOL-CPAs have also been examined, but no better result was obtained. [b]Determined by HPLC analysis on a chiral stationary phase. N.A. = not applicable. N.R. = no reaction.

Table S2. Optimization of Reaction Conditions-(2)[a]

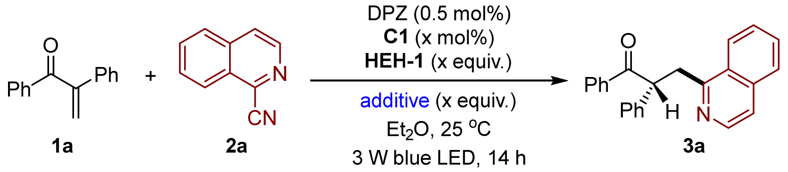

entry	**C1** (x mol%)	**HEH-1** (x equiv.)	additive (x equiv.)	ee (%)[b]
1	5	2.0	-	73
2	7.5	2.0	-	80
3	10	2.0	-	83
4	12.5	2.0	-	83
5	15	2.0	-	84
6	20	2.0	-	85
7	20	1.0	-	90
8	20	1.2	-	90
9	20	1.5	-	89
10[c]	20	1.2	-	87
11[d]	20	1.2	LiH_2PO_4 (1.0)	92

[a]Reaction conditions: **1a** (0.02 mmol), **2a** (0.01 mmol). LED distance = 3 cm. [b]Determined by HPLC analysis on a chiral stationary phase. [c]0.1 mmol scale, t = 64 h, yield = 86%. [d]0.1 mmol scale, 10 °C, t = 64 h, yield = 78%. Note: a series of additive have also been examined, but no better result was obtained.

Table S3. Optimization of Reaction Conditions-(3)[a]

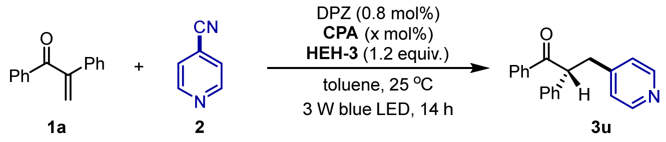

entry	CPA (10 mol%)	ee (%)[b]
1	**C5**	59
2	**C8**	76
3	**C9**	28
4	**C10**	6
5	**C11**	75
6	**C13**	28
7	**C15**	76
8	**C16**	55

[a]Reaction conditions: **1a** (0.02 mmol), **2a** (0.01 mmol). LED distance = 3 cm. Note: a series of BINOL-CPAs have also been examined, but no better result was obtained. [b]Determined by HPLC analysis on a chiral stationary phase.

Table S4. Optimization of Reaction Conditions-(4)[a]

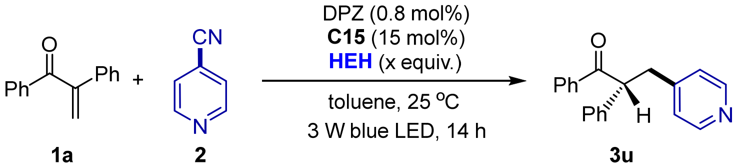

entry	**HEH** (x equiv.)	ee (%)[b]
1	**HEH-1** (1.2)	85
2	**HEH-2** (1.2)	76
3	**HEH-3** (1.2)	76
4	**HEH-4** (1.2)	76
5	**HEH-5** (1.2)	74
6	**HEH-1** (1.5)	85
7	**HEH-1** (2.0)	87

[a]Reaction conditions: **1a** (0.02 mmol), **2a** (0.01 mmol). LED distance = 3 cm. [b]Determined by HPLC analysis on a chiral stationary phase.

Table S5. Solvent Effect Investigation-(5)[a]

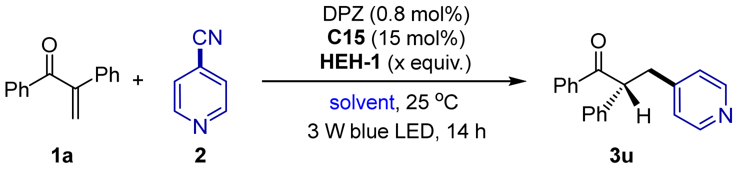

entry	solvent (x mL)	ee (%)[b]
1	toluene (0.6)	88
2	ethylbenzene (0.6)	93
3	*p*-xylene (0.6)	77
4	*tert*-Butylbenzene (0.6)	87
5	MTBE (0.6)	82
6	toluene (0.4) + MTBE (0.2)	87
7[c]	*tert*-Butylbenzene (0.4) + MTBE (0.2)	91
8	*tert*-Butylbenzene (0.5) + MTBE (0.1)	93
9	*tert*-Butylbenzene (0.3) + MTBE (0.3)	90
10	*tert*-Butylbenzene (0.4) + ETBE (0.2)	93

[a]Reaction conditions: **1a** (0.02 mmol), **2a** (0.01 mmol). LED distance = 3 cm. [b]Determined by HPLC analysis on a chiral stationary phase. [c]0.1 mmol scale, t = 72 h, yield = 77%.

Table S6. Optimization of Reaction Conditions-(6)[a]

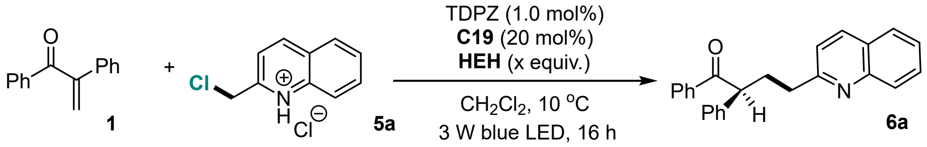

entry	**HEH** (x equiv)	ee (%)[b]
1	**HEH-1** (1.2)	76
2	**HEH-2** (1.2)	76
3	**HEH-3** (1.2)	76
4	**HEH-4** (1.2)	72
5	**HEH-5** (1.2)	70
6	**HEH-6** (1.2)	81
7	**HEH-6** (2.0)	83

[a]Reaction conditions: **1a** (0.02 mmol), **2a** (0.01 mmol). LED distance = 3 cm. [b]Determined by HPLC analysis on a chiral stationary phase.

Table S7. Solvent Effect Investigation-(7)[a]

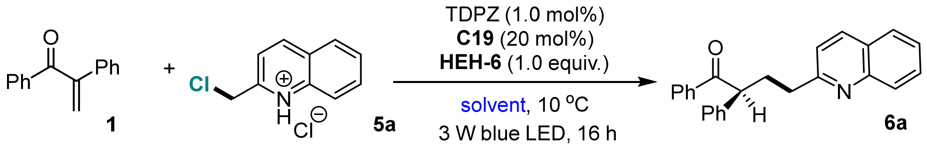

entry	solvent (mL)	ee (%)[b]
1	CH_2Cl_2 (0.3)	80
2	chlorobenzene (0.3)	87
3	bromobenzene (0.3)	88
4	1,3,5-trifluorobenzene (0.3)	73
5	4-bromofluorobenzene (0.3)	87
6	2-bromofluorobenzene (0.3)	83
7	3-bromofluorobenzene (0.3)	84
8	pentafluorobenzene (0.3)	76
9	fluorobenzene (0.3)	87
10[c]	bromobenzene (0.25) + DCM (0.05)	91

[a]Reaction conditions: **1a** (0.015 mmol), **2a** (0.01 mmol). LED distance = 3 cm. [b]Determined by HPLC analysis on a chiral stationary phase. [c]0.1 mmol scale, t = 72 h, yield = 77%.

3. General experimental procedures

(1) General procedure for reaction of enones 1 with cyanoazaarenes 2

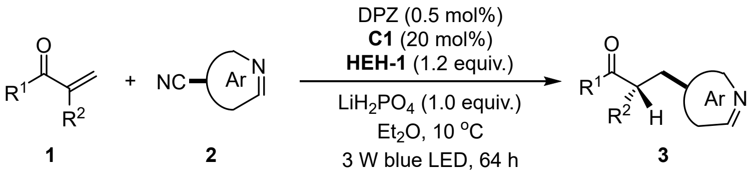

70.0 μL (0.001 mmol, 0.01 equiv) of DPZ solution (1.0 mg of DPZ in 200 μL of toluene) was added into a 25 mL Schlenk tube, and then solvent was removed in *vacuo*.

<u>For 3a–3g, 3l, 3n, 3r, 3s</u>: **1** (0.20 mmol, 2.0 equiv), **2** (0.10 mmol, 1.0 equiv), **C1** (0.02 mmol, 0.20 equiv), **HEH-1** (0.12 mmol, 1.2 equiv), lithium dihydrogen phosphate (0.10 mmol, 1.0 equiv) and Et_2O (3.0 mL) were sequentially added into the Schlenk tube, and the reaction was degassed three times by freeze-pump-thaw method. Then the mixture worked at 10 °C under argon atmosphere with an irradiation of a 3 W blue LED (λ = 450～455 nm) for another 64 h.

<u>For 3h</u>: **1** (0.20 mmol, 2.0 equiv), **2** (0.10 mmol, 1.0 equiv), **C18** (0.02 mmol, 0.20 equiv), **HEH-1** (0.12 mmol, 1.2 equiv), lithium dihydrogen phosphate (0.10 mmol, 1.0 equiv) and Et_2O (3.0 mL) were sequentially added into the Schlenk tube, and the reaction was degassed three times by freeze-pump-thaw method. Then the mixture worked at 10 °C under argon atmosphere with an irradiation of a 3 W blue LED (λ = 450～455 nm) for another 64 h.

<u>For 3i</u>: **1** (0.20 mmol, 2.0 equiv), **2** (0.10 mmol, 1.0 equiv), **C1** (0.02 mmol, 0.20 equiv), **HEH-1** (0.12 mmol, 1.2 equiv), lithium dihydrogen phosphate (0.10 mmol, 1.0 equiv) and Et_2O (3.0 mL) were sequentially added into the Schlenk tube, and the reaction was degassed three times by freeze-pump-thaw method. Then the mixture worked at -10 °C under argon atmosphere with an irradiation of a 3 W blue LED (λ = 450～455 nm) for another 64 h.

<u>For 3j</u>: **1** (0.20 mmol, 2.0 equiv), **2** (0.10 mmol, 1.0 equiv), **C2** (0.02 mmol, 0.20 equiv), **HEH-1** (0.12 mmol, 1.2 equiv), lithium dihydrogen phosphate (0.10 mmol, 1.0 equiv) and Et_2O (3.0 mL) were sequentially added into the Schlenk tube, and the reaction was degassed three times by freeze-pump-thaw method. Then the mixture worked at 10 °C under argon atmosphere with an irradiation of a 3 W blue LED (λ = 450～455 nm) for another 64 h.

<u>For 3k, 3m, 3o-3q</u>: **1** (0.20 mmol, 2.0 equiv), **2** (0.10 mmol, 1.0 equiv), **C15** (0.02 mmol, 0.20 equiv), **HEH-1** (0.12 mmol, 1.2 equiv), lithium dihydrogen phosphate (0.10 mmol, 1.0 equiv) and Et_2O (3.0 mL) were sequentially added into the Schlenk tube, and the reaction was degassed three times by freeze-pump-thaw method. Then the mixture worked at 10 °C under argon atmosphere with an irradiation of a 3 W blue LED (λ = 450～455 nm) for another 64 h.

<u>For 3t</u>: **1** (0.20 mmol, 2.0 equiv), **2** (0.10 mmol, 1.0 equiv), **C3** (0.02 mmol, 0.20 equiv), **HEH-1** (0.12 mmol, 1.2 equiv), lithium dihydrogen phosphate (0.10 mmol, 1.0 equiv) and MTBE (5.0 mL) were sequentially added into the Schlenk tube, and the reaction was

degassed three times by freeze-pump-thaw method. Then the mixture worked at 10 °C under argon atmosphere with an irradiation of a 3 W blue LED (λ = 450～455 nm) for another 64 h.

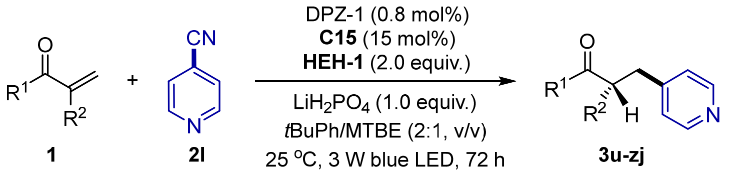

70.0 μL (0.001 mmol, 0.01 equiv) of DPZ solution (1.0 mg of DPZ in 200 μL of toluene) was added into a 25 mL Schlenk tube, and then solvent was removed in *vacuo*.

<u>**For 3u, 3v, 3y, 3ze, 3zg-3zj**</u>: **1** (0.20 mmol, 2.0 equiv), **2** (0.10 mmol, 1.0 equiv), **C15** (0.015 mmol, 0.15 equiv), **HEH-1** (0.20 mmol, 2.0 equiv), lithium dihydrogen phosphate (0.10 mmol, 1.0 equiv), *t*Bu-Ph (4.0 mL) and MTBE (2.0 mL) were sequentially added into the Schlenk tube, and the reaction was degassed three times by freeze-pump-thaw method. Then the mixture worked at 25 °C under argon atmosphere with an irradiation of a 3 W blue LED (λ = 450～455 nm) for another 72 h.

<u>**For 3w, 3x, 3za-3zd, 3zf**</u>: **1** (0.25 mmol, 2.5 equiv), **2** (0.10 mmol, 1.0 equiv), **C15** (0.015 mmol, 0.15 equiv), **HEH-1** (0.25 mmol, 2.5 equiv), lithium dihydrogen phosphate (0.10 mmol, 1.0 equiv), *t*Bu-Ph (4.0 mL) and MTBE (2.0 mL) were sequentially added into the Schlenk tube, and the reaction was degassed three times by freeze-pump-thaw method. Then the mixture worked at 25 °C under argon atmosphere with an irradiation of a 3 W blue LED (λ = 450～455 nm) for another 72 h.

(2) General procedure for reaction of enones 1 with 2-(chloromethyl)azaaren-1-ium chlorides 5

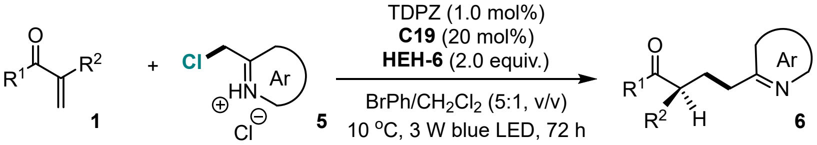

60.0 μL (0.001 mmol, 0.01 equiv) of TDPZ solution (1.0 mg of TDPZ in 200 μL of toluene) was added into a 25 mL Schlenk tube, and then solvent was removed in *vacuo*.

<u>**For 6a-6c, 6f**</u>: **1** (0.20 mmol, 2.0 equiv), **5** (0.10 mmol, 1.0 equiv), **C19** (0.02 mmol, 0.20 equiv), **HEH-6** (0.20 mmol, 2.0 equiv), bromobenzene (2.5 mL) and DCM (0.5 mL) were sequentially added to the Schlenk tube, and the reaction was degassed three times by freeze-pump-thaw method. Then the mixture worked at 10 °C under argon atmosphere with an irradiation of a 3 W blue LED (λ = 450～455 nm) for another 72 h. $NaHCO_3$ (3 mL of a 0.36 M) was added to the mixture and stirred for 15 min.

<u>**For 6d, 6e, 6g**</u>: **1** (0.20 mmol, 2.0 equiv), **5** (0.10 mmol, 1.0 equiv), **C19** (0.02 mmol, 0.20 equiv), **HEH-6** (0.20 mmol, 2.0 equiv), bromobenzene (2.5 mL) and DCM (0.5 mL) were sequentially added to the Schlenk tube, and the reaction was degassed three times by

freeze-pump-thaw method. Then the mixture worked at 0 ºC under argon atmosphere with an irradiation of a 3 W blue LED (λ = 450～455 nm) for another 72 h. $NaHCO_3$ (3 mL of a 0.36 M) was added to the mixture and stirred for 15 min.

For 6h: **1** (0.20 mmol, 2.0 equiv), **7** (0.10 mmol, 1.0 equiv), DPZ (0.001 mmol, 0.01 equiv), **C19** (0.02 mmol, 0.20 equiv), **HEH-6** (0.12 mmol, 1.2 equiv) and ethyl acetate (5.0 mL) were sequentially added to the Schlenk tube, and the reaction was degassed three times by freeze-pump-thaw method. Then the mixture worked at -15 ºC under argon atmosphere with an irradiation of a 3 W blue LED (λ = 450～455 nm) for another 72 h. $NaHCO_3$ (3 mL of a 0.36 M) was added to the mixture and stirred for 15 min.

For 6i: **1** (0.20 mmol, 2.0 equiv), **5** (0.10 mmol, 1.0 equiv), DPZ (0.001 mmol, 0.01 equiv), **C19** (0.02 mmol, 0.20 equiv), **HEH-6** (0.12 mmol, 1.2 equiv) and Chloroform (5.0 mL) were sequentially added to the Schlenk tube, and the reaction was degassed three times by freeze-pump-thaw method. Then the mixture worked at -10 ºC under argon atmosphere with an irradiation of a 3 W blue LED (λ = 450～455 nm) for another 72 h. $NaHCO_3$ (3 mL of a 0.36 M) was added to the mixture and stirred for 15 min.

The reaction was monitored by TLC. After completion, the reaction mixture was directly loaded onto a short *silica gel* column, followed by gradient elution with petroleum ether/dichloromenthan/acetone (2/1/0–1/1/0.1 ratio). Removing the solvent in *vacuo*, afforded products **3a**–**6i**.

4. Mechanism studies

(1) UV-vis absorption spectra

UV-vis absorption spectroscopy was performed using a spectrophotometer, equipped with a temperature control unit at 25 °C. The samples were measured in a 3.0 mL quartz cuvette fitted with a PTFE stopper, **1a**, **2a**, **C1**, and **HEH-1** *et al* were prepared a 0.1 mM solution and used fresh Et_2O for measurement.

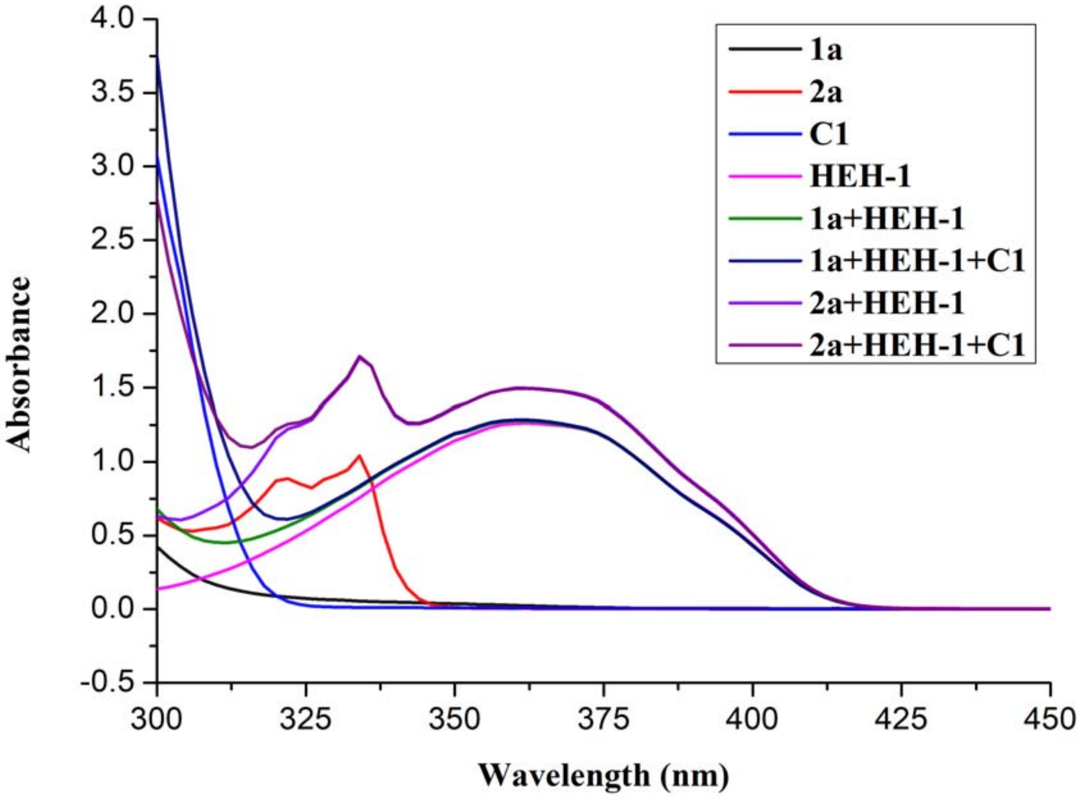

Fig. S1. UV-vis absorption spectra of **1a**, **2a**, **C1** and **HEH-1** *et al*.

(2) Emission quenching experiments

The Luminescence Quenching Experiments of DPZ (excitation wavelength = 448 nm)

Emission intensities were recorded on a spectrofluorometer. DPZ solution was excited at 448 nm and the emission intensity at 535 nm was observed. A solution of DPZ (1.0×10^{-4} M) was added to the appropriate amount of quencher in 3.0 mL volumetric flask under N_2. The solution was transferred to a 3.0 mL quartz cell and the emission spectrum of the sample was collected.

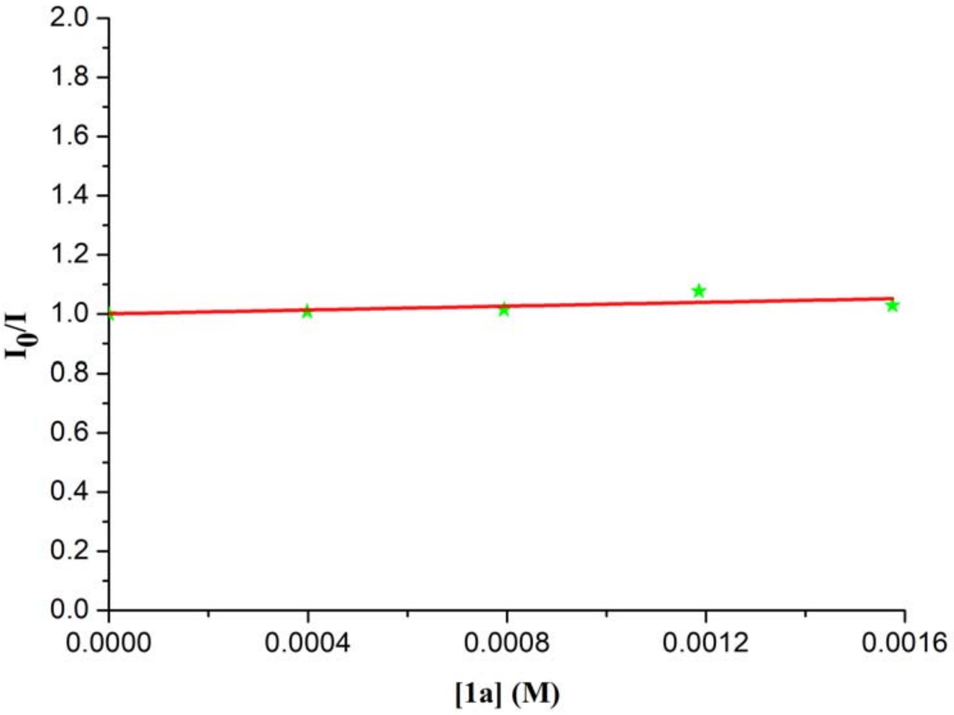

Fig. S2. Stern–Volmer quenching experiment of *DPZ and **1a**.

(No measurable luminescence quenching in MeCN)

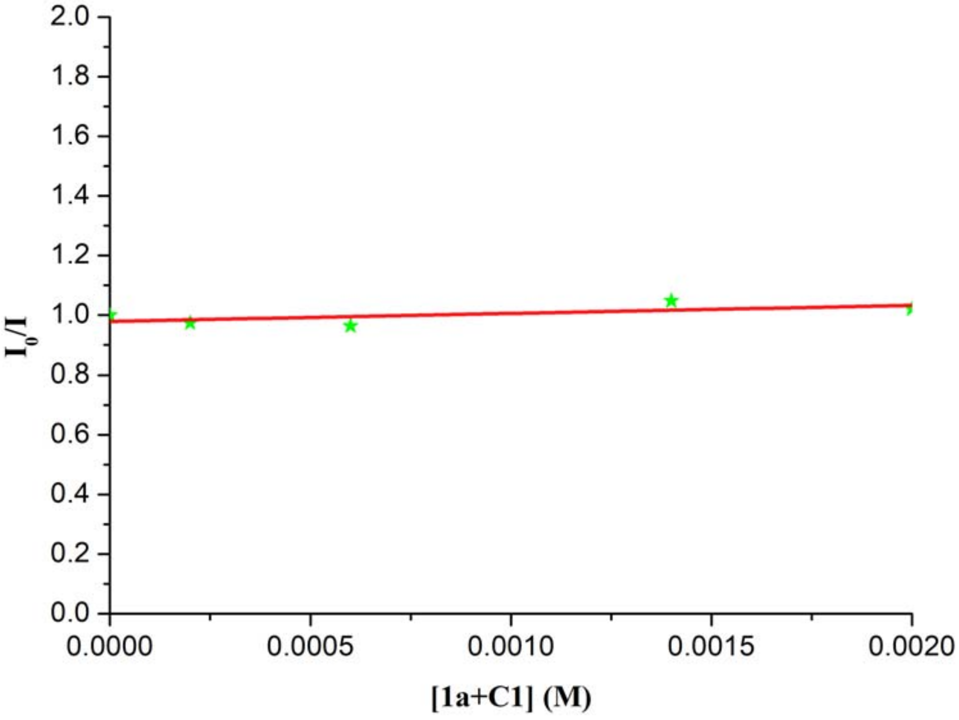

Fig. S3. Stern–Volmer quenching experiment of *DPZ and **1a** + **C1**.

(No measurable luminescence quenching in MeCN)

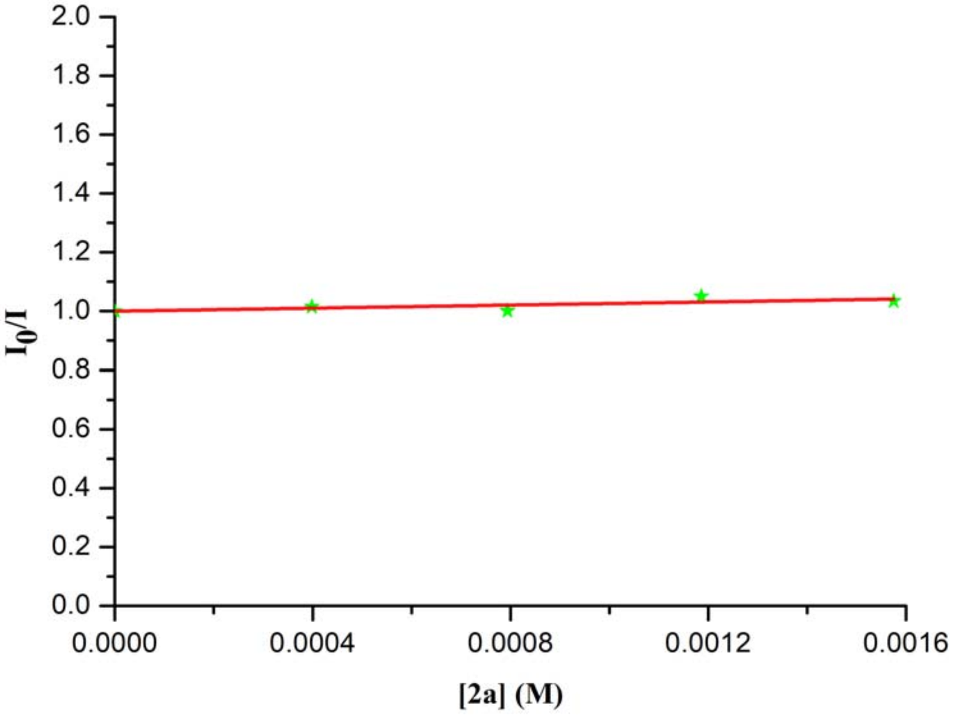

Fig. S4. Stern–Volmer quenching experiment of *DPZ and **2a**.
(No measurable luminescence quenching in MeCN)

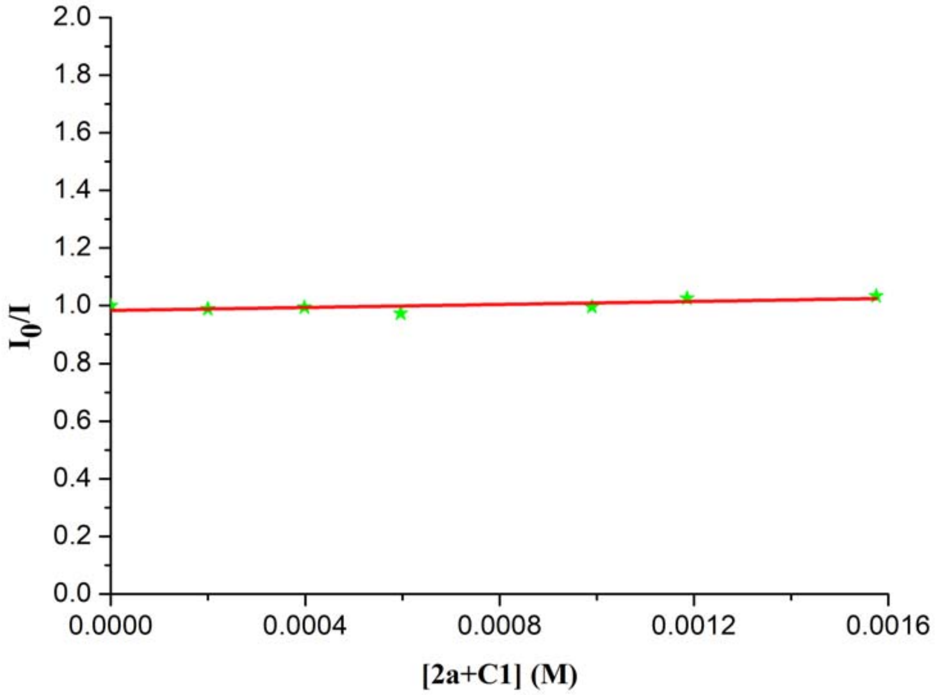

Fig. S5. Stern–Volmer quenching experiment of ***DPZ** and **2a + C1**.
(No measurable luminescence quenching in MeCN)

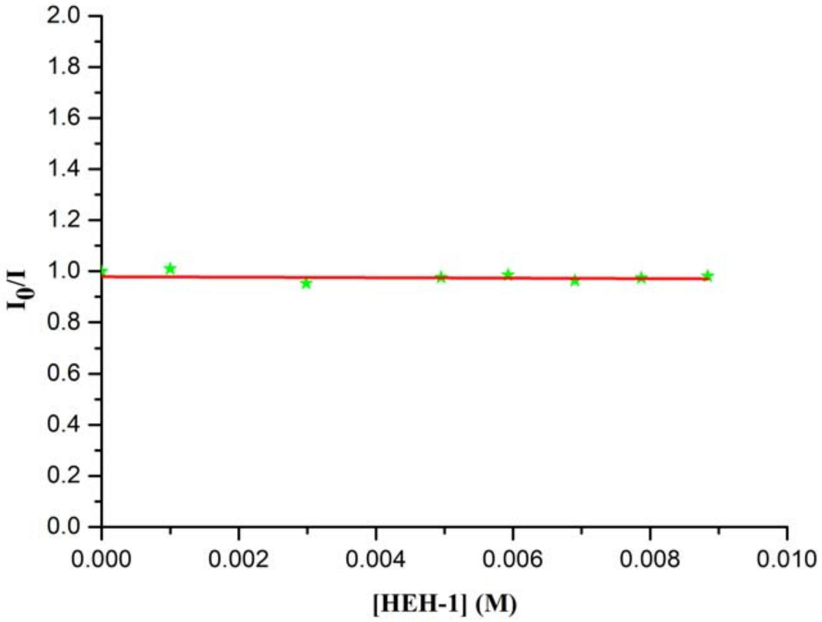

Fig. S6. Stern–Volmer quenching experiment of *DPZ and **HEH-1**.

(No measurable luminescence quenching in MeCN)

The Luminescence Quenching Experiments of HEH-1 (excitation wavelength = 418 nm)

Emission intensities were recorded on a spectrofluorometer. **HEH-1** solution was excited at 448 nm and the emission intensity at 450 nm was observed. A solution of **HEH-1** (1.0×10^{-3} M) was added to the appropriate amount of quencher in 3.0 mL volumetric flask under N_2. The solution was transferred to a 3.0 mL quartz cell and the emission spectrum of the sample was collected.

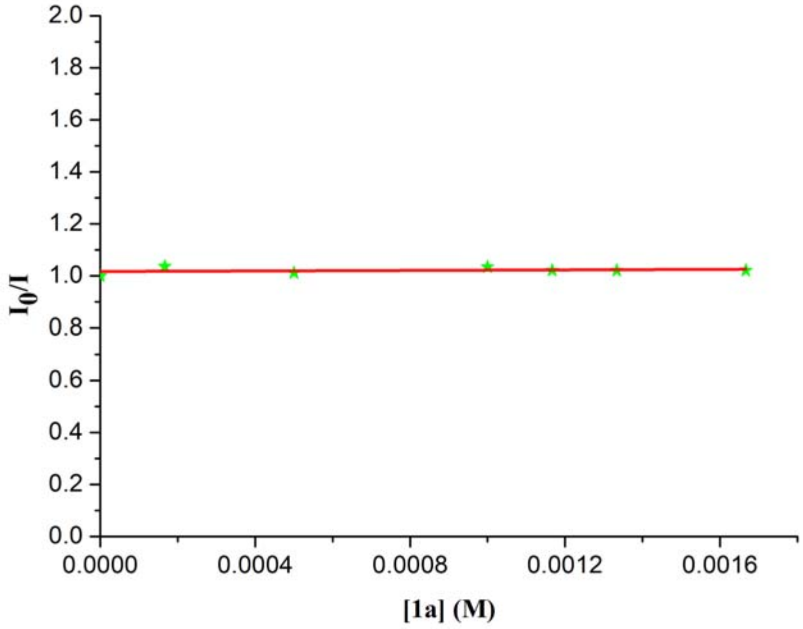

Fig. S7. Stern–Volmer quenching experiment of ***HEH-1** and **1a**.

(No measurable luminescence quenching in MeCN)

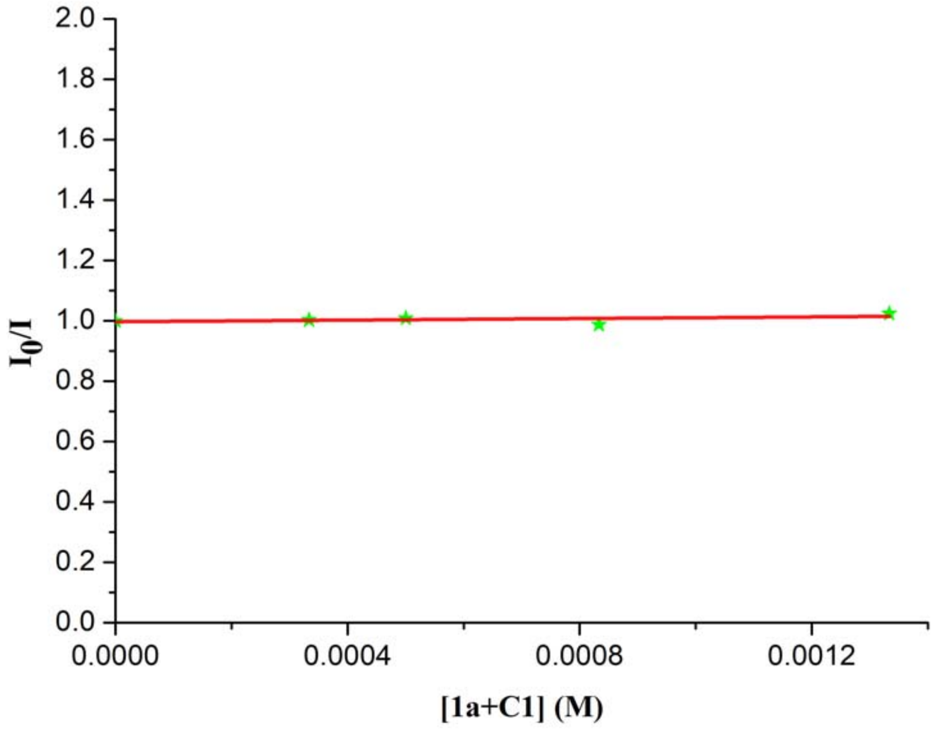

Fig. S8. Stern–Volmer quenching experiment of *HEH-1 and **1a** + **C1**.
(No measurable luminescence quenching in MeCN)

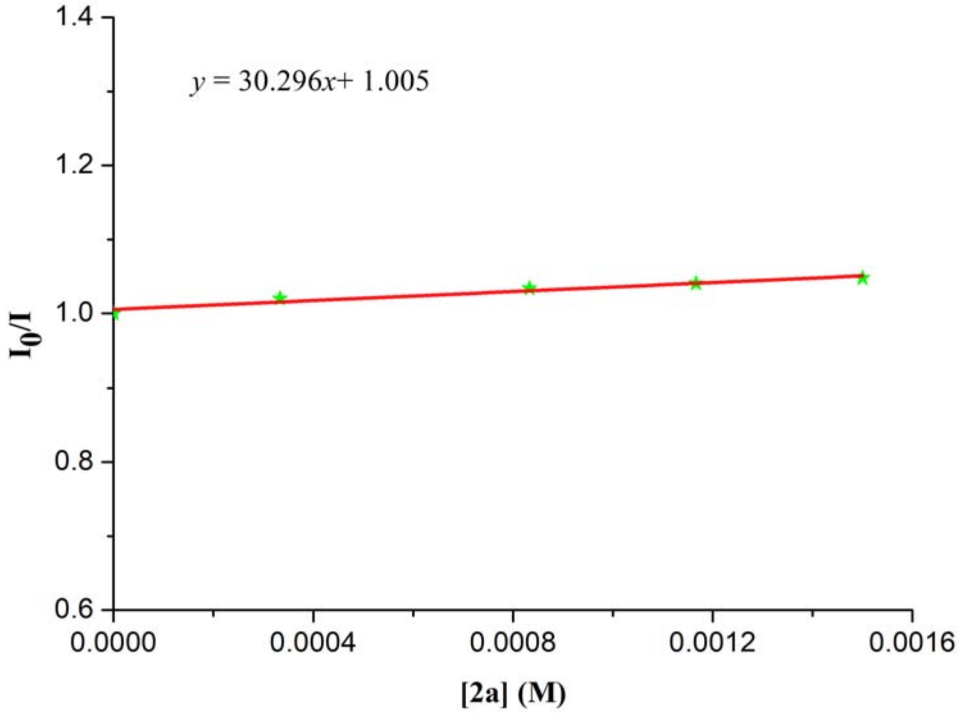

Fig. S9. Stern–Volmer quenching experiment of ***HEH-1** and **2a**.
(***HEH-1** can be quenched by **2a** in MeCN)

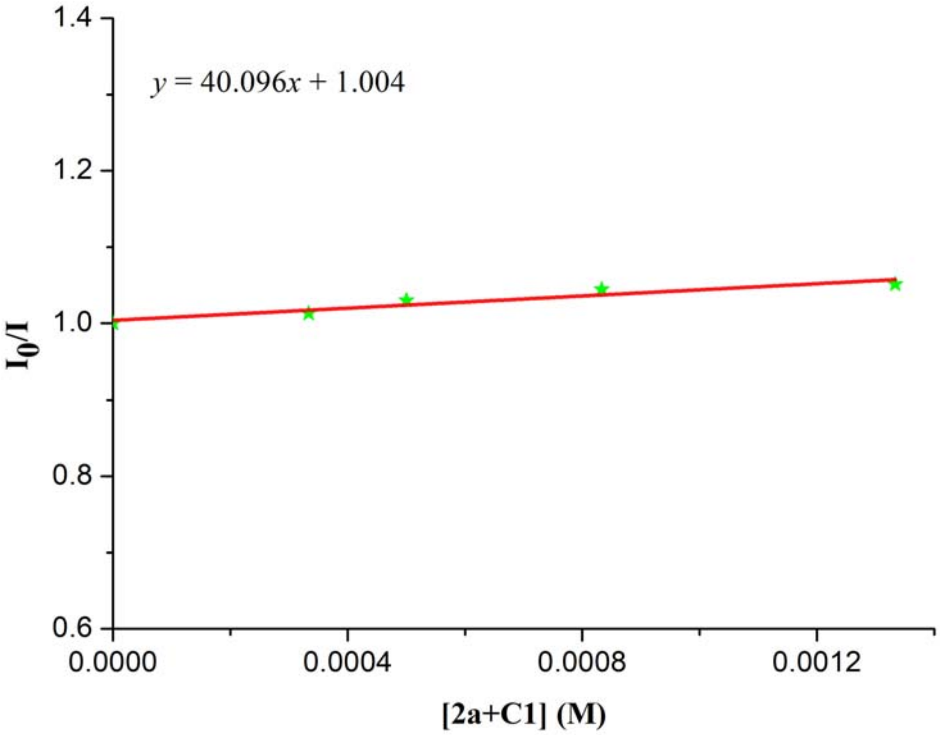

Fig. S10. Stern–Volmer quenching experiment of ***HEH-1** and **2a + C1**.
(Quenching of ***HEH-1** by **2a** could be improved by **C1** in MeCN)

(3) Cyclic voltammetry measurement

Electrochemical potentials were obtained with a standard set of conditions to main internal consistency. Cyclic voltammograms were collected with a potentiostat. Samples were prepared with 0.005 mmol of **HEH-1**, in 5 mL of 0.1 M tetrabutylammonium hexafluorophosphate in anhydrous acetonitrile. Samples were prepared with 50 mM / MeCN. Measurements employed a radium glassy carbon working electrode, platinum wire counter electrode, saturated KCl silver-silver chloride reference electrode. The obtained value was referenced to Ag/AgCl. The obtained value was referenced to Ag/AgCl and converted to SCE by adding 0.015 V.

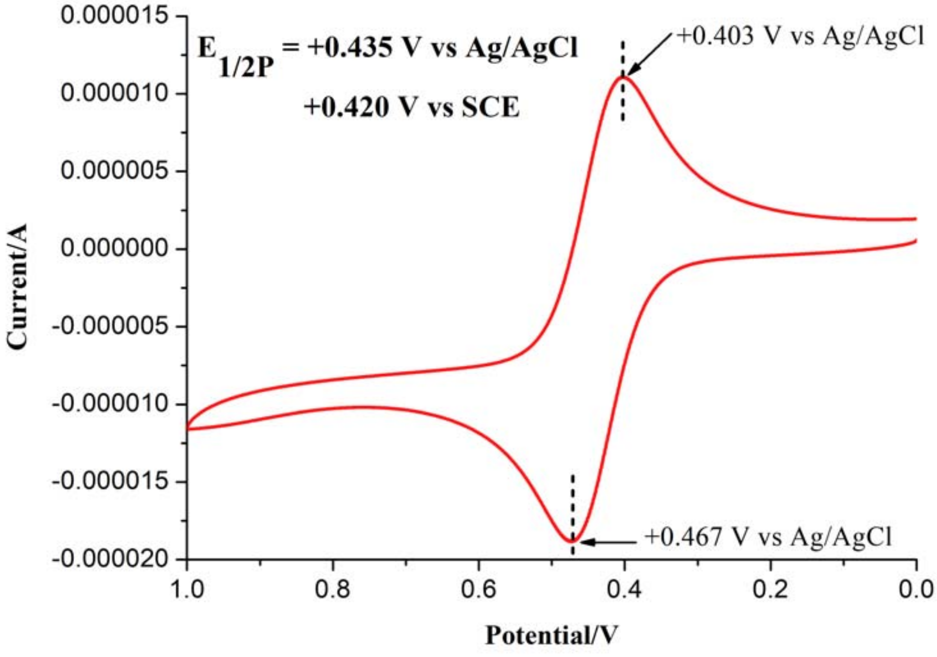

Fig. S11. Cyclic voltammogram of **Cp_2Fe** in MeCN

.

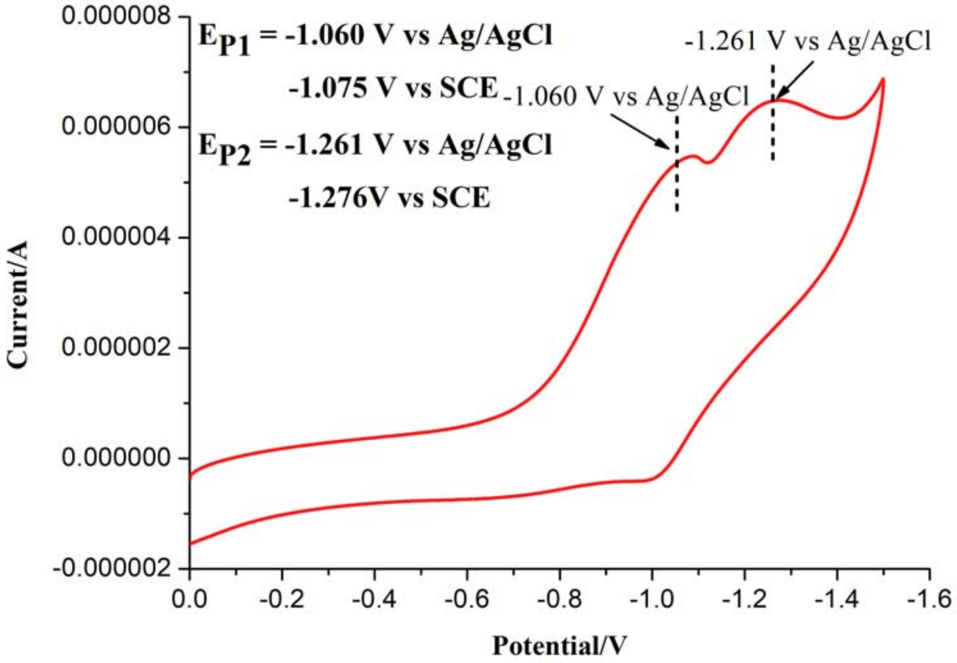

Fig. S12. Cyclic voltammogram of **1a** in MeCN.

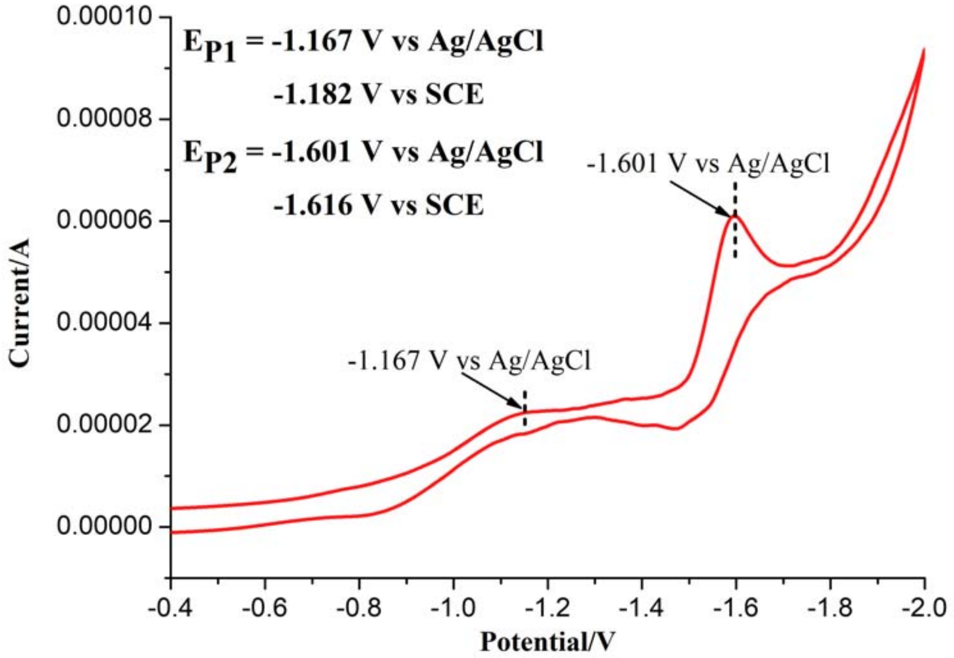

Fig. S13. Cyclic voltammogram of **2a** in MeCN.

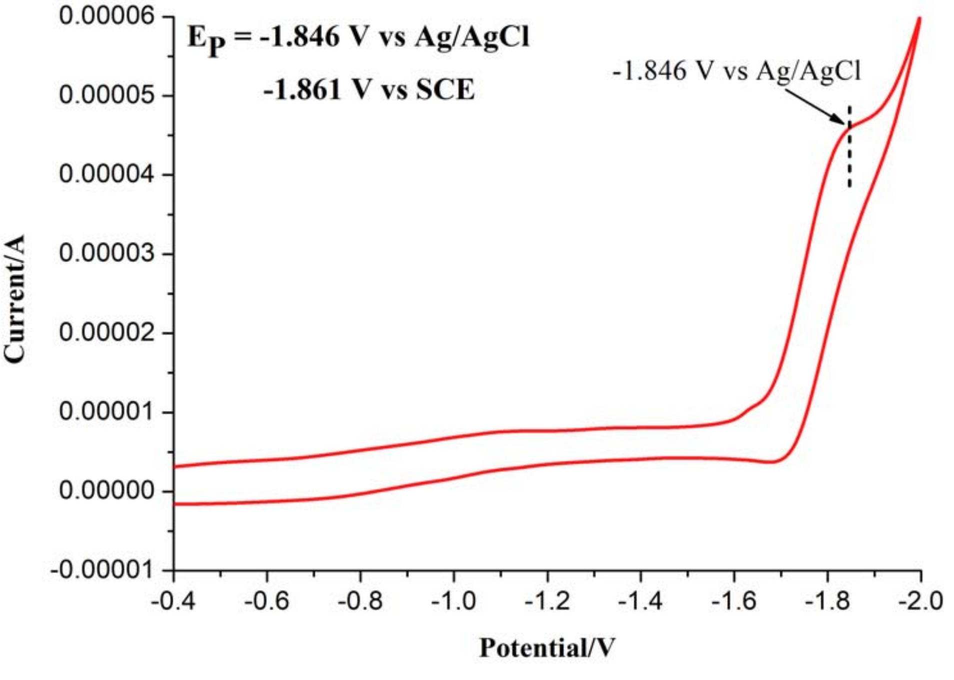

Fig. S14. Cyclic voltammogram of isonicotinonitrile (**2l**) in MeCN.

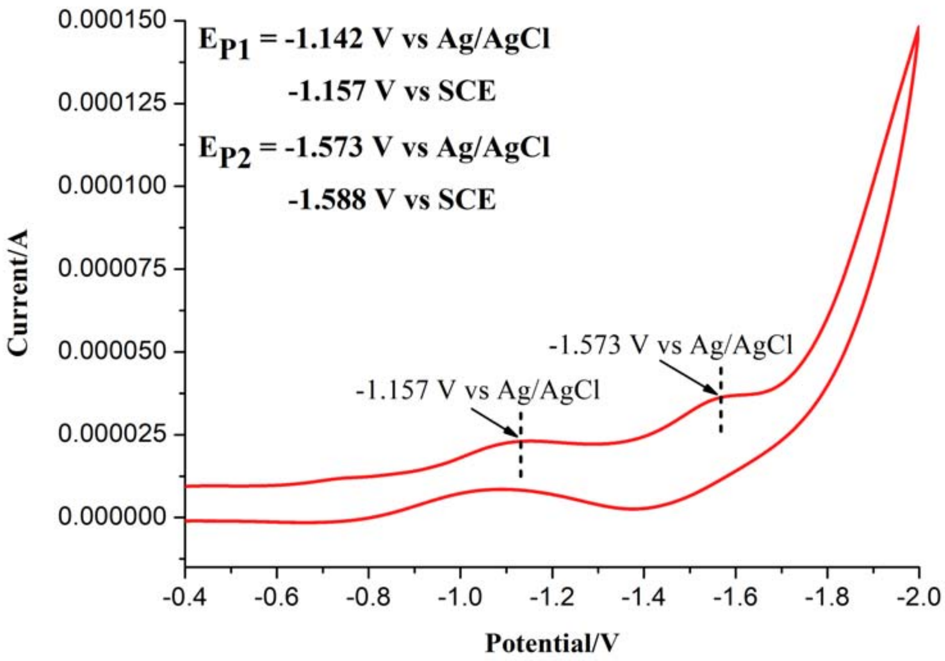

Fig. S15. Cyclic voltammogram of **5a** in MeCN.

(4) Control experiments-HRMS analysis

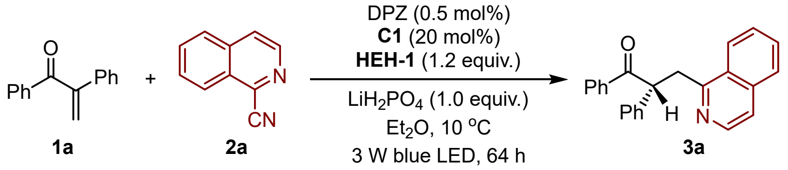

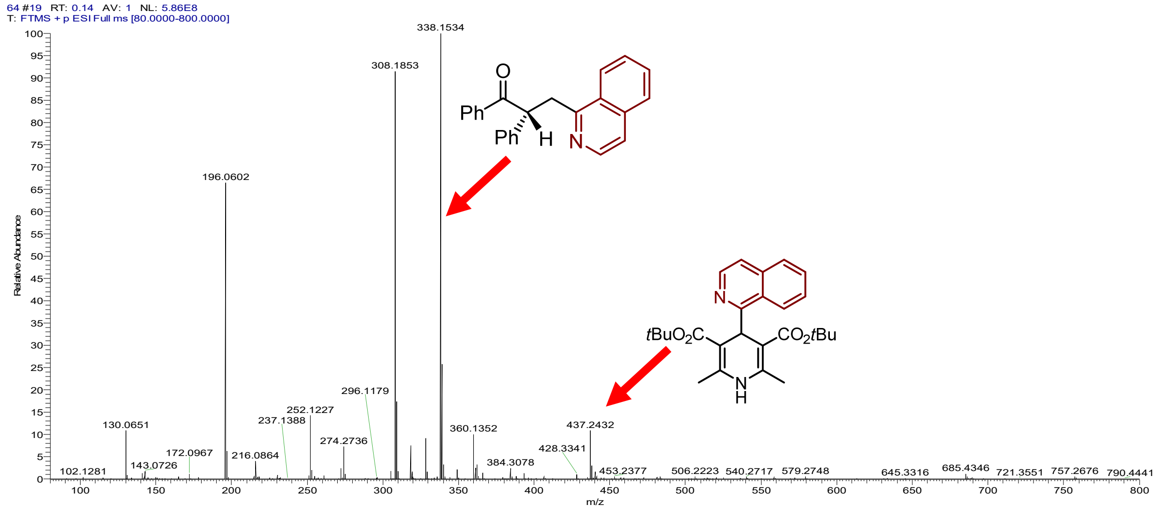

Fig. S16 HRMS analysis of the model reaction of **1a** and **2a**

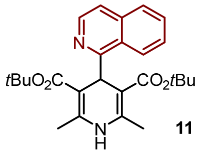

11

HRMS (ESI) m/z 437.2432 (M+H$^+$), calc. for $C_{26}H_{33}N_2O_4$ 437.2432.

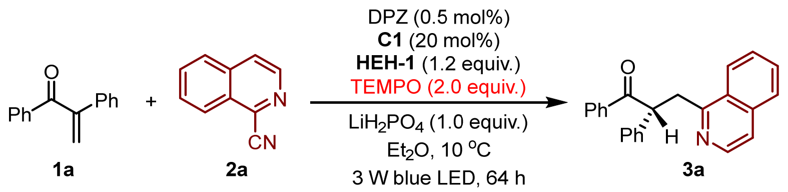

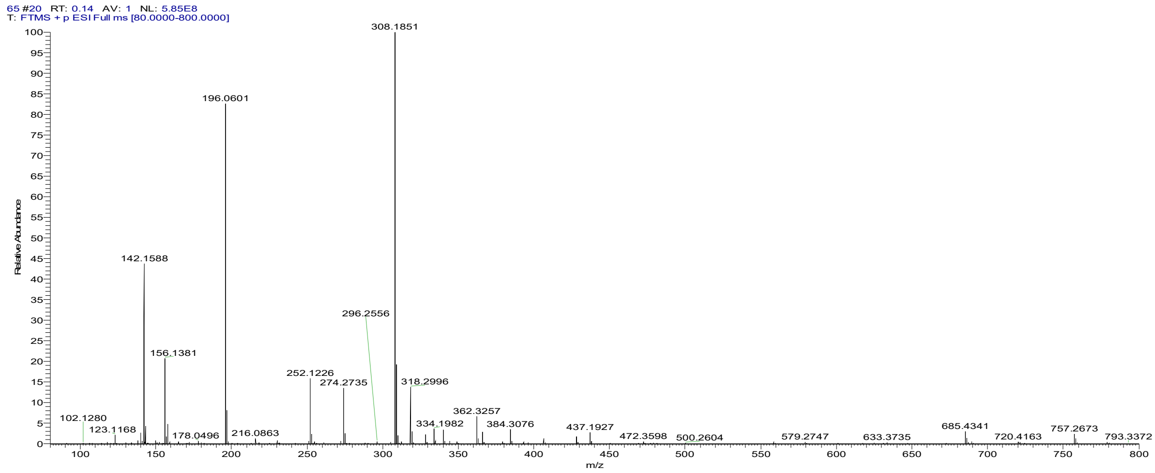

Fig. S17 HRMS analysis of the model reaction in the presence of TEMPO

1a (0.2 mmol), **2a** (0.10 mmol), DPZ (5.0 x 10^{-4} mmol), **C1** (0.02 mmol), **HE-1** (0.12 mmol), LiH_2PO_4 (0.1 mmol), TEMPO (0.2 mmol) and Et_2O were sequentially added into the Schlenk tube, and the reaction was degassed three times by freeze-pump-thaw method. Then the mixture worked at 10 °C under argon atmosphere with an irradiation of a 3 W blue LED (λ = 450～455 nm) for another 64 h. We found that the reaction was inhibited completely.

(5) Proposed mechanism for the reactions of 1 with 5

With the transformation of **1a** with **5a** as the representative:

In the reaction system, HCl should be generated after reduction of **5**. Due to the basicity of the resulting pyridine derivative (i.e., **Py'**) from **HEH-6** after twice single-electron oxidation processes, radical **20** could be neutralized by it to render radical **21**. As such, a ternary transition state **22** for radical coupling occurs, wherein quinoline will first capture the released HCl to generate a hydrogen-bonding donor. After enantioselective protonation, salt **23** was formed, and after treatment of $NaHCO_3$, product **6a** was obtained.

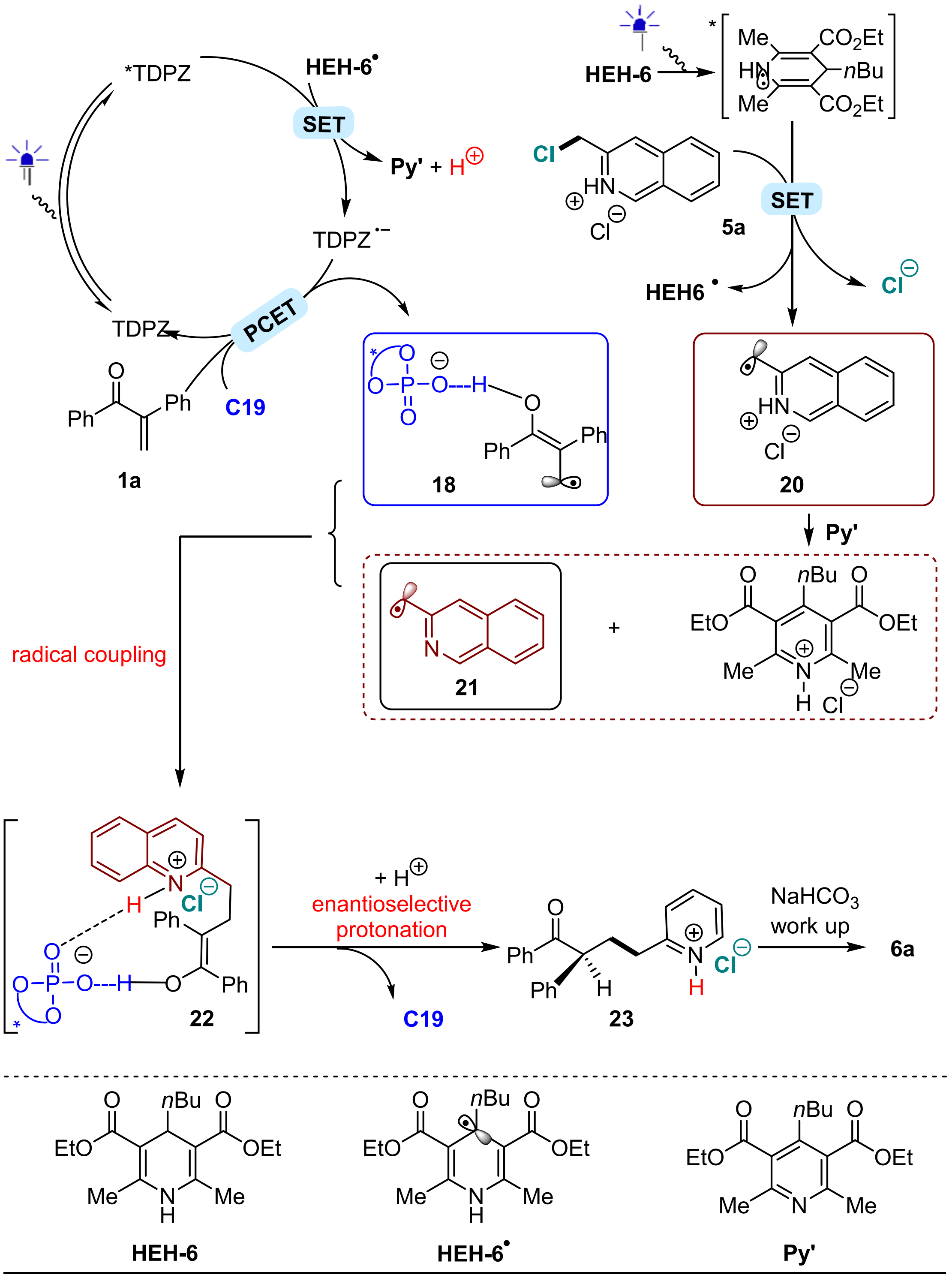
*TDPZ
HEH-6•
SET
Py' + H⊕
TDPZ•−
PCET
TDPZ
1a
C19
18
HEH-6
5a
SET
HEH6•
20
Py'
21
radical coupling
22
+ H⊕
enantioselective
protonation
C19
23
NaHCO3
work up
6a
HEH-6
HEH-6•
Py'

5. Synthetic applications

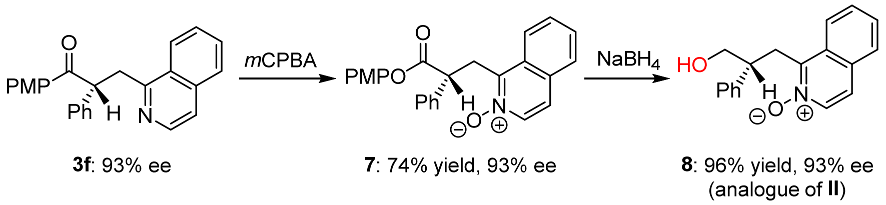

(*R*)-1-(3-(4-methoxyphenoxy)-3-oxo-2-phenylpropyl)isoquinoline 2-oxide (7): To a solution of **3n** (0.1 mmol, 1.0 equiv) in dry CH_2Cl_2 (1.0 mL), 3.0 equiv of *m*CPBA was added slowly at 0 °C under an argon atmosphere. The reaction mixture was stirred for 14 hours at 10 °C, then quenched with $NaHCO_3$ (aq.) and extracted with DCM (3 × 1.0 mL). The combined organic layers were dried over anhydrous Na_2SO_4, filtered and concentrated under reduced pressure. The residue was purified by silica gel column chromatography (1% MeOH in DCM) to give compound **9** as a colorless solid.

(*R*)-1-(3-hydroxy-2-phenylpropyl)isoquinoline 2-oxide (8): To a solution of **9** (0.1 mmol, 1.0 equiv) in dry MeOH (1.0 mL), 20.0 equiv of $NaBH_4$ was added slowly at 25 °C under an air atmosphere. The reaction mixture was stirred for 15 min at 60 °C and detected by TLC. After completion, organic solvents were evaporated, the residual oil was subjected to silica gel column chrotmatography (2% MeOH in DCM) to give compound **10** as a colorless solid.

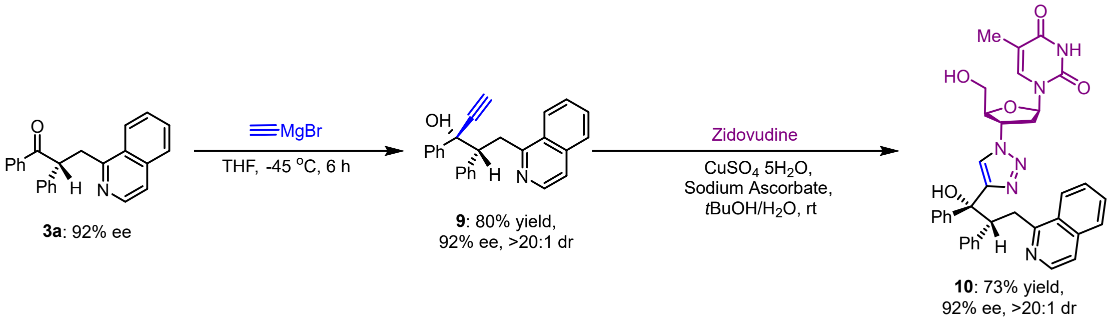

(3*S*,4*R*)-5-(isoquinolin-1-yl)-3,4-diphenylpent-1-yn-3-ol (9): To a solution of **3a** (0.1 mmol, 1.0 equiv) in dry tetrahydrofuran (1.0 mL), 3.0 equiv of ethynylmagnesium bromide (0.5 M in tetrahydrofuran) was added slowly at 0 °C under an argon atmosphere. After stirring for 5 hours at room temperature, 3.0 equiv of ethynylmagnesium bromide (0.5 M in tetrahydrofuran) was again added slowly at 0 °C under an argon atmosphere. After stirring for 10 hours at room temperature, the reaction mixture was quenched with NH_4Cl (aq) and extracted with DCM (3 × 1.0 mL). The combined organic layers were dried over anhydrous Na_2SO_4, filtered and concentrated under reduced pressure. The residue was purified by silica gel column chromatography (25% EtOAc in petroleum ether) to give the title compound **9** as a colorless solid.

Zidovudine derivative (10):To a solution of **9** (0.1 mmol, 1.0 equiv) in *tert*-Butanol (1.0 mL) and H_2O (1.0 mL), 0.1 equiv of $CuSO_4•5H_2O$ and 0.1 equiv of sodium ascorbate was added at room temperature, After stirring for 16 hours at room temperature, the reaction mixture was

quenched with NH_4Cl (aq) and extracted with DCM (3 × 1.0 mL). The combined organic layers were dried over anhydrous Na_2SO_4, filtered and concentrated under reduced pressure. The residue was purified by silica gel column chromatography (25% MeOH in DCM) to give the title compound **10** as a colorless solid.

6. Determination of the absolute configurations

Absolute configurations of products **3**, **7** and **8** are determined by X-ray structure analysis of product **3c**.

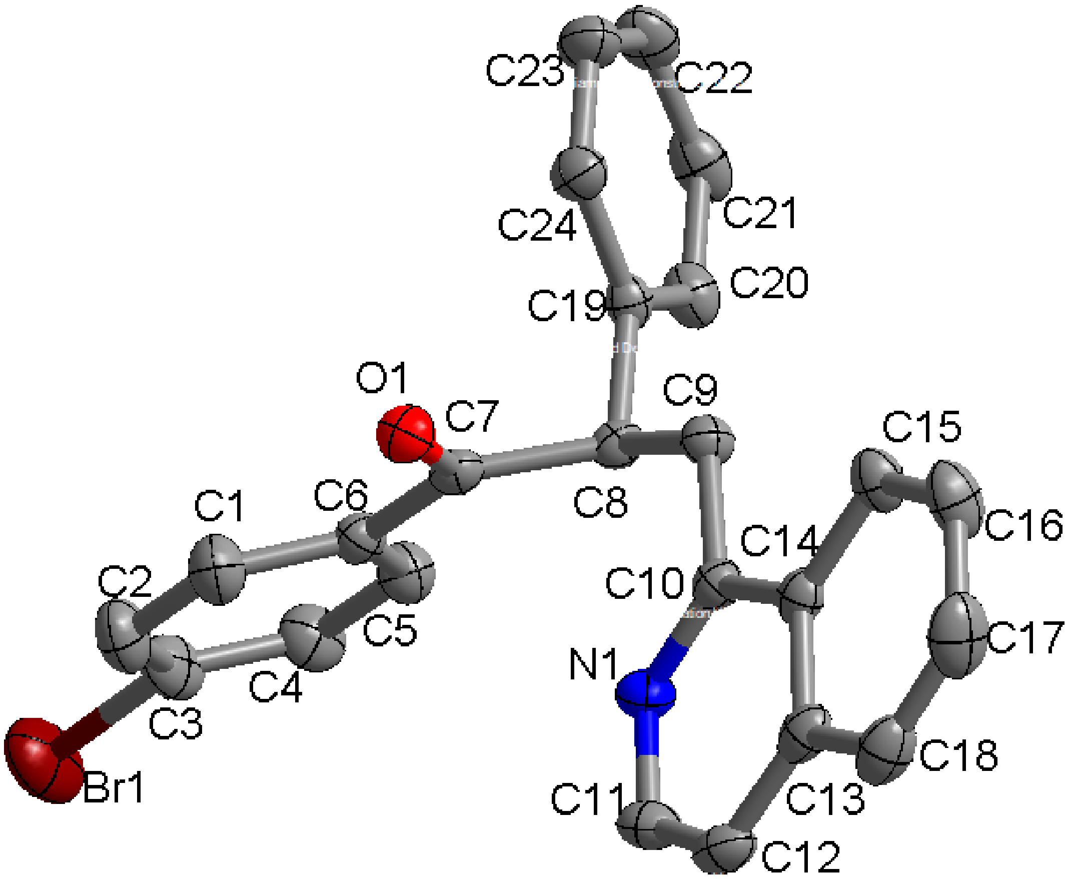

Fig. S18. Absolute configuration of product generated from the reduction of **3c** (CCDC 2272223)

Displacement ellipsoids are drawn at the 30% probability level.

(Solvents: ethyl acetate/hexane = 1:10)

Table S8 Crystal Data and Structure Refinement

Identification code	kmm20230301_auto
Empirical formula	$C_{24}H_{18}BrNO$
Formula weight	416.30
Temperature/K	293(2)
Crystal system	monoclinic
Space group	C2/c
a/Å	37.9124(15)
b/Å	10.2703(6)
c/Å	10.2751(4)
α/°	90
β/°	105.329(4)
γ/°	90
Volume/Å^3	3858.5(3)
Z	8
ρ_{calc}g/cm^3	1.433
μ/mm^{-1}	2.995

F(000)	1696.0
Crystal size/mm^3	0.13 × 0.11 × 0.1
Radiation	CuKα (λ = 1.54184)
2Θ range for data collection/°	8.944 to 134.156
Index ranges	$-45 \leq h \leq 45$, $-12 \leq k \leq 12$, $-12 \leq l \leq 8$
Reflections collected	13107
Independent reflections	3451 [R_{int} = 0.0357, R_{sigma} = 0.0310]
Data/restraints/parameters	3451/0/244
Goodness-of-fit on F^2	1.029
Final R indexes [I>=2σ (I)]	R_1 = 0.0471, wR_2 = 0.1261
Final R indexes [all data]	R_1 = 0.0561, wR_2 = 0.1363
Largest diff. peak/hole / e Å^{-3}	0.33/-0.59

Table S9 Fractional Atomic Coordinates (×10^4) and Equivalent Isotropic Displacement Parameters (Å^2×10^3) for kmm20230301_auto. U_{eq} is defined as 1/3 of of the trace of the orthogonalised U_{IJ} tensor.

Atom	*x*	*y*	*z*	U(eq)
Br1	5143.1(2)	7736.7(5)	4731.1(6)	99.8(2)
C1	4266.7(8)	7032(3)	6301(3)	58.9(7)
C2	4574.1(9)	7570(3)	6041(4)	67.5(8)
C3	4722.4(8)	6987(3)	5105(4)	64.8(8)
C4	4567.8(8)	5900(3)	4406(3)	61.3(7)
C5	4256.7(7)	5375(3)	4661(3)	51.5(6)
C6	4100.9(7)	5935(2)	5612(2)	42.9(5)
C7	3759.7(6)	5435(2)	5905(2)	38.1(5)
C8	3555.5(6)	4285(2)	5107(2)	38.0(5)
C9	3152.5(6)	4307(2)	5103(2)	41.0(5)
C10	2939.6(6)	5432(2)	4341(2)	38.0(5)
C11	2935.9(9)	7317(3)	3126(3)	52.1(6)
C12	2576.1(9)	7531(3)	2970(3)	52.4(7)
C13	2370.1(7)	6641(3)	3525(2)	46.9(6)
C14	2557.1(6)	5558(2)	4237(2)	40.0(5)
C15	2357.7(7)	4670(3)	4815(3)	50.9(6)
C16	1993.8(8)	4850(3)	4683(4)	65.0(8)
C17	1809.4(8)	5927(4)	3979(4)	71.4(9)
C18	1991.9(8)	6796(3)	3407(3)	60.5(8)
C19	3739.5(6)	3035(2)	5731(2)	39.1(5)
C20	3879.0(8)	2142(3)	4978(3)	50.3(6)
C21	4047.4(8)	1013(3)	5567(4)	61.6(8)
C22	4079.1(9)	760(3)	6915(4)	64.5(8)
C23	3932.9(9)	1619(3)	7653(3)	61.0(7)
C24	3765.8(8)	2749(3)	7072(3)	48.0(6)
N1	3121.3(6)	6270(2)	3795(2)	46.1(5)
O1	3655.5(5)	5914.5(18)	6823.4(17)	48.6(4)

Table S10 Anisotropic Displacement Parameters (Å^2×10^3) for kmm20230301_auto. The Anisotropic displacement factor exponent takes the form: $-2\pi^2[h^2a^{*2}U_{11}+2hka^*b^*U_{12}+\ldots]$.

Atom	U_{11}	U_{22}	U_{33}	U_{23}	U_{13}	U_{12}
Br1	58.7(3)	103.5(4)	145.8(5)	14.4(3)	41.9(3)	-22.0(2)
C1	53.8(16)	58.3(17)	64.7(17)	-10.2(13)	16.2(13)	-7.6(13)
C2	49.9(17)	63.4(19)	87(2)	-9.4(16)	14.8(16)	-13.5(14)
C3	41.4(14)	68.7(19)	85(2)	14.1(16)	18.3(14)	-7.7(14)
C4	48.0(15)	69.6(19)	72.9(18)	2.5(15)	27.9(14)	-0.1(14)
C5	44.8(13)	55.2(16)	56.4(14)	-1.0(12)	16.8(12)	-0.1(12)

C6	37.7(12)	44.5(13)	44.5(12)	4.2(10)	7.1(10)	1.5(10)
C7	39.0(11)	38.6(12)	35.8(11)	4.9(9)	8.5(9)	5.9(9)
C8	37.4(11)	43.9(13)	34.5(10)	0.9(9)	12.5(9)	2.1(9)
C9	38.4(12)	43.0(13)	42.8(12)	2.7(10)	13.0(10)	0.7(10)
C10	39.5(12)	40.5(12)	34.2(10)	-2.6(9)	9.9(9)	1.8(9)
C11	66.3(18)	47.4(14)	45.5(13)	10.8(11)	19.5(12)	4.1(12)
C12	66.1(18)	48.0(14)	40.1(12)	6.0(11)	8.8(12)	14.9(12)
C13	48.6(13)	51.1(14)	37.1(11)	-9.6(10)	4.6(10)	7.8(11)
C14	39.1(12)	40.5(12)	38.6(11)	-8.8(9)	7.4(9)	1.6(10)
C15	45.0(14)	43.1(14)	66.0(16)	-6.7(12)	17.2(12)	-0.7(11)
C16	46.1(15)	58.3(18)	95(2)	-14.0(16)	26.3(15)	-8.8(13)
C17	37.4(14)	79(2)	95(2)	-19.5(19)	11.2(15)	5.7(14)
C18	46.6(15)	65.8(19)	61.2(16)	-9.9(14)	0.2(13)	16.6(14)
C19	32.7(11)	38.5(12)	46.1(12)	-5.1(9)	10.8(9)	-1.5(9)
C20	50.2(14)	47.4(14)	58.9(15)	-10.3(12)	24.3(12)	-3.6(11)
C21	53.2(16)	42.7(15)	96(2)	-13.7(14)	31.3(15)	2.8(12)
C22	56.3(17)	41.9(15)	89(2)	4.7(14)	8.8(15)	9.3(13)
C23	73.0(19)	47.8(15)	54.7(15)	5.0(12)	3.8(14)	6.5(14)
C24	54.7(15)	43.2(14)	44.9(13)	-2.3(10)	10.9(11)	6.3(11)
N1	49.1(12)	46.5(12)	44.9(11)	5.5(9)	16.3(9)	1.4(9)
O1	55.5(10)	50.1(10)	43.9(9)	-5.6(7)	19.5(8)	2.0(8)

Table S11 Bond Lengths for kmm20230301_auto.

Atom	Atom	Length/Å	Atom	Atom	Length/Å
Br1	C3	1.899(3)	C11	C12	1.349(4)
C1	C2	1.378(5)	C11	N1	1.366(3)
C1	C6	1.389(4)	C12	C13	1.417(4)
C2	C3	1.373(5)	C13	C14	1.414(4)
C3	C4	1.373(5)	C13	C18	1.415(4)
C4	C5	1.384(4)	C14	C15	1.412(4)
C5	C6	1.393(4)	C15	C16	1.362(4)
C6	C7	1.494(3)	C16	C17	1.403(5)
C7	C8	1.526(3)	C17	C18	1.355(5)
C7	O1	1.219(3)	C19	C20	1.391(3)
C8	C9	1.527(3)	C19	C24	1.386(4)
C8	C19	1.520(3)	C20	C21	1.384(4)
C9	C10	1.506(3)	C21	C22	1.383(5)
C10	C14	1.432(3)	C22	C23	1.372(4)
C10	N1	1.317(3)	C23	C24	1.380(4)

Table S12 Bond Angles for kmm20230301_auto.

Atom	Atom	Atom	Angle/°	Atom	Atom	Atom	Angle/°
C2	C1	C6	121.2(3)	C11	C12	C13	119.7(2)
C3	C2	C1	119.0(3)	C14	C13	C12	117.5(2)
C2	C3	Br1	119.1(3)	C14	C13	C18	119.4(3)
C4	C3	Br1	119.3(3)	C18	C13	C12	123.1(3)
C4	C3	C2	121.6(3)	C13	C14	C10	117.9(2)
C3	C4	C5	119.1(3)	C15	C14	C10	123.7(2)
C4	C5	C6	120.8(3)	C15	C14	C13	118.3(2)
C1	C6	C5	118.3(2)	C16	C15	C14	120.8(3)
C1	C6	C7	118.4(2)	C15	C16	C17	120.8(3)
C5	C6	C7	123.3(2)	C18	C17	C16	120.1(3)
C6	C7	C8	120.07(19)	C17	C18	C13	120.7(3)

O1	C7	C6	119.4(2)	C20	C19	C8	121.5(2)
O1	C7	C8	120.4(2)	C24	C19	C8	120.2(2)
C7	C8	C9	110.82(19)	C24	C19	C20	118.4(2)
C19	C8	C7	108.43(18)	C21	C20	C19	120.6(3)
C19	C8	C9	111.15(19)	C22	C21	C20	120.2(3)
C10	C9	C8	114.26(19)	C23	C22	C21	119.4(3)
C14	C10	C9	120.1(2)	C22	C23	C24	120.6(3)
N1	C10	C9	117.0(2)	C23	C24	C19	120.8(3)
N1	C10	C14	122.9(2)	C10	N1	C11	118.1(2)
C12	C11	N1	123.9(3)				

Table S13 Torsion Angles for kmm20230301_auto.

A	B	C	D	Angle/°	A	B	C	D	Angle/°
Br1	C3	C4	C5	-178.6(2)	C10	C14	C15	C16	179.7(3)
C1	C2	C3	Br1	179.4(3)	C11	C12	C13	C14	0.5(4)
C1	C2	C3	C4	1.3(5)	C11	C12	C13	C18	180.0(3)
C1	C6	C7	C8	176.1(2)	C12	C11	N1	C10	-1.3(4)
C1	C6	C7	O1	-7.1(3)	C12	C13	C14	C10	-0.3(3)
C2	C1	C6	C5	1.0(4)	C12	C13	C14	C15	179.1(2)
C2	C1	C6	C7	-177.6(3)	C12	C13	C18	C17	-178.8(3)
C2	C3	C4	C5	-0.5(5)	C13	C14	C15	C16	0.4(4)
C3	C4	C5	C6	0.0(5)	C14	C10	N1	C11	1.4(3)
C4	C5	C6	C1	-0.2(4)	C14	C13	C18	C17	0.7(4)
C4	C5	C6	C7	178.3(2)	C14	C15	C16	C17	-0.5(5)
C5	C6	C7	C8	-2.4(3)	C15	C16	C17	C18	0.8(5)
C5	C6	C7	O1	174.4(2)	C16	C17	C18	C13	-0.9(5)
C6	C1	C2	C3	-1.5(5)	C18	C13	C14	C10	-179.9(2)
C6	C7	C8	C9	-155.3(2)	C18	C13	C14	C15	-0.4(3)
C6	C7	C8	C19	82.4(2)	C19	C8	C9	C10	-172.48(19)
C7	C8	C9	C10	66.9(2)	C19	C20	C21	C22	-0.1(4)
C7	C8	C19	C20	-122.1(2)	C20	C19	C24	C23	1.3(4)
C7	C8	C19	C24	59.1(3)	C20	C21	C22	C23	1.9(5)
C8	C9	C10	C14	178.94(19)	C21	C22	C23	C24	-2.1(5)
C8	C9	C10	N1	-1.2(3)	C22	C23	C24	C19	0.5(5)
C8	C19	C20	C21	179.6(2)	C24	C19	C20	C21	-1.5(4)
C8	C19	C24	C23	-179.8(3)	N1	C10	C14	C13	-0.6(3)
C9	C8	C19	C20	115.8(2)	N1	C10	C14	C15	180.0(2)
C9	C8	C19	C24	-63.0(3)	N1	C11	C12	C13	0.4(4)
C9	C10	C14	C13	179.2(2)	O1	C7	C8	C9	27.9(3)
C9	C10	C14	C15	-0.2(3)	O1	C7	C8	C19	-94.3(2)
C9	C10	N1	C11	-178.4(2)					

Table S14 Hydrogen Atom Coordinates (Å×10^4) and Isotropic Displacement Parameters (Å^2×10^3) for kmm20230301_auto.

Atom	*x*	*y*	*z*	U(eq)
H1	4168	7411	6949	71
H2	4679	8317	6493	81
H4	4671	5521	3770	74
H5	4150	4638	4192	62
H8	3573	4344	4174	46
H9A	3039	3501	4708	49
H9B	3136	4338	6029	49
H11	3066	7917	2757	63
H12	2464	8259	2500	63

H15	2476	3955	5292	61
H16	1866	4251	5064	78
H17	1562	6045	3905	86
H18	1867	7501	2931	73
H20	3859	2306	4071	60
H21	4139	422	5054	74
H22	4199	13	7318	77
H23	3946	1438	8552	73
H24	3670	3326	7586	58

Absolute configuration of product **10** is determined by X-ray structure analysis of product **9**.

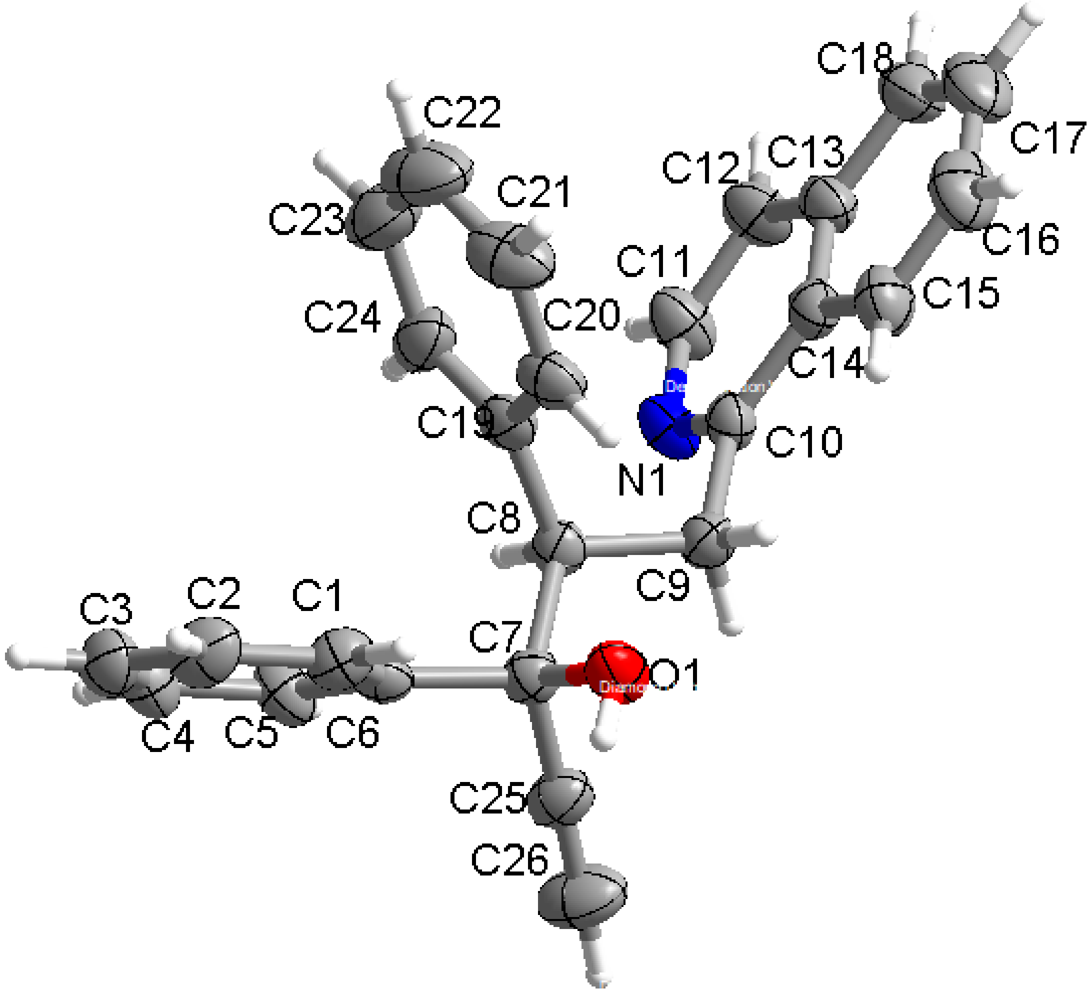

Fig. S19. Absolute configuration of product generated from the reduction of **9**

(CCDC 2272224)

Displacement ellipsoids are drawn at the 30% probability level.

(Solvents: ethyl acetate/hexane = 1:10)

Table S15 Crystal Data and Structure Refinement

Identification code	KM48165
Empirical formula	$C_{26}H_{21}NO$
Formula weight	363.44
Temperature/K	293(2)

Crystal system	monoclinic
Space group	C2
a/Å	24.5950(8)
b/Å	10.3051(3)
c/Å	18.3746(6)
α/°	90
β/°	109.663(4)
γ/°	90
Volume/Å^3	4385.6(3)
Z	8
ρ_{calc}g/cm^3	1.101
μ/mm^{-1}	0.516
F(000)	1536.0
Crystal size/mm^3	0.17 × 0.1 × 0.07
Radiation	CuKα (λ = 1.54184)
2Θ range for data collection/°	7.624 to 134.118
Index ranges	$-29 \le h \le 27$, $-12 \le k \le 12$, $-16 \le l \le 21$
Reflections collected	15383
Independent reflections	7799 [R_{int} = 0.0265, R_{sigma} = 0.0369]
Data/restraints/parameters	7799/1/497
Goodness-of-fit on F^2	1.015
Final R indexes [I>=2σ (I)]	R_1 = 0.0467, wR_2 = 0.1152
Final R indexes [all data]	R_1 = 0.0591, wR_2 = 0.1254
Largest diff. peak/hole / e Å^{-3}	0.12/-0.18
Flack parameter	0.33(17)

Table S16 Fractional Atomic Coordinates (×10^4) and Equivalent Isotropic Displacement Parameters (Å^2×10^3) for KM48165. U_{eq} is defined as 1/3 of of the trace of the orthogonalised U_{IJ} tensor.

Atom	*x*	*y*	*z*	U(eq)
O1'	7614.2(9)	-695.0(19)	9676.4(15)	59.7(5)
N1'	6284.2(11)	3142(3)	9692.2(19)	64.7(7)
C1F	6718(4)	-159(6)	8236(3)	75.7(16)
C1E	6563(3)	-438(7)	7453(4)	106(3)
C1D	6596(4)	521(9)	6937(3)	144(4)
C1C	6785(4)	1759(8)	7206(3)	132(4)
C1B	6940(3)	2038(6)	7990(4)	91(3)
C1A	6907(4)	1079(7)	8505(3)	60.2(13)
C1'	8485.3(16)	2137(3)	9536(2)	64.2(8)
C2'	8880.0(19)	2458(4)	9181(3)	82.6(12)
C3'	8941.5(19)	1694(4)	8604(2)	80.8(11)
C4'	8602.4(18)	613(4)	8377(2)	76.9(10)
C5'	8209.2(15)	289(3)	8738(2)	63.6(9)
C6'	8152.7(12)	1039(3)	9327.0(18)	50.1(6)
C7'	7726.0(12)	658(3)	9739.2(18)	48.1(6)
C8'	7145.5(12	1379(3)	9377.0(17)	49.1(6)

	)			
C9'	6724.7(12)	1052(3)	9811(2)	56.2(7)
C10'	6189.1(12)	1887(3)	9568.1(18)	51.0(7)
C11'	5824.7(16)	3966(4)	9509(3)	75.9(11)
C12'	5274.4(15)	3578(4)	9219(3)	74.4(10)
C13'	5150.9(14)	2257(4)	9084(2)	61.2(8)
C14'	5619.5(13)	1375(3)	9256.7(17)	53.4(7)
C15'	5502.1(17)	37(4)	9121(2)	72.4(10)
C16'	4947(2)	-381(5)	8829(3)	98.1(15)
C17'	4482.7(19)	482(6)	8649(3)	102.0(17)
C18'	4582.1(16)	1760(5)	8768(3)	83.8(12)
C19'	6852(9)	1300(20)	8536(7)	60.2(13)
C20'	6629(9)	137(17)	8173(10)	75.7(16)
C21'	6345(8)	108(17)	7379(10)	106(3)
C22'	6284(9)	1240(20)	6948(7)	144(4)
C23'	6507(9)	2403(19)	7311(9)	132(4)
C24'	6791(8)	2431(17)	8105(10)	91(3)
C25'	7983.0(13)	991(3)	10576.1(19)	56.3(7)
C26'	8188.4(16)	1193(5)	11241(2)	81.3(11)
O1	7532.1(11)	7899(2)	5507.6(16)	72.9(7)
N1	6441.9(13)	4274(3)	3689.2(16)	67.0(7)
C1	7690.4(16)	6946(4)	6999(2)	66.8(9)
C2	7768(2)	6500(5)	7735(2)	82.9(12)
C3	7882.3(19)	5224(5)	7924(2)	80.4(11)
C4	7933.5(18)	4386(4)	7376(2)	79.8(11)
C5	7863.4(17)	4821(4)	6641(2)	70.5(9)
C6	7734.5(13)	6107(3)	6437.4(18)	54.2(7)
C7	7613.4(14)	6545(3)	5602(2)	58.3(7)
C8	7046.7(13)	5872(3)	5081.1(17)	55.3(7)
C9	6899.0(15)	6257(4)	4227.2(19)	64.6(8)
C10	6374.1(14)	5531(3)	3720.3(18)	56.9(7)
C11	5989.4(18)	3538(4)	3259(2)	80.5(11)
C12	5469.4(18	4030(4)	2854(2)	79.6(11)

	)			
C13	5377.5(15)	5381(4)	2858(2)	68.1(9)
C14	5838.4(14)	6154(4)	3307.3(18)	61.1(8)
C15	5752(2)	7509(4)	3312(2)	80.3(11)
C16	5228(3)	8042(6)	2890(3)	103.6(16)
C17	4779(2)	7266(7)	2448(3)	105.6(17)
C18	4844.3(18)	5969(6)	2424(2)	88.9(13)
C19A	6533(6)	6138(19)	5365(6)	61.9(13)
C20A	6276(9)	7356(17)	5283(10)	75(3)
C21A	5819(9)	7573(17)	5552(11)	108(4)
C22A	5619(7)	6570(20)	5902(10)	136(5)
C23A	5876(7)	5360(20)	5984(10)	118(5)
C24A	6333(6)	5138(17)	5715(9)	86(3)
C19	6555(10)	6040(30)	5382(11)	61.9(13)
C20	6349(14)	7250(30)	5494(14)	75(3)
C21	5859(14)	7350(30)	5705(17)	108(4)
C22	5574(11)	6230(40)	5806(16)	136(5)
C23	5779(11)	5020(30)	5694(13)	118(5)
C24	6270(10)	4920(30)	5482(11)	86(3)
C25	8100.5(16)	6127(4)	5352(2)	72.9(9)
C26	8496(2)	5831(7)	5176(3)	111.1(18)

Table S17 Anisotropic Displacement Parameters (Å^2×10^3) for KM48165. The Anisotropic displacement factor exponent takes the form: -2π^2[h^{2}a*^{2}U$_{11}$+2hka*b*U$_{12}$+…].

Atom	U_{11}	U_{22}	U_{33}	U_{23}	U_{13}	U_{12}
O1'	51.8(11)	41.8(10)	86.1(16)	8.7(10)	24.1(11)	0.3(9)
N1'	43.8(13)	54.4(15)	89(2)	-6.1(14)	13.4(13)	-2.5(11)
C1F	72(4)	84(4)	64(2)	-4(3)	14(2)	-9(3)
C1E	110(7)	116(7)	72(4)	-18(5)	6(4)	-3(5)
C1D	157(10)	197(12)	60(3)	1(5)	14(5)	-18(8)
C1C	141(9)	167(10)	73(4)	46(5)	18(5)	-15(7)
C1B	97(5)	99(5)	68(3)	29(3)	18(3)	1(4)
C1A	53(2)	67(3)	59.7(19)	7.0(19)	16.8(15)	-1(2)
C1'	80(2)	50.7(17)	72(2)	-7.9(15)	38.9(18)	-12.2(16)
C2'	95(3)	71(2)	97(3)	-8(2)	52(2)	-31(2)
C3'	84(3)	93(3)	83(3)	-1(2)	51(2)	-19(2)
C4'	85(3)	85(2)	74(2)	-11(2)	44(2)	-2(2)
C5'	66(2)	59.1(19)	73(2)	-11.7(16)	33.7(18)	-9.2(15)
C6'	49.8(15)	43.8(14)	58.7(16)	4.0(13)	20.9(13)	4.1(12)
C7'	49.1(15)	41.0(13)	57.1(17)	2.0(12)	21.6(13)	0.1(12)
C8'	48.3(14)	42.3(14)	58.5(16)	2.6(12)	20.5(13)	-1.6(12)
C9'	47.3(15)	55.0(16)	67.5(19)	10.9(15)	20.9(13)	2.9(13)
C10'	43.7(14)	56.4(17)	53.1(16)	3.4(13)	16.5(12)	-1.7(13)
C11'	54.0(19)	57.6(19)	111(3)	-3.8(19)	21(2)	4.8(15)
C12'	48.7(19)	77(2)	93(3)	10(2)	17.8(18)	16.1(17)
C13'	44.4(15)	79(2)	60.5(19)	11.8(16)	17.9(14)	-1.5(15)
C14'	48.7(15)	64.9(18)	50.0(15)	4.2(14)	21.1(12)	-9.3(14)

C15'	71(2)	68(2)	87(3)	-2.2(19)	37(2)	-17.6(18)
C16'	84(3)	87(3)	128(4)	-16(3)	42(3)	-43(3)
C17'	60(2)	124(4)	113(4)	8(3)	18(2)	-43(3)
C18'	45.6(18)	112(3)	88(3)	20(2)	15.4(18)	-8(2)
C19'	53(2)	67(3)	59.7(19)	7.0(19)	16.8(15)	-1(2)
C20'	72(4)	84(4)	64(2)	-4(3)	14(2)	-9(3)
C21'	110(7)	116(7)	72(4)	-18(5)	6(4)	-3(5)
C22'	157(10)	197(12)	60(3)	1(5)	14(5)	-18(8)
C23'	141(9)	167(10)	73(4)	46(5)	18(5)	-15(7)
C24'	97(5)	99(5)	68(3)	29(3)	18(3)	1(4)
C25'	45.0(14)	67.1(19)	59(2)	4.9(15)	19.8(13)	2.9(14)
C26'	63(2)	121(3)	58(2)	-2(2)	17.7(17)	4(2)
O1	68.2(15)	59.3(13)	77.3(16)	2.6(12)	6.1(12)	-14.3(11)
N1	64.2(17)	69.3(18)	54.5(16)	-2.8(13)	2.7(13)	7.6(14)
C1	70(2)	63.1(19)	72(2)	-11.6(17)	29.7(18)	-8.3(17)
C2	92(3)	93(3)	70(2)	-27(2)	35(2)	-21(2)
C3	82(3)	99(3)	57(2)	0(2)	20.6(19)	-11(2)
C4	82(3)	78(2)	68(2)	12(2)	10.3(19)	10(2)
C5	75(2)	72(2)	56.8(19)	-8.4(17)	11.3(16)	17.0(18)
C6	43.6(14)	59.3(16)	55.2(16)	-8.9(14)	10.9(12)	-5.9(13)
C7	55.8(17)	58.3(17)	59.1(18)	-5.2(14)	17.3(14)	-7.5(14)
C8	58.8(17)	56.5(18)	48.4(16)	-3.1(13)	14.9(13)	-9.0(14)
C9	60.7(18)	74(2)	58.5(18)	5.4(16)	18.6(15)	-10.0(16)
C10	61.8(18)	65.1(19)	42.5(16)	4.9(13)	15.8(14)	2.3(15)
C11	81(3)	71(2)	72(3)	-4.4(19)	2(2)	4(2)
C12	71(2)	87(3)	66(2)	-13(2)	4.2(19)	-8(2)
C13	57.1(19)	96(3)	48.5(18)	3.2(17)	13.6(15)	6.8(18)
C14	63.4(18)	76(2)	47.0(16)	7.6(16)	22.7(14)	7.9(17)
C15	88(3)	77(2)	74(2)	12(2)	25(2)	15(2)
C16	113(4)	95(3)	105(4)	21(3)	40(3)	40(3)
C17	82(3)	137(5)	95(3)	26(3)	26(3)	44(3)
C18	63(2)	125(4)	74(3)	9(3)	16.6(18)	15(2)
C19A	53.2(19)	81(4)	46.1(17)	-4.9(19)	8.9(14)	-8(2)
C20A	59(5)	100(4)	56(9)	-14(5)	6(6)	3(4)
C21A	81(4)	152(8)	88(8)	-34(6)	24(5)	20(5)
C22A	81(4)	238(16)	102(6)	-20(7)	46(4)	-14(7)
C23A	93(7)	178(13)	96(11)	5(9)	49(8)	-27(6)
C24A	80(4)	116(7)	60(8)	4(6)	23(6)	-25(4)
C19	53.2(19)	81(4)	46.1(17)	-4.9(19)	8.9(14)	-8(2)
C20	59(5)	100(4)	56(9)	-14(5)	6(6)	3(4)
C21	81(4)	152(8)	88(8)	-34(6)	24(5)	20(5)
C22	81(4)	238(16)	102(6)	-20(7)	46(4)	-14(7)
C23	93(7)	178(13)	96(11)	5(9)	49(8)	-27(6)
C24	80(4)	116(7)	60(8)	4(6)	23(6)	-25(4)
C25	69(2)	88(2)	64(2)	-11.8(19)	25.5(17)	-18(2)
C26	86(3)	158(5)	106(4)	-30(4)	54(3)	-15(3)

Table S18 Bond Lengths for KM48165.

Atom	Atom	Length/Å	Atom	Atom	Length/Å
O1'	C7'	1.418(3)	O1	C7	1.412(4)
N1'	C10'	1.320(4)	N1	C10	1.310(5)
N1'	C11'	1.362(5)	N1	C11	1.359(5)
C1F	C1E	1.3900	C1	C2	1.379(6)

C1F	C1A	1.3900	C1	C6	1.379(5)
C1E	C1D	1.3900	C2	C3	1.365(6)
C1D	C1C	1.3900	C3	C4	1.364(6)
C1C	C1B	1.3900	C4	C5	1.377(5)
C1B	C1A	1.3900	C5	C6	1.385(5)
C1A	C8'	1.541(6)	C6	C7	1.529(5)
C1'	C2'	1.380(5)	C7	C8	1.563(4)
C1'	C6'	1.373(4)	C7	C25	1.484(5)
C2'	C3'	1.369(6)	C8	C9	1.539(4)
C3'	C4'	1.370(6)	C8	C19A	1.544(15)
C4'	C5'	1.385(5)	C8	C19	1.50(2)
C5'	C6'	1.375(5)	C9	C10	1.512(5)
C6'	C7'	1.537(4)	C10	C14	1.433(5)
C7'	C8'	1.547(4)	C11	C12	1.344(6)
C7'	C25'	1.493(4)	C12	C13	1.411(6)
C8'	C9'	1.542(4)	C13	C14	1.405(5)
C8'	C19'	1.471(12)	C13	C18	1.421(5)
C9'	C10'	1.510(4)	C14	C15	1.413(6)
C10'	C14'	1.424(4)	C15	C16	1.375(6)
C11'	C12'	1.338(5)	C16	C17	1.384(8)
C12'	C13'	1.399(5)	C17	C18	1.348(8)
C13'	C14'	1.417(5)	C19A	C20A	1.3900
C13'	C18'	1.418(5)	C19A	C24A	1.3900
C14'	C15'	1.414(5)	C20A	C21A	1.3900
C15'	C16'	1.358(6)	C21A	C22A	1.3900
C16'	C17'	1.397(7)	C22A	C23A	1.3900
C17'	C18'	1.343(7)	C23A	C24A	1.3900
C19'	C20'	1.3900	C19	C20	1.3900
C19'	C24'	1.3900	C19	C24	1.3900
C20'	C21'	1.3900	C20	C21	1.3900
C21'	C22'	1.3900	C21	C22	1.3900
C22'	C23'	1.3900	C22	C23	1.3900
C23'	C24'	1.3900	C23	C24	1.3900
C25'	C26'	1.172(5)	C25	C26	1.164(6)

Table S19 Bond Angles for KM48165.

Atom	Atom	Atom	Angle/°	Atom	Atom	Atom	Angle/°
C10'	N1'	C11'	118.9(3)	C10	N1	C11	119.1(3)
C1E	C1F	C1A	120.0	C2	C1	C6	120.4(4)
C1D	C1E	C1F	120.0	C3	C2	C1	121.3(4)
C1E	C1D	C1C	120.0	C4	C3	C2	118.9(4)
C1B	C1C	C1D	120.0	C3	C4	C5	120.4(4)
C1A	C1B	C1C	120.0	C4	C5	C6	121.2(3)
C1F	C1A	C8'	121.2(4)	C1	C6	C5	117.8(3)
C1B	C1A	C1F	120.0	C1	C6	C7	121.9(3)
C1B	C1A	C8'	118.5(4)	C5	C6	C7	120.2(3)
C6'	C1'	C2'	120.9(3)	O1	C7	C6	112.8(3)
C3'	C2'	C1'	120.5(4)	O1	C7	C8	107.6(3)
C2'	C3'	C4'	119.3(3)	O1	C7	C25	110.1(3)
C3'	C4'	C5'	120.2(3)	C6	C7	C8	108.4(3)
C6'	C5'	C4'	120.9(3)	C25	C7	C6	108.7(3)
C1'	C6'	C5'	118.3(3)	C25	C7	C8	109.1(3)
C1'	C6'	C7'	120.9(3)	C9	C8	C7	111.5(3)

C5'	C6'	C7'	120.8(3)	C9	C8	C19A	111.1(6)
O1'	C7'	C6'	110.9(2)	C19A	C8	C7	112.1(6)
O1'	C7'	C8'	108.1(2)	C19	C8	C7	112.8(9)
O1'	C7'	C25'	108.3(3)	C19	C8	C9	113.8(9)
C6'	C7'	C8'	110.5(2)	C10	C9	C8	111.1(3)
C25'	C7'	C6'	109.0(2)	N1	C10	C9	114.9(3)
C25'	C7'	C8'	110.0(2)	N1	C10	C14	121.9(3)
C1A	C8'	C7'	108.9(4)	C14	C10	C9	123.2(3)
C1A	C8'	C9'	114.0(4)	C12	C11	N1	123.5(4)
C9'	C8'	C7'	110.8(2)	C11	C12	C13	119.6(4)
C19'	C8'	C7'	118.7(9)	C12	C13	C18	122.5(4)
C19'	C8'	C9'	110.7(9)	C14	C13	C12	117.6(3)
C10'	C9'	C8'	113.2(3)	C14	C13	C18	119.8(4)
N1'	C10'	C9'	114.9(3)	C13	C14	C10	118.3(3)
N1'	C10'	C14'	121.7(3)	C13	C14	C15	118.1(3)
C14'	C10'	C9'	123.4(3)	C15	C14	C10	123.6(4)
C12'	C11'	N1'	123.8(3)	C16	C15	C14	120.4(5)
C11'	C12'	C13'	119.5(3)	C15	C16	C17	120.8(5)
C12'	C13'	C14'	118.2(3)	C18	C17	C16	120.7(5)
C12'	C13'	C18'	123.4(4)	C17	C18	C13	120.2(5)
C14'	C13'	C18'	118.4(4)	C20A	C19A	C8	121.2(11)
C13'	C14'	C10'	117.9(3)	C20A	C19A	C24A	120.0
C15'	C14'	C10'	123.2(3)	C24A	C19A	C8	118.8(11)
C15'	C14'	C13'	118.9(3)	C19A	C20A	C21A	120.0
C16'	C15'	C14'	119.8(4)	C22A	C21A	C20A	120.0
C15'	C16'	C17'	121.7(4)	C23A	C22A	C21A	120.0
C18'	C17'	C16'	119.7(4)	C24A	C23A	C22A	120.0
C17'	C18'	C13'	121.4(4)	C23A	C24A	C19A	120.0
C20'	C19'	C8'	121.9(13)	C20	C19	C8	122.3(19)
C20'	C19'	C24'	120.0	C20	C19	C24	120.0
C24'	C19'	C8'	118.1(13)	C24	C19	C8	117.5(19)
C19'	C20'	C21'	120.0	C21	C20	C19	120.0
C22'	C21'	C20'	120.0	C20	C21	C22	120.0
C23'	C22'	C21'	120.0	C21	C22	C23	120.0
C22'	C23'	C24'	120.0	C24	C23	C22	120.0
C23'	C24'	C19'	120.0	C23	C24	C19	120.0
C26'	C25'	C7'	176.9(4)	C26	C25	C7	177.4(5)

Table S20 Hydrogen Bonds for KM48165.

D	H	A	d(D-H)/Å	d(H-A)/Å	d(D-A)/Å	D-H-A/°
O1'	H1'	N1'[1]	0.82	2.02	2.830(4)	169.9
O1	H1	N1[2]	0.82	2.02	2.836(4)	171.3

[1]3/2-X,-1/2+Y,2-Z; [2]3/2-X,1/2+Y,1-Z

Table S21 Torsion Angles for KM48165.

A	B	C	D	Angle/°	A	B	C	D	Angle/°
O1'	C7'	C8'	C1A	-65.1(4)	O1	C7	C8	C9	58.3(4)
O1'	C7'	C8'	C9'	61.1(3)	O1	C7	C8	C19A	-67.0(8)
O1'	C7'	C8'	C19'	-68.6(10)	O1	C7	C8	C19	-71.3(13)
N1'	C10'	C14'	C13'	-0.3(5)	N1	C10	C14	C13	-0.1(5)
N1'	C10'	C14'	C15'	-179.6(3)	N1	C10	C14	C15	-178.5(3)

N1'	C11'	C12'	C13'	-0.5(7)	N1	C11	C12	C13	0.7(7)
C1F	C1E	C1D	C1C	0.0	C1	C2	C3	C4	1.5(7)
C1F	C1A	C8'	C7'	67.8(4)	C1	C6	C7	O1	7.9(4)
C1F	C1A	C8'	C9'	-56.6(4)	C1	C6	C7	C8	-111.2(3)
C1E	C1F	C1A	C1B	0.0	C1	C6	C7	C25	130.3(3)
C1E	C1F	C1A	C8'	-173.2(6)	C2	C1	C6	C5	-0.7(5)
C1E	C1D	C1C	C1B	0.0	C2	C1	C6	C7	176.0(3)
C1D	C1C	C1B	C1A	0.0	C2	C3	C4	C5	-0.8(7)
C1C	C1B	C1A	C1F	0.0	C3	C4	C5	C6	-0.6(6)
C1C	C1B	C1A	C8'	173.4(6)	C4	C5	C6	C1	1.3(6)
C1B	C1A	C8'	C7'	-105.5(4)	C4	C5	C6	C7	-175.3(3)
C1B	C1A	C8'	C9'	130.1(4)	C5	C6	C7	O1	-175.6(3)
C1A	C1F	C1E	C1D	0.0	C5	C6	C7	C8	65.3(4)
C1A	C8'	C9'	C10'	-65.9(4)	C5	C6	C7	C25	-53.2(4)
C1'	C2'	C3'	C4'	0.7(7)	C6	C1	C2	C3	-0.7(6)
C1'	C6'	C7'	O1'	-155.7(3)	C6	C7	C8	C9	-179.5(3)
C1'	C6'	C7'	C8'	84.4(4)	C6	C7	C8	C19A	55.3(8)
C1'	C6'	C7'	C25'	-36.6(4)	C6	C7	C8	C19	51.0(13)
C2'	C1'	C6'	C5'	-2.1(6)	C7	C8	C9	C10	176.1(3)
C2'	C1'	C6'	C7'	178.1(4)	C7	C8	C19A	C20A	71.7(9)
C2'	C3'	C4'	C5'	-1.1(7)	C7	C8	C19A	C24A	-107.6(9)
C3'	C4'	C5'	C6'	-0.1(6)	C7	C8	C19	C20	58.8(14)
C4'	C5'	C6'	C1'	1.7(6)	C7	C8	C19	C24	-127.0(11)
C4'	C5'	C6'	C7'	-178.5(3)	C8	C9	C10	N1	-65.4(4)
C5'	C6'	C7'	O1'	24.5(4)	C8	C9	C10	C14	114.1(3)
C5'	C6'	C7'	C8'	-95.4(3)	C8	C19A	C20A	C21A	-179.3(10)
C5'	C6'	C7'	C25'	143.6(3)	C8	C19A	C24A	C23A	179.3(10)
C6'	C1'	C2'	C3'	0.9(7)	C8	C19	C20	C21	174.0(17)
C6'	C7'	C8'	C1A	56.4(4)	C8	C19	C24	C23	-174.3(17)
C6'	C7'	C8'	C9'	-177.3(3)	C9	C8	C19A	C20A	-53.8(10)
C6'	C7'	C8'	C19'	52.9(10)	C9	C8	C19A	C24A	126.9(9)
C7'	C8'	C9'	C10'	170.7(3)	C9	C8	C19	C20	-69.5(13)
C7'	C8'	C19'	C20'	66.0(13)	C9	C8	C19	C24	104.6(12)
C7'	C8'	C19'	C24'	-115.2(10)	C9	C10	C14	C13	-179.5(3)
C8'	C9'	C10'	N1'	-61.3(4)	C9	C10	C14	C15	2.1(5)
C8'	C9'	C10'	C14'	121.2(3)	C10	N1	C11	C12	0.4(6)
C8'	C19'	C20'	C21'	178.8(18)	C10	C14	C15	C16	178.9(4)
C8'	C19'	C24'	C23'	-178.8(17)	C11	N1	C10	C9	178.8(3)
C9'	C8'	C19'	C20'	-63.8(11)	C11	N1	C10	C14	-0.7(5)
C9'	C8'	C19'	C24'	115.0(11)	C11	C12	C13	C14	-1.5(6)
C9'	C10'	C14'	C13'	177.1(3)	C11	C12	C13	C18	178.1(4)
C9'	C10'	C14'	C15'	-2.2(5)	C12	C13	C14	C10	1.2(5)
C10'	N1'	C11'	C12'	1.2(7)	C12	C13	C14	C15	179.7(3)
C10'	C14'	C15'	C16'	179.4(4)	C12	C13	C18	C17	180.0(4)
C11'	N1'	C10'	C9'	-178.3(3)	C13	C14	C15	C16	0.5(6)
C11'	N1'	C10'	C14'	-0.7(5)	C14	C13	C18	C17	-0.5(6)
C11'	C12'	C13'	C14'	-0.5(6)	C14	C15	C16	C17	-0.7(7)
C11'	C12'	C13'	C18'	-179.0(4)	C15	C16	C17	C18	0.4(8)
C12'	C13'	C14'	C10'	0.9(5)	C16	C17	C18	C13	0.3(8)
C12'	C13'	C14'	C15'	-179.7(4)	C18	C13	C14	C10	-178.4(3)
C12'	C13'	C18'	C17'	-179.9(5)	C18	C13	C14	C15	0.1(5)
C13'	C14'	C15'	C16'	0.0(6)	C19A	C8	C9	C10	-58.2(8)
C14'	C13'	C18'	C17'	1.6(6)	C19A	C20A	C21A	C22A	0.0

C14'	C15'	C16'	C17'	0.7(7)	C20A	C19A	C24A	C23A	0.0
C15'	C16'	C17'	C18'	-0.3(9)	C20A	C21A	C22A	C23A	0.0
C16'	C17'	C18'	C13'	-0.9(8)	C21A	C22A	C23A	C24A	0.0
C18'	C13'	C14'	C10'	179.5(3)	C22A	C23A	C24A	C19A	0.0
C18'	C13'	C14'	C15'	-1.1(5)	C24A	C19A	C20A	C21A	0.0
C19'	C8'	C9'	C10'	-55.4(9)	C19	C8	C9	C10	-54.9(13)
C19'	C20'	C21'	C22'	0.0	C19	C20	C21	C22	0.0
C20'	C19'	C24'	C23'	0.0	C20	C19	C24	C23	0.0
C20'	C21'	C22'	C23'	0.0	C20	C21	C22	C23	0.0
C21'	C22'	C23'	C24'	0.0	C21	C22	C23	C24	0.0
C22'	C23'	C24'	C19'	0.0	C22	C23	C24	C19	0.0
C24'	C19'	C20'	C21'	0.0	C24	C19	C20	C21	0.0
C25'	C7'	C8'	C1A	176.8(4)	C25	C7	C8	C9	-61.2(4)
C25'	C7'	C8'	C9'	-56.9(3)	C25	C7	C8	C19A	173.6(8)
C25'	C7'	C8'	C19'	173.3(10)	C25	C7	C8	C19	169.2(13)

Table S22 Hydrogen Atom Coordinates (Å×10^4) and Isotropic Displacement Parameters (Å^2×10^3) for KM48165.

**Atom**	***x***	***y***	***z***	**U(eq)**
H1'	7915	-1093	9889	90
H1F	6696	-801	8581	91
H1E	6436	-1266	7273	127
H1D	6492	335	6413	173
H1C	6807	2401	6861	158
H1B	7066	2866	8169	109
H1'A	8444	2671	9922	77
H2'	9106	3198	9334	99
H3'	9210	1907	8369	97
H4'	8636	96	7980	92
H5'	7980	-446	8579	76
H8'	7238	2300	9482	59
H8'A	7225	2312	9435	59
H9'A	6611	149	9721	67
H9'B	6923	1161	10362	67
H11'	5898	4849	9590	91
H12'	4976	4181	9107	89
H15'	5804	-555	9231	87
H16'	4875	-1264	8748	118
H17'	4106	174	8448	122
H18'	4271	2330	8640	101
H20'	6670	-621	8461	91
H21'	6196	-669	7135	127
H22'	6094	1222	6417	173
H23'	6466	3161	7023	158
H24'	6939	3208	8349	109
H26'	8351	1352	11768	98
H1	7822	8276	5783	109
H1A	7608	7818	6882	80
H2	7741	7080	8109	100
H3	7925	4930	8419	96
H4	8016	3517	7499	96
H5	7903	4240	6275	85
H8A	7131	4940	5107	66

H8B	7116	4934	5116	66
H9A	6826	7183	4172	78
H9B	7226	6067	4061	78
H11	6042	2646	3244	97
H12	5172	3483	2573	96
H15	6051	8044	3602	96
H16	5174	8934	2903	124
H17	4428	7643	2164	127
H18	4540	5459	2123	107
H20A	6410	8025	5049	90
H21A	5647	8387	5497	130
H22A	5313	6718	6082	164
H23A	5742	4686	6218	142
H24A	6505	4324	5770	103
H20	6540	7996	5427	90
H21	5721	8161	5780	130
H22	5246	6300	5947	164
H23	5589	4274	5761	142
H24	6407	4108	5408	103
H26	8812	5595	5035	133

Table S23 Atomic Occupancy for KM48165.

Atom	*Occupancy*	Atom	*Occupancy*	Atom	*Occupancy*
C1F	0.712(11)	H1F	0.712(11)	C1E	0.712(11)
H1E	0.712(11)	C1D	0.712(11)	H1D	0.712(11)
C1C	0.712(11)	H1C	0.712(11)	C1B	0.712(11)
H1B	0.712(11)	C1A	0.712(11)	H8'	0.288(11)
H8'A	0.712(11)	C19'	0.288(11)	C20'	0.288(11)
H20'	0.288(11)	C21'	0.288(11)	H21'	0.288(11)
C22'	0.288(11)	H22'	0.288(11)	C23'	0.288(11)
H23'	0.288(11)	C24'	0.288(11)	H24'	0.288(11)
H8A	0.40(3)	H8B	0.60(3)	C19A	0.60(3)
C20A	0.60(3)	H20A	0.60(3)	C21A	0.60(3)
H21A	0.60(3)	C22A	0.60(3)	H22A	0.60(3)
C23A	0.60(3)	H23A	0.60(3)	C24A	0.60(3)
H24A	0.60(3)	C19	0.40(3)	C20	0.40(3)
H20	0.40(3)	C21	0.40(3)	H21	0.40(3)
C22	0.40(3)	H22	0.40(3)	C23	0.40(3)
H23	0.40(3)	C24	0.40(3)	H24	0.40(3)

Table 24 Solvent masks information for KM48165.

Number	X	Y	Z	Volume	Electron count	Content
1	0.000	0.148	0.000	29.3	4.8	?
2	0.000	0.634	0.500	150.1	44.3	?
3	0.500	0.134	0.500	150.1	44.3	?
4	0.500	0.648	0.000	29.3	4.8	?

7. Characterization of adducts

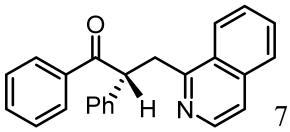

(*R*)-3-(isoquinolin-1-yl)-1,2-diphenylpropan-1-one (3a): light yellow solid; Mp: 99.3 °C; 26.3 mg, 77% yield; 92% ee; $[\alpha]_D^{25}$ = 78.8 (*c* 1.0, $CHCl_3$); ^1H NMR (300 MHz, chloroform-d) δ 8.27 (d, *J* = 5.7 Hz, 1H), 8.19 (d, *J* = 8.4 Hz, 1H), 8.07 (d, *J* = 7.6 Hz, 2H), 7.76 (d, *J* = 8.1 Hz, 1H), 7.64 (t, *J* = 7.4 Hz, 1H), 7.56 (t, *J* = 7.7 Hz, 1H), 7.52 – 7.36 (m, 6H), 7.30 (t, *J* = 7.4 Hz, 2H), 7.21 (t, *J* = 7.3 Hz, 1H), 5.72 (dd, *J* = 9.9, 4.4 Hz, 1H), 4.44 (dd, *J* = 16.3, 9.9 Hz, 1H), 3.68 (dd, *J* = 16.3, 4.5 Hz, 1H); ^{13}C NMR (75 MHz, chloroform-d) δ 199.8, 158.7, 141.2, 139.5, 137.3, 135.8, 132.4, 129.8, 128.9, 128.8, 128.4, 128.3, 127.1, 127.1, 127.0, 125.0, 119.3, 51.4, 38.8. HRMS (ESI) m/z 338.1535 ($M+H^+$), calc. for $C_{24}H_{22}NO$ 338.1540.

The ee was determined by HPLC analysis: CHIRALPAK IG (4.6 mm i.d. x 250 mm); hexane/2–propanol = 85/15; flow rate 1.0 mL/min; 25 °C; 254 nm; retention time: 16.4 min (major) and 19.4 min (minor).

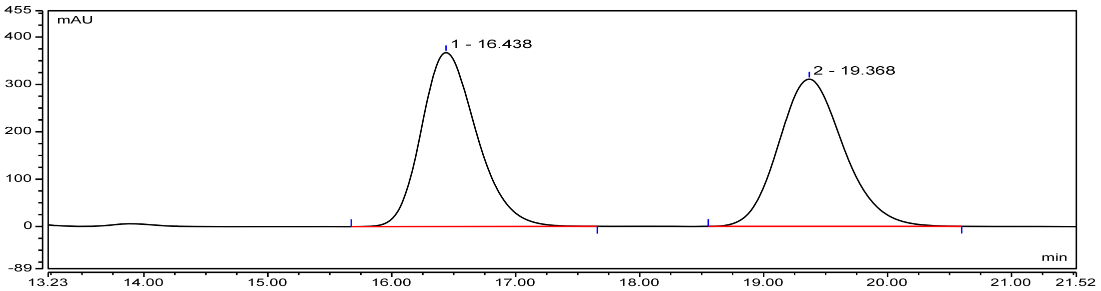

Entry	Retention Time	%Area	Area	Height
1	16.438	50.01	190.2	367.2
2	19.368	49.99	190.1	310.8

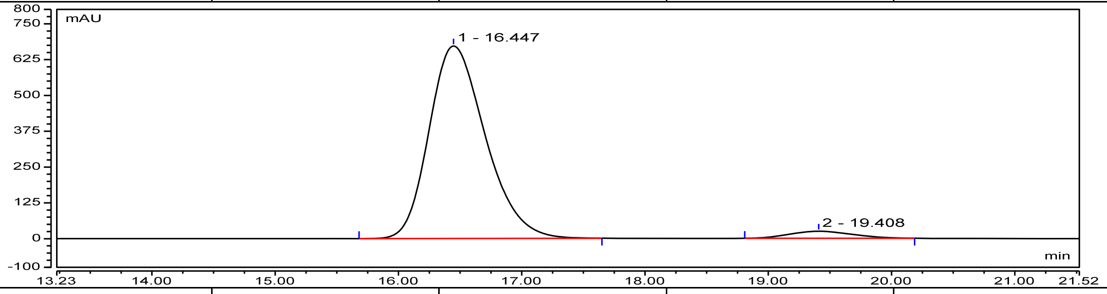

Entry	Retention Time	%Area	Area	Height
1	16.447	96.05	355.6	671.8
2	19.408	3.95	14.6	24.7

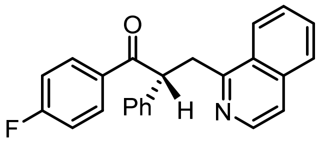

(*R*)-1-(4-fluorophenyl)-3-(isoquinolin-1-yl)-2-phenylpropan-1-one (3b): white solid; Mp: 162.3 °C; 24.5 mg, 70% yield; 92% ee; $[\alpha]_D^{25}$ = 9.5 (*c* 1.0, $CHCl_3$); ^1H NMR (300 MHz, chloroform-d) δ 8.25 (d, *J* = 5.7 Hz, 1H), 8.17 (d, *J* = 8.3 Hz, 1H), 8.12 – 8.05 (m, 2H), 7.77 (d, *J* = 8.1 Hz, 1H), 7.64 (t, *J* = 7.1 Hz, 1H), 7.59 – 7.52 (m, 1H), 7.47 – 7.39 (m, 3H), 7.33 – 7.28 (m, 2H), 7.25 – 7.19 (m, 1H), 7.07 (t, *J* = 8.6 Hz, 2H), 5.65 (dd, *J* = 10.0, 4.3 Hz, 1H), 4.43 (dd, *J* = 16.5, 10.1 Hz, 1H), 3.66 (dd, *J* = 16.5, 4.3 Hz, 1H); ^{13}C NMR (75 MHz, chloroform-d) δ

198.3, 165.3 (d, J = 253.7 Hz), 158.5, 141.3, 139.4, 135.8, 133.7 (d, J = 2.9 Hz), 131.4 (d, J = 9.1 Hz), 129.8, 129.0, 128.3, 127.2, 127.1, 127.1, 127.0, 124.9, 119.3, 115.39 (d, J = 21.8 Hz), 51.2, 38.9; ^{19}F NMR (376 MHz, chloroform-d) δ -105. HRMS (ESI) m/z 356.1443 (M+H$^+$), calc. for $C_{24}H_{19}FNO$ 356.1446.

The ee was determined by HPLC analysis: CHIRALPAK IA (4.6 mm i.d. x 250 mm); hexane/2–propanol = 90/10; flow rate 1.0 mL/min; 25 °C; 254 nm; retention time: 8.9 min (major) and 12.8 min (minor).

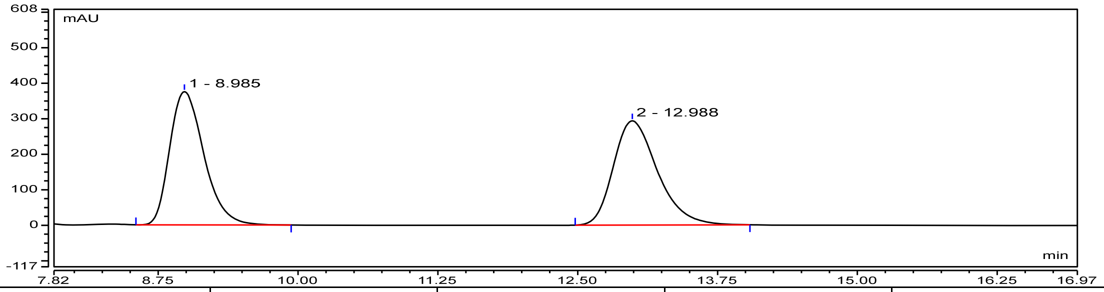

Entry	Retention Time	%Area	Area	Height
1	8.985	49.87	133.4161	375.06
2	12.988	50.13	134.1248	293.22

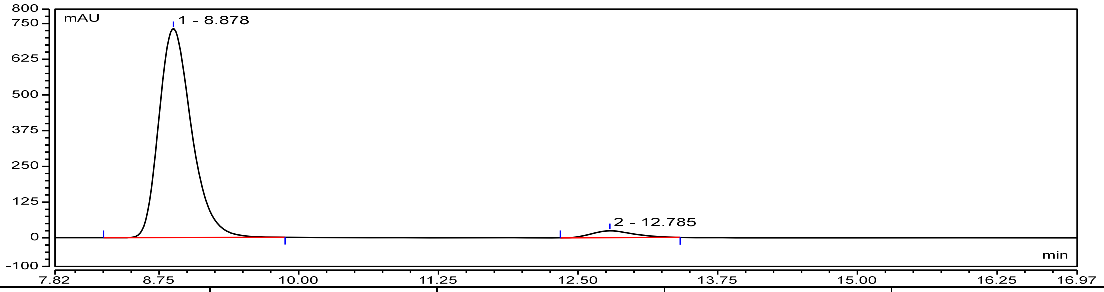

Entry	Retention Time	%Area	Area	Height
1	8.878	96.05	248.0311	730.74
2	12.785	3.95	10.1936	23.84

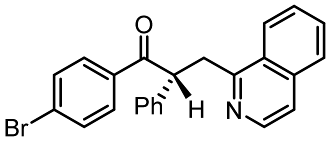

(*R*)-1-(4-bromophenyl)-3-(isoquinolin-1-yl)-2-phenylpropan-1-one (3c): white solid; Mp: 150.4 °C; 33.7 mg, 81% yield; 94% ee; $[\alpha]_D^{25}$ = –6.5 (c 1.0, $CHCl_3$); ^{1}H NMR (600 MHz, chloroform-d) δ 8.25 (d, J = 5.7 Hz, 1H), 8.17 (dd, J = 8.6, 1.2 Hz, 1H), 7.95 – 7.90 (m, 2H), 7.77 (d, J = 8.1 Hz, 1H), 7.64 – 7.63 (m, 1H), 7.58 – 7.51 (m, 3H), 7.45 (d, J = 5.7 Hz, 1H), 7.42 – 7.39 (m, 2H), 7.31 (t, J = 7.7 Hz, 2H), 7.25 – 7.20 (m, 1H), 5.65 (dd, J = 10.1, 4.2 Hz, 1H), 4.43 (dd, J = 16.4, 10.1 Hz, 1H), 3.67 (dd, J = 16.4, 4.2 Hz, 1H); ^{13}C NMR (151 MHz, chloroform-d) δ 198.9, 158.4, 141.0, 139.1, 136.1, 135.9, 131.6, 130.4, 129.9, 129.1, 128.3, 127.5, 127.3, 127.2, 127.1, 127.1, 119.4, 51.2, 38.8. HRMS (ESI) m/z 416.0641 (M+H$^+$), calc. for $C_{24}H_{19}BrNO$ 416.0645.

The ee was determined by HPLC analysis: Lux® 5µm Amylose-1 (4.6 mm i.d. x 250 mm); hexane/2–propanol = 85/15; flow rate 1.0 mL/min; 25 °C; 254 nm; retention time: 16.1 min (major) and 27.1 min (minor).

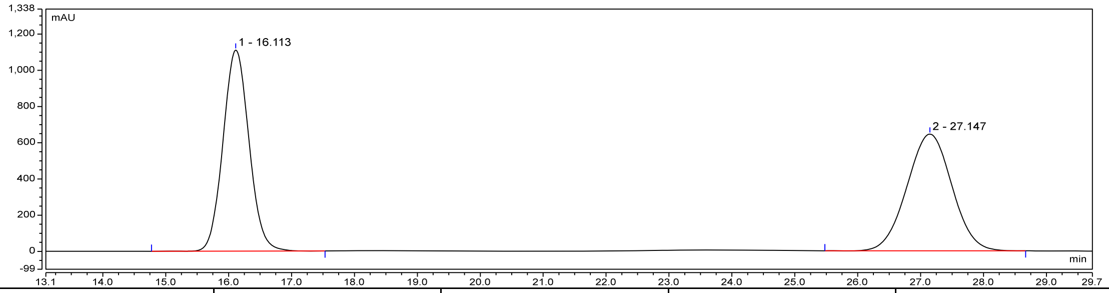

Entry	Retention Time	%Area	Area	Height
1	16.113	49.93	531.9758	1110.41
2	27.147	50.07	533.4382	644.35

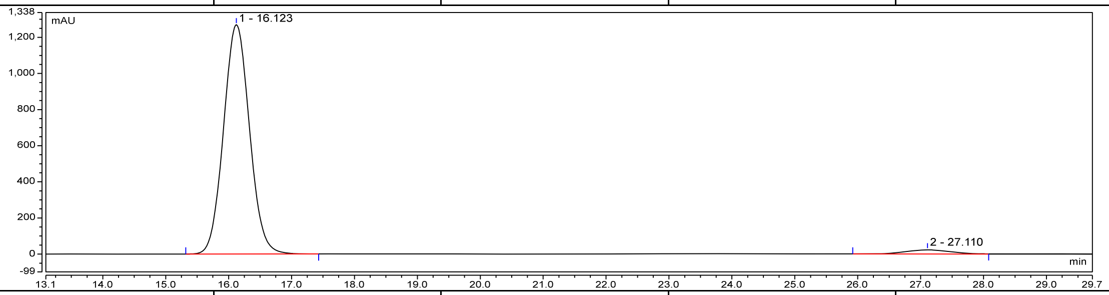

Entry	Retention Time	%Area	Area	Height
1	16.123	96.99	606.7912	1269.41
2	27.110	3.01	18.8074	22.83

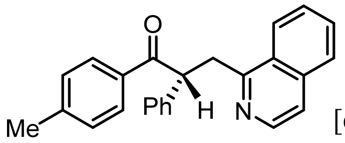

(*R*)-3-(isoquinolin-1-yl)-2-phenyl-1-(p-tolyl)propan-1-one (3d): white solid; Mp: 99.3 °C; 31.6 mg, 90% yield; 94% ee; $[\alpha]_D^{25}$ = 39.0 (*c* 1.0, $CHCl_3$); ^{1}H NMR (300 MHz, chloroform-d) δ 8.28 (d, *J* = 5.7 Hz, 1H), 8.18 (d, *J* = 8.4 Hz, 1H), 7.97 (d, *J* = 7.9 Hz, 2H), 7.76 (d, *J* = 8.1 Hz, 1H), 7.63 (t, *J* = 7.5 Hz, 1H), 7.54 (t, *J* = 7.7 Hz, 1H), 7.44 (d, *J* = 5.5 Hz, 2H), 7.33 – 7.24 (m, 3H), 7.20 (dd, *J* = 7.9, 3.3 Hz, 3H), 5.68 (dd, *J* = 9.8, 4.6 Hz, 1H), 4.42 (dd, *J* = 16.2, 9.7 Hz, 1H), 3.66 (dd, *J* = 16.3, 4.7 Hz, 1H), 2.36 (s, 3H); ^{13}C NMR (75 MHz, chloroform-d) δ 199.3, 158.8, 143.1, 141.4, 139.8, 135.8, 134.6, 129.7, 129.0, 129.0, 128.4, 127.2, 127.1, 127.0, 126.9, 125.0, 119.2, 51.3, 38.7, 21.6. HRMS (ESI) m/z 352.1694 ($M+H^+$), calc. for $C_{25}H_{22}NO$ 352.1696.

The ee was determined by HPLC analysis: CHIRALPAK IG (4.6 mm i.d. x 250 mm); hexane/2–propanol = 85/15; flow rate 1.0 mL/min; 25 °C; 254 nm; retention time: 23.9 min (major) and 22.2 min (minor).

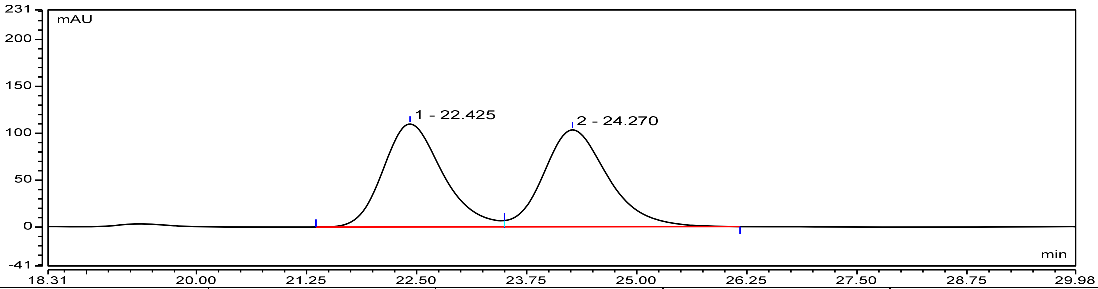

Entry	Retention Time	%Area	Area	Height
1	22.425	49.73	86.2974	109.72
2	24.270	50.27	87.2216	103.23

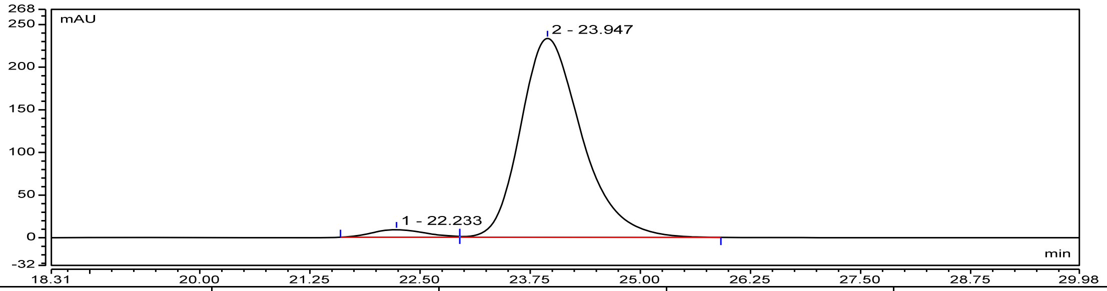

Entry	Retention Time	%Area	Area	Height
1	22.233	3.20	6.2095	8.95
2	23.947	96.80	187.8255	233.14

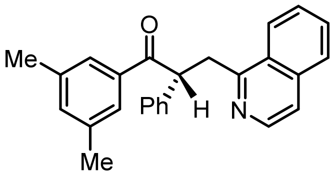

(*R*)-1-(3,5-dimethylphenyl)-3-(isoquinolin-1-yl)-2-phenylpropan-1-one (3e): white solid; Mp: 102.1 °C; 26.0 mg, 71% yield; 90% ee; $[\alpha]_D^{25}$ = 122.8 (*c* 1.0, $CHCl_3$); ^{1}H NMR (300 MHz, chloroform-d) δ 8.65 (s, 1H), 8.27 – 8.20 (m, 2H), 8.11 (d, *J* = 8.5 Hz, 1H), 7.94 (d, *J* = 7.8 Hz, 1H), 7.85 – 7.81 (m, 2H), 7.77 (d, *J* = 8.1 Hz, 1H), 7.64 (t, *J* = 7.5 Hz, 1H), 7.60 – 7.46 (m, 5H), 7.44 (d, *J* = 5.8 Hz, 1H), 7.31 (t, *J* = 7.5 Hz, 2H), 7.21 (t, *J* = 7.4 Hz, 1H), 5.87 (dd, *J* = 10.0, 4.3 Hz, 1H), 4.51 (dd, *J* = 16.4, 10.0 Hz, 1H), 3.73 (dd, *J* = 16.4, 4.4 Hz, 1H); ^{13}C NMR (75 MHz, chloroform-d) δ 199.7, 158.7, 141.4, 139.6, 135.8, 135.3, 134.6, 132.5, 130.4, 129.7, 129.6, 129.0, 128.4, 128.1, 127.6, 127.2, 127.1, 127.1, 126.4, 125.0, 124.8, 119.2, 51.3, 38.9. HRMS (ESI) m/z 366.1851 (M+H$^+$), calc. for $C_{26}H_{24}NO$ 366.1853.

The ee was determined by HPLC analysis: CHIRALPAK IA (4.6 mm i.d. x 250 mm); hexane/2–propanol = 98/2; flow rate 1.0 mL/min; 25 °C; 254 nm; retention time: 18.3 min (major) and 35.2 min (minor).

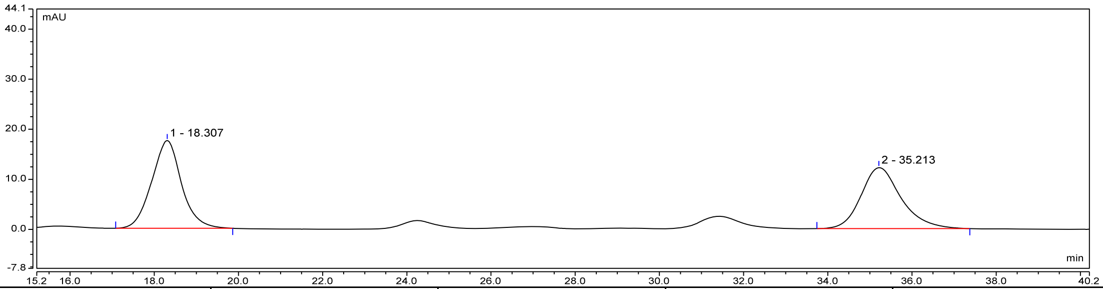

Entry	Retention Time	%Area	Area	Height
1	18.307	51.65	14.3957	17.52
2	35.213	48.35	13.4774	12.20

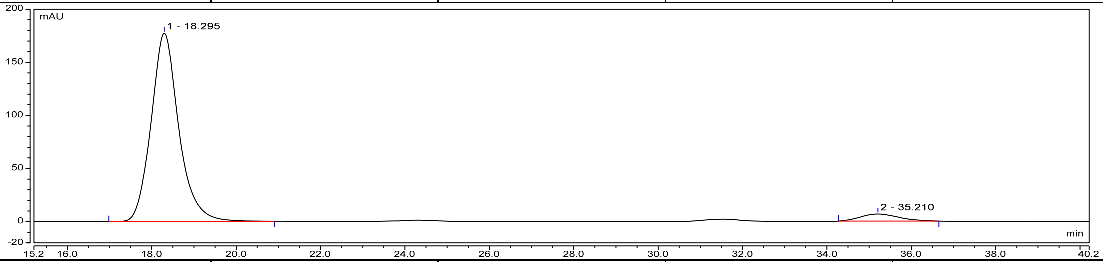

Entry	Retention Time	%Area	Area	Height
1	18.295	95.08	135.4351	177.38
2	35.210	4.92	7.0011	6.75

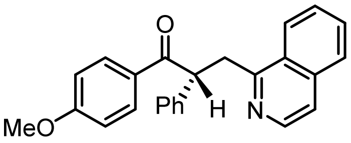

(*R*)-3-(isoquinolin-1-yl)-1-(4-methoxyphenyl)-2-phenylpropan-1-one (3f): white solid; Mp: 99.8 ºC; 27.2 mg, 74% yield; 93% ee; $[\alpha]_D^{25}$ = 101 (*c* 1.0, $CHCl_3$); ^{1}H NMR (300 MHz, chloroform-d) δ 8.29 (d, *J* = 5.7 Hz, 1H), 8.19 (d, *J* = 8.4 Hz, 1H), 8.05 (d, *J* = 8.8 Hz, 2H), 7.76 (d, *J* = 8.1 Hz, 1H), 7.63 (t, *J* = 7.4 Hz, 1H), 7.55 (t, *J* = 7.6 Hz, 1H), 7.46 – 7.40 (m, 4H), 7.33 – 7.26 (m, 3H), 7.20 (t, *J* = 7.2 Hz, 1H), 6.88 (d, *J* = 8.8 Hz, 2H), 5.66 (dd, *J* = 9.6, 4.7 Hz, 1H), 4.41 (dd, *J* = 16.2, 9.6 Hz, 1H), 3.82 (s, 3H), 3.65 (dd, *J* = 16.1, 4.7 Hz, 1H); ^{13}C NMR (75 MHz, chloroform-d) δ 198.2, 163.0, 159.0, 141.4, 140.0, 135.8, 131.1, 130.1, 129.7, 128.9, 128.3, 127.2, 127.1, 127.0, 126.9, 125.0, 119.2, 113.5, 55.3, 51.1, 38.7. HRMS (ESI) m/z 368.1641(M+H$^+$), calc. for $C_{25}H_{22}NO_2$ 368.1646.

The ee was determined by HPLC analysis: CHIRALPAK IG (4.6 mm i.d. x 250 mm); hexane/2–propanol = 60/40; flow rate 1.0 mL/min; 25 ºC; 254 nm; retention time: 20.2 min (major) and 17.8 min (minor).

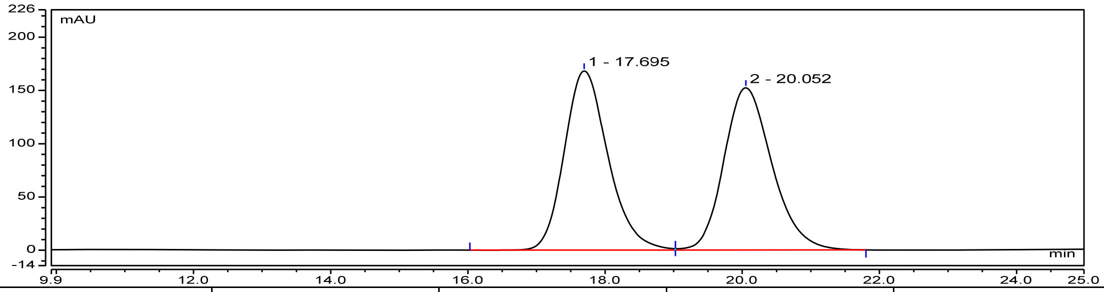

Entry	Retention Time	%Area	Area	Height
1	17.695	50.18	124.6461	168.15
2	20.052	49.82	123.7544	152.29

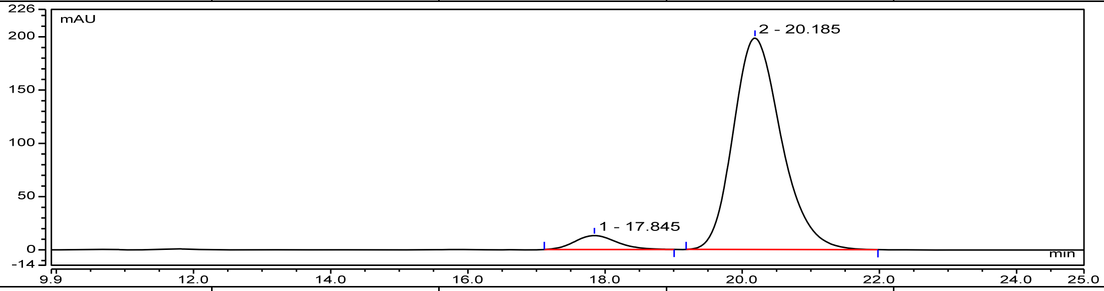

Entry	Retention Time	%Area	Area	Height
1	17.845	3.62	8.5207	12.67
2	20.185	96.38	161.2277	198.44

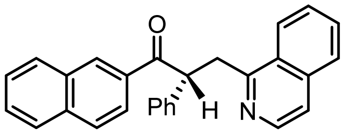

(*R*)-3-(isoquinolin-1-yl)-1-(naphthalen-2-yl)-2-phenylpropan-1-one (3g): white solid; Mp: 159.7 ºC; 29.0 mg, 75% yield; 90% ee; $[\alpha]_D^{25}$ = 29.1 (*c* 1.0, $CHCl_3$); ^{1}H NMR (300 MHz, chloroform-d) δ 8.65 (s, 1H), 8.27 – 8.20 (m, 2H), 8.11 (d, *J* = 8.5 Hz, 1H), 7.94 (d, *J* = 7.8 Hz, 1H), 7.85 – 7.81 (m, 2H), 7.77 (d, *J* = 8.1 Hz, 1H), 7.64 (t, *J* = 7.5 Hz, 1H), 7.60 – 7.46 (m, 5H), 7.44 (d, *J* = 5.8 Hz, 1H), 7.31 (t, *J* = 7.5 Hz, 2H), 7.21 (t, *J* = 7.4 Hz, 1H), 5.87 (dd, *J* = 10.0, 4.3 Hz, 1H), 4.51 (dd, *J* = 16.4, 10.0 Hz, 1H), 3.73 (dd, *J* = 16.4, 4.4 Hz, 1H); ^{13}C NMR (75 MHz, chloroform-d) δ 199.7, 158.7, 141.4, 139.6, 135.8, 135.3, 134.6, 132.5, 130.4,

129.7, 129.6, 129.0, 128.4, 128.1, 127.6, 127.2, 127.1, 127.1, 126.4, 125.0, 124.8, 119.2, 51.3, 38.9. HRMS (ESI) m/z 388.1695 (M+H$^+$), calc. for $C_{28}H_{22}NO$ 388.1696.

The ee was determined by HPLC analysis: CHIRALPAK IG (4.6 mm i.d. x 250 mm); hexane/2–propanol = 80/20; flow rate 1.0 mL/min; 25 °C; 254 nm; retention time: 25.7 min (major) and 33.1 min (minor).

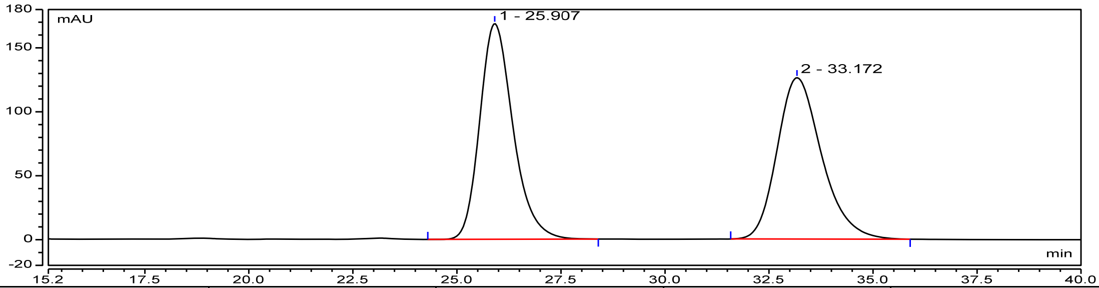

Entry	Retention Time	%Area	Area	Height
1	25.907	50.00	156.7556	168.55
2	33.172	50.00	156.7732	126.09

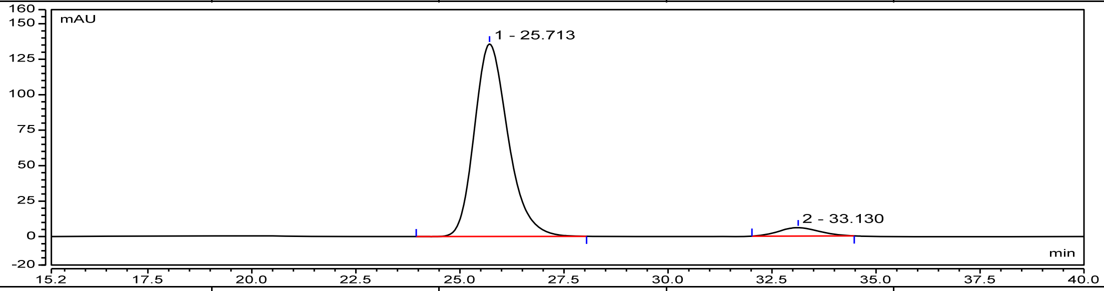

Entry	Retention Time	%Area	Area	Height
1	25.713	95.02	127.4495	135.72
2	33.130	4.98	6.6728	5.90

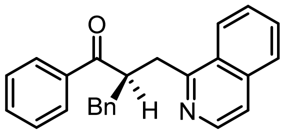

(*R*)-2-benzyl-3-(isoquinolin-1-yl)-1-phenylpropan-1-one (3h): white solid; Mp: 93.6 °C; 22.8 mg, 65% yield; 91% ee; $[\alpha]_D^{25}$ = –54 (*c* 1.0, $CHCl_3$); ^{1}H NMR (400 MHz, chloroform-d) δ 8.24 (d, *J* = 5.7 Hz, 1H), 8.15 (d, *J* = 8.4 Hz, 1H), 7.95 (d, *J* = 7.7 Hz, 2H), 7.74 (d, *J* = 8.1 Hz, 1H), 7.64 (t, *J* = 7.5 Hz, 1H), 7.58 (t, *J* = 7.6 Hz, 1H), 7.50 – 7.46 (m, 1H), 7.44 – 7.34 (m, 3H), 7.25 – 7.20 (m, 4H), 7.18 – 7.12 (m, 1H), 4.78 (s, 1H), 3.92 (dd, *J* = 15.8, 9.0 Hz, 1H), 3.49 (dd, *J* = 15.9, 5.0 Hz, 1H), 3.24 (dd, *J* = 13.6, 6.7 Hz, 1H), 2.90 (dd, *J* = 13.6, 7.7 Hz, 1H); ^{13}C NMR (101 MHz, chloroform-d) δ 203.7, 158.7, 139.3, 137.5, 136.0, 132.4, 130.0, 129.1, 128.4, 128.4, 128.3, 127.2, 126.3, 125.1, 119.4, 46.6, 38.6, 35.7. HRMS (ESI) m/z 352.1693 (M+H$^+$), calc. for $C_{25}H_{22}NO$ 352.1696.

The ee was determined by HPLC analysis: Lux® 5μm Amylose-1 (4.6 mm i.d. x 250 mm); hexane/2–propanol = 85/15; flow rate 1.0 mL/min; 25 °C; 254 nm; retention time: 13.7 min (major) and 16.2 min (minor).

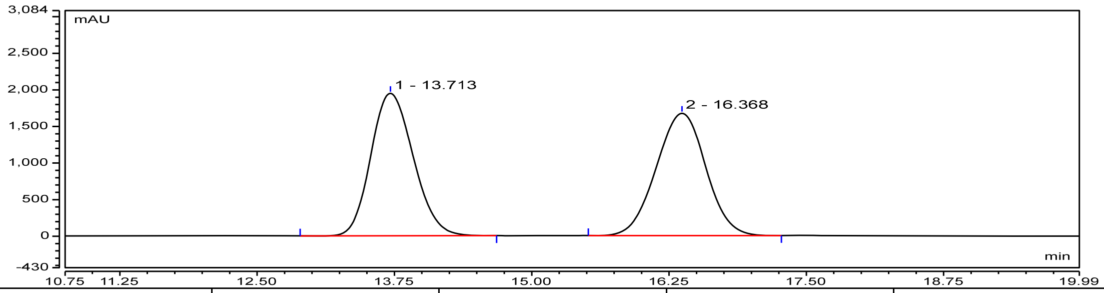

Entry	Retention Time	%Area	Area	Height
1	13.713	49.91	874.2331	1946.34
2	16.368	50.09	877.2523	1670.41

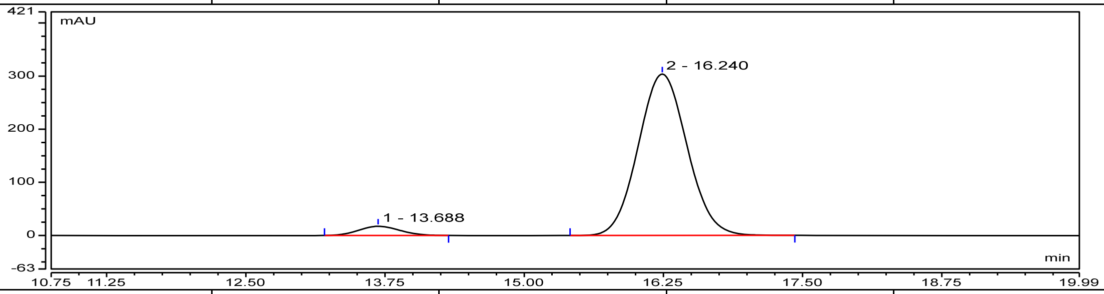

Entry	Retention Time	%Area	Area	Height
1	13.688	4.59	7.4500	17.31
2	16.240	95.41	154.9544	303.77

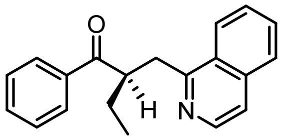

(*R*)-2-(isoquinolin-1-ylmethyl)-1-phenylbutan-1-one (3i): yellow solid; Mp: 75.7 °C; 15.9 mg, 55% yield; 90% ee; $[\alpha]_D^{25}$ = 39.4 (*c* 1.0, $CHCl_3$); ^{1}H NMR (300 MHz, chloroform-d) δ 8.26 (dd, *J* = 11.3, 6.9 Hz, 2H), 8.02 (d, *J* = 7.5 Hz, 2H), 7.75 (d, *J* = 7.9 Hz, 1H), 7.66 – 7.56 (m, 2H), 7.50 (d, *J* = 7.2 Hz, 1H), 7.46 – 7.37 (m, 3H), 4.37 (dt, *J* = 12.4, 6.0 Hz, 1H), 3.91 (dd, *J* = 15.7, 8.7 Hz, 1H), 3.47 (dd, *J* = 15.7, 5.3 Hz, 1H), 1.93 (dp, *J* = 14.5, 7.4 Hz, 1H), 1.71 (dp, *J* = 14.0, 7.2 Hz, 1H), 0.97 (t, *J* = 7.5 Hz, 3H); ^{13}C NMR (75 MHz, chloroform-d) δ 204.3, 159.3, 141.4, 137.8, 135.9, 132.4, 129.7, 128.4, 128.3, 127.2, 127.2, 127.0, 125.1, 46.1, 35.7, 25.8, 11.9. HRMS (ESI) m/z 290.1537 (M+H$^+$), calc. for $C_{20}H_{20}NO$ 290.154.

The ee was determined by HPLC analysis: CHIRALPAK IG (4.6 mm i.d. x 250 mm) and Lux® 5µm Iamylose-1; hexane/2–propanol = 95/05; flow rate 1.0 mL/min; 25 °C; 254 nm; retention time: 32.1 min (major) and 39.6 min (minor).

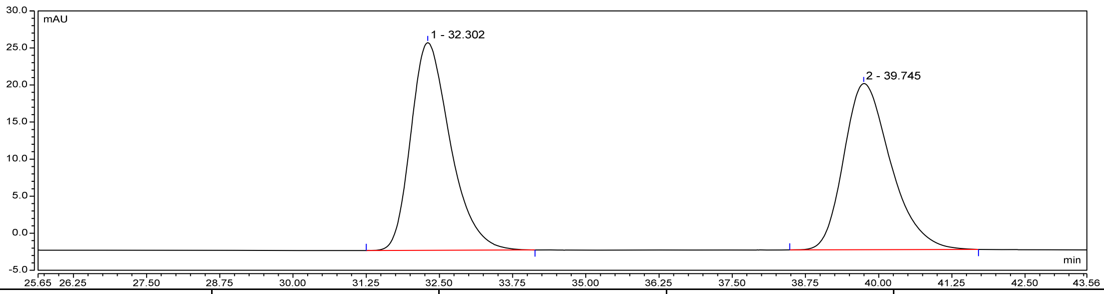

Entry	Retention Time	%Area	Area	Height
1	32.302	50.70	21.4747	27.87
2	39.745	49.30	20.8844	22.41

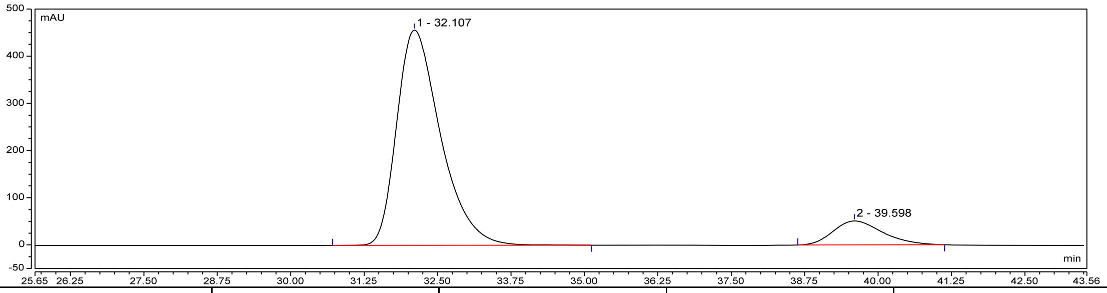

Entry	Retention Time	%Area	Area	Height
1	32.107	89.96	393.9165	455.96
2	39.598	10.04	43.9809	48.61

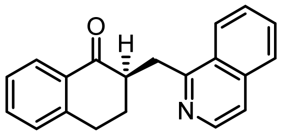

(*R*)-2-(isoquinolin-1-ylmethyl)-3,4-dihydronaphthalen-1(2*H*)-one (3j): light yellow solid; Mp: 82.8 °C; 19.2 mg, 67% yield; 92% ee; $[\alpha]_D^{25}$ = 5.2 (*c* 1.0, $CHCl_3$); ^{1}H NMR (300 MHz, chloroform-d) δ 8.50 – 8.48 (m, 1H), 8.30 (d, *J* = 8.4 Hz, 1H), 8.15 (d, *J* = 7.9 Hz, 1H), 7.87 (d, *J* = 8.2 Hz, 1H), 7.72 – 7.64 (m, 2H), 7.54 (dd, *J* = 24.0, 6.5 Hz, 2H), 7.38 (d, *J* = 7.8 Hz, 1H), 7.27 (d, *J* = 7.9 Hz, 1H), 4.38 (d, *J* = 14.4 Hz, 1H), 3.39 – 3.33 (m, 1H), 3.27 – 3.19 (m, 1H), 3.02 (q, *J* = 4.1, 3.7 Hz, 2H), 2.25 (dt, *J* = 8.9, 4.3 Hz, 1H), 2.06 (d, *J* = 12.1 Hz, 1H); ^{13}C NMR (75 MHz, chloroform-d) δ 199.4, 159.9, 144.2, 141.6, 136.2, 133.2, 132.4, 129.8, 128.7, 127.5, 127.4, 127.2, 127.2, 126.5, 125.2, 119.4, 47.6, 34.7, 28.9, 28.8. HRMS (ESI) m/z 288.1380 (M+H$^+$), calc. for $C_{20}H_{18}NO$ 288.1383.

The ee was determined by HPLC analysis: Lux® 5μm Amylose-1 (4.6 mm i.d. x 250 mm); Hexane/2-propanol = 85/15; flow rate 1.0 mL/min; 25 °C; 254 nm; retention time: 9.86 min (major) and 11.0 min (minor).

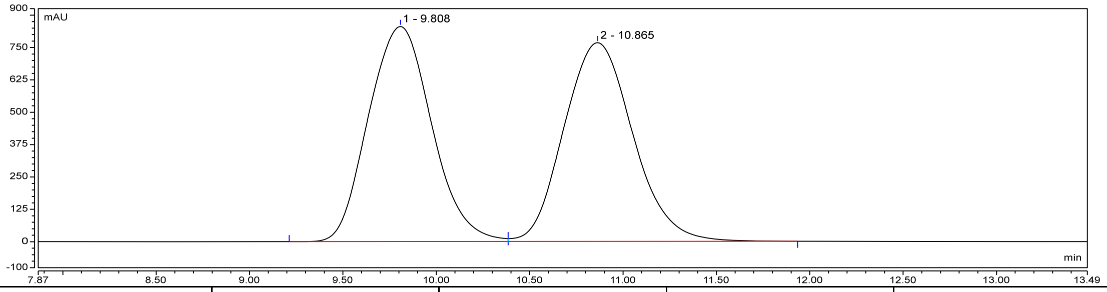

Entry	Retention Time	%Area	Area	Height
1	9.808	49.84	326.0895	830.56
2	10.865	50.16	328.2185	767.49

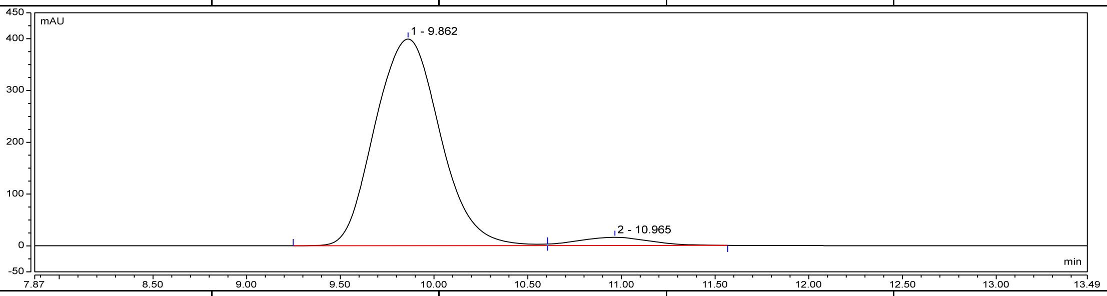

Entry	Retention Time	%Area	Area	Height
1	9.862	95.90	160.6838	398.90
2	10.965	4.10	6.8726	15.71

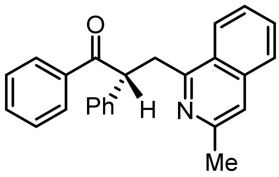

(*R*)-3-(3-methylisoquinolin-1-yl)-1,2-diphenylpropan-1-one (3k): white solid; Mp: 100.8 ºC; 25.6 mg, 73% yield; 91% ee; $[\alpha]_D^{25}$ = 50.5 (*c* 1.0, $CHCl_3$); ^1H NMR (300 MHz, chloroform-d) δ 8.13 – 8.08 (m, 3H), 7.66 (d, *J* = 8.0 Hz, 1H), 7.60 – 7.54 (m, 1H), 7.53 – 7.40 (m, 6H), 7.32 (t, *J* = 7.4 Hz, 2H), 7.26 – 7.20 (m, 2H), 5.69 (dd, *J* = 10.4, 4.0 Hz, 1H), 4.45 (dd, *J* = 16.7, 10.4 Hz, 1H), 3.71 (dd, *J* = 16.7, 4.1 Hz, 1H), 2.32 (s, 3H); ^{13}C NMR (75 MHz, chloroform-d) δ 200.2, 157.7, 149.8, 139.4, 137.9, 136.6, 132.2, 129.6, 128.9, 128.7, 128.5, 128.2, 127.0, 126.5, 125.9, 125.2, 124.7, 117.0, 50.7, 39.4, 23.5. HRMS (ESI) m/z 352.1693 ($M+H^+$), calc. for $C_{25}H_{22}NO$ 352.1696

The ee was determined by HPLC analysis: CHIRALPAK IG (4.6 mm i.d. x 250 mm); hexane/2–propanol = 85/15; flow rate 1.0 mL/min; 25 ºC; 254 nm; retention time: 12.3 min (major) and 19.6 min (minor).

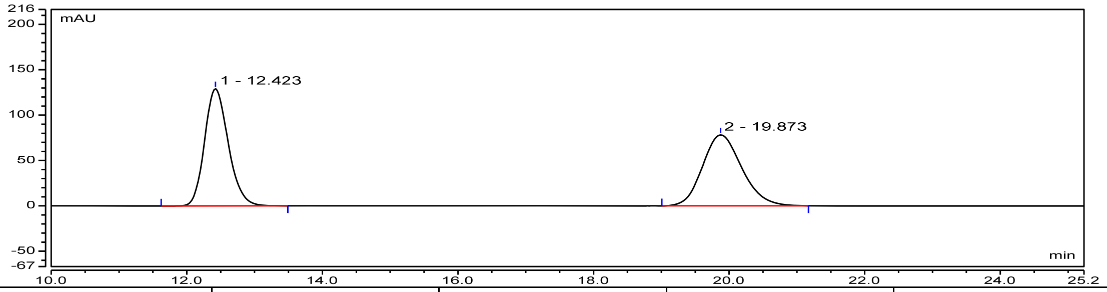

Entry	Retention Time	%Area	Area	Height
1	12.423	49.98	52.3660	129.10
2	19.873	50.02	52.4161	78.13

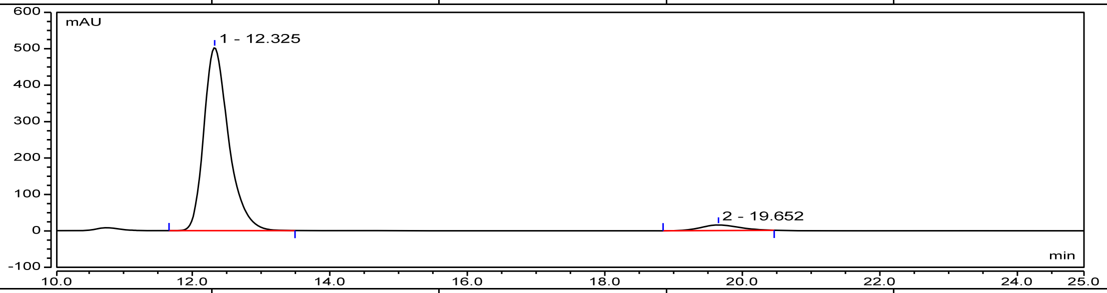

Entry	Retention Time	%Area	Area	Height
1	12.325	95.52	209.8448	502.35
2	19.652	4.48	9.8465	15.30

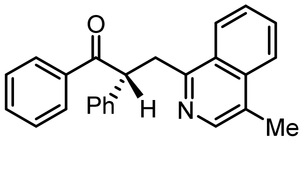

(*R*)-3-(4-methylisoquinolin-1-yl)-1,2-diphenylpropan-1-one (3l): white solid; Mp: 93.1 ºC; 23.5 mg, 67% yield; 91% ee; $[\alpha]_D^{25}$ = –4.1 (*c* 1.0, $CHCl_3$); ^1H NMR (300 MHz, chloroform-d) δ 8.19 (d, *J* = 8.5 Hz, 1H), 8.11 (s, 1H), 8.06 (d, *J* = 7.6 Hz, 2H), 7.90 (d, *J* = 8.4 Hz, 1H), 7.68 (t, *J* = 7.6 Hz, 1H), 7.56 (t, *J* = 7.7 Hz, 1H), 7.50 – 7.37 (m, 5H), 7.29 (t, *J* = 7.9 Hz, 2H), 7.24 – 7.17 (m, 1H), 5.69 (dd, *J* = 10.0, 4.4 Hz, 1H), 4.40 (dd, *J* = 16.3, 9.9 Hz, 1H), 3.65 (dd, *J* = 16.3, 4.4 Hz, 1H), 2.52 (s, 3H); ^{13}C NMR (75 MHz, chloroform-d) δ 199.9, 156.9, 139.5, 137.3, 135.4, 132.4, 129.7, 128.9, 128.8, 128.4, 128.3, 127.1, 126.7, 126.6, 125.7, 125.5, 123.7, 51.4, 38.6, 15.8. HRMS (ESI) m/z 352.1694 ($M+H^+$), calc. for $C_{25}H_{22}NO$ 352.1696.

The ee was determined by HPLC analysis: CHIRALPAK IG (4.6 mm i.d. x 250 mm); hexane/2–propanol = 85/15; flow rate 1.0 mL/min; 25 °C; 254 nm; retention time: 18.7 min (major) and 15.3 min (minor).

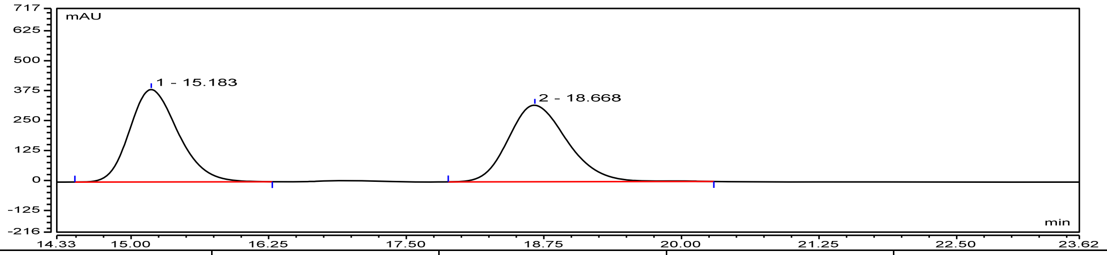

Entry	Retention Time	%Area	Area	Height
1	15.183	49.89	197.5409	385.30
2	18.668	50.11	198.4126	319.39

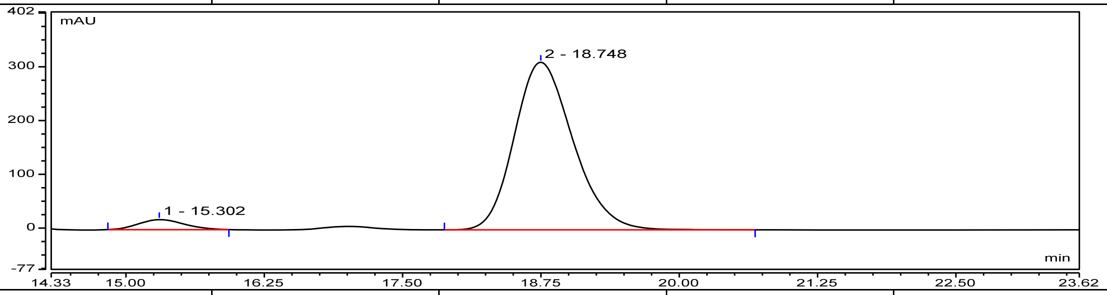

Entry	Retention Time	%Area	Area	Height
1	15.302	4.46	8.9306	18.67
2	18.748	95.54	191.4720	311.55

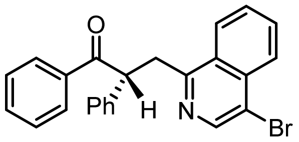

(*R*)-3-(4-bromoisoquinolin-1-yl)-1,2-diphenylpropan-1-one (3m): white solid; Mp: 125.2 °C; 32.5 mg, 78% yield; 93% ee; $[\alpha]_D^{25}$ = 35 (*c* 1.0, $CHCl_3$); ^1H NMR (300 MHz, chloroform-d) δ 8.45 (s, 1H), 8.19 – 8.12 (m, 2H), 8.09 – 8.00 (m, 2H), 7.76 (t, *J* = 7.6 Hz, 1H), 7.62 (t, *J* = 7.6 Hz, 1H), 7.54 – 7.36 (m, 5H), 7.33 – 7.28 (m, 2H), 7.22 (t, *J* = 7.1 Hz, 1H), 5.65 (dd, *J* = 10.0, 4.2 Hz, 1H), 4.40 (dd, *J* = 16.6, 10.0 Hz, 1H), 3.61 (dd, *J* = 16.6, 4.3 Hz, 1H); ^{13}C NMR (75 MHz, chloroform-d) δ 199.6, 158.3, 143.0, 139.2, 137.2, 134.4, 132.6, 131.0, 129.0, 128.8, 128.4, 127.9, 127.2, 126.5, 125.3, 118.0, 51.0, 38.8. HRMS (ESI) m/z 416.0642 ($M+H^+$), calc. for $C_{24}H_{19}BrNO$ 416.0645.

The ee was determined by HPLC analysis: CHIRALPAK IG (4.6 mm i.d. x 250 mm); hexane/2–propanol = 85/15; flow rate 1.0 mL/min; 25 °C; 254 nm; retention time: 15.7 min (major) and 12.4 min (minor).

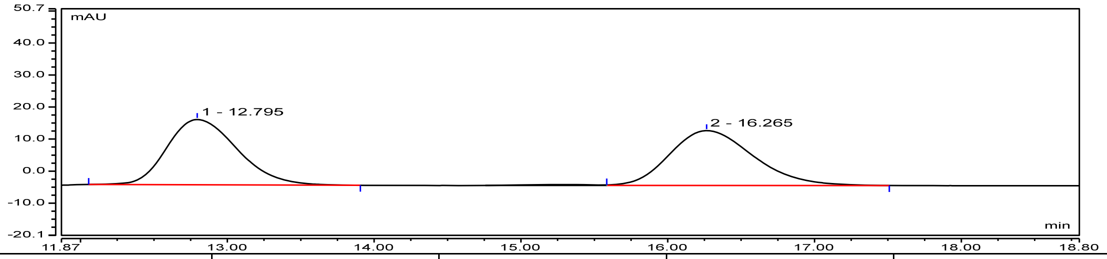

Entry	Retention Time	%Area	Area	Height
1	12.795	50.02	11.3381	20.39
2	16.265	49.98	11.3284	17.11

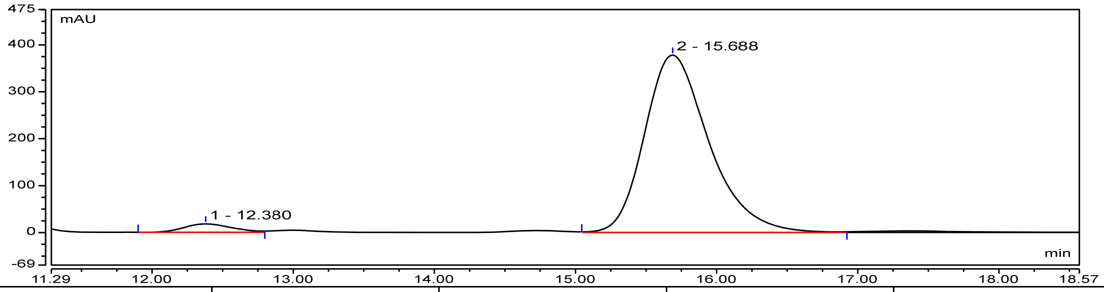

Entry	Retention Time	%Area	Area	Height
1	12.380	3.40	7.0442	17.96
2	15.688	96.60	199.9804	377.79

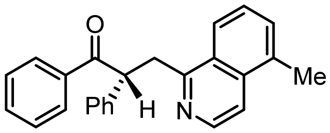

(*R*)-3-(5-methylisoquinolin-1-yl)-1,2-diphenylpropan-1-one (3n): yellow solid; Mp: 126.2 °C; 30.9 mg, 88% yield; 92% ee; $[\alpha]_D^{25}$ = 108.5 (*c* 1.0, $CHCl_3$); ^{1}H NMR (400 MHz, chloroform-d) δ 8.27 (d, *J* = 6.0 Hz, 1H), 8.11 – 7.93 (m, 3H), 7.57 (d, *J* = 6.0 Hz, 1H), 7.48 – 7.37 (m, 5H), 7.36 – 7.32 (m, 2H), 7.28 – 7.20 (m, 2H), 7.18 – 7.13 (m, 1H), 5.74 (dd, *J* = 10.0, 4.6 Hz, 1H), 4.39 (dd, *J* = 16.1, 9.8 Hz, 1H), 3.67 (dd, *J* = 16.2, 4.6 Hz, 1H), 2.59 (s, 3H); ^{13}C NMR (151 MHz, chloroform-d) δ 199.7, 159.0, 139.4, 137.2, 135.5, 134.0, 132.4, 130.6, 129.0, 128.9, 128.4, 128.3, 127.2, 127.1, 126.8, 123.3, 116.0, 51.6, 38.6, 19.0. HRMS (ESI) m/z 352.1693 (M+H$^+$), calc. for $C_{25}H_{22}NO$ 352.1696.

The ee was determined by HPLC analysis: Lux® 5μm Amylose-1 (4.6 mm i.d. x 250 mm); hexane/2–propanol = 85/15; flow rate 1.0 mL/min; 25 °C; 254 nm; retention time: 13.4 min (major) and 18.6 min (minor).

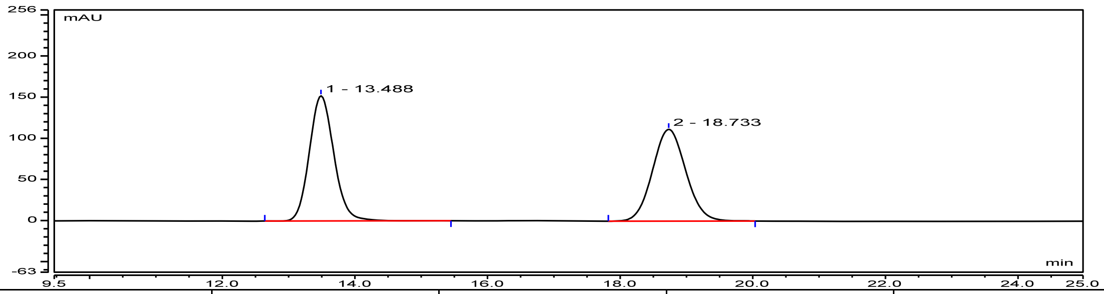

Entry	Retention Time	%Area	Area	Height
1	13.488	50.27	65.5312	152.18
2	18.733	49.73	64.8330	111.53

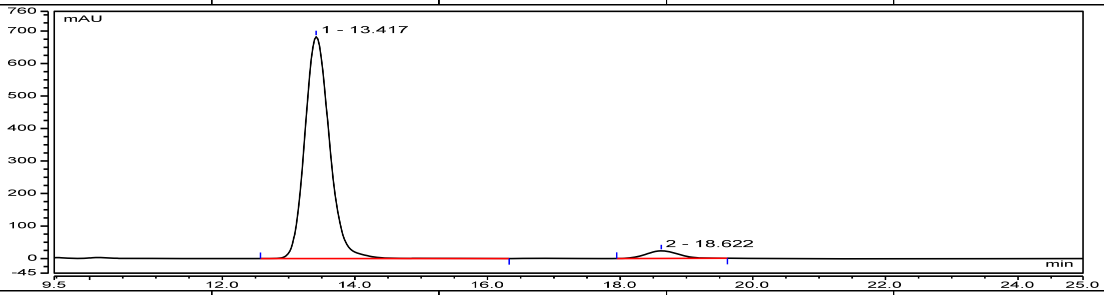

Entry	Retention Time	%Area	Area	Height
1	13.417	95.84	295.0052	682.44
2	18.622	4.16	12.8054	23.01

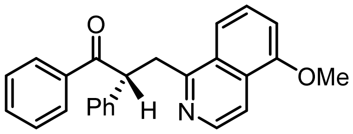

(*R*)-3-(5-methoxyisoquinolin-1-yl)-1,2-diphenylpropan-1-one (3o): white solid; Mp: 130.3 ºC; 9.4 mg, 48% yield; 90% ee; $[\alpha]_D^{25}$ = 28.8 (*c* 1.0, $CHCl_3$); ^1H NMR (300 MHz, chloroform-d) δ 8.27 (d, *J* = 5.9 Hz, 1H), 8.08 – 8.04 (m, 2H), 7.85 (d, *J* = 5.9 Hz, 1H), 7.74 (d, *J* = 8.5 Hz, 1H), 7.51 – 7.38 (m, 6H), 7.38 – 7.26 (m, 2H), 7.23 – 7.16 (m, 1H), 6.96 (d, *J* = 7.7 Hz, 1H), 5.71 (dd, *J* = 9.9, 4.5 Hz, 1H), 4.40 (dd, *J* = 16.3, 9.9 Hz, 1H), 3.98 (s, 3H), 3.66 (dd, *J* = 16.3, 4.5 Hz, 1H); ^{13}C NMR (75 MHz, chloroform-d) δ 199.8, 158.0 , 154.8, 139.5, 137.3, 132.4, 128.9, 128.8, 128.6, 128.4, 128.3, 127.8, 127.0, 116.8, 113.6, 107.3, 55.6, 51.4, 39.0. HRMS (ESI) m/z 368.1638 ($M+H^+$), calc. for $C_{25}H_{22}NO_2$ 368.1646.

The ee was determined by HPLC analysis: CHIRALPAK IG (4.6 mm i.d. x 250 mm); hexane/2–propanol = 85/15; flow rate 1.0 mL/min; 25 ºC; 254 nm; retention time: 22.1 min (major) and 24.7 min (minor).

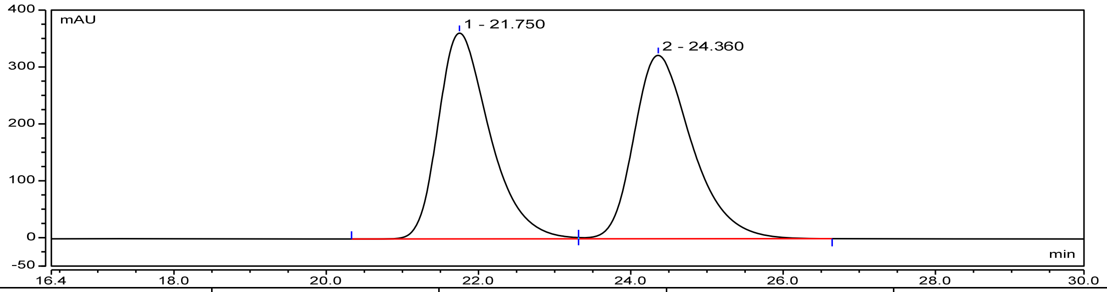

Entry	Retention Time	%Area	Area	Height
1	21.750	49.89	284.0760	361.71
2	24.360	50.11	285.3707	322.45

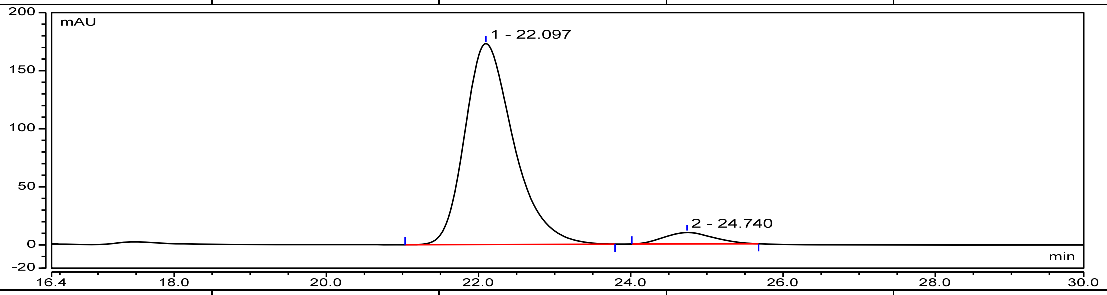

Entry	Retention Time	%Area	Area	Height
1	22.097	94.89	128.4524	172.92
2	24.740	5.11	6.9127	9.49

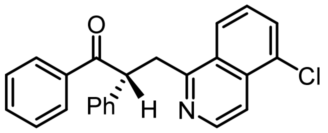

(*R*)-3-(5-chloroisoquinolin-1-yl)-1,2-diphenylpropan-1-one (3p): yellow solid; Mp: 124.0 ºC; 30.5 mg, 82% yield; 93% ee; $[\alpha]_D^{25}$ = 48.3 (*c* 1.0, $CHCl_3$); ^1H NMR (300 MHz, chloroform-d) δ 8.38 (d, *J* = 6.0 Hz, 1H), 8.14 (d, *J* = 8.5 Hz, 1H), 8.10 – 8.00 (m, 2H), 7.86 (d, *J* = 6.0 Hz, 1H), 7.73 (d, *J* = 7.4 Hz, 1H), 7.55 – 7.35 (m, 6H), 7.34 – 7.27 (m, 2H), 7.25 – 7.18 (m, 1H), 5.71 (dd, *J* = 10.0, 4.4 Hz, 1H), 4.43 (dd, *J* = 16.4, 10.0 Hz, 1H), 3.67 (dd, *J* = 16.4, 4.4 Hz, 1H); ^{13}C NMR (75 MHz, chloroform-d) δ 199.6, 159.0, 142.1, 139.2, 137.1, 133.8, 132.5, 131.5, 129.9, 129.0, 128.8, 128.4, 128.0, 127.2, 126.8, 124.1, 115.6, 51.4, 38.9. HRMS (ESI) m/z 372.1148 ($M+H^+$), calc. for $C_{24}H_{19}ClNO$ 372.1150.

The ee was determined by HPLC analysis: CHIRALPAK IG (4.6 mm i.d. x 250 mm) and CHIRALPAK IG (4.6 mm i.d. x 250 mm); hexane/2–propanol = 90/10; flow rate 1.0 mL/min; 25 °C; 254 nm; retention time: 36.7 min (major) and 34.8 min (minor).

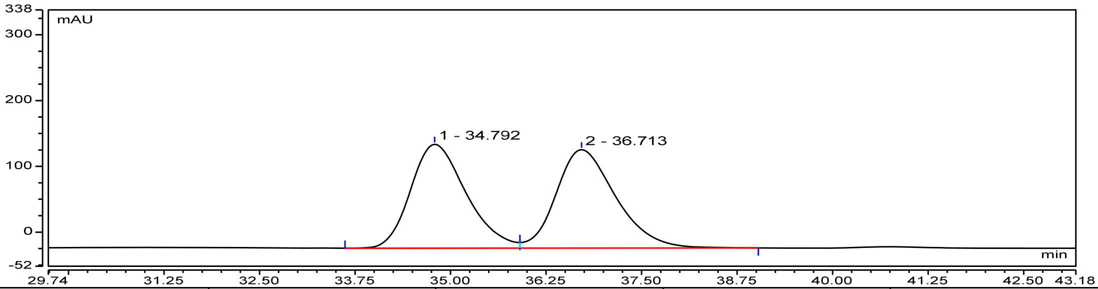

Entry	Retention Time	%Area	Area	Height
1	34.792	49.61	132.8966	157.67
2	36.713	50.39	134.9849	149.43

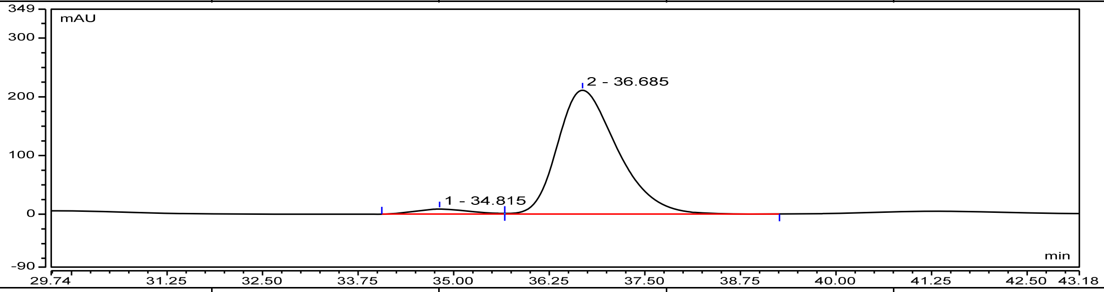

Entry	Retention Time	%Area	Area	Height
1	34.815	3.47	6.8704	8.54
2	36.685	96.53	191.2002	210.83

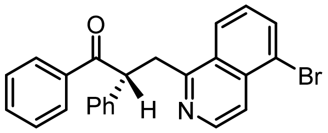

(*R*)-3-(5-bromoisoquinolin-1-yl)-1,2-diphenylpropan-1-one (3q): light yellow solid; Mp: 122.2 °C; 33.3 mg, 80% yield; 93% ee; $[\alpha]_D^{25}$ = 24.6 (*c* 1.0, $CHCl_3$); ^{1}H NMR (300 MHz, chloroform-d) δ 8.37 (d, *J* = 6.0 Hz, 1H), 8.18 (d, *J* = 8.5 Hz, 1H), 8.04 (d, *J* = 7.7 Hz, 2H), 7.92 (d, *J* = 7.5 Hz, 1H), 7.82 (d, *J* = 6.0 Hz, 1H), 7.54 – 7.34 (m, 6H), 7.29 (t, *J* = 7.4 Hz, 2H), 7.24 – 7.17 (m, 1H), 5.69 (dd, *J* = 9.9, 4.3 Hz, 1H), 4.43 (dd, *J* = 16.4, 9.9 Hz, 1H), 3.66 (dd, *J* = 16.3, 4.4 Hz, 1H); ^{13}C NMR (75 MHz, chloroform-d) δ 199.6, 159.2, 142.7, 139.3, 137.2, 135.0, 133.5, 132.5, 129.0, 128.8, 128.4, 128.2, 127.2, 127.2, 124.7, 122.2, 118.1, 51.3, 39.0. HRMS (ESI) m/z 416.0642 (M+H$^+$), calc. for $C_{24}H_{19}BrNO$ 416.0645.

The ee was determined by HPLC analysis: CHIRALPAK IG (4.6 mm i.d. x 250 mm) and CHIRALPAK IG (4.6 mm i.d. x 250 mm); hexane/2–propanol = 90/10; flow rate 1.0 mL/min; 25 °C; 254 nm; retention time: 40.4 min (major) and 38.3 min (minor).

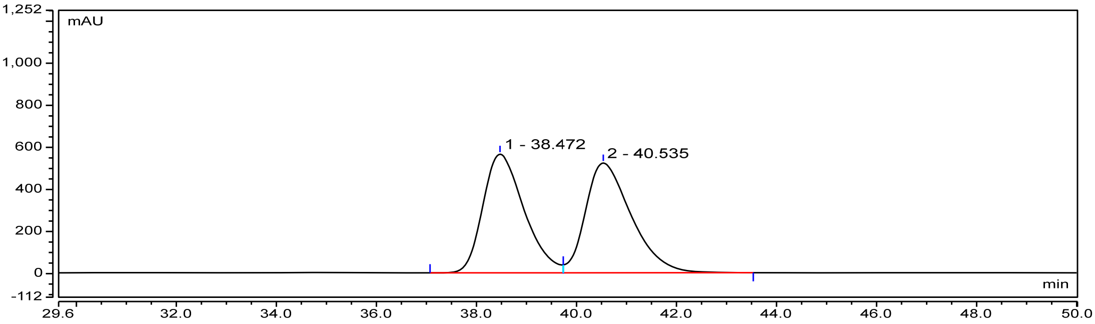

Entry	Retention Time	%Area	Area	Height
1	38.472	49.37	537.9897	563.71
2	40.535	50.63	551.6234	521.56

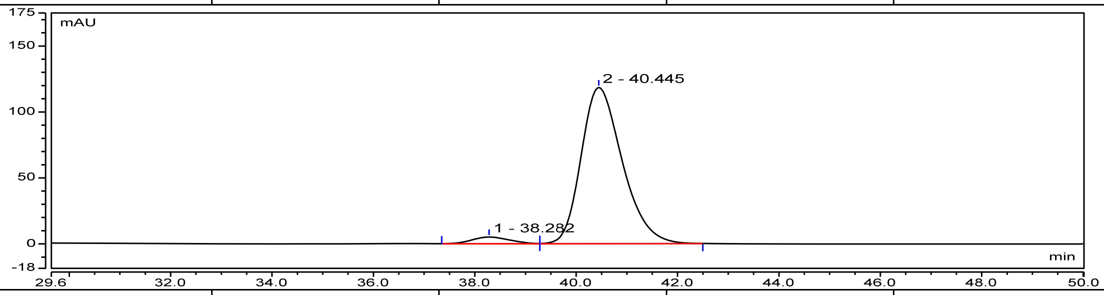

Entry	Retention Time	%Area	Area	Height
1	38.282	3.65	4.2149	5.01
2	40.445	96.35	111.3467	118.26

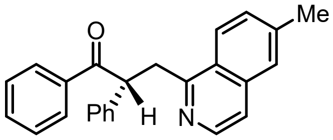

(*R*)-3-(6-methylisoquinolin-1-yl)-1,2-diphenylpropan-1-one (3r): white solid; Mp: 134.0 ºC; 21.1 mg, 60% yield; 90% ee; $[\alpha]_D^{25}$ = 3.6 (*c* 1.0, $CHCl_3$); ^{1}H NMR (300 MHz, chloroform-d) δ 8.22 (d, *J* = 5.7 Hz, 1H), 8.10 – 8.03 (m, 3H), 7.52 (s, 1H), 7.49 – 7.38 (m, 5H), 7.38 – 7.27 (m, 4H), 7.24 – 7.17 (m, 1H), 5.68 (dd, *J* = 9.9, 4.5 Hz, 1H), 4.40 (dd, *J* = 16.2, 9.9 Hz, 1H), 3.64 (dd, *J* = 16.2, 4.5 Hz, 1H), 2.51 (s, 3H); ^{13}C NMR (75 MHz, chloroform-d) δ 199.9, 158.4, 141.5, 139.9, 139.6, 137.3, 136.1, 132.4, 129.1, 128.9, 128.8, 128.4, 128.3, 127.0, 126.0, 125.5, 124.8, 118.8, 51.4, 38.8, 21.8. HRMS (ESI) m/z 352.1693 ($M+H^+$), calc. for $C_{25}H_{22}NO$ 352.1696.

The ee was determined by HPLC analysis: CHIRALPAK IG (4.6 mm i.d. x 250 mm); hexane/2–propanol = 85/15; flow rate 1.0 mL/min; 25 ºC; 254 nm; retention time: 23.0 min (major) and 30.9 min (minor).

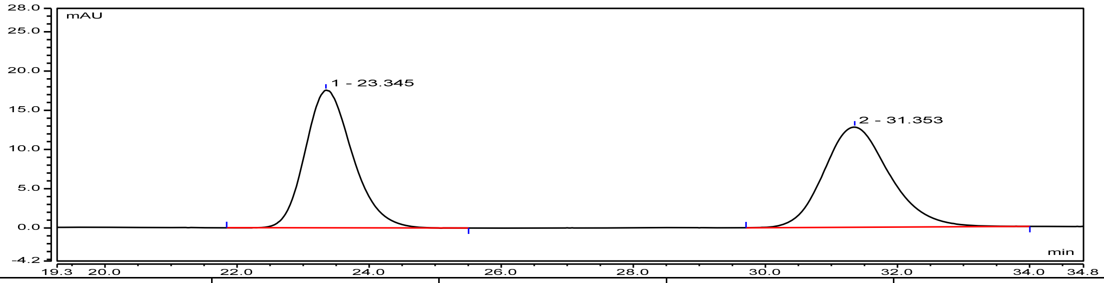

Entry	Retention Time	%Area	Area	Height
1	23.345	49.76	14.6146	17.56
2	31.353	50.24	14.7573	12.78

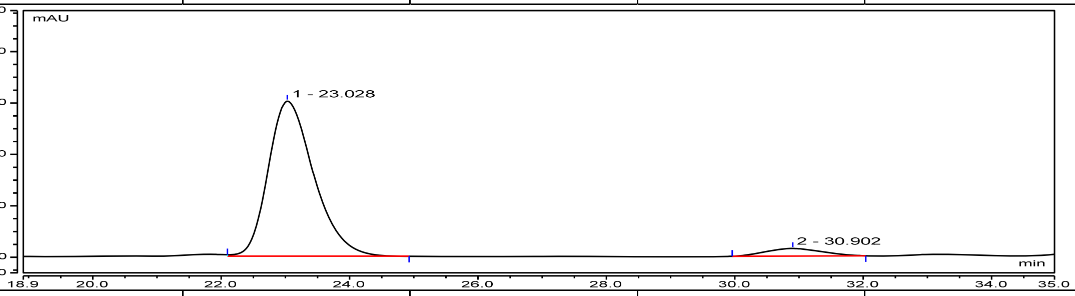

Entry	Retention Time	%Area	Area	Height
1	23.028	94.76	251.8585	301.82
2	30.902	5.24	14.0566	14.38

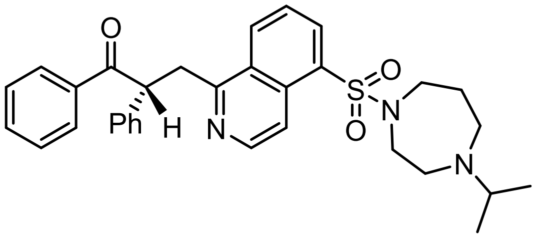

(*R*)-3-(4-((4-isopropyl-1,4-diazepan-1-yl)sulfonyl)isoquinolin-1-yl)-1,2-diphenylpropan-1-one (3s): white solid; Mp: 110.6 °C; 43.9 mg, 81% yield; 91% ee; $[\alpha]_D^{25}$ = 115 (*c* 1.0, $CHCl_3$); ^1H NMR (300 MHz, chloroform-d) δ 8.46 – 8.40 (m, 2H), 8.28 (t, *J* = 7.7 Hz, 1H), 8.20 (d, *J* = 6.2 Hz, 1H), 8.08 – 7.97 (m, 2H), 7.61 (t, *J* = 8.0 Hz, 1H), 7.51 – 7.46 (m, 1H), 7.44 – 7.34 (m, 4H), 7.32 – 7.27 (m, 2H), 7.24 – 7.19 (m, 1H), 5.69 (dt, *J* = 9.8, 3.3 Hz, 1H), 4.89 (p, *J* = 6.2 Hz, 1H), 4.46 (dd, *J* = 16.5, 10.0 Hz, 1H), 3.75 – 3.47 (m, 5H), 3.48 – 3.31 (m, 4H), 1.94 (dp, *J* = 12.5, 6.3 Hz, 2H), 1.20 (d, *J* = 6.3 Hz, 6H). ^{13}C NMR (75 MHz, chloroform-d) δ 199.6, 159.7, 155.5, 143.4, 139.1, 137.0, 134.6, 132.6, 132.4, 131.7, 130.7, 129.1, 128.8, 128.4, 128.33 , 127.8, 127.2, 125.3, 115.7, 68.8, 68.7, 51.2, 49.9, 49.8, 49.3, 48.9, 47.4, 47.3, 45.8, 45.6, 39.3, 29.7, 28.4, 28.1, 22.2. HRMS (ESI) m/z 542.2477 ($M+H^+$), calc. for $C_{32}H_{36}N_3O_3S$ 542.2472.

The ee was determined by HPLC analysis: Chiral INB (4.6 mm i.d. x 250 mm) and Chiral INB (4.6 mm i.d. x 250 mm); hexane/2–propanol = 85/15; flow rate 1.0 mL/min; 25 °C; 254 nm; retention time: 31.7 min (major) and 34.3 min (minor).

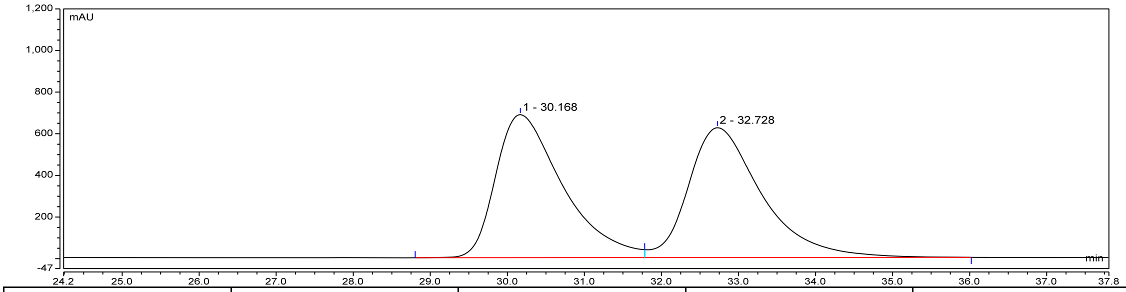

Entry	Retention Time	%Area	Area	Height
1	30.168	50.18	691.7536	686.52
2	32.728	49.82	686.7395	623.29

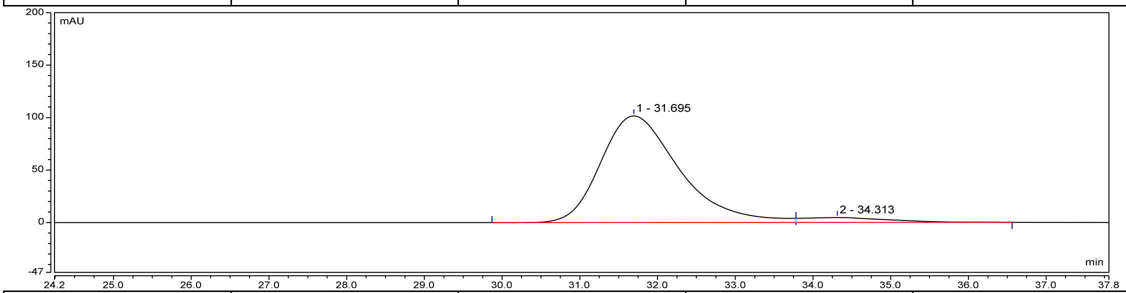

Entry	Retention Time	%Area	Area	Height
1	31.695	95.30	119.2049	101.52
2	34.313	4.70	5.8841	4.70

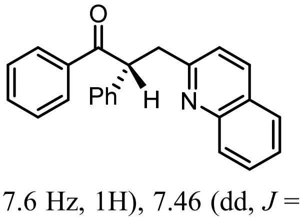

(*R*)-1,2-diphenyl-3-(quinolin-2-yl)propan-1-one (3t): yellow oil; 22.9 mg, 71% yield; 80% ee; $[\alpha]_D^{25}$ = 71.9 (*c* 1.0, $CHCl_3$); ^1H NMR (300 MHz, chloroform-d) δ 8.05 (d, *J* = 7.4 Hz, 2H), 7.96 (d, *J* = 8.4 Hz, 1H), 7.82 (d, *J* = 8.4 Hz, 1H), 7.73 (d, *J* = 8.0 Hz, 1H), 7.60 (t, *J* = 7.6 Hz, 1H), 7.46 (dd, *J* = 7.4, 4.1 Hz, 2H), 7.41 – 7.37 (m, 4H), 7.28 (t, *J* = 7.2 Hz, 2H), 7.22 – 7.16 (m, 2H), 5.54 (dd, *J* = 9.1, 5.6 Hz, 1H), 4.02 (dd, *J* = 15.2, 9.1 Hz, 1H), 3.43 (dd, *J* = 15.2,

5.6 Hz, 1H); ^{13}C NMR (75 MHz, chloroform-d) δ 199.7, 159.8, 147.7, 139.1, 137.2, 135.8, 132.6, 129.1, 128.9, 128.8, 128.7, 128.4, 128.3, 127.4, 127.1, 126.8, 125.7, 122.1, 52.4, 42.9. HRMS (ESI) m/z 338.1536 (M+H$^+$), calc. for $C_{24}H_{22}NO$ 338.1540.

The ee was determined by HPLC analysis: Lux® 5μm Amylose-1 (4.6 mm i.d. x 250 mm); hexane/2–propanol = 85/15; flow rate 1.0 mL/min; 25 °C; 254 nm; retention time: 13.6 min (major) and 10.8 min (minor).

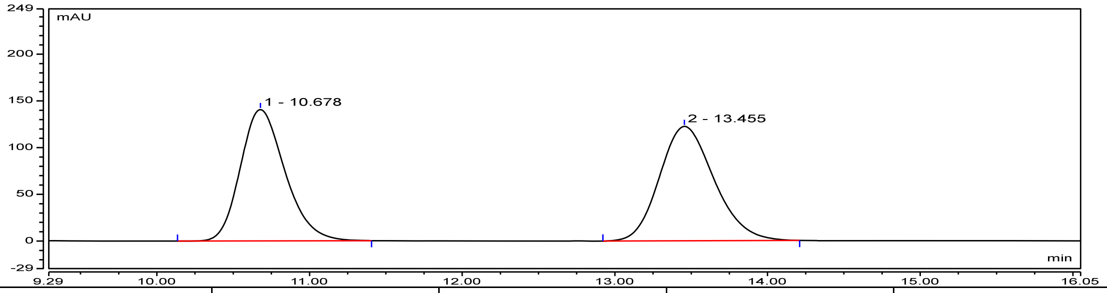

Entry	Retention Time	%Area	Area	Height
1	10.678	49.44	47.6986	140.54
2	13.455	50.56	48.7737	120.70

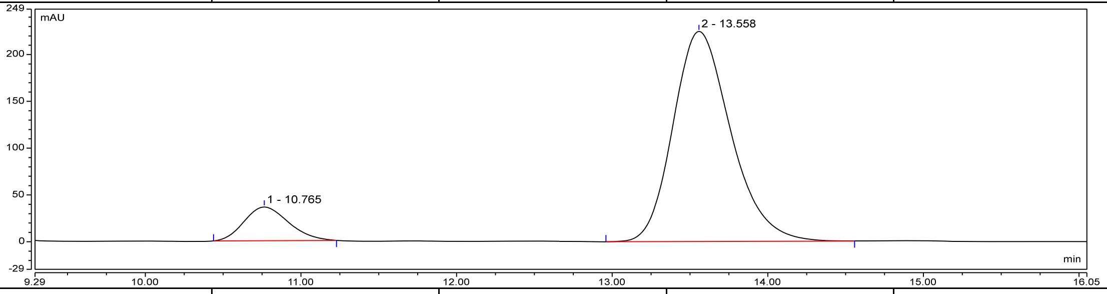

Entry	Retention Time	%Area	Area	Height
1	10.765	9.76	10.4280	34.03
2	13.558	90.24	96.3964	224.40

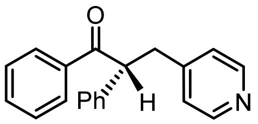

(*R*)-1,2-diphenyl-3-(pyridin-4-yl)propan-1-one (3u): light yellow solid; Mp: 119.4 °C, 22.1 mg, 77% yield; 90% ee; $[\alpha]_D^{25}$ = –51.9 (*c* 1.0, $CHCl_3$); ^{1}H NMR (400 MHz, chloroform-d) δ 8.37 (d, *J* = 5.0 Hz, 2H), 7.90 – 7.80 (m, 2H), 7.46 – 7.41 (m, 1H), 7.34 – 7.31 (m, 2H), 7.27 – 7.21 (m, 2H), 7.21 – 7.16 (m, 3H), 6.99 – 6.95 (m, 2H), 4.76 (t, *J* = 7.3 Hz, 1H), 3.50 (dd, *J* = 13.8, 7.5 Hz, 1H), 3.04 (dd, *J* = 13.7, 7.2 Hz, 1H); ^{13}C NMR (101 MHz, chloroform-d) δ 198.2, 149.4, 148.8, 138.2, 136.2, 133.1, 129.1, 128.7, 128.5, 128.1, 127.5, 124.5, 54.9, 39.3. HRMS (ESI) m/z 288.1381 (M+H$^+$), calc. for $C_{20}H_{18}NO$ 288.1383.

The ee was determined by HPLC analysis: CHIRALPAK IG (4.6 mm i.d. x 250 mm); hexane/2–propanol = 70/30; flow rate 1.0 mL/min; 25 °C; 254 nm; retention time: 11.2 min (major) and 11.6 min (minor).

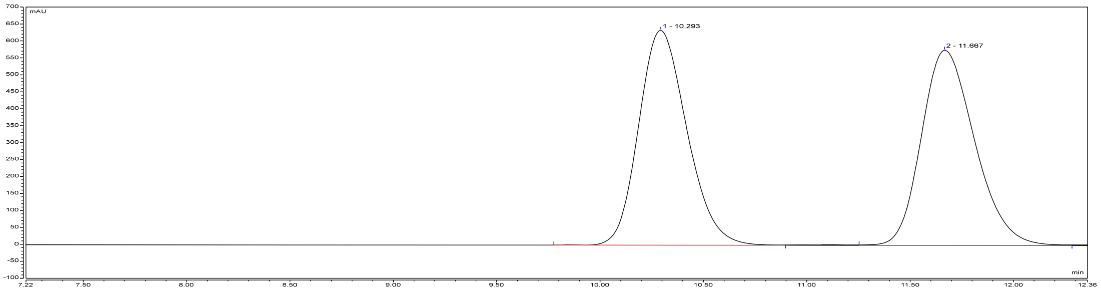

Entry	Retention Time	%Area	Area	Height
1	10.293	49.93	171.3115	633.67
2	11.667	50.07	171.8157	575.73

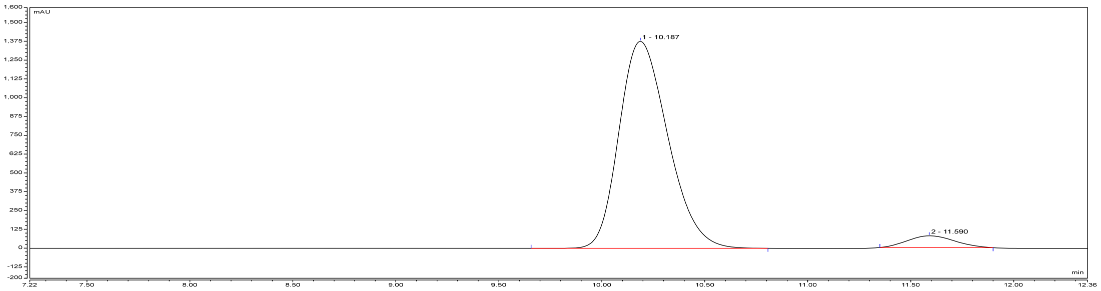

Entry	Retention Time	%Area	Area	Height
1	10.187	95.17	377.8419	1375.90
2	11.590	4.83	19.1563	75.72

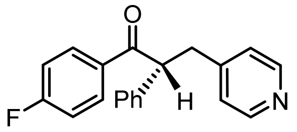

(*R*)-1-(4-fluorophenyl)-2-phenyl-3-(pyridin-4-yl)propan-1-one (3v): yellow solid; Mp: 66.6 ºC; 18.3 mg, 60% yield; 81% ee; $[\alpha]_D^{25}$ = –49 (*c* 1.0, $CHCl_3$); ^1H NMR (400 MHz, chloroform-d) δ 8.38 (d, J = 6.1 Hz, 2H), 8.03 – 7.79 (m, 2H), 7.26 – 7.22 (m, 2H), 7.21 – 7.17 (m, 1H), 7.17 – 7.13 (m, 2H), 7.02 – 6.93 (m, 4H), 4.69 (t, *J* = 7.3 Hz, 1H), 3.48 (dd, *J* = 13.8, 7.4 Hz, 1H), 3.03 (dd, *J* = 13.7, 7.2 Hz, 1H); ^{13}C NMR (101 MHz, chloroform-d) δ 196.7, 165.6 (d, *J* = 255.5 Hz), 149.5, 138.1, 132.6 (d, *J* = 3.0 Hz), 131.4 (d, *J* = 9.4 Hz), 129.2, 128.1, 127.6, 124.5, 115.8, 115.6, 55.0, 39.3; ^{19}F NMR (376 MHz, chloroform-d) δ -104.7. HRMS (ESI) m/z 306.1287 ($M+H^+$), calc. for $C_{20}H_{17}FNO$ 306.1289.

The ee was determined by HPLC analysis: CHIRALPAK IG (4.6 mm i.d. x 250 mm); hexane/2–propanol = 80/20; flow rate 1.0 mL/min; 25 ºC; 254 nm; retention time: 13.0 min (major) and 16.3 min (minor).

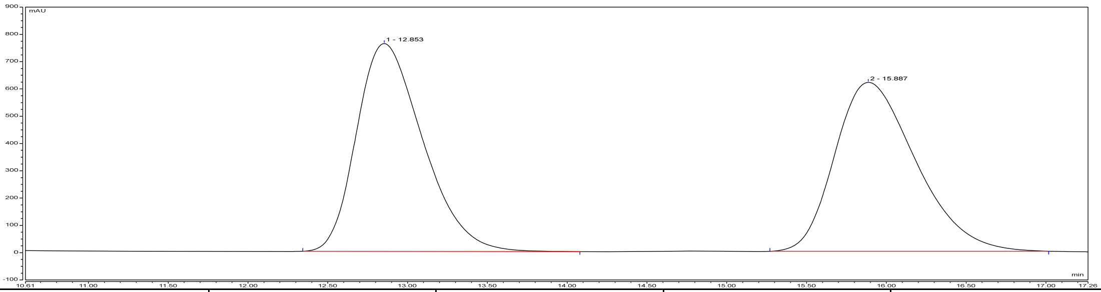

Entry	Retention Time	%Area	Area	Height
1	12.853	50.18	366.6343	761.66
2	15.887	49.82	363.9411	618.99

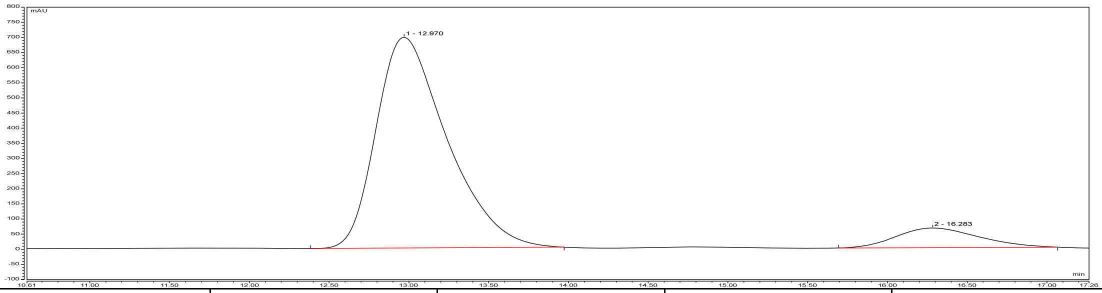

Entry	Retention Time	%Area	Area	Height
1	12.970	90.26	357.7067	696.01
2	16.283	9.74	38.6028	64.71

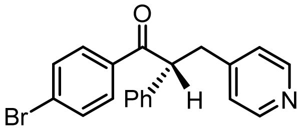

(*R*)-1-(4-bromophenyl)-2-phenyl-3-(pyridin-4-yl)propan-1-one (3w): yellow solid; Mp: 145.8 ºC; 25.9 mg, 71% yield; 90% ee; $[\alpha]_D^{25}$ = –72 (*c* 1.0, $CHCl_3$); ^1H NMR (400 MHz, chloroform-d) δ 8.48 (d, *J* = 5.5 Hz, 2H), 7.82 – 7.79 (m, 2H), 7.56 – 7.54 (m, 2H), 7.38 – 7.26 (m, 3H), 7.24 – 7.22 (m, 2H), 7.05 (d, *J* = 5.7 Hz, 2H), 4.77 (t, *J* = 7.3 Hz, 1H), 3.58 (dd, *J* = 13.8, 7.3 Hz, 1H), 3.12 (dd, *J* = 13.8, 7.3 Hz, 1H); ^{13}C NMR (101 MHz, chloroform-d) δ 197.2, 149.5, 148.6, 137.8, 134.8, 131.84, 130.2, 129.2, 128.3, 128.0, 127.6, 124.5, 55.0, 39.2. HRMS (ESI) m/z 366.0486 ($M+H^+$), calc. for $C_{20}H_{17}BrNO$ 366.0489.

The ee was determined by HPLC analysis: CHIRALPAK IG (4.6 mm i.d. x 250 mm); hexane/2–propanol = 80/20; flow rate 1.0 mL/min; 25 ºC; 254 nm; retention time: 16.0 min (major) and 22.8 min (minor).

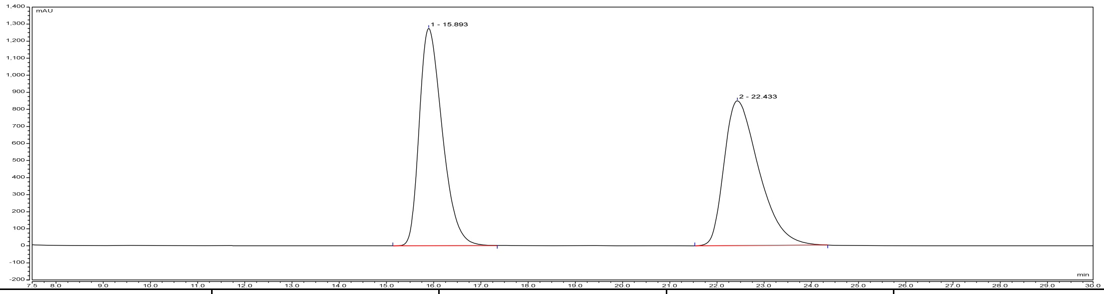

Entry	Retention Time	%Area	Area	Height
1	15.893	49.81	718.1363	1273.97
2	22.433	50.19	723.6166	848.55

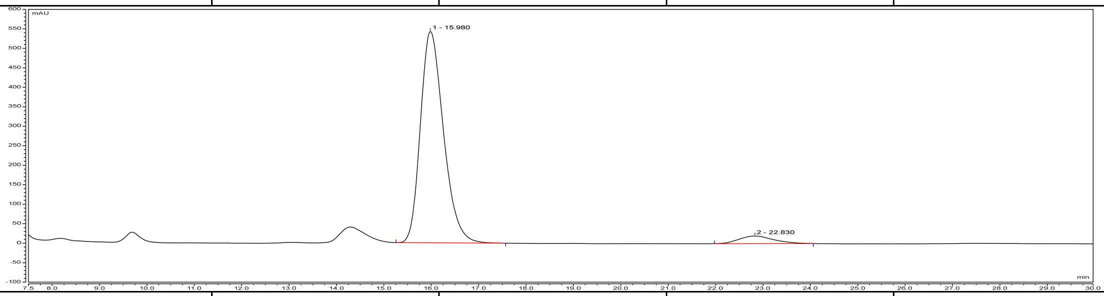

Entry	Retention Time	%Area	Area	Height
1	15.980	94.89	306.0681	542.24
2	22.830	5.11	16.4816	19.54

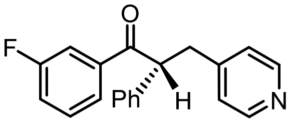

(*R*)-1-(3-fluorophenyl)-2-phenyl-3-(pyridin-4-yl)propan-1-one (3x): yellow liquid; 21.4 mg, 70% yield; 90% ee; $[\alpha]_D^{25}$ = –19.8 (*c* 1.0, $CHCl_3$); ^{1}H NMR (400 MHz, chloroform-d) δ 8.50 (d, *J* = 4.8 Hz, 2H), 7.73 (d, *J* = 7.8 Hz, 1H), 7.64 (dt, *J* = 9.5, 2.1 Hz, 1H), 7.44 – 7.29 (m, 5H), 7.25 – 7.22 (m, 2H), 7.08 (d, *J* = 4.9 Hz, 2H), 4.78 (t, *J* = 7.3 Hz, 1H), 3.59 (dd, *J* = 13.8, 7.4 Hz, 1H), 3.14 (dd, *J* = 13.7, 7.2 Hz, 1H); ^{13}C NMR (101 MHz, chloroform-d) δ 197.0, 162.7 (d, *J* = 248.0 Hz), 149.2, 149.0, 138.3, 137.7, 130.2(d, *J* = 7.7 Hz), 129.3, 128.1, 127.7, 124.4(d, *J* = 3.1 Hz), 120.2(d, *J* = 21.6 Hz), 115.5(d, *J* = 22.5 Hz), 55.2, 39.3; ^{19}F NMR (376 MHz, chloroform-d) δ -111.51. HRMS (ESI) m/z 306.1287 (M+H$^+$), calc. for $C_{20}H_{17}FNO$ 306.1289. The ee was determined by HPLC analysis: CHIRALPAK IG (4.6 mm i.d. x 250 mm); hexane/2–propanol = 85/15; flow rate 1.0 mL/min; 25 ºC; 254 nm; retention time: 16.4 min (major) and 20.9 min (minor).

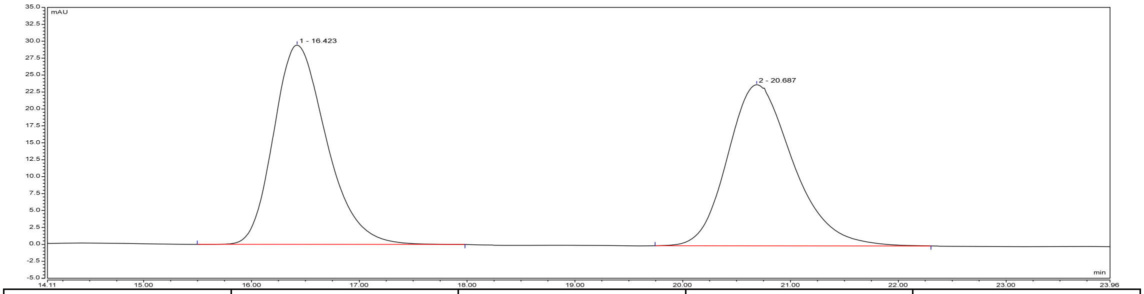

Entry	Retention Time	%Area	Area	Height
1	16.423	49.93	16.7099	29.43
2	20.687	50.07	16.7547	23.79

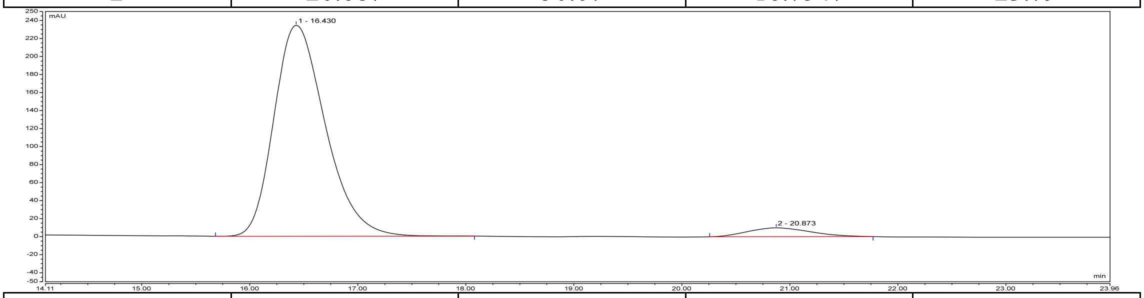

Entry	Retention Time	%Area	Area	Height
1	16.430	95.14	129.0165	234.27
2	20.873	4.86	6.5913	9.86

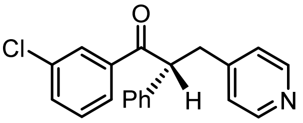

(*R*)-1-(3-chlorophenyl)-2-phenyl-3-(pyridin-4-yl)propan-1-one (3y): yellow liquid; 21.6 mg, 67% yield; 83% ee; $[\alpha]_D^{25}$ = –23.3 (*c* 1.0, $CHCl_3$); ^{1}H NMR (400 MHz, chloroform-d) δ 8.47 (d, *J* = 5.5 Hz, 2H), 7.92 (t, *J* = 1.9 Hz, 1H), 7.79 (d, *J* = 7.8 Hz, 1H), 7.48 (dd, *J* = 7.9, 2.1 Hz, 1H), 7.36 – 7.32 (m, 3H), 7.30 – 7.27 (m, 1H), 7.25 – 7.21 (m, 2H), 7.06 (d, *J* = 5.4 Hz, 2H), 4.77 (t, *J* = 7.3 Hz, 1H), 3.57 (dd, *J* = 13.7, 7.4 Hz, 1H), 3.12 (dd, *J* = 13.7, 7.2 Hz, 1H); ^{13}C NMR (101 MHz, chloroform-d) δ 196.9, 149.2, 149.0, 137.7, 137.6, 134.9, 133.0, 129.9, 129.3, 128.7, 128.1, 127.7, 126.7, 124.6, 55.1, 39.2. HRMS (ESI) m/z 322.0992 (M+H$^+$), calc. for $C_{20}H_{17}ClNO$ 322.0994.

The ee was determined by HPLC analysis: CHIRALPAK IG (4.6 mm i.d. x 250 mm); hexane/2–propanol = 85/15; flow rate 1.0 mL/min; 25 ºC; 254 nm; retention time: 16.0 min (major) and 18.9 min (minor).

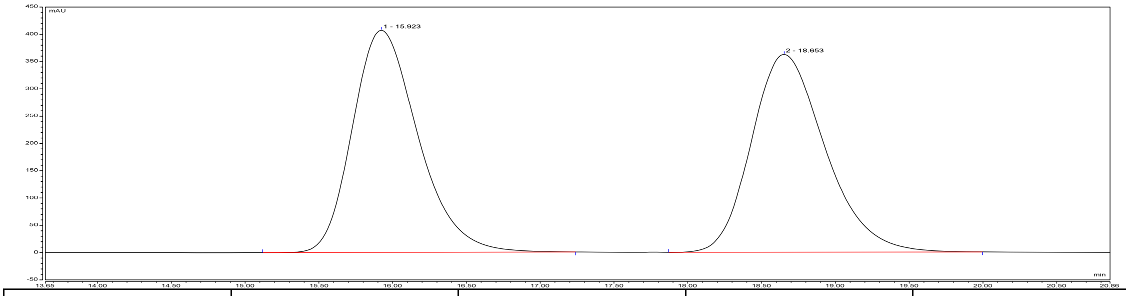

Entry	Retention Time	%Area	Area	Height
1	15.923	50.01	209.8883	406.88
2	18.653	49.99	209.7866	362.26

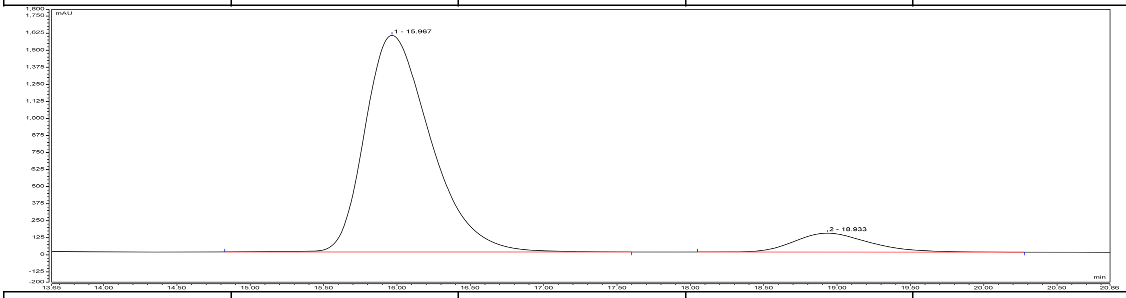

Entry	Retention Time	%Area	Area	Height
1	15.967	91.30	208.5254	405.51
2	18.933	8.70	19.8726	34.73

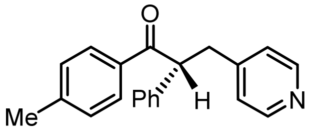

(*R*)-2-phenyl-3-(pyridin-4-yl)-1-(p-tolyl)propan-1-one (3za): yellow solid; Mp: 118.2 ºC, 22.0 mg, 73% yield; 90% ee; $[\alpha]_D^{25}$ = –72.1 (*c* 1.0, $CHCl_3$); ^{1}H NMR (400 MHz, chloroform-d) δ 8.39 (d, *J* = 5.0 Hz, 2H), 7.78 (d, *J* = 8.0 Hz, 2H), 7.24 (t, *J* = 4.2 Hz, 2H), 7.21 – 7.16 (m, 3H), 7.13 (d, *J* = 8.0 Hz, 2H), 7.02 (d, *J* = 5.0 Hz, 2H), 4.74 (t, *J* = 7.3 Hz, 1H), 3.52 (dd, *J* = 13.8, 7.5 Hz, 1H), 3.06 (dd, *J* = 13.7, 7.1 Hz, 1H), 2.31 (s, 3H); ^{13}C NMR (101 MHz, chloroform-d) δ 197.7, 150.0, 148.6, 144.1, 138.4, 133.6, 129.3, 129.1, 128.8, 127.5, 124.8, 54.7, 39.4, 21.6. HRMS (ESI) m/z 302.1538 ($M+H^+$), calc. for $C_{21}H_{20}NO$ 302.1540.

The ee was determined by HPLC analysis: CHIRALPAK IG (4.6 mm i.d. x 250 mm); hexane/2–propanol = 75/25; flow rate 1.0 mL/min; 25 ºC; 254 nm; retention time: 15.4 min (major) and 23.0 min (minor).

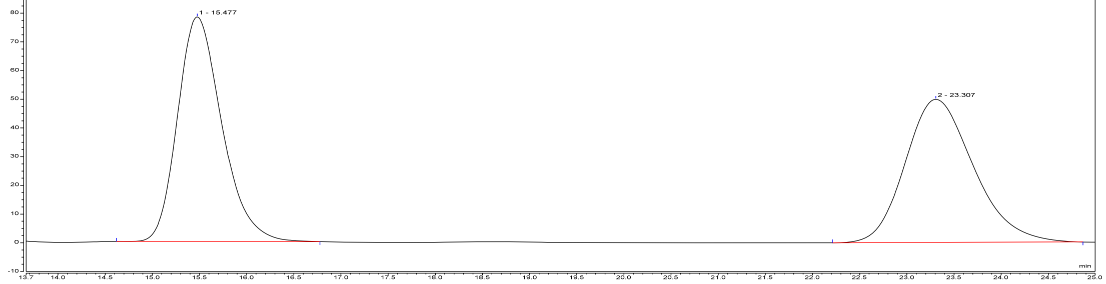

Entry	Retention Time	%Area	Area	Height
1	15.477	50.54	42.6088	78.24
2	23.307	49.46	41.7023	49.84

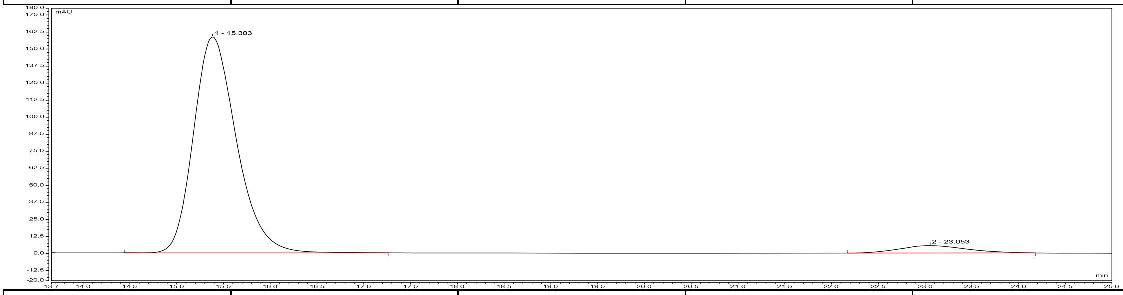

Entry	Retention Time	%Area	Area	Height
1	15.383	95.03	84.1721	158.44
2	23.053	4.97	4.4004	5.40

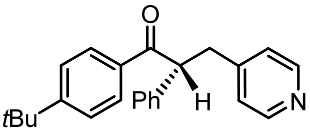

(*R*)-1-(4-(*tert*-butyl)phenyl)-2-phenyl-3-(pyridin-4-yl)propan-1-one (3zb): yellow solid; Mp: 77.9 °C; 27.1 mg, 79% yield; 90% ee; $[\alpha]_D^{25}$ = –148.5 (*c* 1.0, $CHCl_3$); ^1H NMR (400 MHz, chloroform-d) δ 8.48 (d, *J* = 5.1 Hz, 2H), 7.93 (d, *J* = 8.3 Hz, 2H), 7.44 (d, *J* = 8.3 Hz, 2H), 7.38 – 7.27 (m, 5H), 7.07 (d, *J* = 5.2 Hz, 2H), 4.86 (t, *J* = 7.3 Hz, 1H), 3.60 (dd, *J* = 13.7, 7.6 Hz, 1H), 3.12 (dd, *J* = 13.7, 7.0 Hz, 1H), 1.34 (s, 9H); ^{13}C NMR (101 MHz, chloroform-d) δ 197.7, 156.9, 149.4, 149.0, 138.5, 133.6, 129.0, 128.6, 128.1, 127.4, 125.5, 124.5, 54.7, 39.4, 35.0, 30.9. HRMS (ESI) m/z 344.2006 ($M+H^+$), calc. for $C_{24}H_{26}NO$ 344.2009.

The ee was determined by HPLC analysis: CHIRALPAK IG (4.6 mm i.d. x 250 mm); hexane/2–propanol = 80/20; flow rate 1.0 mL/min; 25 °C; 254 nm; retention time: 11.9 min (major) and 17.6 min (minor).

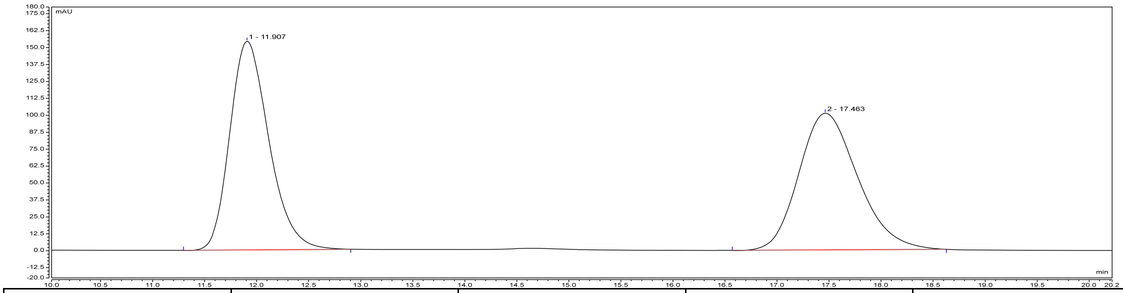

Entry	Retention Time	%Area	Area	Height
1	11.907	50.37	67.0642	153.98
2	17.463	49.63	66.0686	100.89

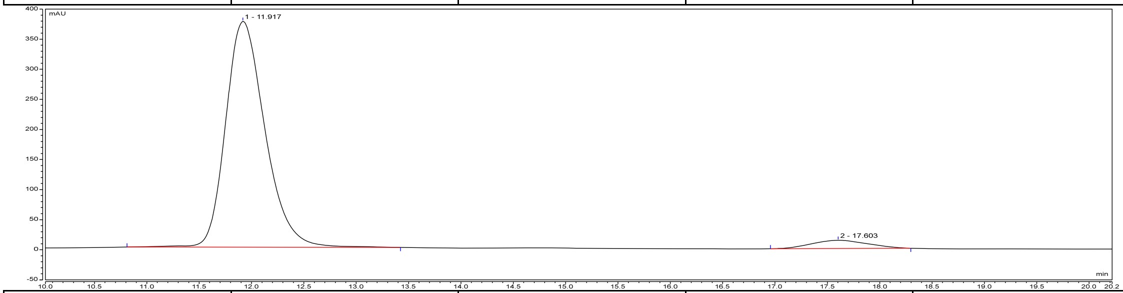

Entry	Retention Time	%Area	Area	Height
1	11.917	95.14	163.6468	375.43
2	17.603	4.86	8.3605	13.58

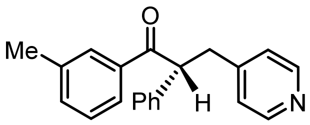

(*R*)-2-phenyl-3-(pyridin-4-yl)-1-(m-tolyl)propan-1-one (3zc): white solid; 21.1 mg, 70% yield; 91% ee; $[\alpha]_D^{25}$ = –146.6 (*c* 1.0, $CHCl_3$); ^{1}H NMR (300 MHz, chloroform-d) δ 8.43 (d, *J* = 5.0 Hz, 2H), 7.88 – 7.63 (m, 2H), 7.32 – 7.28 (m, 4H), 7.26 – 7.17 (m, 3H), 7.03 (d, *J* = 5.1 Hz, 2H), 4.82 (t, *J* = 7.3 Hz, 1H), 3.56 (dd, *J* = 13.8, 7.5 Hz, 1H), 3.09 (dd, *J* = 13.7, 7.1 Hz, 1H), 2.36 (s, 3H); ^{13}C NMR (75 MHz, chloroform-d) δ 198.4, 149.5, 148.9, 138.4, 138.3, 136.2, 133.9, 129.2, 129.1, 128.4, 128.1, 127.4, 125.9, 124.5, 54.8, 39.3, 21.3. HRMS (ESI) m/z 302.1537 (M+H$^+$), calc. for $C_{21}H_{20}NO$ 302.1540.

The ee was determined by HPLC analysis: CHIRALPAK IG (4.6 mm i.d. x 250 mm); hexane/2–propanol = 85/15; flow rate 1.0 mL/min; 25 °C; 254 nm; retention time: 17.9 min (major) and 20.7 min (minor).

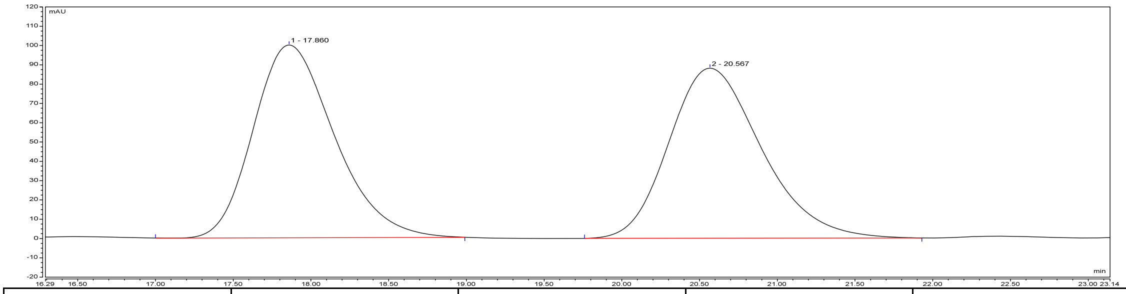

Entry	Retention Time	%Area	Area	Height
1	17.860	49.85	60.4167	99.87
2	20.567	50.15	60.7823	88.09

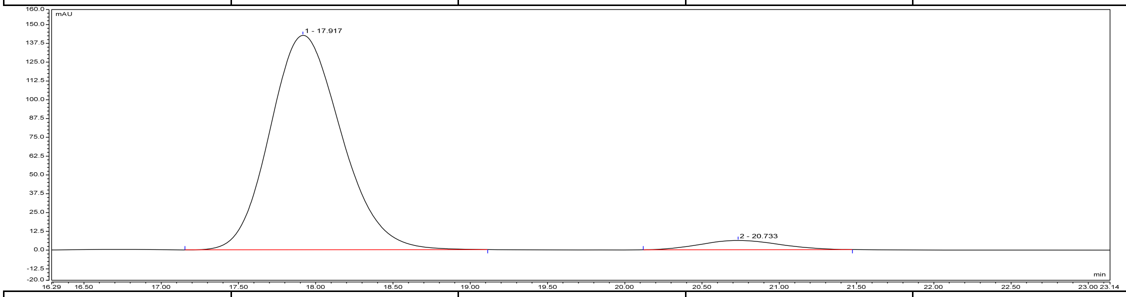

Entry	Retention Time	%Area	Area	Height
1	17.917	95.41	76.4272	142.58
2	20.733	4.59	3.6745	6.17

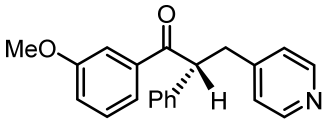

(*R*)-1-(3-methoxyphenyl)-2-phenyl-3-(pyridin-4-yl)propan-1-one (3zd): yellow liquid; 17.1 mg, 54% yield; 91% ee; $[\alpha]_D^{25}$ = –110 (*c* 1.0, $CHCl_3$); ^{1}H NMR (400 MHz, chloroform-d) δ 8.45 (d, *J* = 5.7 Hz, 2H), 7.51 – 7.45 (m, 2H), 7.32 – 7.30 (m, 3H), 7.26 – 7.21 (m, 3H), 7.07 – 7.01 (m, 3H), 4.80 (t, *J* = 7.3 Hz, 1H), 3.80 (s, 3H), 3.56 (dd, *J* = 13.8, 7.5 Hz, 1H), 3.11 (dd, *J* = 13.7, 7.1 Hz, 1H); ^{13}C NMR (101 MHz, chloroform-d) δ 198.0, 159.7, 149.1, 149.0, 138.2, 137.5, 129.5, 129.1, 128.1, 127.5, 124.6, 121.2, 119.6, 113.1, 55.3, 55.0, 39.4. HRMS (ESI) m/z 318.1486 (M+H$^+$), calc. for $C_{21}H_{20}NO_2$ 318.1489.

The ee was determined by HPLC analysis: CHIRALPAK IG (4.6 mm i.d. x 250 mm); hexane/2–propanol = 85/15; flow rate 1.0 mL/min; 25 ºC; 254 nm; retention time: 27.8 min (major) and 32.3 min (minor).

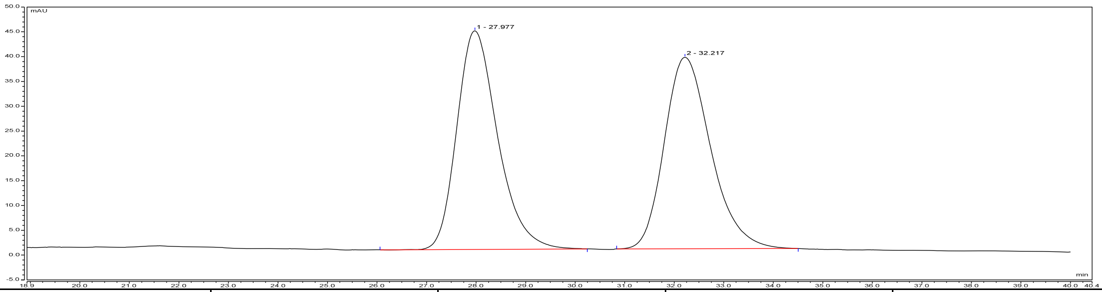

Entry	Retention Time	%Area	Area	Height
1	27.837	95.46	228.1795	243.36
2	32.290	4.54	10.8409	10.62

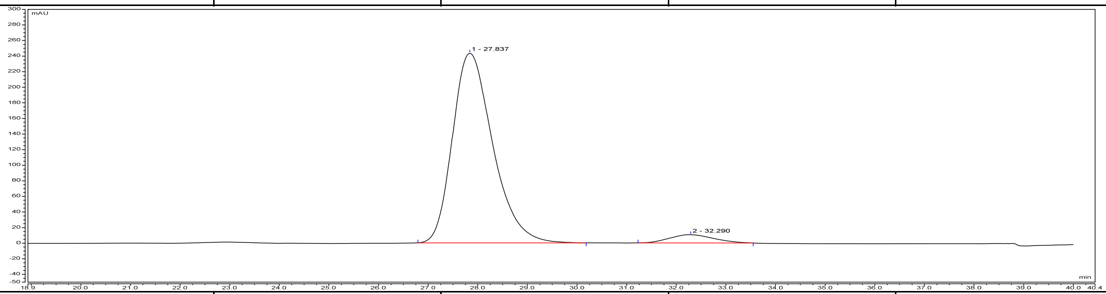

Entry	Retention Time	%Area	Area	Height
1	27.837	95.46	228.1795	243.36
2	32.290	4.54	10.8409	10.62

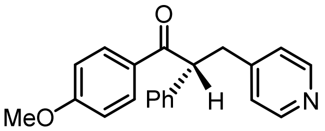

(*R*)-1-(4-methoxyphenyl)-2-phenyl-3-(pyridin-4-yl)propan-1-one (3ze): yellow solid; Mp: 117.5 ºC; 16.5 mg, 52% yield; 85% ee; $[\alpha]_D^{25}$ = –19.8 (*c* 1.0, $CHCl_3$); ^1H NMR (400 MHz, chloroform-d) δ 8.49 (d, *J* = 5.1 Hz, 2H), 8.01 – 7.93 (m, 2H), 7.40 – 7.26 (m, 5H), 7.12 – 7.06 (m, 2H), 6.94 – 6.88 (m, 2H), 4.83 (t, *J* = 7.3 Hz, 1H), 3.87 (S, 3H), 3.61 (dd, *J* = 13.7, 7.5 Hz, 1H), 3.14 (dd, *J* = 13.7, 7.1 Hz, 1H); ^{13}C NMR (101 MHz, chloroform-d) δ 196.6, 163.4, 149.2, 138.7, 131.0, 129.1, 129.0, 128.0, 127.3, 124.5, 113.7, 55.4, 54.4, 39.3. HRMS (ESI) m/z 318.1488 ($M+H^+$), calc. for $C_{21}H_{20}NO_2$ 318.1489.

The ee was determined by HPLC analysis: CHIRALPAK IG (4.6 mm i.d. x 250 mm); hexane/2–propanol = 70/30; flow rate 1.0 mL/min; 25 ºC; 254 nm; retention time: 16.8 min (major) and 25.4 min (minor).

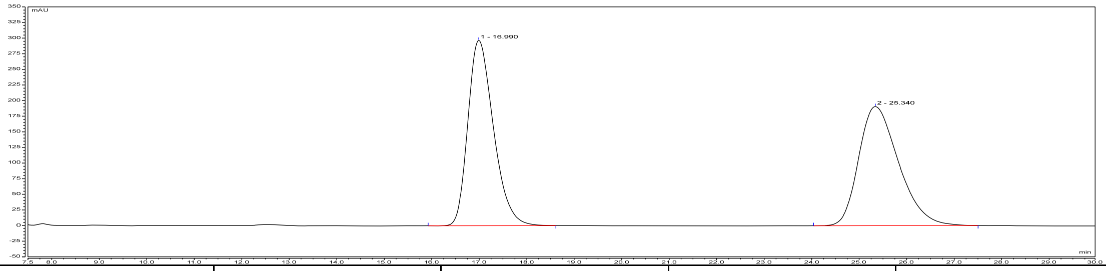

Entry	Retention Time	%Area	Area	Height
1	16.990	49.92	184.9396	296.64
2	25.340	50.08	185.5655	190.60

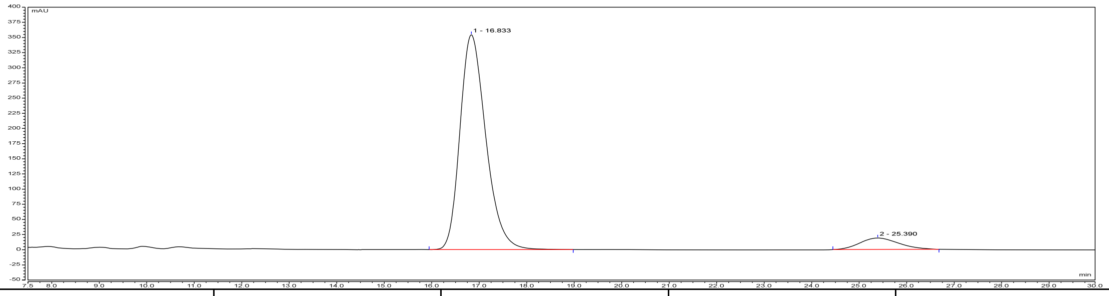

Entry	Retention Time	%Area	Area	Height
1	16.833	92.64	223.3953	354.38
2	25.390	7.36	17.7570	18.85

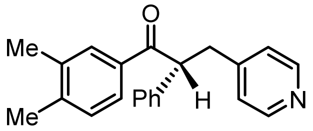

(*R*)-1-(3,4-dimethylphenyl)-2-phenyl-3-(pyridin-4-yl)propan-1-one (3zf): yellow solid; Mp: 73.7 ºC; 20.2 mg, 64% yield; 91% ee; $[\alpha]^{25}_{D}$ = –81.9 (*c* 1.0, $CHCl_3$); ^1H NMR (400 MHz, chloroform-d) δ 8.46 (d, *J* = 5.3 Hz, 2H), 7.73 (s, 1H), 7.66 (dd, *J* = 7.9, 1.9 Hz, 1H), 7.32 – 7.29 (m, 3H), 7.26 – 7.24 (m, 3H), 7.14 (d, *J* = 7.9 Hz, 1H), 7.12 – 7.05 (m, 2H), 4.82 (t, *J* = 7.3 Hz, 1H), 3.58 (dd, *J* = 13.8, 7.6 Hz, 1H), 3.11 (dd, *J* = 13.7, 7.0 Hz, 1H), 2.27 (s, 6H); ^{13}C NMR (101 MHz, chloroform-d) δ 198.0, 149.9, 148.8, 142.9, 138.5, 137.0, 134.0, 129.8, 129.8, 129.1, 128.1, 127.4, 126.5, 124.7, 54.6, 39.4, 19.9, 19.8. HRMS (ESI) m/z 316.1696 $(M+H^+)$, calc. for $C_{22}H_{22}NO$ 316.1696.

The ee was determined by HPLC analysis: CHIRALPAK IG (4.6 mm i.d. x 250 mm); hexane/2–propanol = 80/20; flow rate 1.0 mL/min; 25 ºC; 254 nm; retention time: 16.1 min (major) and 20.8 min (minor).

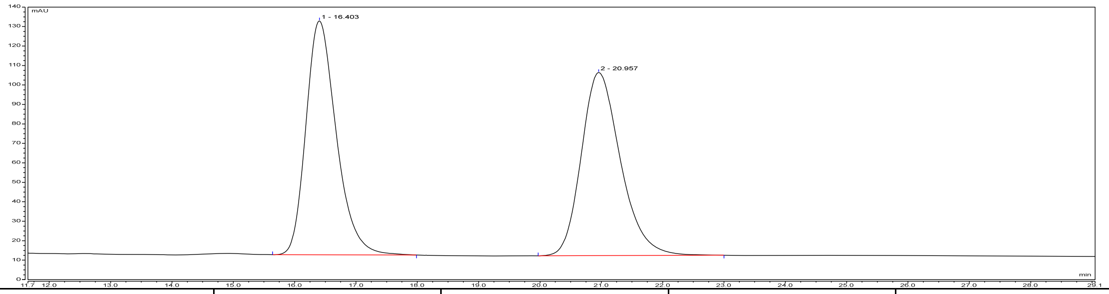

Entry	Retention Time	%Area	Area	Height
1	16.403	49.80	68.9973	120.13
2	20.957	50.20	69.5436	94.03

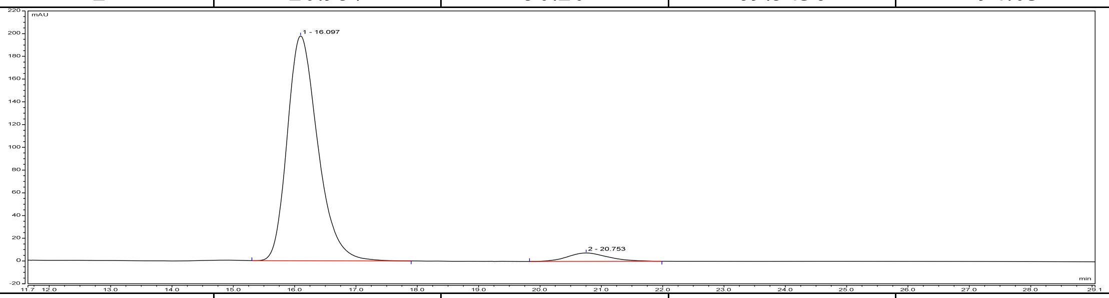

Entry	Retention Time	%Area	Area	Height
1	16.097	95.35	114.0178	197.69
2	20.753	4.65	5.5635	7.34

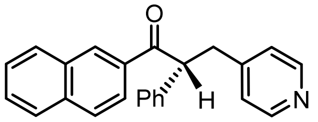

(*R*)-1-(naphthalen-2-yl)-2-phenyl-3-(pyridin-4-yl)propan-1-one (3zg): yellow solid; Mp: 61.6 °C; 17.9 mg, 53% yield; 88% ee; $[\alpha]_D^{25}$ = –68.2 (*c* 1.0, $CHCl_3$); ^{1}H NMR (400 MHz, chloroform-d) δ 8.53 – 8.42 (m, 3H), 8.00 (dd, *J* = 8.6, 1.8 Hz, 1H), 7.89 (d, *J* = 8.1 Hz, 1H), 7.82 (d, *J* = 8.6 Hz, 2H), 7.60 – 7.50 (m, 2H), 7.31 – 7.28 (m, 4H), 7.25 – 7.21 (m, 1H), 7.08 (d, J = 5.2 Hz, 2H), 4.99 (t, *J* = 7.3 Hz, 1H), 3.63 (dd, *J* = 13.8, 7.4 Hz, 1H), 3.17 (dd, *J* = 13.8, 7.2 Hz, 1H); ^{13}C NMR (101 MHz, chloroform-d) δ 198.2, 149.3, 149.2, 138.3, 135.4, 133.5, 132.3, 130.5, 129.6, 129.1, 128.6, 128.4, 128.1, 127.6, 127.5, 126.7, 124.6, 124.2, 54.9, 39.4. HRMS (ESI) m/z 338.1538 (M+H$^+$), calc. for $C_{24}H_{20}NO$ 338.1540.The ee was determined by HPLC analysis: CHIRALPAK IG (4.6 mm i.d. x 250 mm); hexane/2–propanol = 75/25; flow rate 1.0 mL/min; 25 °C; 254 nm; retention time: 21.3 min (major) and 25.9 min (minor).

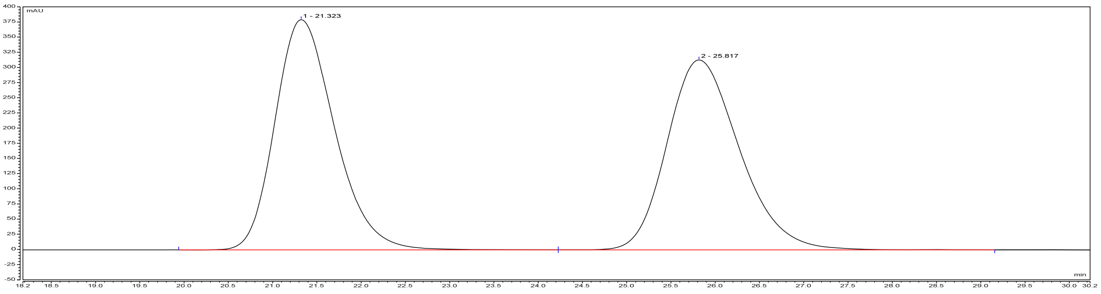

Entry	Retention Time	%Area	Area	Height
1	21.323	49.91	296.7372	379.61
2	25.817	50.09	297.8270	313.23

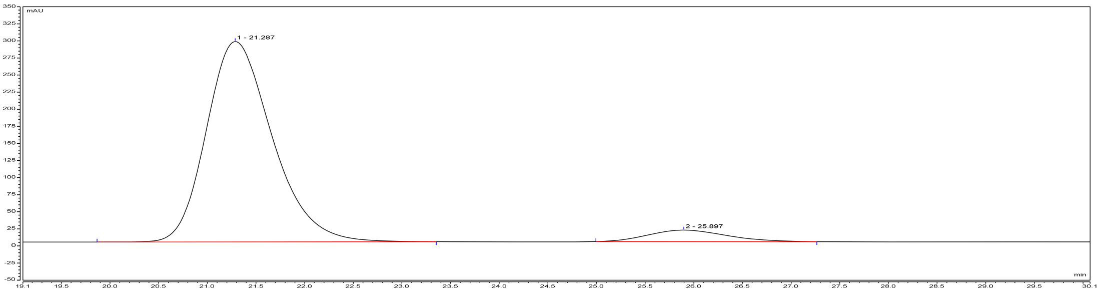

Entry	Retention Time	%Area	Area	Height
1	21.287	93.83	229.9405	293.17
2	25.897	6.17	15.6473	16.84

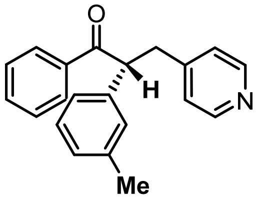

(*R*)-1-phenyl-3-(pyridin-4-yl)-2-(m-tolyl)propan-1-one (3zh): yellow liquid; 19.0 mg, 63% yield; 79% ee; $[\alpha]_D^{25}$ = –83.9 (*c* 1.0, $CHCl_3$); ^{1}H NMR (400 MHz, chloroform-d) δ 8.42 (d, *J* = 5.7 Hz, 2H), 7.93 – 7.87 (m, 2H), 7.50 – 7.43 (m, 1H), 7.38 – 7.34 (m, 2H), 7.16 (t, *J* = 7.8 Hz, 1H), 7.06 – 6.97 (m, 5H), 4.75 (t, *J* = 7.3 Hz, 1H), 3.53 (dd, *J* = 13.7, 7.7 Hz, 1H), 3.05 (dd, *J* = 13.7, 6.9 Hz, 1H), 2.28 (s, 3H); ^{13}C NMR (101 MHz, chloroform-d) δ 198.3, 149.3, 138.8, 138.2, 136.3, 133.1, 129.0, 128.7, 128.6, 128.6, 128.3, 125.3, 124.6, 54.8, 39.4, 21.4. HRMS (ESI) m/z 302.1538 (M+H$^+$), calc. for $C_{21}H_{20}NO$ 302.1540.

The ee was determined by HPLC analysis: CHIRALPAK IG (4.6 mm i.d. x 250 mm); hexane/2–propanol = 75/25; flow rate 1.0 mL/min; 25 ºC; 254 nm; retention time: 8.0 min (major) and 9.6 min (minor).

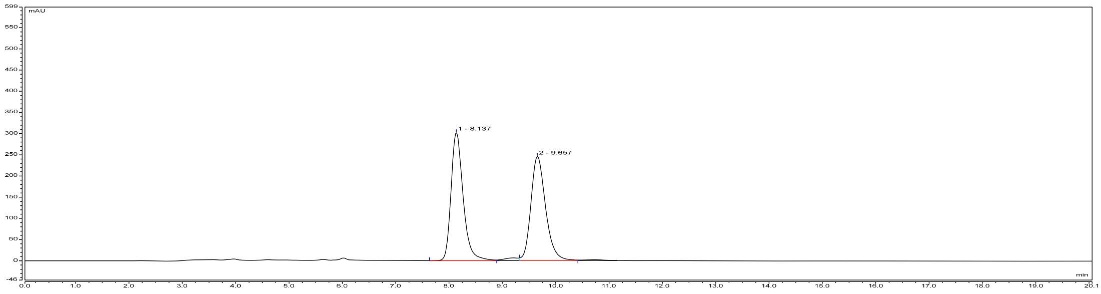

Entry	Retention Time	%Area	Area	Height
1	8.137	50.28	79.2785	301.95
2	9.657	49.72	78.4030	245.55

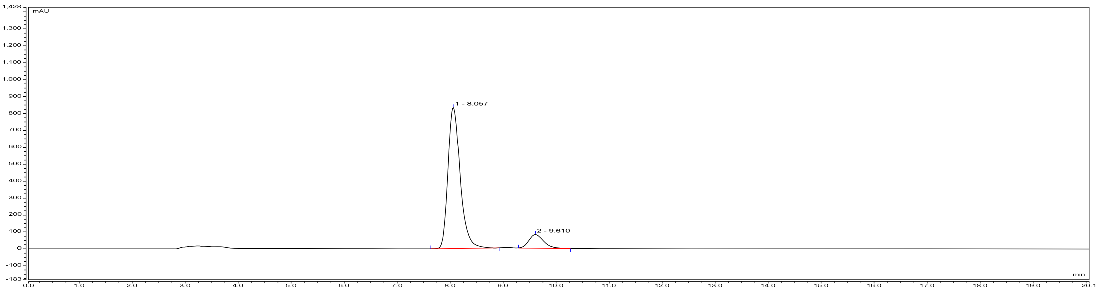

Entry	Retention Time	%Area	Area	Height
1	8.057	89.50	223.0177	832.80
2	9.610	10.50	26.1677	81.09

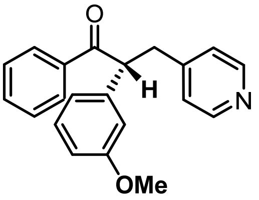

(*R*)-2-(3-methoxyphenyl)-1-phenyl-3-(pyridin-4-yl)propan-1-one (3zi): yellow solid; Mp: 55.6 ºC; 20.0 mg, 63% yield; 80% ee; $[\alpha]_D^{25}$ = –44 (*c* 1.0, $CHCl_3$);^{1}H NMR (400 MHz, chloroform-d) δ 8.42 (d, *J* = 5.7 Hz, 2H), 7.93 – 7.83 (m, 2H), 7.50 – 7.43 (m, 1H), 7.36 (t, *J* = 7.6 Hz, 2H), 7.22 – 7.15 (m, 1H), 7.04 – 7.00 (m, 2H), 6.79 (d, *J* = 7.7 Hz, 1H), 6.76 – 6.71 (m, 2H), 4.75 (t, *J* = 7.3 Hz, 1H), 3.73 (s, 3H), 3.52 (dd, *J* = 13.8, 7.6 Hz, 1H), 3.06 (dd, *J* = 13.8, 7.1 Hz, 1H); ^{13}C NMR (101 MHz, chloroform-d) δ 198.1, 160.0, 149.4, 149.0, 139.7, 136.2, 133.1, 130.1, 128.7, 128.6, 124.5, 120.6, 113.8, 112.7, 55.2, 54.9, 39.2. HRMS (ESI) m/z 318.1487 (M+H$^+$), calc. for $C_{21}H_{20}NO_2$ 318.1489.

The ee was determined by HPLC analysis: CHIRALPAK IG (4.6 mm i.d. x 250 mm); hexane/2–propanol = 75/25; flow rate 1.0 mL/min; 25 ºC; 254 nm; retention time: 16.4 min (major) and 21.1 min (minor).

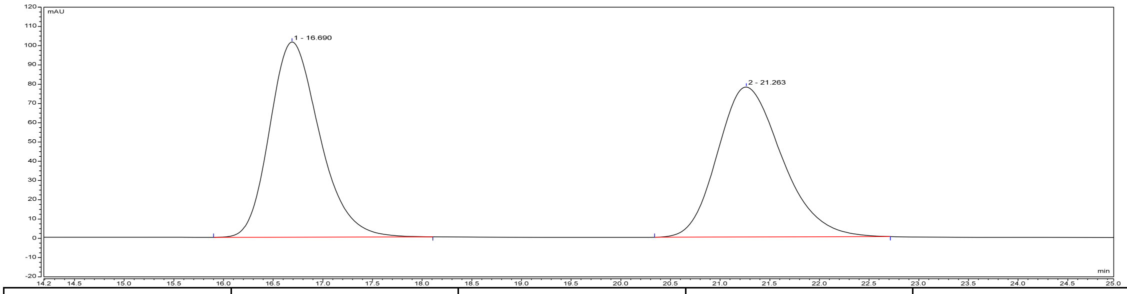

Entry	Retention Time	%Area	Area	Height
1	16.690	50.16	59.0336	101.30
2	21.263	49.84	58.6585	77.89

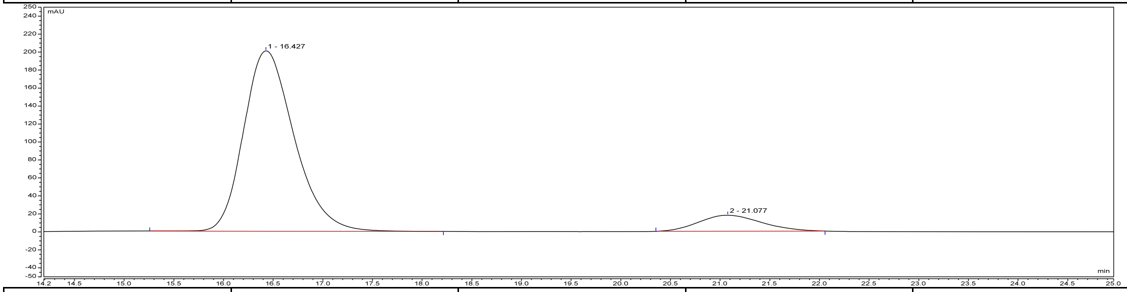

Entry	Retention Time	%Area	Area	Height
1	16.427	90.09	118.4287	200.83
2	21.077	9.91	13.0233	17.78

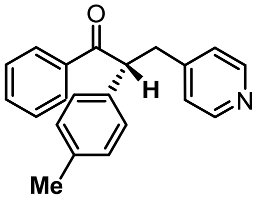

(*R*)-1-phenyl-3-(pyridin-4-yl)-2-(p-tolyl)propan-1-one (3zj): yellow solid; Mp: 103.2 ºC; 18.1 mg, 60% yield; 90% ee; $[\alpha]_D^{25}$ = –74.4 (*c* 1.0, $CHCl_3$); ^{1}H NMR (400 MHz, chloroform-d) δ 8.41 (d, *J* = 5.5 Hz, 2H), 7.92 – 7.86 (m, 2H), 7.49 – 7.43 (m, 1H), 7.35 (t, *J* = 7.7 Hz, 2H), 7.12 – 7.05 (m, 4H), 7.03 – 6.99 (m, 2H), 4.75 (t, *J* = 7.3 Hz, 1H), 3.51 (dd, *J* = 13.7, 7.4 Hz, 1H), 3.05 (dd, *J* = 13.7, 7.2 Hz, 1H), 2.27 (s, 3H); ^{13}C NMR (101 MHz, chloroform-d) δ 198.4, 149.3, 137.2, 136.2, 135.2, 133.0, 129.8, 128.7, 128.5, 128.0, 124.6, 54.5, 39.3, 21.0. HRMS (ESI) m/z 302.1538 (M+H$^+$), calc. for $C_{21}H_{20}NO$ 302.1540.

The ee was determined by HPLC analysis: CHIRALPAK amylose-1 (4.6 mm i.d. x 250 mm); hexane/2–propanol = 93/07; flow rate 1.0 mL/min; 25 ºC; 254 nm; retention time: 28.7 min (major) and 25.6 min (minor).

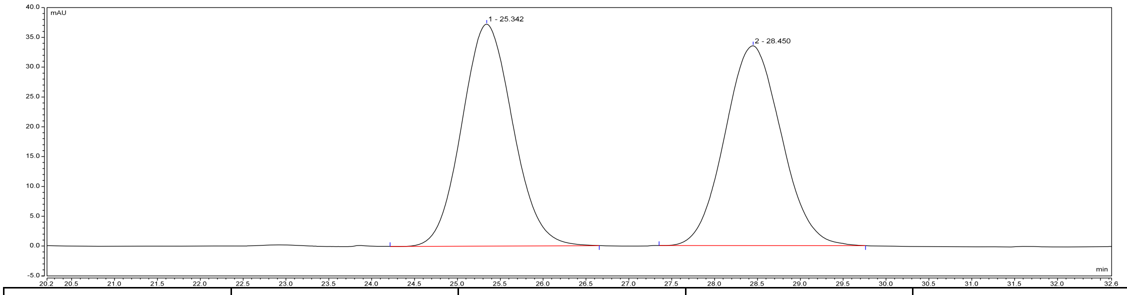

Entry	Retention Time	%Area	Area	Height
1	25.342	50.08	25.7073	37.22
2	28.450	49.92	25.6219	33.50

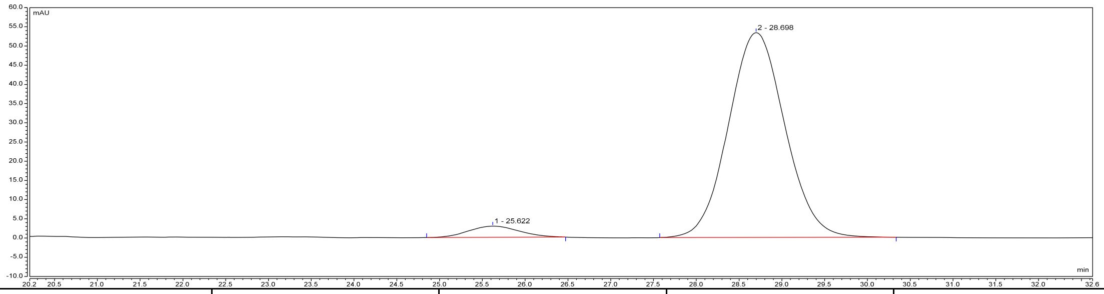

Entry	Retention Time	%Area	Area	Height
1	25.622	4.71	2.0151	2.93
2	28.698	95.29	40.8127	53.34

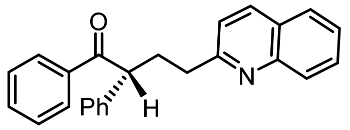

(*R*)-1,2-diphenyl-4-(quinolin-2-yl)butan-1-one (6a): white solid; Mp: 74.8 ºC; 27.0 mg, 77% yield; 90% ee; $[\alpha]_D^{25}$ = 46.6 (*c* 1.0, $CHCl_3$); ^{1}H NMR (300 MHz, chloroform-d) δ 8.06 – 7.93 (m, 4H), 7.77 (d, *J* = 8.1 Hz, 1H), 7.67 (ddd, *J* = 8.5, 6.8, 1.4 Hz, 1H), 7.51 – 7.44 (m, 2H), 7.39 – 7.31 (m, 5H), 7.29 (d, *J* = 3.0 Hz, 2H), 7.23 – 7.18 (m, 1H), 4.74 (t, *J* = 7.2 Hz, 1H), 3.06 – 2.90 (m, 2H), 2.81 – 2.69 (m, 1H), 2.43 – 2.31 (m, 1H); ^{13}C NMR (75 MHz, chloroform-d) δ 199.7, 161.9, 147.8, 139.3, 136.8, 136.2, 132.8, 129.3, 128.9, 128.8, 128.7, 128.4, 128.4, 127.5, 127.1, 126.7, 125.7, 121.5, 52.9, 36.8, 33.6. HRMS (ESI) m/z 352.1694 (M+H$^+$), calc. for $C_{25}H_{22}NO$ 352.1696.

The ee was determined by HPLC analysis: Lux® 5μm Iamylose-1 (4.6 mm i.d. x 250 mm); hexane/2–propanol = 90/10; flow rate 1.0 mL/min; 25 ºC; 254 nm; retention time: 11.7 min (major) and 14.4 min (minor).

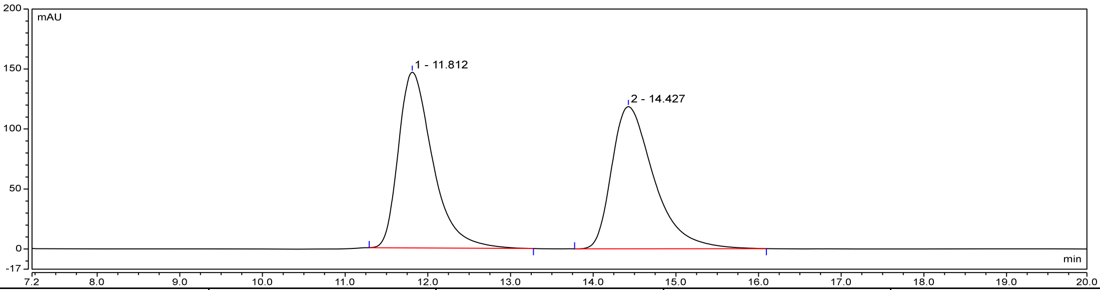

Entry	Retention Time	%Area	Area	Height
1	11.812	50.11	70.5255	146.34
2	14.427	49.89	70.2137	118.47

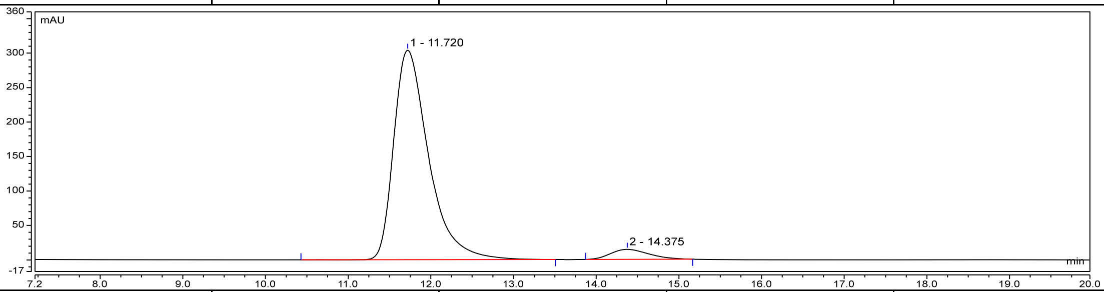

Entry	Retention Time	%Area	Area	Height
1	11.720	95.03	148.5283	303.71
2	14.375	4.97	7.7602	14.44

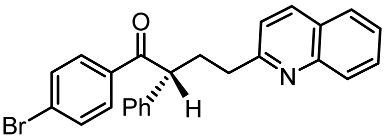

(*R*)-1-(4-bromophenyl)-2-phenyl-4-(quinolin-2-yl)butan-1-one (6b): colorless liquid; 34.0 mg, 79% yield; 91% ee; $[\alpha]_D^{25}$ = 43.8 (*c* 1.0, $CHCl_3$); ^{1}H NMR (300 MHz, chloroform-d) δ 8.05 (d, *J* = 8.5 Hz, 1H), 7.97 (d, *J* = 8.4 Hz, 1H), 7.78 (dd, *J* = 8.9, 2.2 Hz, 3H), 7.68 (ddd, *J* = 8.5, 6.8, 1.5 Hz, 1H), 7.51 – 7.46 (m, 3H), 7.30 – 7.27 (m, 4H), 7.24 – 7.18 (m, 2H), 4.66 (t, *J* = 7.1 Hz, 1H), 3.05 – 2.89 (m, 2H), 2.80 – 2.69 (m, 1H), 2.40 – 2.29 (m, 1H); ^{13}C NMR (75 MHz, chloroform-d) δ 198.7, 161.7, 147.8, 138.9, 136.3, 135.4, 131.7, 130.2, 129.4, 129.0, 128.7, 128.3, 128.0, 127.5, 127.2, 126.7, 125.8, 121.5, 52.8, 36.7, 33.4. HRMS (ESI) m/z 430.0802 (M+H$^+$), calc. for $C_{25}H_{21}BrNO$ 430.0801.

The ee was determined by HPLC analysis: Lux® 5µm IAmylose-1 (4.6 mm i.d. x 250 mm); hexane/2–propanol = 90/10; flow rate 1.0 mL/min; 25 ºC; 254 nm; retention time: 13.3 min (major) and 15.5 min (minor).

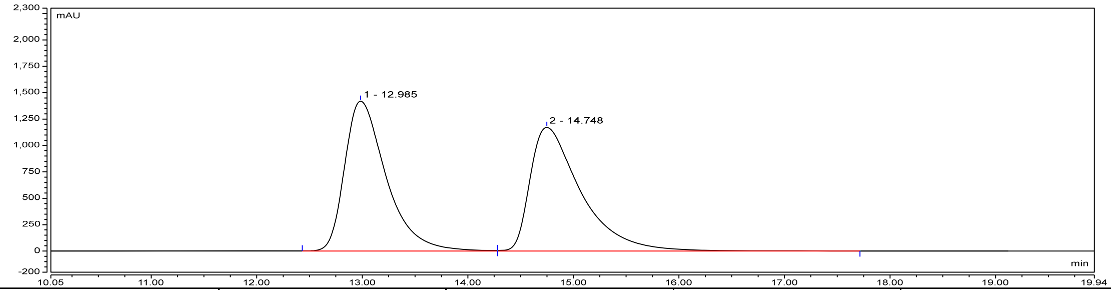

Entry	Retention Time	%Area	Area	Height
1	12.985	49.80	648.4961	1420.26
2	14.748	50.20	653.7585	1171.43

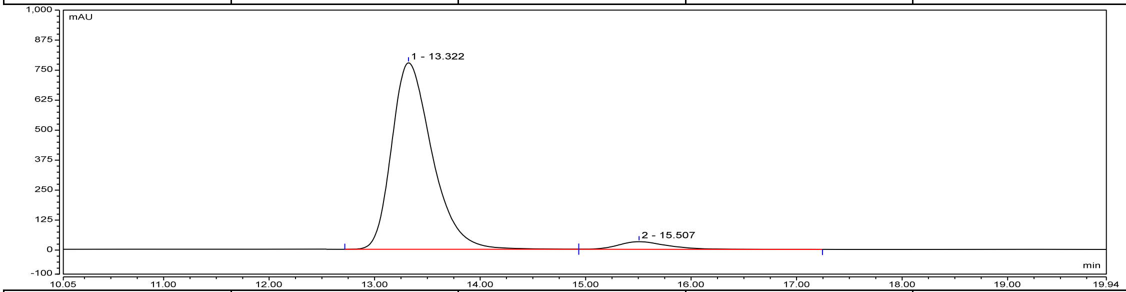

Entry	Retention Time	%Area	Area	Height
1	13.322	95.37	345.0621	776.91
2	15.507	4.63	17.1350	31.85

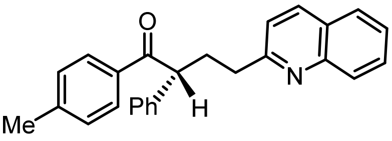

(*R*)-2-phenyl-4-(quinolin-2-yl)-1-(p-tolyl)butan-1-one (6c): white solid; Mp: 78.8 ºC; 27.0 mg, 74% yield; 90% ee; $[\alpha]_D^{25}$ = 65.1 (*c* 1.0, $CHCl_3$);^{1}H NMR (300 MHz, chloroform-d) δ 8.02 (dd, *J* = 13.9, 8.5 Hz, 2H), 7.87 – 7.84 (m, 2H), 7.77 (d, *J* = 8.0 Hz, 1H), 7.67 (ddd, *J* = 8.5, 6.8, 1.5 Hz, 1H), 7.48 (ddd, *J* = 8.1, 6.8, 1.2 Hz, 1H), 7.35 – 7.30 (m, 4H), 7.26 (d, *J* = 2.8 Hz, 1H), 7.18 (dd, *J* = 12.6, 7.4 Hz, 3H), 4.72 (t, *J* = 7.2 Hz, 1H), 3.06 – 2.88 (m, 2H), 2.80 – 2.68 (m, 1H), 2.42 – 2.30 (m, 4H); ^{13}C NMR (75 MHz, chloroform-d) δ 199.3, 162.0 , 147.9, 143.6, 139.6, 136.2, 134.3, 129.3,

129.1, 128.9, 128.3, 127.5, 127.0, 126.8, 125.7, 121.5, 52.8 , 36.9, 33.6, 21.6. HRMS (ESI) m/z 366.1851 (M+H$^+$), calc. for $C_{26}H_{24}NO$ 366.1853.The ee was determined by HPLC analysis: Lux® 5μm IAmylose-1 (4.6 mm i.d. x 250 mm); hexane/2–propanol = 90/10; flow rate 1.0 mL/min; 25 °C; 254 nm; retention time: 12.4 min (major) and 15.9 min (minor).

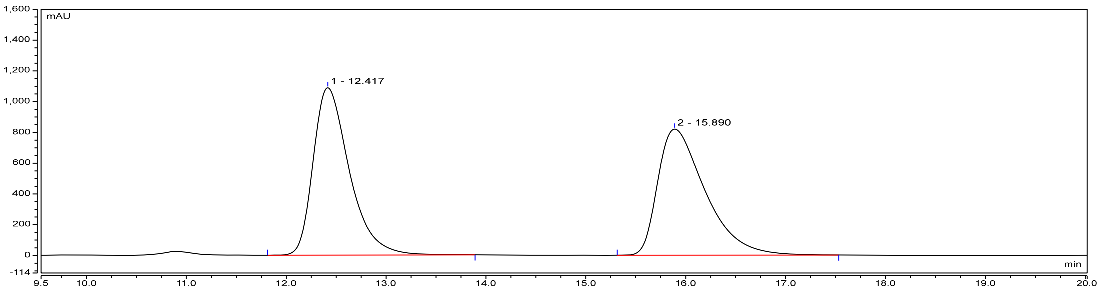

Entry	Retention Time	%Area	Area	Height
1	12.417	50.31	457.3638	1087.57
2	15.890	49.69	451.6507	819.94

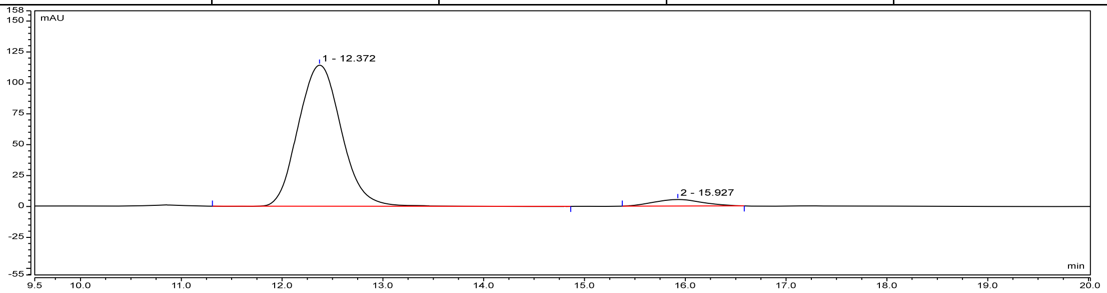

Entry	Retention Time	%Area	Area	Height
1	12.372	95.06	56.4074	114.15
2	15.927	4.94	2.9323	5.26

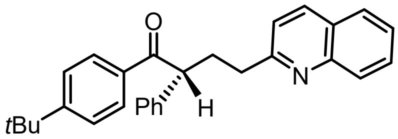

(*R*)-1-(4-(tert-butyl)phenyl)-2-phenyl-4-(quinolin-2-yl) butan-1-one (6d): white solid; Mp: 85.4 °C; 32.6 mg, 80% yield; 93% ee; $[\alpha]_D^{25}$ = 78.6 (*c* 1.0, $CHCl_3$); ^{1}H NMR (300 MHz, chloroform-d) δ 8.03 (dd, *J* = 14.5, 8.5 Hz, 2H), 7.91 (d, *J* = 8.3 Hz, 2H), 7.77 (d, *J* = 8.0 Hz, 1H), 7.67 (ddd, *J* = 8.4, 6.9, 1.5 Hz, 1H), 7.48 (t, *J* = 7.5 Hz, 2H), 7.39 – 7.33 (m, 4H), 7.32 – 7.25 (m, 3H), 7.22 – 7.17 (m, 1H), 4.74 (t, *J* = 7.2 Hz, 1H), 3.07 – 2.90 (m, 2H), 2.79 – 2.67 (m, 1H), 2.42 – 2.30 (m, 1H), 1.28 (s, 9H); ^{13}C NMR (75 MHz, chloroform-d) δ 199.2, 161.9, 156.5, 147.6, 139.5, 136.4, 134.0, 129.4, 128.9, 128.7, 128.3, 127.4, 127.0, 126.7, 125.7, 125.4, 121.5, 52.8, 36.8, 35.0, 33.7, 31.0. HRMS (ESI) m/z 408.2321 (M+H$^+$), calc. for $C_{29}H_{30}NO$ 408.2322.

The ee was determined by HPLC analysis: Lux® 5μm IAmylose-1 (4.6 mm i.d. x 250 mm); hexane/2–propanol = 90/10; flow rate 1.0 mL/min; 25 °C; 254 nm; retention time: 11.1 min (major) and 14.4 min (minor).

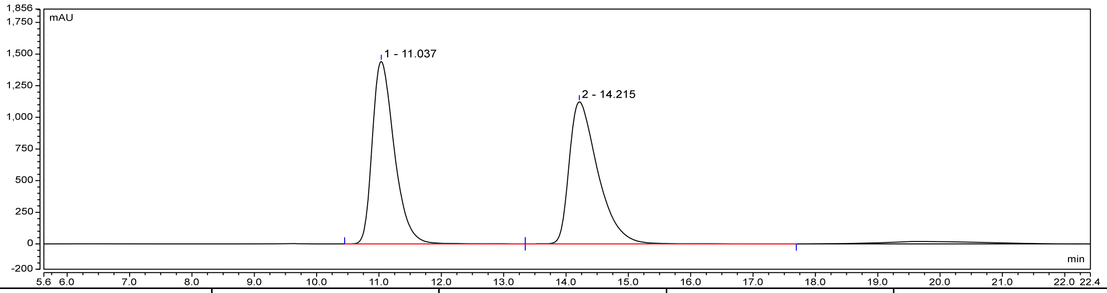

Entry	Retention Time	%Area	Area	Height
1	11.037	49.65	590.8430	1442.01
2	14.215	50.35	599.2447	1122.45

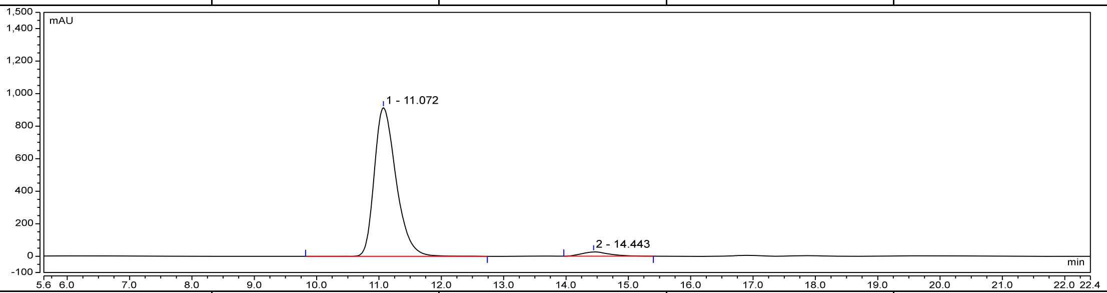

Entry	Retention Time	%Area	Area	Height
1	11.072	96.54	369.2259	913.07
2	14.443	3.46	13.2162	25.99

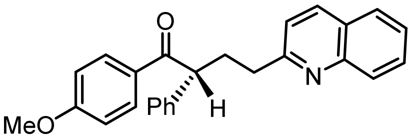

(*R*)-1-(4-methoxyphenyl)-2-phenyl-4-(quinolin-2-yl)butan-1-one (6e): yellow liquid; 28.6 mg, 75% yield; 91% ee; $[\alpha]_D^{25}$ = 166.5 (*c* 1.0, $CHCl_3$); ^{1}H NMR (300 MHz, chloroform-d) δ 8.05 – 7.93 (m, 4H), 7.76 (d, *J* = 8.2 Hz, 1H), 7.67 (ddd, *J* = 8.4, 6.9, 1.5 Hz, 1H), 7.48 (ddd, *J* = 8.1, 6.9, 1.1 Hz, 1H), 7.33 (t, *J* = 6.5 Hz, 3H), 7.28 (d, *J* = 2.7 Hz, 1H), 7.25 (s, 1H), 7.22 – 7.17 (m, 1H), 6.86 – 6.82 (m, 2H), 4.69 (t, *J* = 7.2 Hz, 1H), 3.80 (s, 3H), 3.06 – 2.89 (m, 2H), 2.81 – 2.68 (m, 1H), 2.42 – 2.30 (m, 1H); ^{13}C NMR (75 MHz, chloroform-d) δ 198.2, 163.2, 162.0, 147.8, 139.8, 136.2, 131.0, 129.8, 129.2, 128.8, 128.8, 128.3, 127.4, 127.0, 126.7, 125.7, 121.5, 113.6, 55.3, 52.5, 36.9, 33.6. HRMS (ESI) m/z 382.1798 (M+H$^+$), calc. for $C_{26}H_{24}NO_2$ 382.1802.

The ee was determined by HPLC analysis: Lux® 5μm IAmylose-1 (4.6 mm i.d. x 250 mm); hexane/2–propanol = 90/10; flow rate 1.0 mL/min; 25 ºC; 254 nm; retention time: 21.2 min (major) and 26.7 min (minor).

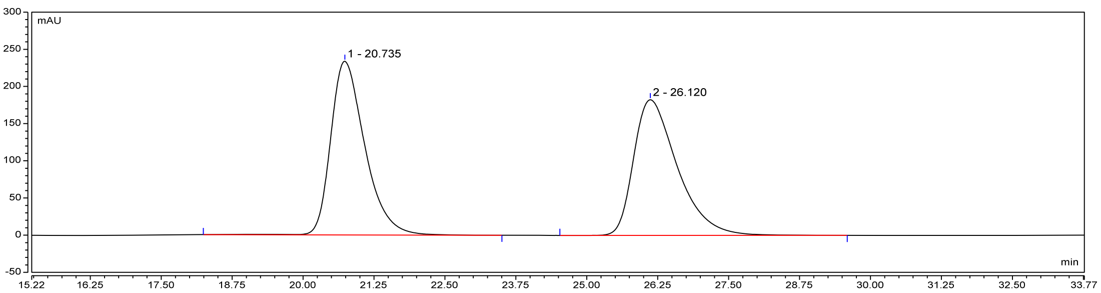

Entry	Retention Time	%Area	Area	Height
1	20.735	50.02	162.8701	233.51
2	26.120	49.98	162.7156	182.40

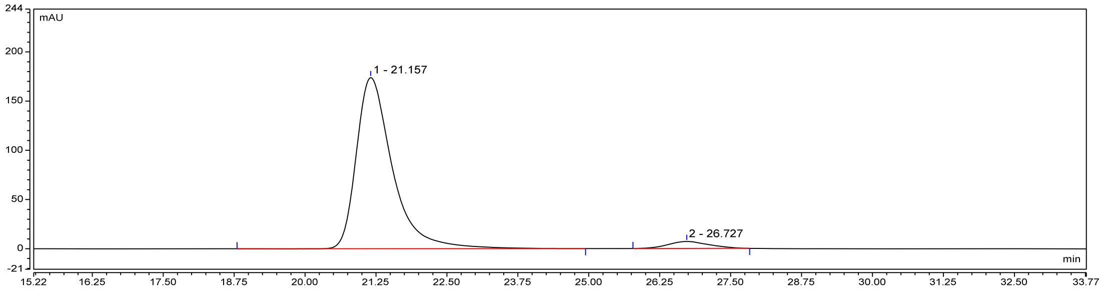

Entry	Retention Time	%Area	Area	Height
1	21.157	95.67	128.3186	173.83
2	26.727	4.33	5.8046	7.00

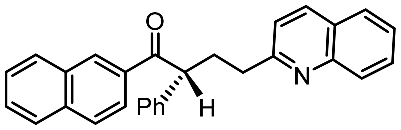

(*R*)-1-(naphthalen-2-yl)-2-phenyl-4-(quinolin-2-yl)butan-1-one (6f): white solid; Mp: 114.2 ºC; 33.3 mg, 83% yield; 90% ee; $[\alpha]_D^{25}$ = 23.0 (*c* 1.0, $CHCl_3$); ^1H NMR (300 MHz, chloroform-d) δ 8.43 (s, 1H), 8.02 (dd, *J* = 17.3, 8.4 Hz, 3H), 7.79 (dt, *J* = 14.1, 7.5 Hz, 4H), 7.65 (ddd, *J* = 8.4, 6.8, 1.4 Hz, 1H), 7.55 – 7.45 (m, 3H), 7.38 (d, *J* = 7.2 Hz, 2H), 7.30 – 7.24 (m, 3H), 7.18 (t, *J* = 7.3 Hz, 1H), 4.90 (t, *J* = 7.2 Hz, 1H), 3.11 – 2.94 (m, 2H), 2.86 – 2.74 (m, 1H), 2.48 – 2.36 (m, 1H); ^{13}C NMR (75 MHz, chloroform-d) δ 199.7, 161.9, 147.6, 139.4, 136.5, 135.3, 134.0, 132.3, 130.4, 129.6, 129.4, 128.9, 128.6, 128.3, 128.3, 127.6, 127.5, 127.1, 126.7, 126.5, 125.8, 124.4, 121.5, 52.8, 36.8, 33.7. HRMS (ESI) m/z 402.1852 ($M+H^+$), calc. for $C_{29}H_{24}NO$ 402.1853.

The ee was determined by HPLC analysis: Lux® 5µm IAmylose-1 (4.6 mm i.d. x 250 mm); hexane/2–propanol = 90/10; flow rate 1.0 mL/min; 25 ºC; 254 nm; retention time: 14.8 min (major) and 17.5 min (minor).

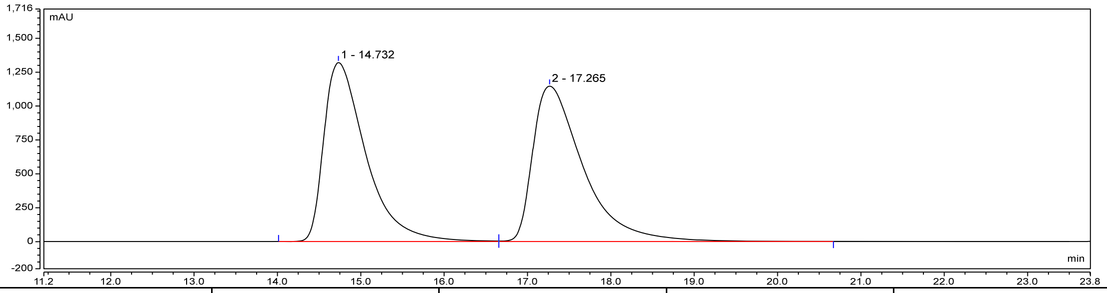

Entry	Retention Time	%Area	Area	Height
1	14.732	49.01	770.4891	1320.42
2	17.265	50.99	801.7392	1147.10

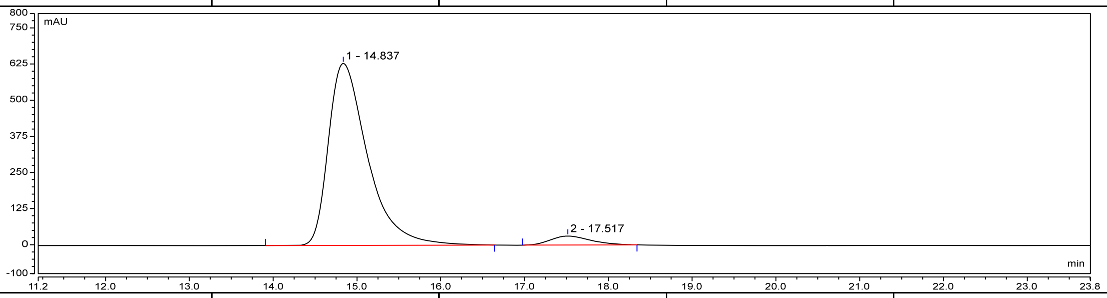

Entry	Retention Time	%Area	Area	Height
1	14.837	95.20	340.7752	629.19
2	17.517	4.80	17.2006	31.05

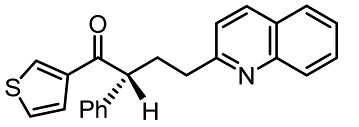

(*R*)-2-phenyl-4-(quinolin-2-yl)-1-(thiophen-3-yl)butan-1-one (6g): white solid; Mp: 89.5 °C; 27.9 mg, 78% yield; 91% ee; $[\alpha]_D^{25}$ = 87.1 (*c* 1.0, $CHCl_3$); ^1H NMR (300 MHz, chloroform-d) δ 8.07 – 7.98 (m, 3H), 7.78 (d, *J* = 8.1 Hz, 1H), 7.68 (ddd, *J* = 8.4, 6.8, 1.5 Hz, 1H), 7.52 – 7.46 (m, 2H), 7.35 – 7.30 (m, 4H), 7.27 (d, *J* = 4.4 Hz, 1H), 7.24 (d, *J* = 2.2 Hz, 1H), 7.21 (dd, *J* = 5.1, 2.8 Hz, 1H), 4.52 (t, *J* = 7.2 Hz, 1H), 3.05 – 2.89 (m, 2H), 2.80 – 2.68 (m, 1H), 2.40 – 2.28 (m, 1H); ^{13}C NMR (75 MHz, chloroform-d) δ 194.0, 161.8, 147.8, 142.0, 139.4, 136.2, 132.7, 129.3, 128.9, 128.8, 128.3, 127.5, 127.4, 127.2, 126.7, 126.0, 125.8, 121.5, 54.7, 36.7, 33.2. HRMS (ESI) m/z 358.1257 ($M+H^+$), calc. for $C_{23}H_{20}NOS$ 358.1261.

The ee was determined by HPLC analysis: Lux® 5µm Iamylose-1 (4.6 mm i.d. x 250 mm); hexane/2–propanol = 90/10; flow rate 1.0 mL/min; 25 °C; 254 nm; retention time: 14.9 min (major) and 17.3 min (minor).

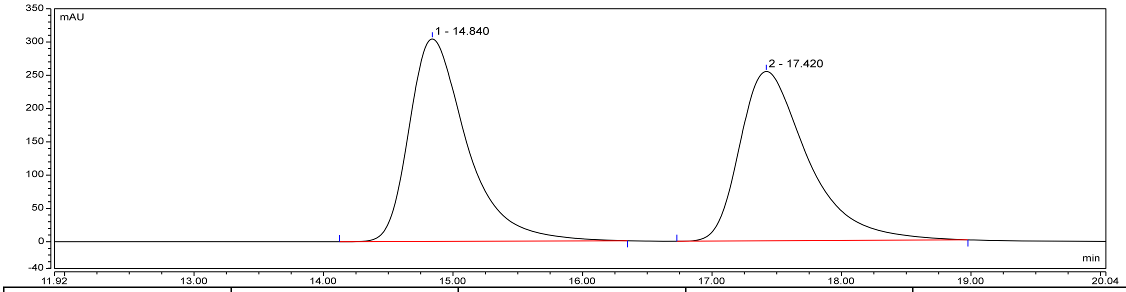

Entry	Retention Time	%Area	Area	Height
1	14.840	50.39	157.1242	304.07
2	17.420	49.61	154.6861	254.42

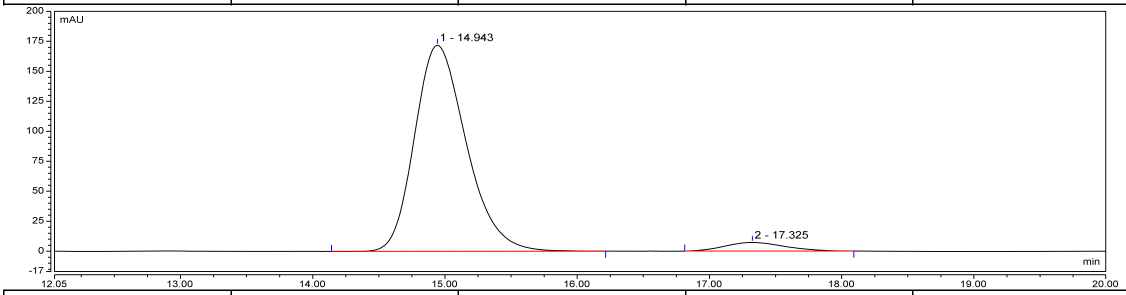

Entry	Retention Time	%Area	Area	Height
1	14.943	95.40	77.7694	171.71
2	17.325	4.60	3.7481	7.25

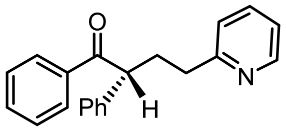

(*R*)-1,2-diphenyl-4-(pyridin-2-yl)butan-1-one (6h): colourless liquid; 18.7 mg, 62% yield; 82% ee; $[\alpha]_D^{25}$ = 38.4 (*c* 1.0, $CHCl_3$); ^1H NMR (300 MHz, chloroform-d) δ 8.51 (dt, *J* = 4.7, 1.5 Hz, 1H), 7.93 (dt, *J* = 7.1, 1.4 Hz, 2H), 7.55 (td, *J* = 7.7, 1.9 Hz, 1H), 7.49 – 7.44 (m, 1H), 7.39 – 7.33 (m, 3H), 7.29 (dd, *J* = 6.7, 1.2 Hz, 3H), 7.22 – 7.16 (m, 1H), 7.11 – 7.06 (m, 2H), 4.63 (t, *J* = 7.2 Hz, 1H), 2.82 – 2.72 (m, 2H), 2.68 – 2.56 (m, 1H), 2.35 – 2.23 (m, 1H); ^{13}C NMR (75 MHz, chloroform-d) δ 199.6, 161.4, 149.2, 139.2, 136.7, 136.3, 132.8, 128.9, 128.6, 128.4, 128.3, 127.0, 122.8, 121.1, 52.9, 36.0, 33.7. HRMS (ESI) m/z 302.1537 ($M+H^+$), calc. for $C_{21}H_{20}NO$ 302.1540.

The ee was determined by HPLC analysis: CHIRALPAK IG (4.6 mm i.d. x 250 mm); hexane/2–propanol = 65/35; flow rate 1.0 mL/min; 25 ºC; 254 nm; retention time: 13.0 min (major) and 17.4 min (minor).

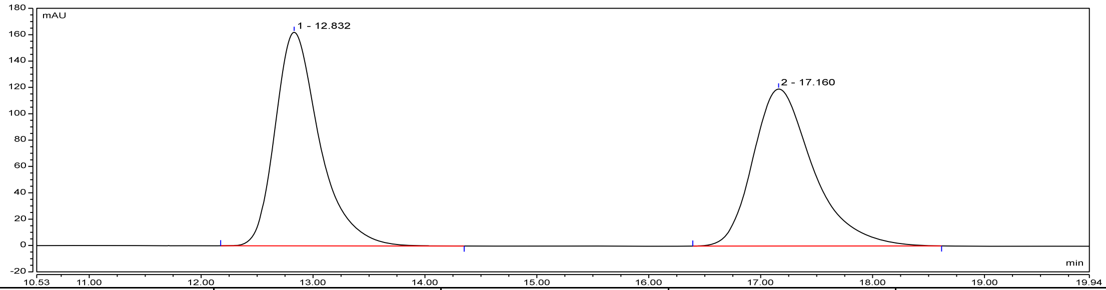

Entry	Retention Time	%Area	Area	Height
1	12.832	50.09	74.5937	162.09
2	17.160	49.91	74.3266	119.20

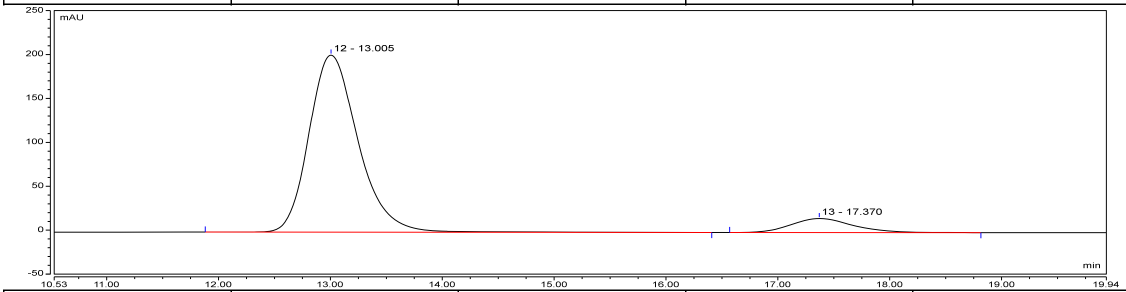

Entry	Retention Time	%Area	Area	Height
1	13.005	90.92	102.6711	201.63
2	17.370	9.08	10.2534	15.97

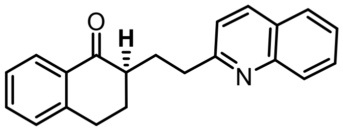

(*R*)-2-(2-(quinolin-2-yl)ethyl)-3,4-dihydronaphthalen-1(2H)-one (6i): white solid; Mp: 58.8 ºC; 18.4 mg, 61% yield; 78% ee; $[\alpha]_D^{25}$ = –14.5 (*c* 1.0, $CHCl_3$); ^{1}H NMR (300 MHz, chloroform-d) δ 8.08 – 8.02 (m, 3H), 7.77 (d, *J* = 8.1 Hz, 1H), 7.67 (ddd, *J* = 8.5, 6.8, 1.5 Hz, 1H), 7.50 – 7.38 (m, 3H), 7.30 (d, *J* = 7.4 Hz, 1H), 7.22 (d, *J* = 7.7 Hz, 1H), 3.13 (t, *J* = 8.0 Hz, 2H), 3.01 (q, *J* = 4.7 Hz, 2H), 2.65 – 2.44 (m, 2H), 2.40 – 2.31 (m, 1H), 2.10 – 1.92 (m, 2H); ^{13}C NMR (75 MHz, chloroform-d) δ 200.0, 162.3, 147.8, 143.9, 136.3, 133.1, 132.5, 129.2, 128.8, 128.6, 127.4, 127.4, 126.7, 126.5, 125.7, 121.4, 47.1, 36.5, 29.6, 28.5. HRMS (ESI) m/z 302.1537 ($M+H^+$), calc. for $C_{21}H_{20}NO$ 302.1540.

The ee was determined by HPLC analysis: CHIRALPAK IG (4.6 mm i.d. x 250 mm) and CHIRALPAK IG (4.6 mm i.d. x 250 mm); Hexane/2-propanol = 75/25; flow rate 1.0 mL/min; 25 ºC; 254 nm; retention time: 35.4 min (major) and 33.3 min (minor).

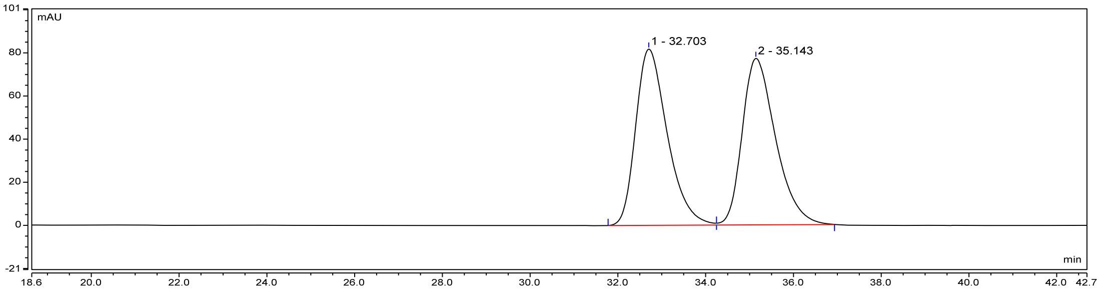

Entry	Retention Time	%Area	Area	Height
1	32.703	50.01	68.1842	81.72
2	35.143	49.99	68.1604	77.21

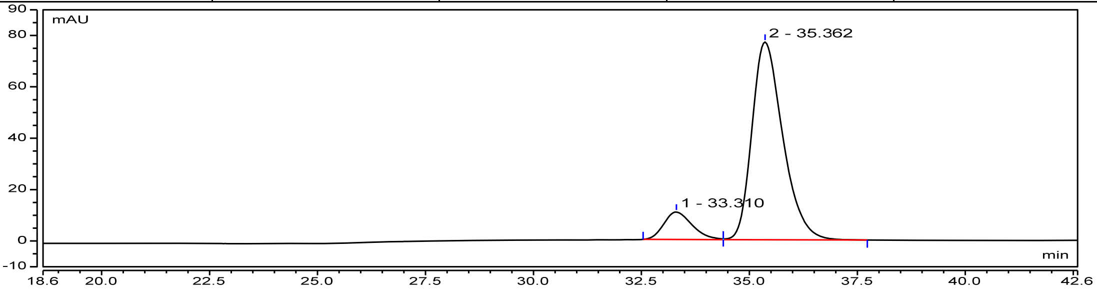

Entry	Retention Time	%Area	Area	Height
1	33.310	11.29	8.1268	10.72
2	35.362	88.71	63.8528	76.93

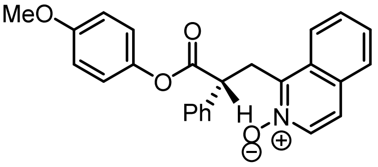

(*R*)-1-(3-(4-methoxyphenoxy)-3-oxo-2-phenylpropyl)isoquinoline 2-oxide (7): yellow solid; Mp: 112.2 ºC; 29.6 mg, 74% yield; 93% ee; $[\alpha]_D^{25}$ = 15.8 (*c* 1.0, $CHCl_3$); ^{1}H NMR (300 MHz, chloroform-*d*) δ 8.24 (d, *J* = 7.1 Hz, 1H), 7.97 – 7.88 (m, 1H), 7.76 – 7.68 (m, 1H), 7.58 (d, *J* = 7.1 Hz, 1H), 7.50 (dd, *J* = 6.4, 3.2 Hz, 2H), 7.43 (d, *J* = 7.4 Hz, 2H), 7.33 – 7.26 (m, 2H), 7.25 – 7.21 (m, 2H), 6.79 – 6.72 (m, 4H), 4.95 (t, *J* = 7.3 Hz, 1H), 4.16 (dd, *J* = 13.4, 7.9 Hz, 1H), 3.74 (s, 4H); ^{13}C NMR (75 MHz, chloroform-*d*) δ 172.5, 157.1, 144.1, 138.2, 136.5, 129.2, 129.0, 128.8, 128.3, 127.8, 127.6, 127.0, 124.4, 122.8, 122.0, 114.2, 55.5, 46.2, 31.4. HRMS (ESI) m/z 400.1545 ($M+H^+$), calc. for $C_{25}H_{22}NO_4$ 400.1549.

The ee was determined by HPLC analysis: Chiral INA (4.6 mm i.d. x 250 mm); hexane/2–propanol = 60/40; flow rate 1.0 mL/min; 25 ºC; 254 nm; retention time: 14.12 min (major) and 17.4 min (minor).

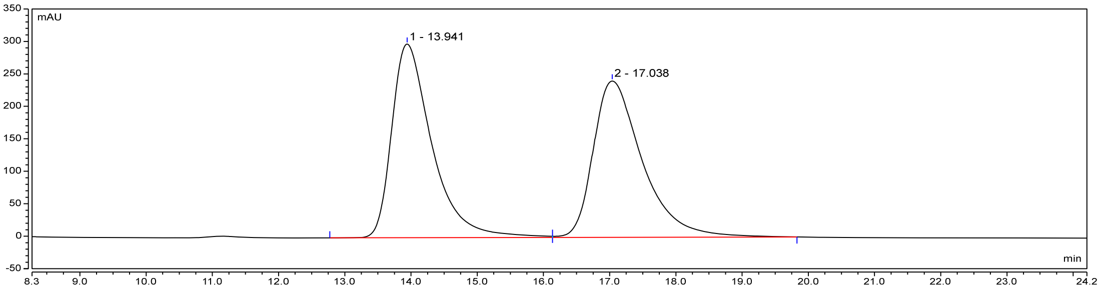

Entry	Retention Time	%Area	Area	Height
1	13.941	50.36	209.5712	298.10
2	17.038	49.64	206.5369	240.81

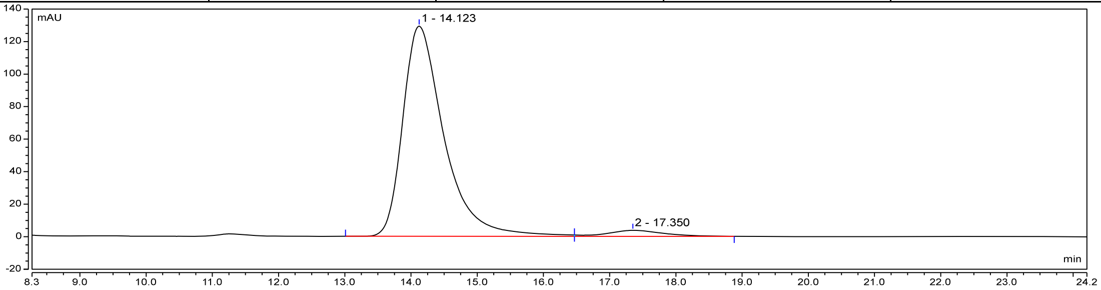

Entry	Retention Time	%Area	Area	Height
1	14.123	96.36	93.0839	129.24
2	17.350	3.64	3.5186	3.67

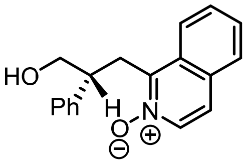

(*R*)-1-(3-hydroxy-2-phenylpropyl)isoquinoline 2-oxide (8): yellow liquid; 26.8 mg, 96% yield; 93% ee; $[\alpha]_D^{25}$ = 16.8 (*c* 1.0, $CHCl_3$); ^{1}H NMR (300 MHz, chloroform-*d*) δ 8.25 (d, *J* = 7.1 Hz, 1H), 7.91 – 7.76 (m, 2H), 7.66 – 7.60 (m, 3H), 7.43 – 7.34 (m, 4H), 7.30 (d, *J* = 7.0 Hz, 1H), 4.27 (dd, *J* = 13.6, 10.3 Hz, 1H), 3.79 (dd, *J* = 12.0, 6.4 Hz, 1H), 3.61 – 3.52 (m, 2H), 3.21 – 3.15 (m, 1H); ^{13}C NMR (75 MHz, chloroform-*d*) δ 148.4, 143.0, 136.1, 129.8, 129.5, 129.2, 128.7, 128.5, 127.7, 127.5, 127.0, 124.2, 122.6, 66.1, 48.6, 29.8. HRMS (ESI) m/z 280.1333 (M+H$^+$), calc. for $C_{18}H_{18}NO_2$ 280.1337.

The ee was determined by HPLC analysis: Chiral INB (4.6 mm i.d. x 250 mm); hexane/2–propanol = 60/40; flow rate 1.0 mL/min; 25 °C; 254 nm; retention time: 10.52 min (major) and 9.44 min (minor).

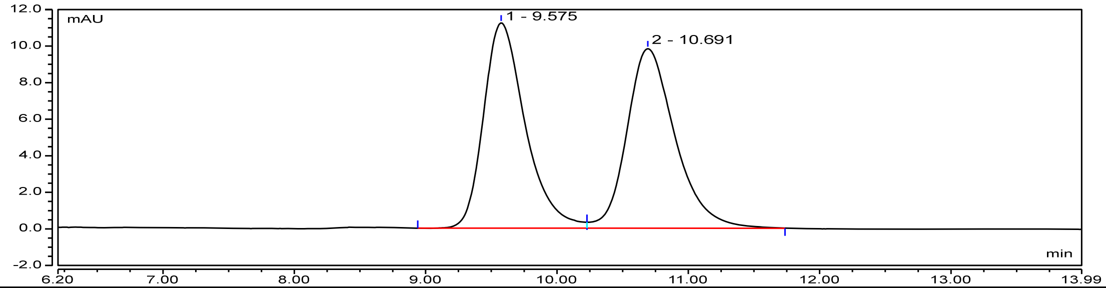

Entry	Retention Time	%Area	Area	Height
1	9.575	49.73	4.1403	11.23
2	10.691	50.27	4.1850	9.82

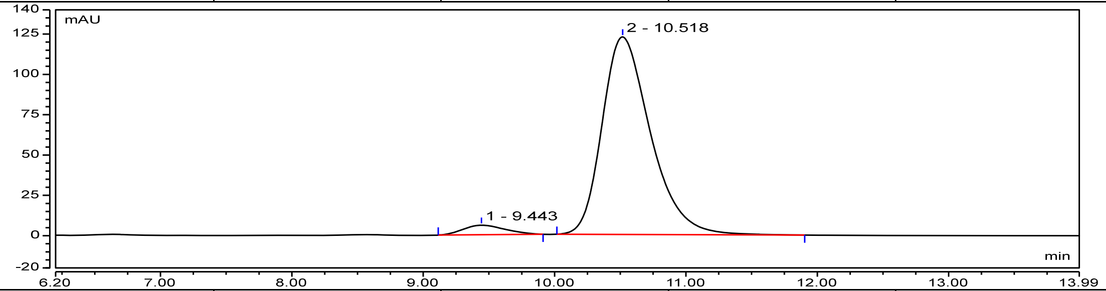

Entry	Retention Time	%Area	Area	Height
1	9.443	3.67	1.9911	5.69
2	10.518	96.33	52.3343	122.41

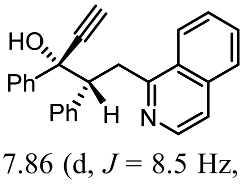

(3*S*,4*R*)-5-(isoquinolin-1-yl)-3,4-diphenylpent-1-yn-3-ol (9): yellow solid; Mp: 163.2 °C; 29.1 mg, 80% yield; 92% ee; $[\alpha]_D^{25}$ = 18.8 (*c* 1.0, $CHCl_3$); ^{1}H NMR (300 MHz, chloroform-*d*) δ 8.29 (d, *J* = 5.8 Hz, 1H), 7.86 (d, *J* = 8.5 Hz, 1H), 7.73 (d, *J* = 8.2 Hz, 1H), 7.63 – 7.28 (m, 3H), 7.48 (d, *J* = 7.9 Hz, 1H), 7.41 (d, *J* = 5.8 Hz, 1H), 7.33 – 7.28 (m, 4H), 7.26 – 7.21 (m, 2H), 7.14 – 7.11 (m, 3H), 5.40 (s, 1H), 4.09 – 4.02 (m, 1H), 4.00 – 3.92 (m, 1H), 3.88 –3.82 (m, 1H), 2.72 (s, 1H); ^{13}C NMR (75 MHz, chloroform-*d*) δ 159.7, 142.2, 140.8, 139.0, 136.0, 130.2, 129.8, 127.6, 127.5,

127.4, 127.2, 127.1, 127.0, 126.8, 126.8, 125.0, 119.4, 86.3, 75.6, 75.3, 57.0, 36.0. HRMS (ESI) m/z 364.1696 (M+H$^+$), calc. for $C_{26}H_{22}NO$ 364.1696.

The ee was determined by HPLC analysis: CHIRALPAK IG (4.6 mm i.d. x 250 mm); hexane/2–propanol = 80/20; flow rate 1.0 mL/min; 25 °C; 254 nm; retention time: 18.12 min (major) and 13.56 min (minor).

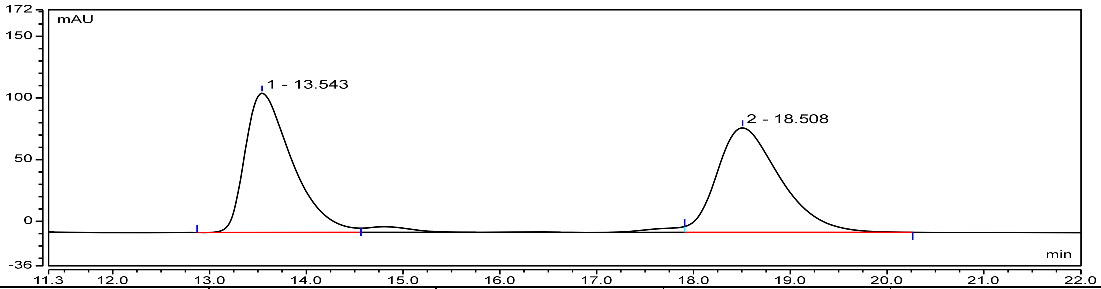

Entry	Retention Time	%Area	Area	Height
1	13.543	49.80	63.6558	112.92
2	18.508	50.20	64.1579	84.72

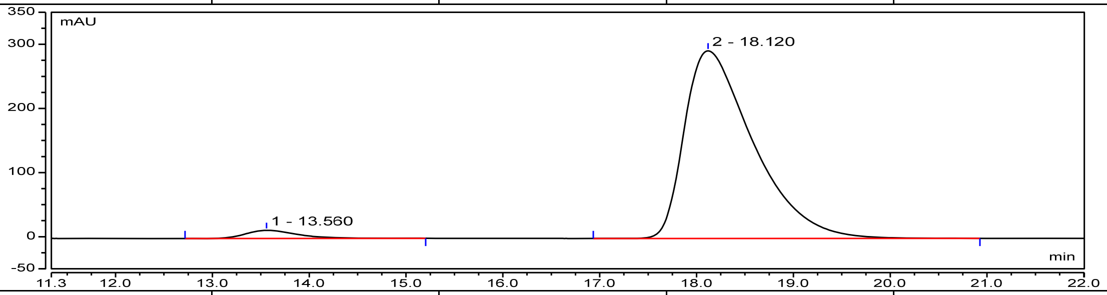

Entry	Retention Time	%Area	Area	Height
1	13.560	3.79	7.9249	12.68
2	18.120	96.21	240.5474	292.42

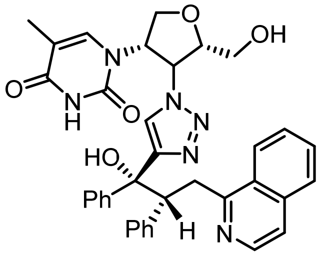

1-((3*S*,5*S*)-4-(4-((1*R*,2*R*)-1-hydroxy-3-(isoquinolin-1-yl)-1,2-diphenylpropyl)-1H-1,2,3-triazol-1-yl)-5-(hydroxymethyl)tetrahydrofuran-3-yl)-5-methylpyrimidine-2,4(1H,3H)-dione (10): white solid; Mp: 59.5 °C; 46.1 mg, 73% yield; 92% ee; $[\alpha]_D^{25}$ = –72.9 (*c* 1.0, $CHCl_3$); ^{1}H NMR (300 MHz, chloroform-*d*) δ 9.60 (s, 1H), 8.26 (d, *J* = 5.8 Hz, 1H), 8.09 (d, *J* = 8.3 Hz, 1H), 7.77 (s, 1H), 7.67 (d, *J* = 8.1 Hz, 1H), 7.59 (t, *J* = 7.4 Hz, 1H), 7.54 – 7.39 (m, 5H), 7.15 – 7.13 (m, 2H), 7.07 – 7.02 (m, 2H), 6.96 – 6.86 (m, 5H), 6.19 (t, *J* = 6.5 Hz, 1H), 5.26 (t, *J* = 6.5 Hz, 1H), 4.74 (t, *J* = 6.8 Hz, 1H), 4.23 (dd, *J* = 5.4, 2.7 Hz, 1H), 3.93 – 3.89 (m, 3H), 3.68 (d, *J* = 11.6 Hz, 1H), 2.81 (t, *J* = 6.9 Hz, 2H), 1.86 (s, 3H); ^{13}C NMR (75 MHz, chloroform-*d*) δ 163.8, 160.6, 155.3, 150.5, 144.7, 140.3, 140.0, 137.5, 136.0, 130.4, 129.8, 127.6, 127.4, 127.4, 127.0, 126.3, 126.1, 125.9, 125.7, 121.6, 119.8, 111.2, 88.1, 85.1, 77.6, 77.2, 61.5, 59.1, 55.1, 37.2, 37.2, 12.4. HRMS (ESI) m/z 631.2664 (M+H$^+$), calc. for $C_{36}H_{35}N_6O_5$ 631.2664.

The ee was determined by HPLC analysis: CHIRALPAK IG (4.6 mm i.d. x 250 mm); hexane/2–propanol = 60/40; flow rate 1.0 mL/min; 25 °C; 230 nm; retention time: 33.92 min (major) and 25.4 min (minor).

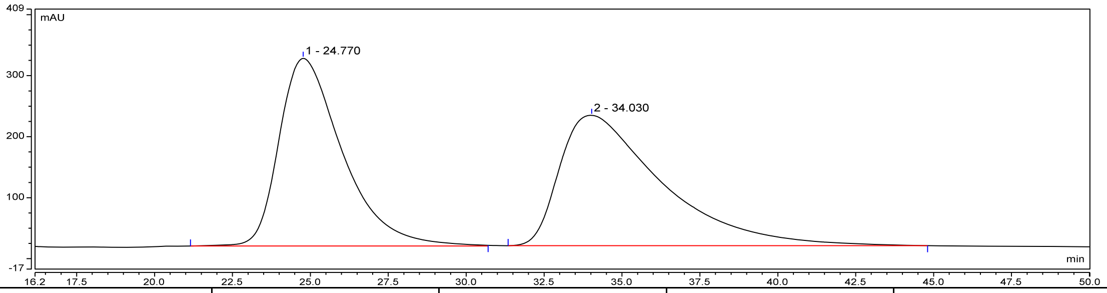

Entry	Retention Time	%Area	Area	Height
1	24.770	48.76	726.8314	307.46
2	34.030	51.24	763.6526	207.51

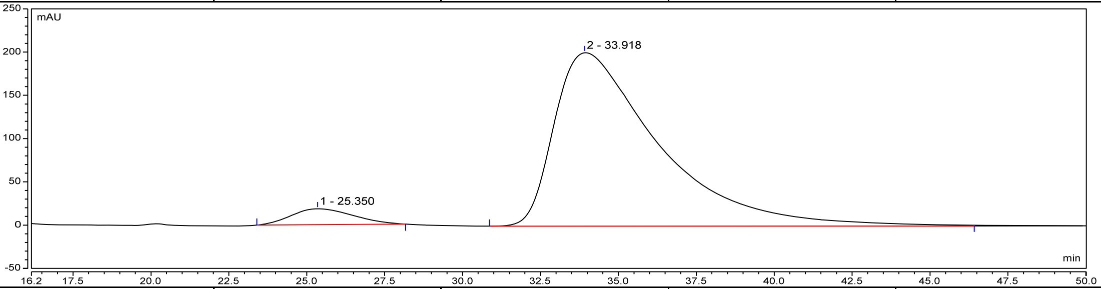

Entry	Retention Time	%Area	Area	Height
1	25.350	4.07	41.5999	18.24
2	33.918	95.93	778.4767	200.48

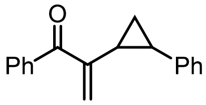

1-phenyl-2-(2-phenylcyclopropyl)prop-2-en-1-one (12): ^{1}H NMR (300 MHz, chloroform-*d*) δ 7.81 (d, *J* = 7.7 Hz, 2H), 7.54 (d, *J* = 7.7 Hz, 1H), 7.44 (t, *J* = 7.7 Hz, 2H), 7.31 – 7.26 (m, 2H), 7.21 – 7.15 (m, 3H), 5.64 (s, 1H), 5.52 (s, 1H), 2.20 – 2.14 (m, 2H), 1.37 – 1.32 (m, 2H); ^{13}C NMR (75 MHz, chloroform-*d*) δ 197.7, 148.6, 141.8, 137.4, 132.4, 129.5, 128.3, 128.2, 126.1, 125.9, 120.6, 26.6, 24.1, 16.9.

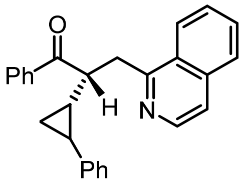

(2*R*)-3-(isoquinolin-1-yl)-1-phenyl-2-((1*R*)-2-phenylcyclopropyl)propan-1-one (13): yellow liquid; 23.8 mg, 63% yield; dr 1:1; 54% ee; 50% ee; $[\alpha]_D^{25}$ = 24.2 (*c* 1.0, $CHCl_3$); ^{1}H NMR (300 MHz, chloroform-*d*) δ 8.38 – 8.18 (m, 2H), 8.14 – 7.96 (m, 2H), 7.82 – 7.71 (m, 1H), 7.70 – 7.39 (m, 6H), 7.25 – 7.02 (m, 3H), 6.83 (ddd, *J* = 13.7, 6.8, 1.8 Hz, 2H), 4.20 – 4.07 (m, 1H), 4.03 – 3.94 (m, 1H), 3.74 – 3.65 (m, 1H), 1.88 – 1.81 (m, 0.5H), 1.74 – 1.67 (m, 0.5H), 1.51 – 1.42 (m, 0.5H), 1.40 – 1.31 (m, 0.5H), 1.04 – 0.77 (m, 2H); ^{13}C NMR (75 MHz, chloroform-*d*) δ 203.3, 203.1, 158.9, 142.5, 142.0, 141.3, 141.2, 138.6, 138.2, 135.9, 135.9, 132.6, 132.4, 129.8, 128.7, 128.4, 128.4, 128.2, 128.1, 127.2, 127.2, 127.2, 127.1, 127.0, 126.1, 125.5, 125.4, 125.4, 125.1, 119.3, 119.2, 49.2, 48.8, 36.9, 36.4, 26.0, 25.6, 22.9, 22.8, 15.6, 14.1. HRMS (ESI) m/z 378.1845 (M+H$^+$), calc. for $C_{27}H_{24}NO$ 378.1852.

The ee was determined by HPLC analysis: CHIRALPAK IF (4.6 mm i.d. x 250 mm); hexane/2–propanol = 92/08; flow rate 1.0 mL/min; 25 °C; 254 nm; retention time: major diastereomer: 13.4 min (major) and 12.0 min (minor); minor diastereomer: 23.0 min (major) and 18.7 min (minor).

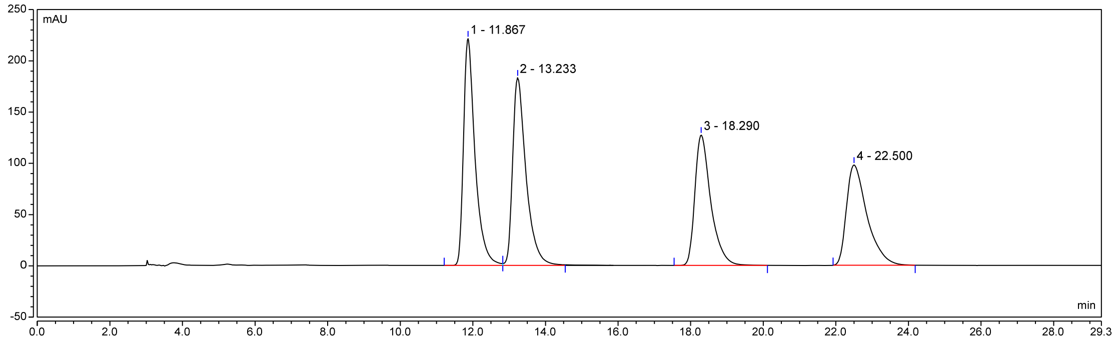

Entry	Retention Time	%Area	Area	Height
1	11.867	28.28	81.6349	221.13
2	13.233	27.13	78.3234	183.07
3	18.290	22.36	64.5600	127.30
4	22.500	22.23	64.1844	97.83

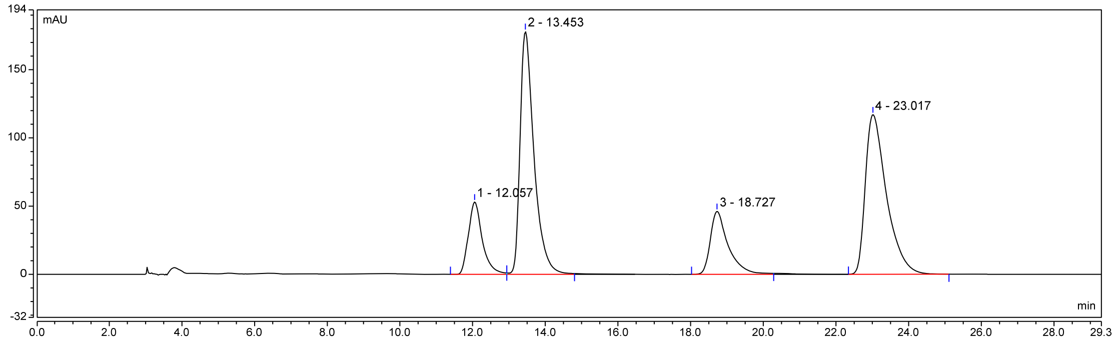

Entry	Retention Time	%Area	Area	Height
1	12.057	11.37	23.6280	53.10
2	13.453	38.02	79.0225	178.11
3	18.727	12.71	26.4253	46.24
4	23.017	37.90	78.7676	117.03

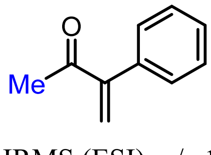

3-phenylbut-3-en-2-one: ^{1}H NMR (300 MHz, Chloroform-*d*) δ 7.52 – 7.29 (m, 5H), 6.19 (s, 1H), 5.98 (s, 1H), 2.45 (s, 3H); ^{13}C NMR (75 MHz, Chloroform-*d*) δ 199.4, 149.4, 136.9, 128.4, 128.1, 128.1, 125.9, 27.4. HRMS (ESI) m/z 147.0802 (M+H^{+}), calc. for $C_{10}H_{11}O$ 147.0804.

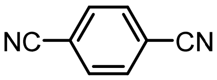

1,4-dicyanobenzene: ^{1}H NMR (300 MHz, Chloroform-*d*) δ 7.79 (s, 4H); ^{13}C NMR (75 MHz, Chloroform-*d*) δ 132.7, 117.0, 116.7.

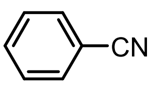

benzonitrile: ^{1}H NMR (300 MHz, Chloroform-*d*) δ 7.70 – 7.55 (m, 3H), 7.47 (t, *J* = 7.6 Hz, 2H); ^{13}C NMR (75 MHz, Chloroform-*d*) δ 132.7, 132.1, 129.0, 118.8, 112.4.

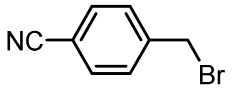

4-cyanobenzyl bromide: ^{1}H NMR (300 MHz, Chloroform-*d*) δ 7.62 (d, *J* = 8.1 Hz, 2H), 7.49 (d, *J* = 8.4 Hz, 2H), 4.47 (s, 2H); ^{13}C NMR (75 MHz, Chloroform-*d*) δ 142.7, 132.5, 129.6, 118.2, 112.1, 31.4.

8. Copies of NMR spectra

^{1}H NMR (300 MHz, Chloroform-d) of compound **3a**

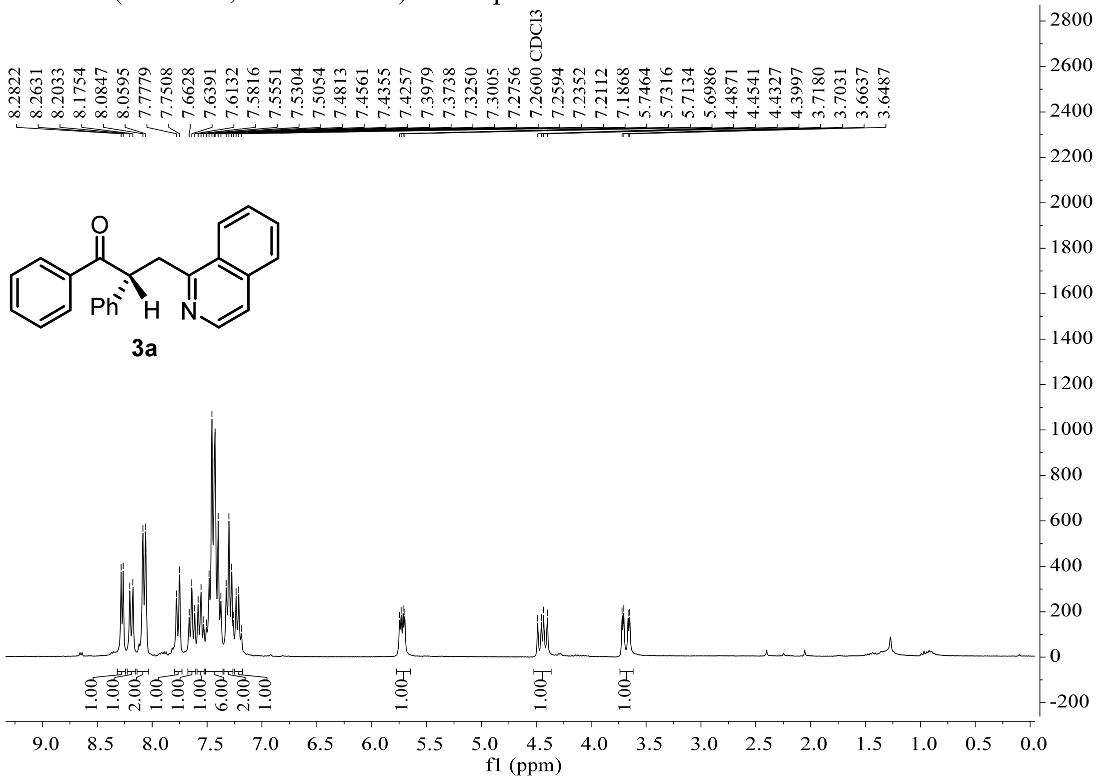

^{13}C NMR (75 MHz, Chloroform-d) of compound **3a**

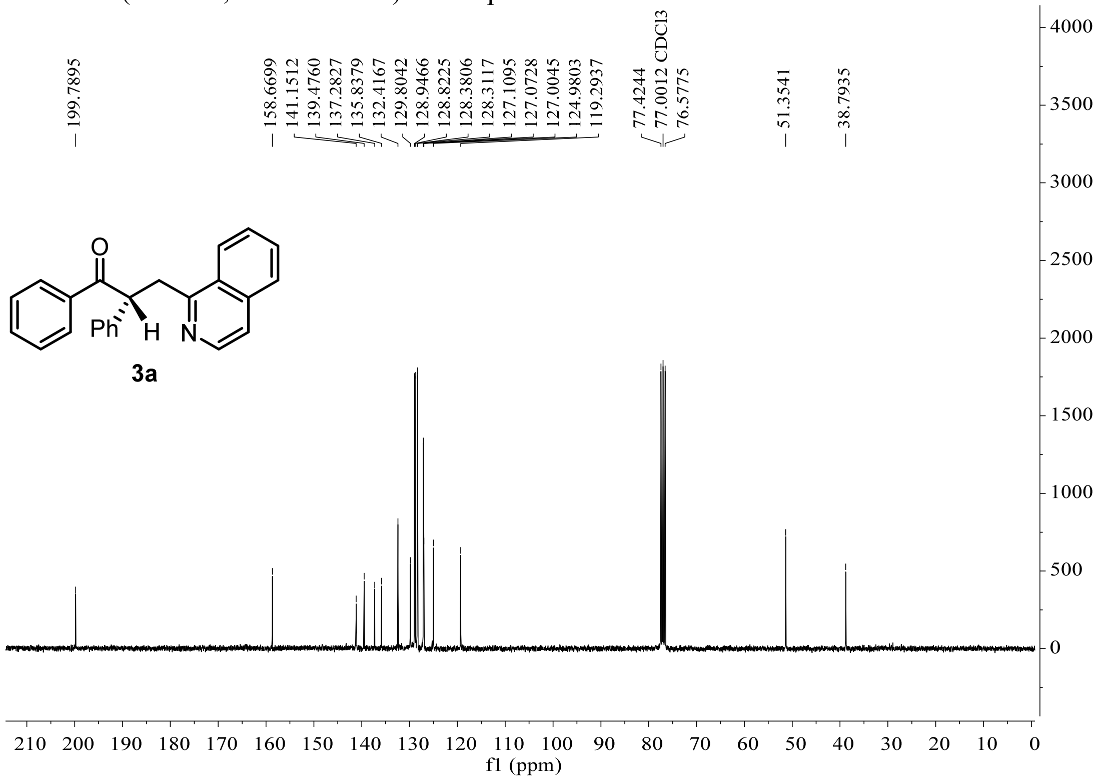

^{1}H NMR (300 MHz, Chloroform-d) of compound **3b**

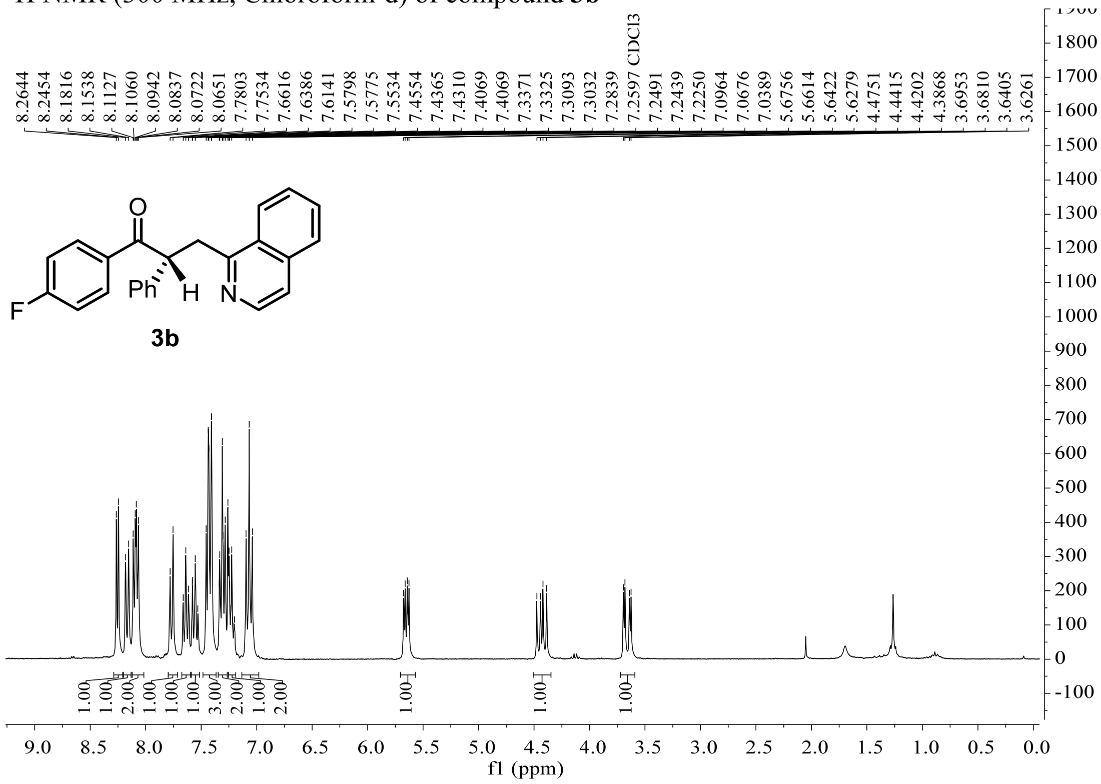

^{13}C NMR (75 MHz, Chloroform-*d*) of compound **3b**

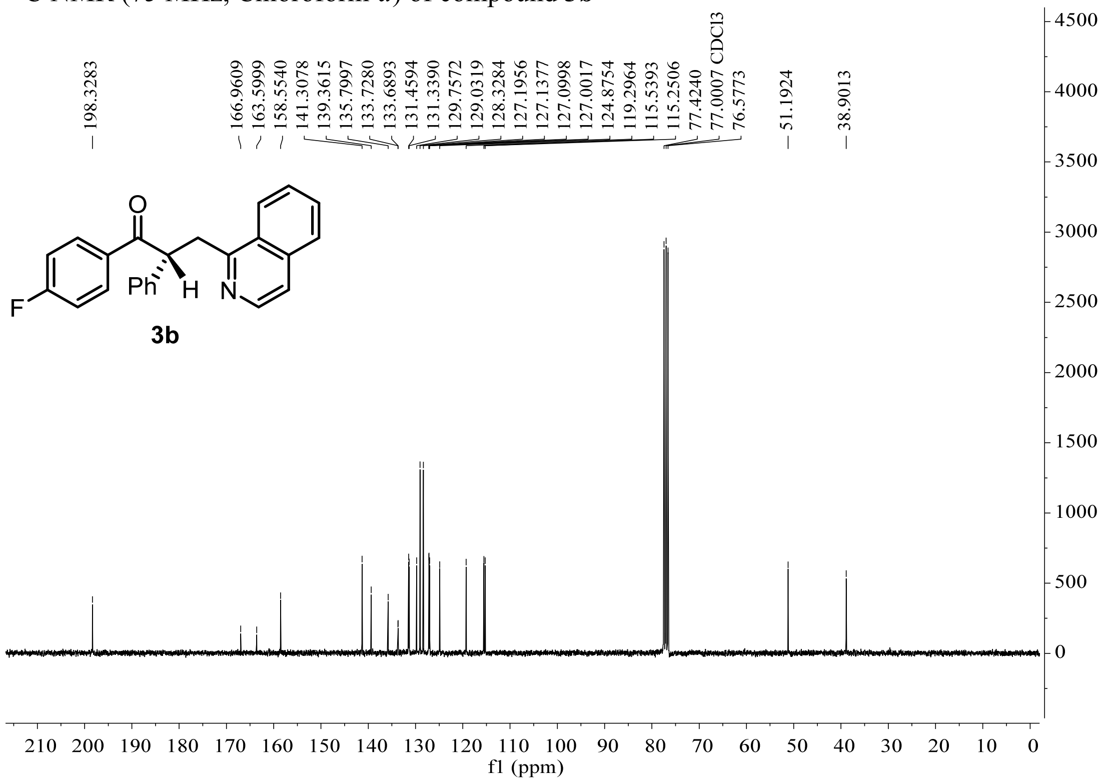

^{19}F NMR (376 MHz, Chloroform-d) of compound **3b**

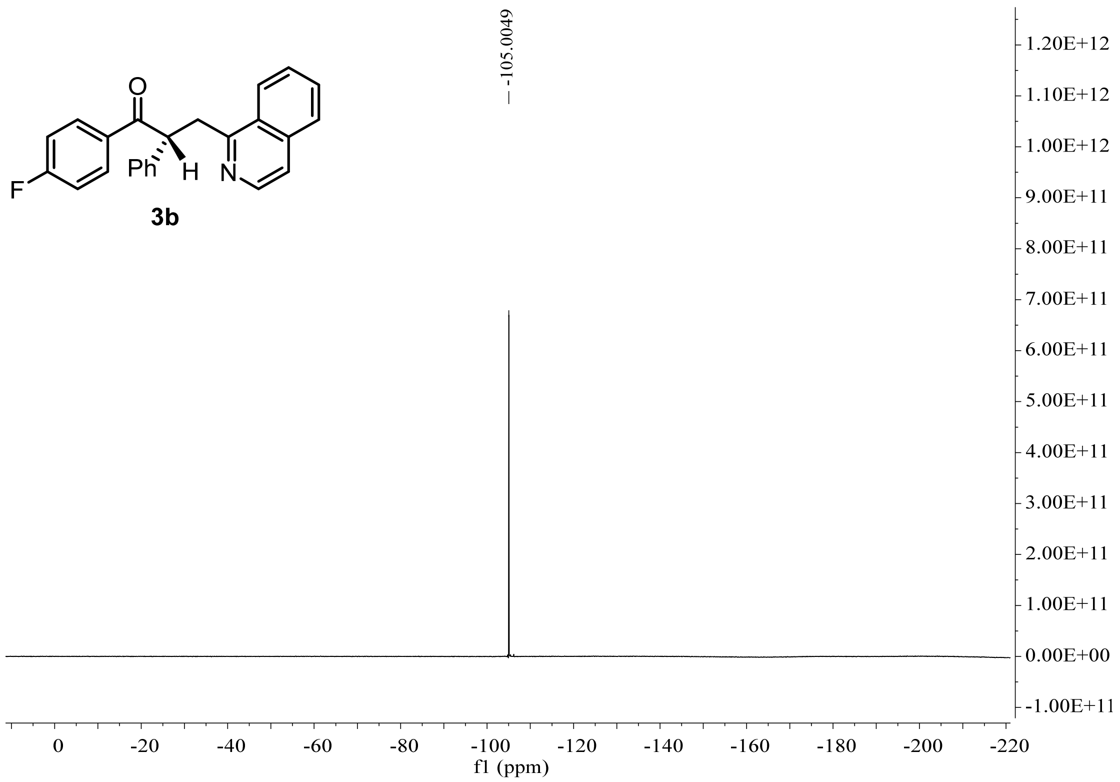

^{1}H NMR (600 MHz, Chloroform-d) of compound **3c**

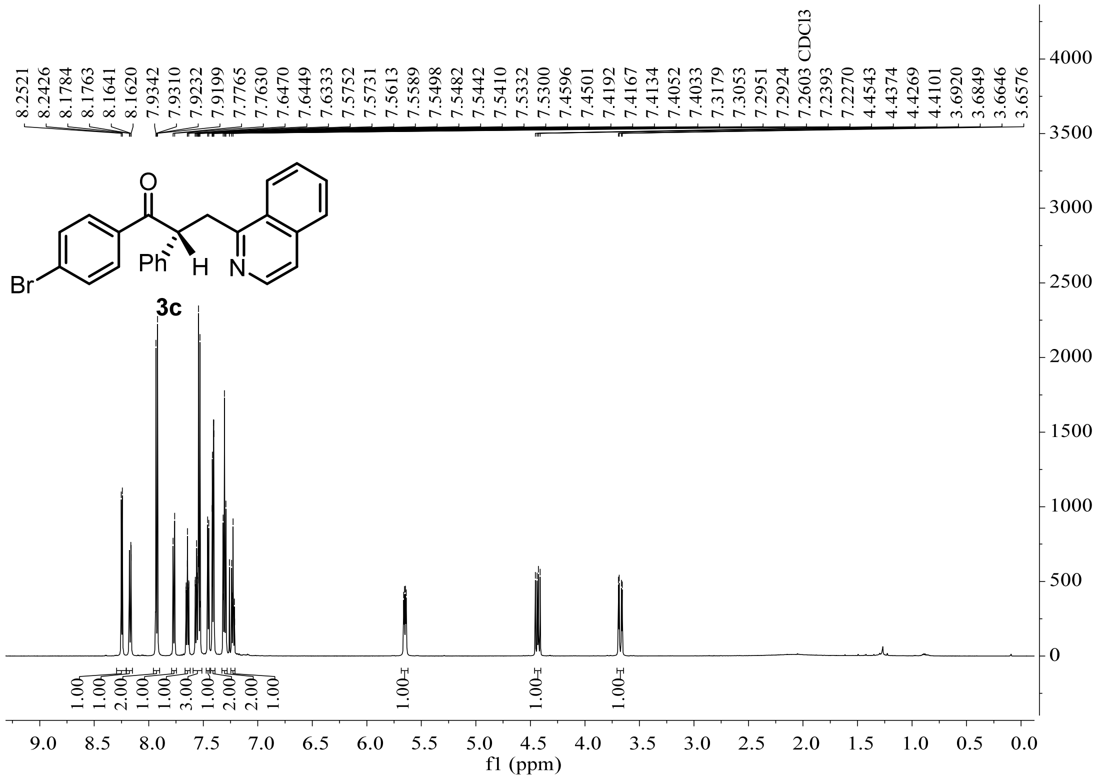

^{13}C NMR (151 MHz, Chloroform-*d*) of compound **3c**

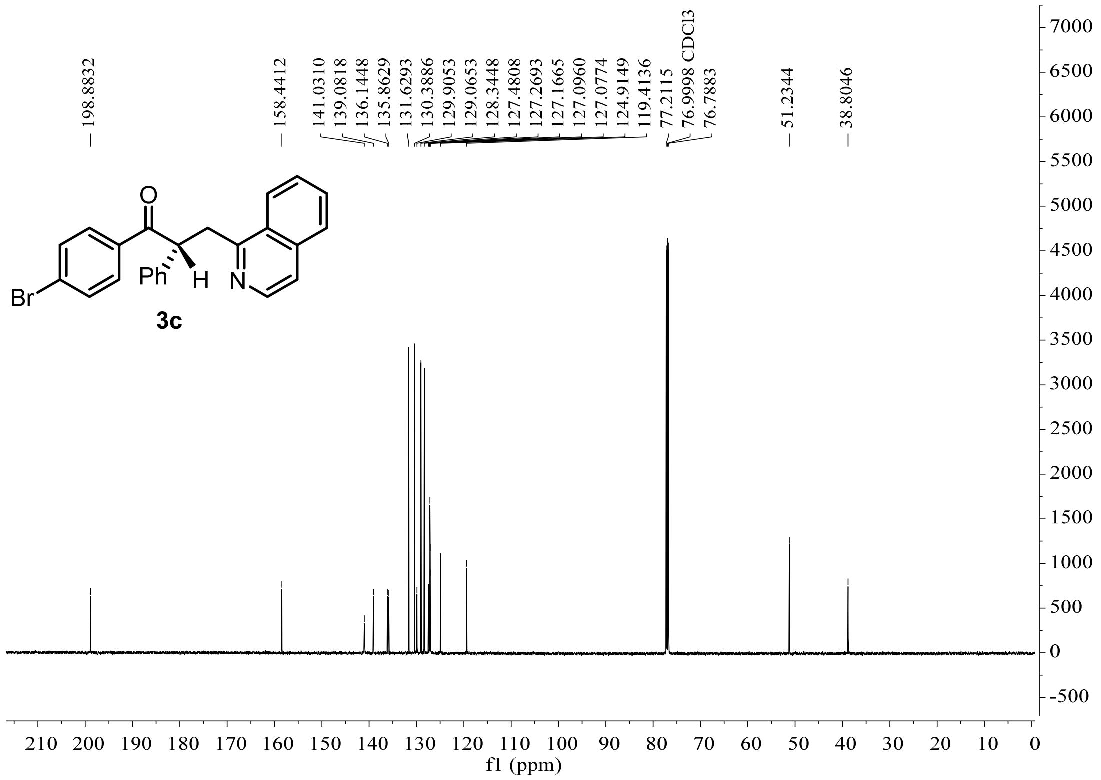

^{1}H NMR (300 MHz, Chloroform-d) of compound **3d**

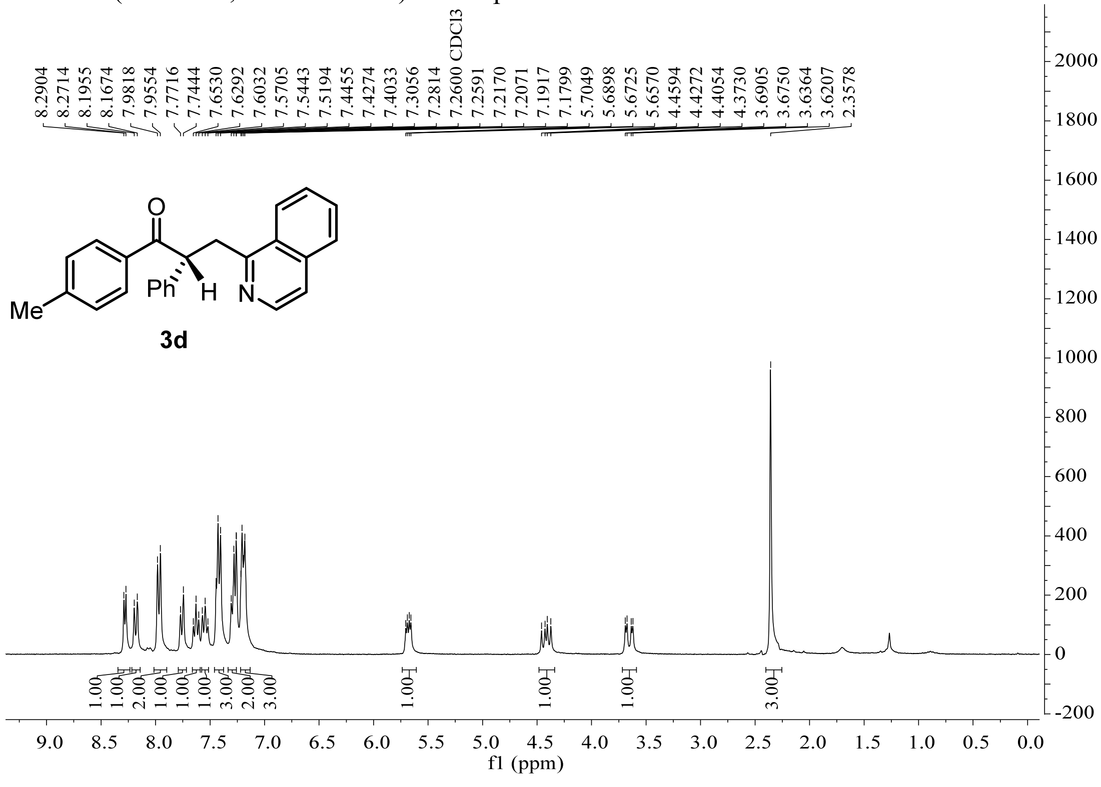

^{13}C NMR (75 MHz, Chloroform-*d*) of compound **3d**

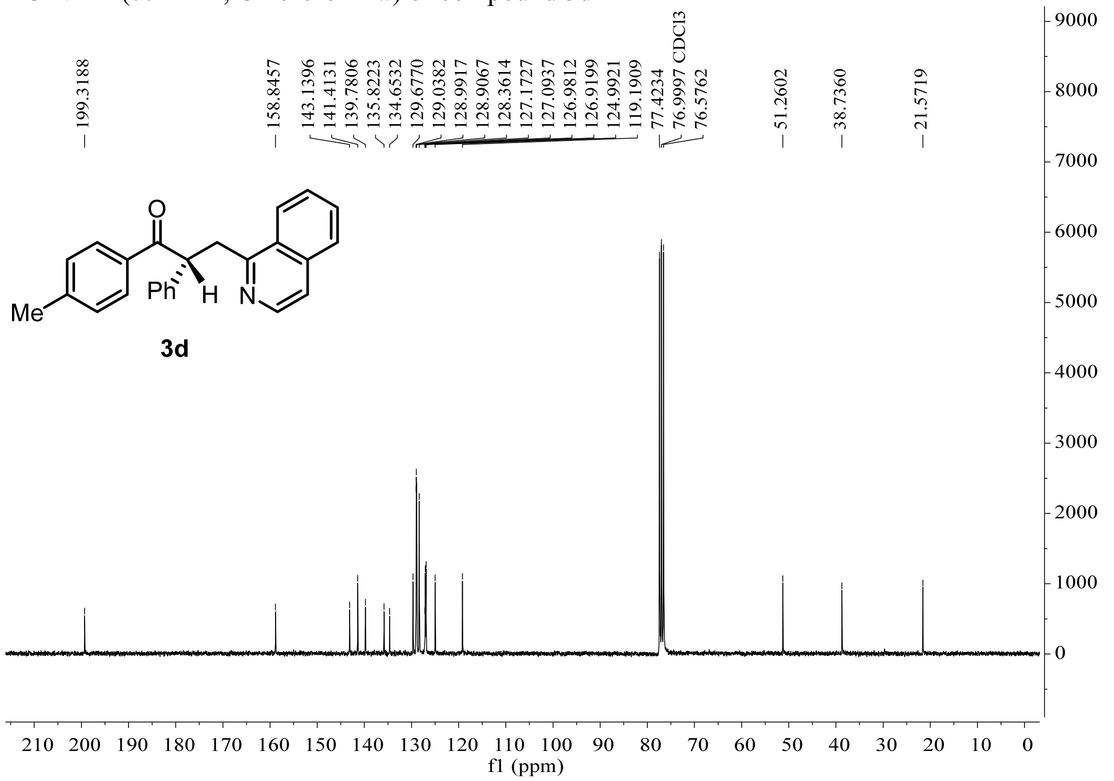

^{1}H NMR (300 MHz, Chloroform-d) of compound **3e**

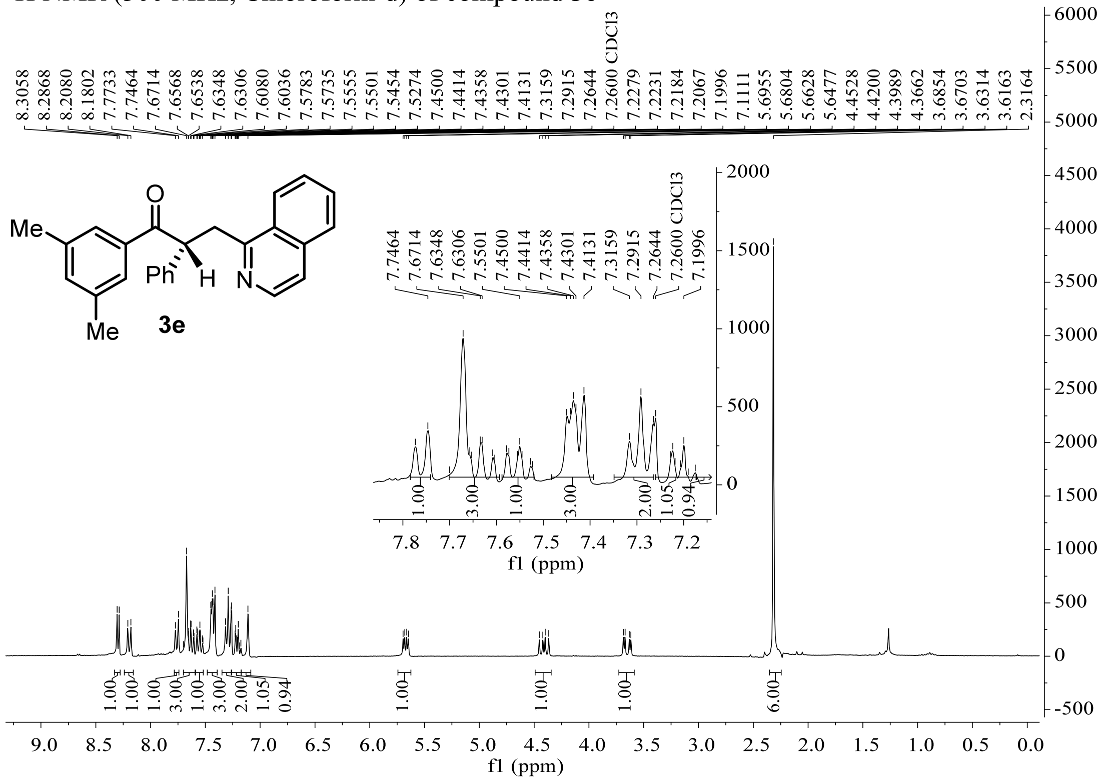

^{13}C NMR (75 MHz, Chloroform-*d*) of compound **3e**

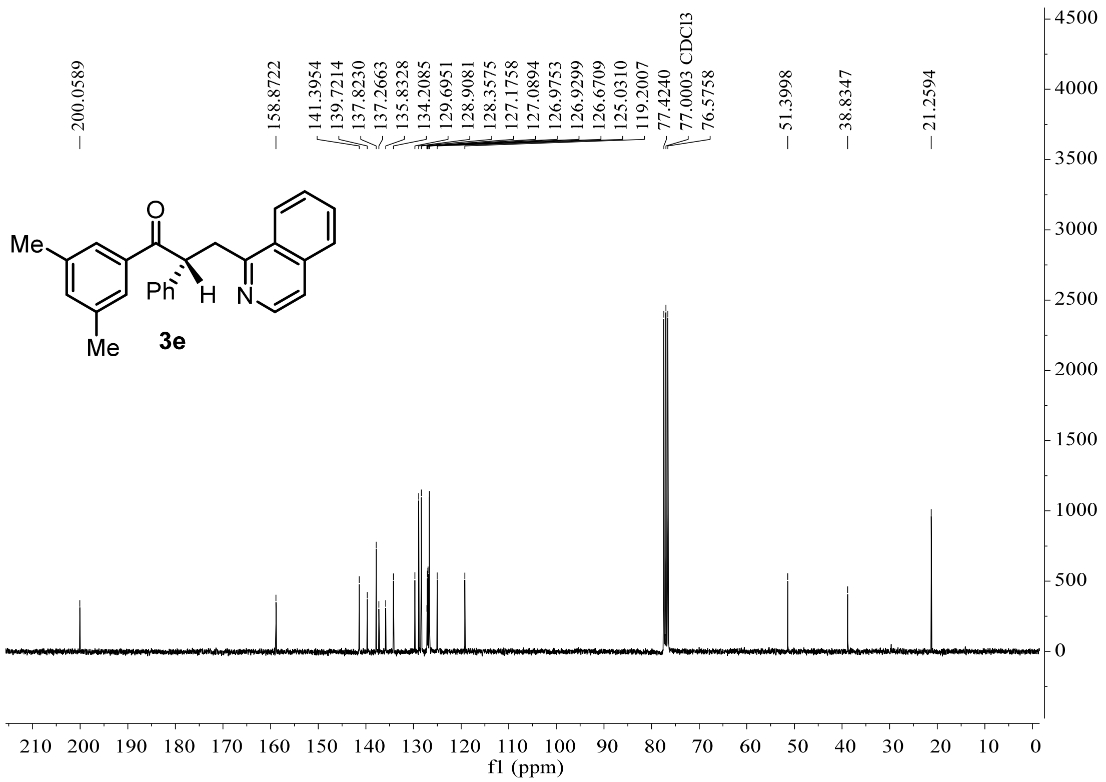

^{1}H NMR (300 MHz, Chloroform-d) of compound **3f**

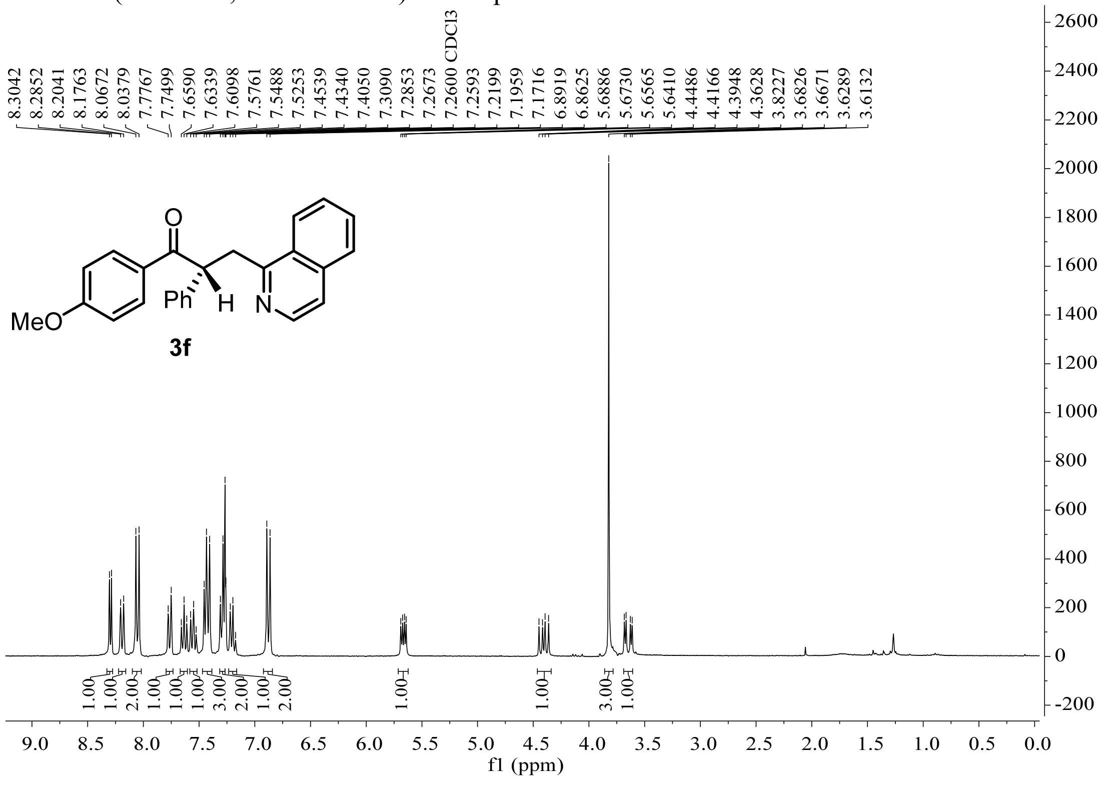

^{13}C NMR (75 MHz, Chloroform-*d*) of compound **3f**

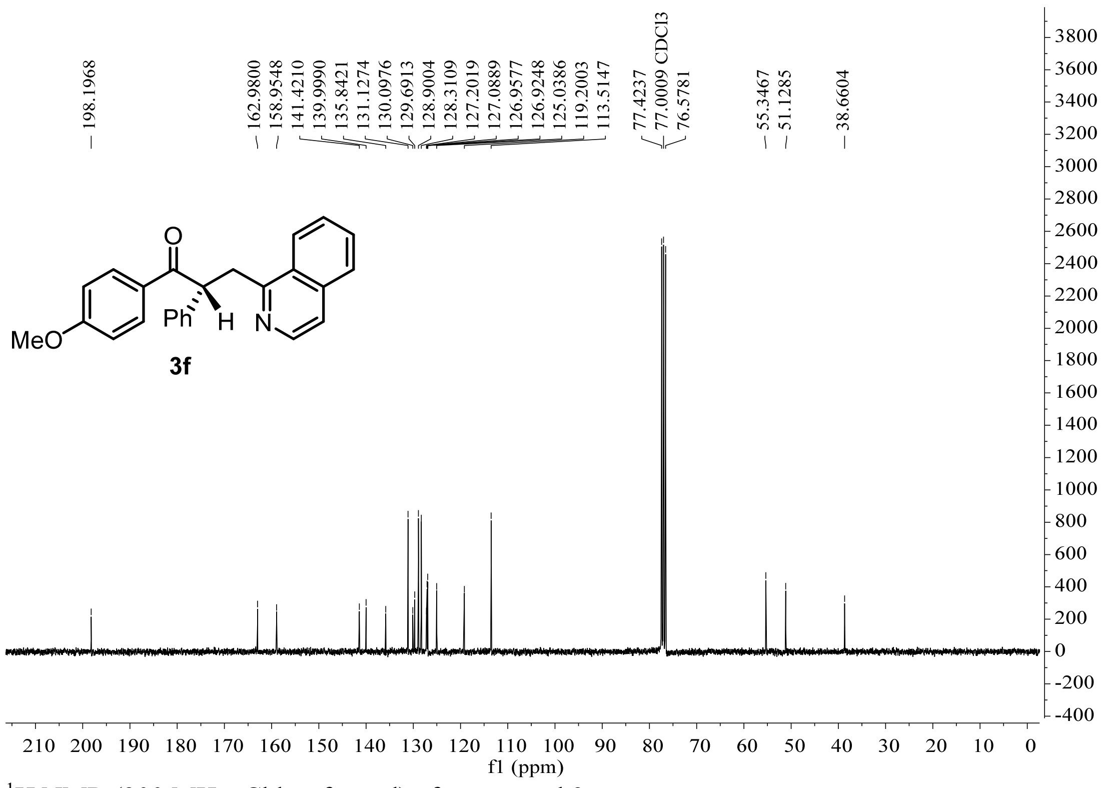

^{1}H NMR (300 MHz, Chloroform-d) of compound **3g**

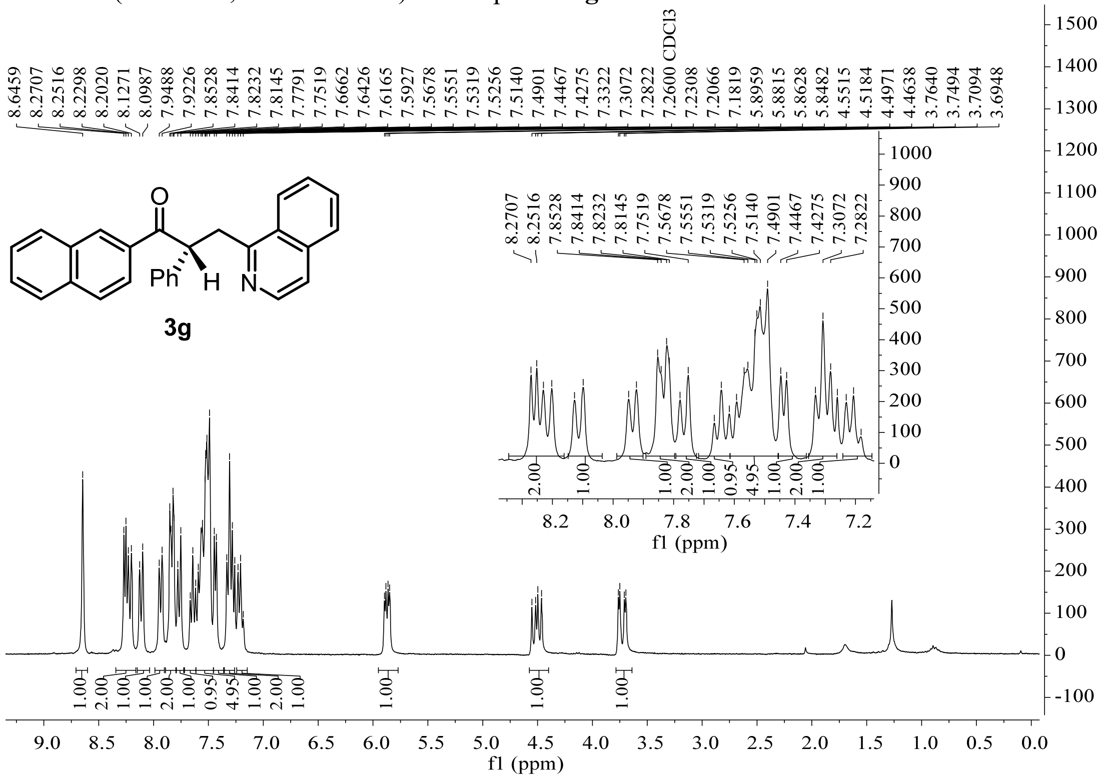

^{13}C NMR (75 MHz, Chloroform-*d*) of compound **3g**

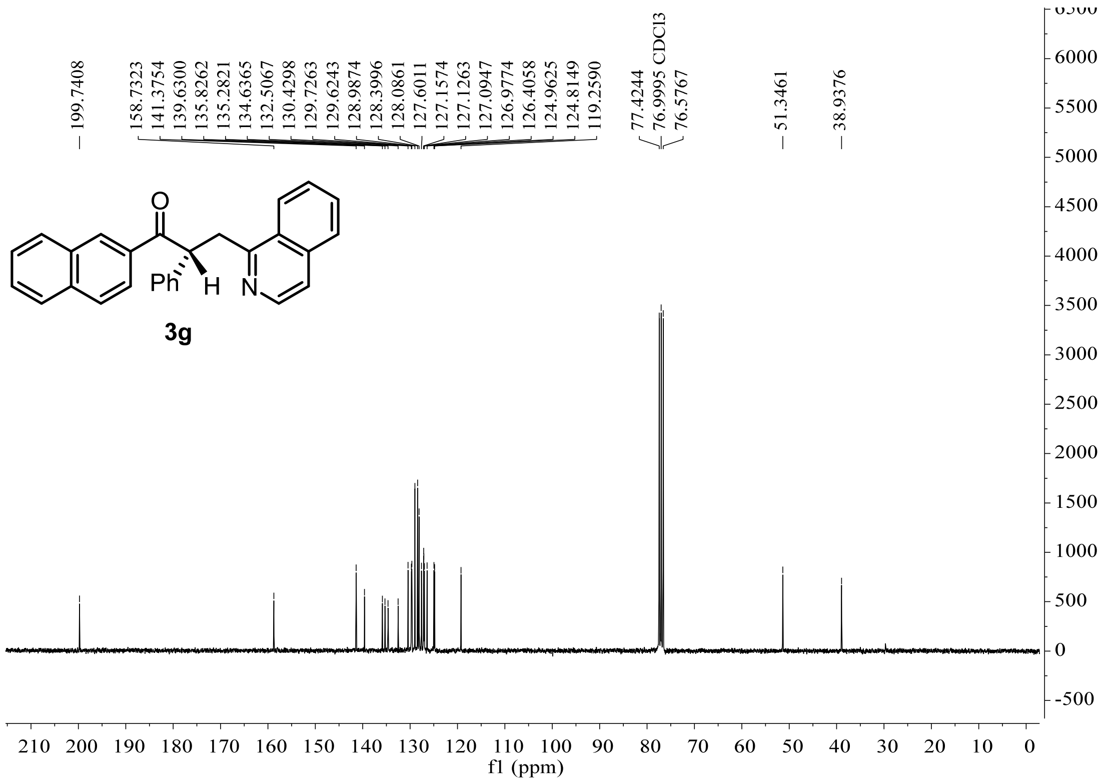

^{1}H NMR (400 MHz, Chloroform-d) of compound **3h**

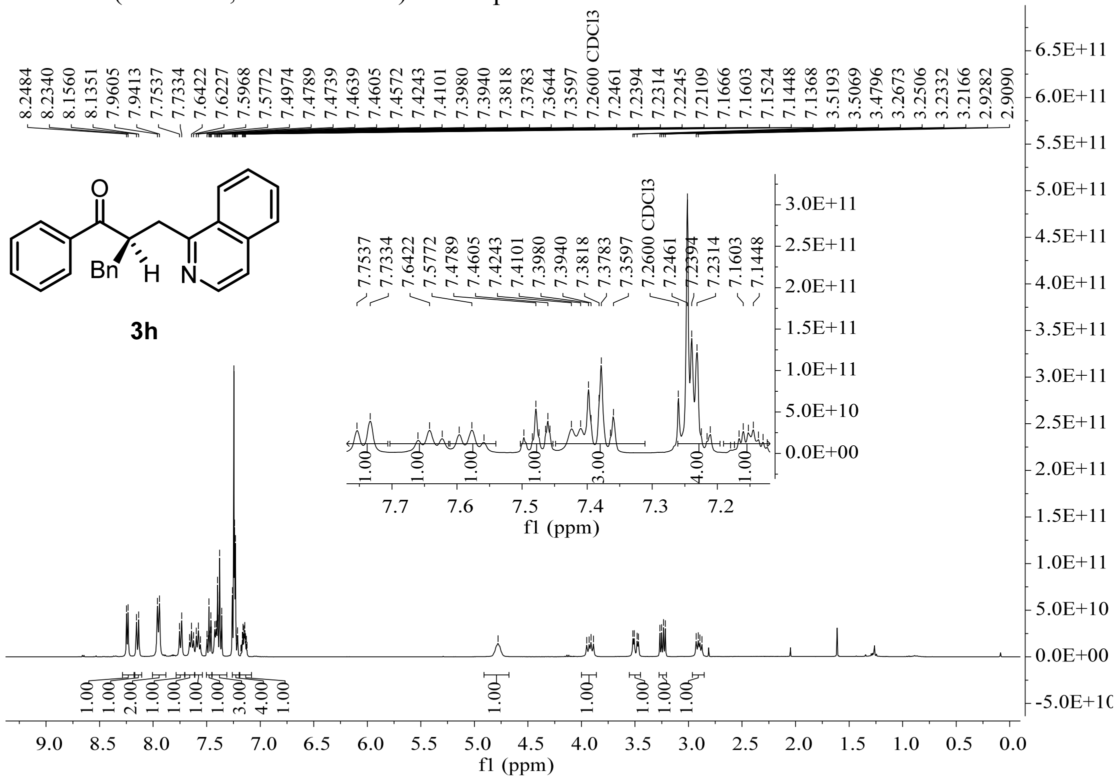

^{13}C NMR (101 MHz, Chloroform-*d*) of compound **3h**

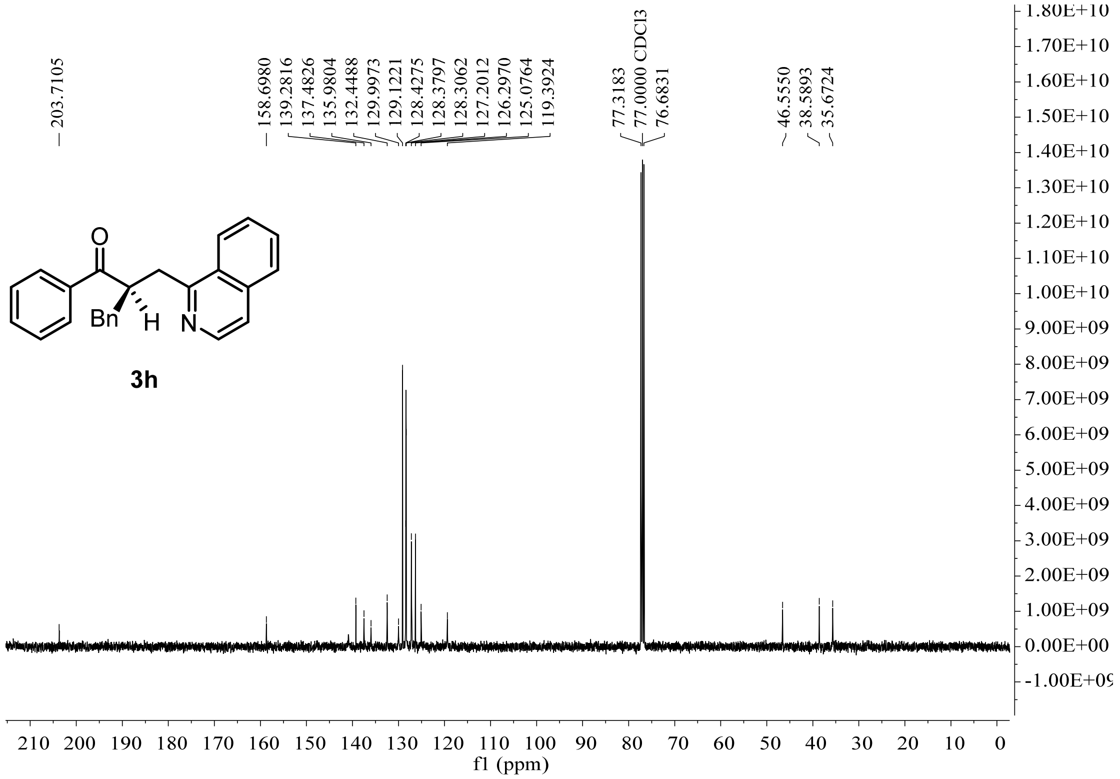

^{1}H NMR (300 MHz, Chloroform-d) of compound **3i**

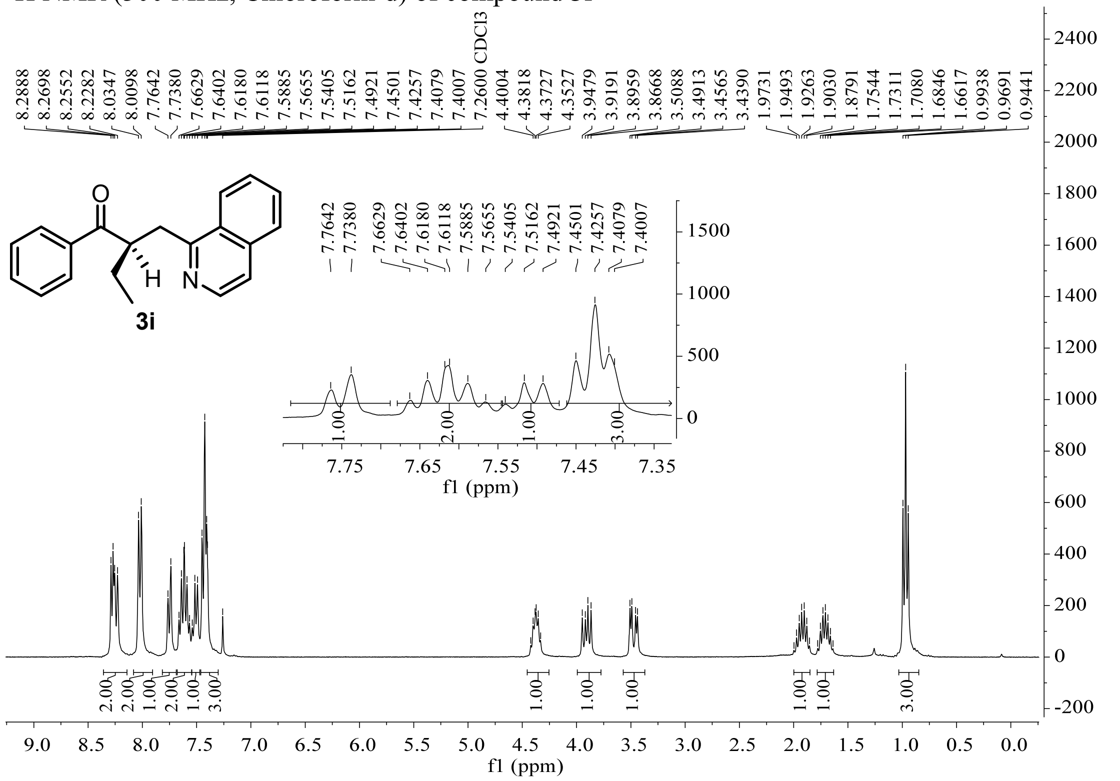

^{13}C NMR (75 MHz, Chloroform-*d*) of compound **3i**

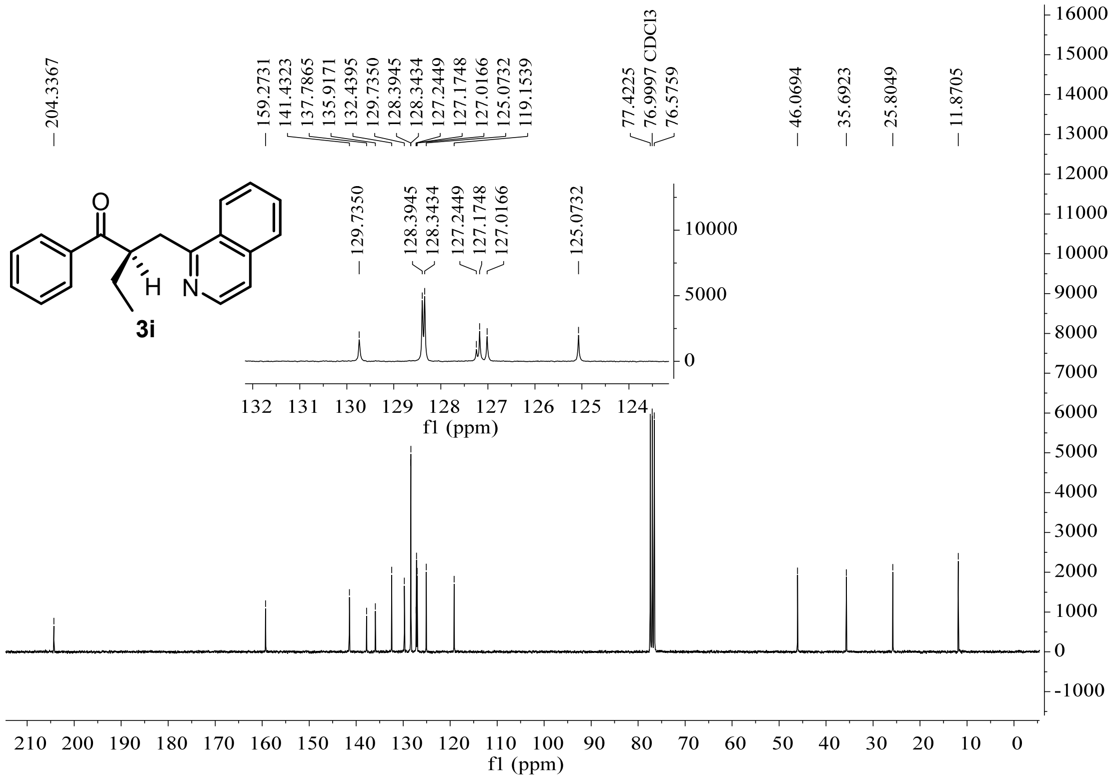

^{1}H NMR (300 MHz, Chloroform-d) of compound **3j**

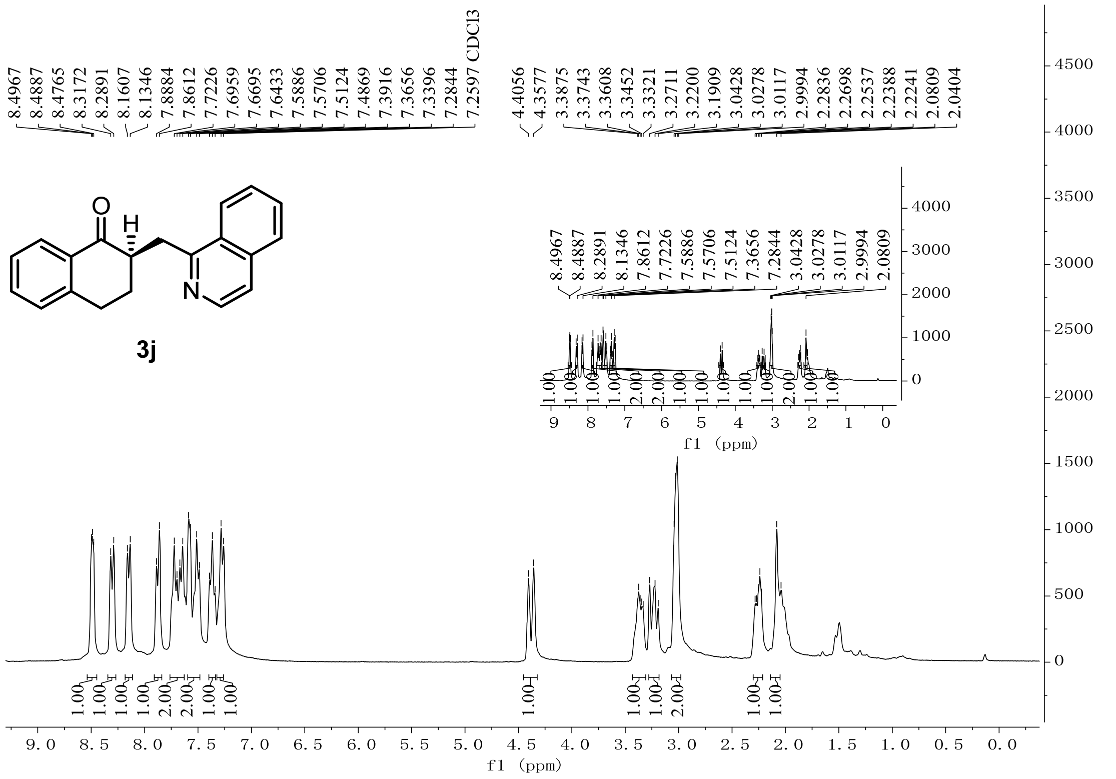

^{13}C NMR (75 MHz, Chloroform-*d*) of compound **3j**

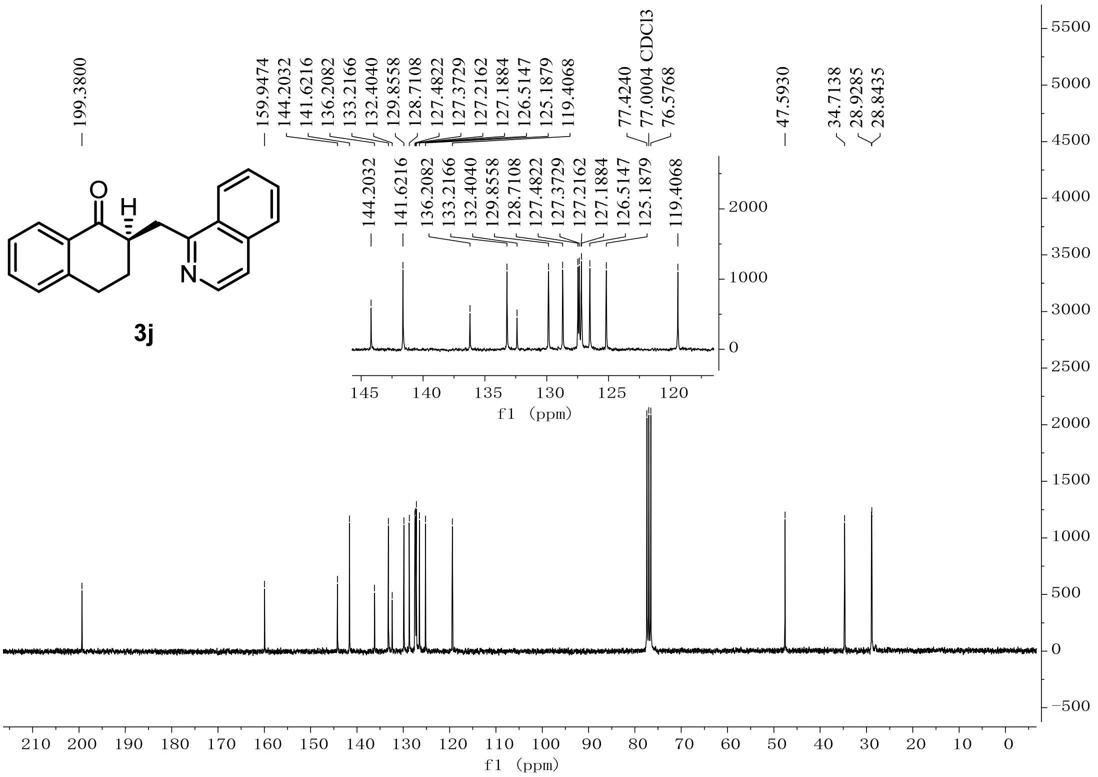

^{1}H NMR (300 MHz, Chloroform-d) of compound **3k**

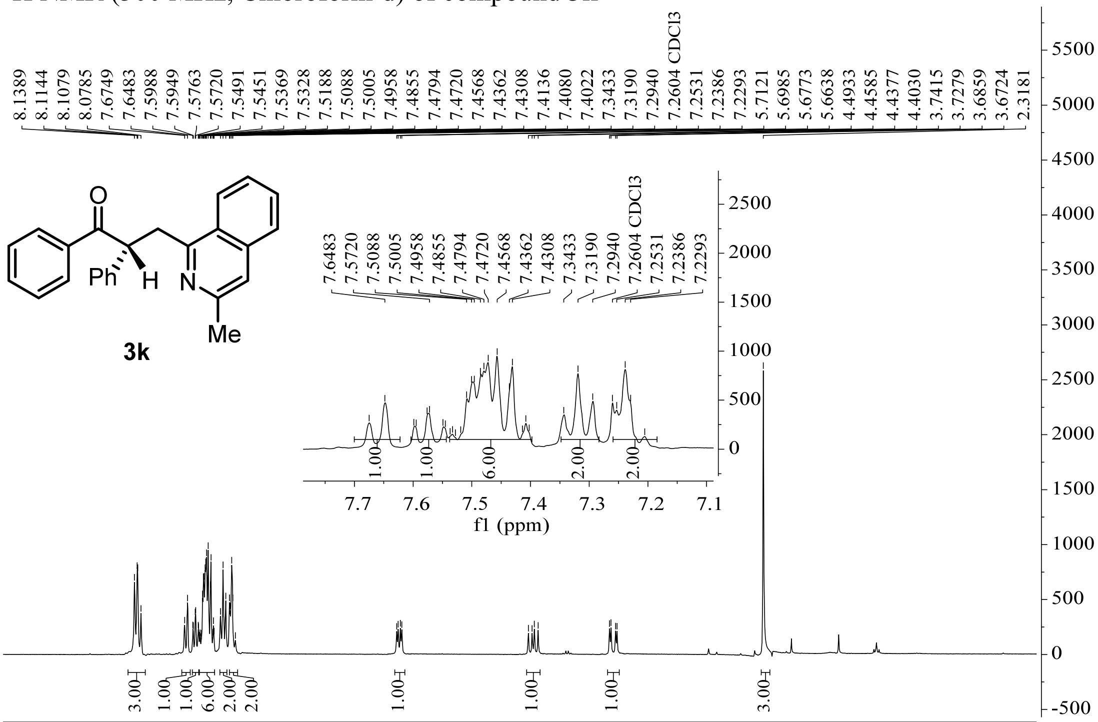

^{13}C NMR (75 MHz, Chloroform-*d*) of compound **3k**

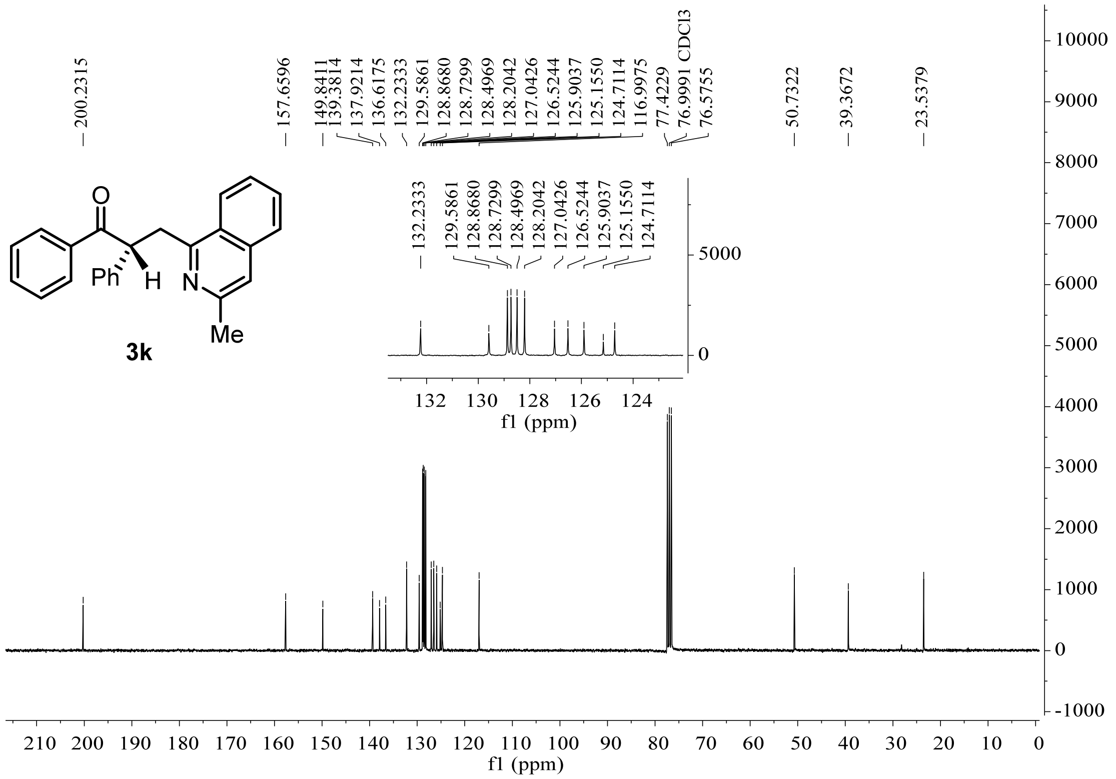

^{1}H NMR (300 MHz, Chloroform-d) of compound **3l**

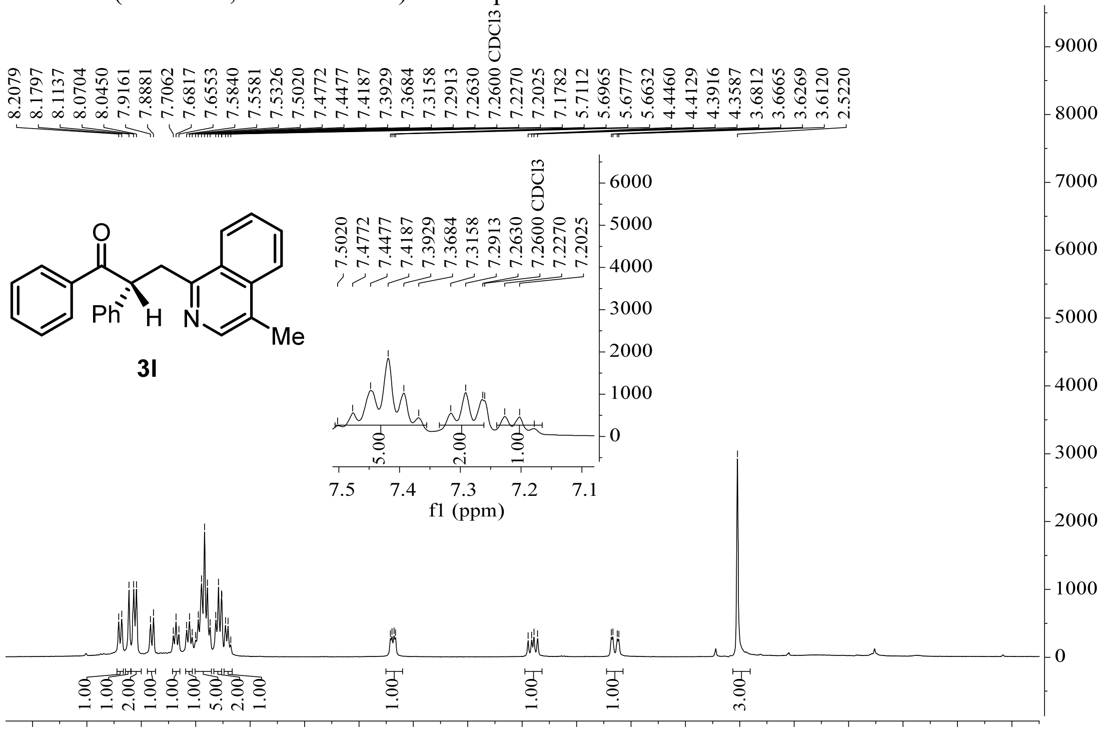

^{13}C NMR (75 MHz, Chloroform-*d*) of compound **3l**

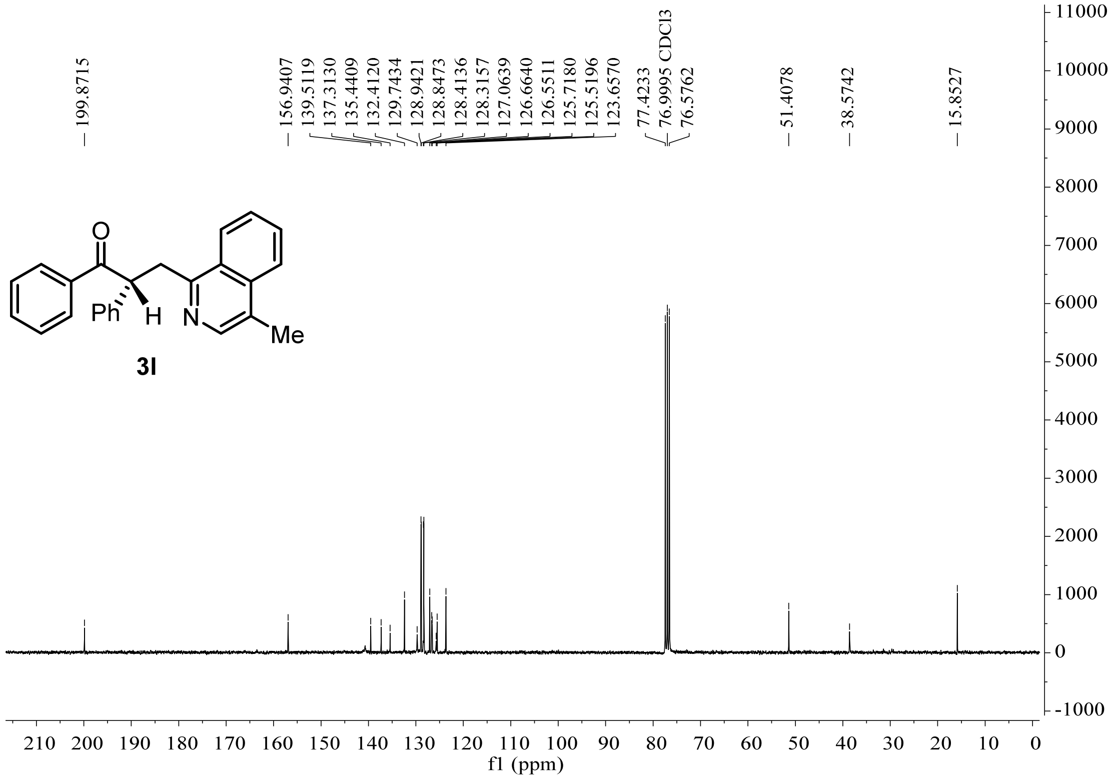

^{1}H NMR (300 MHz, Chloroform-d) of compound **3m**

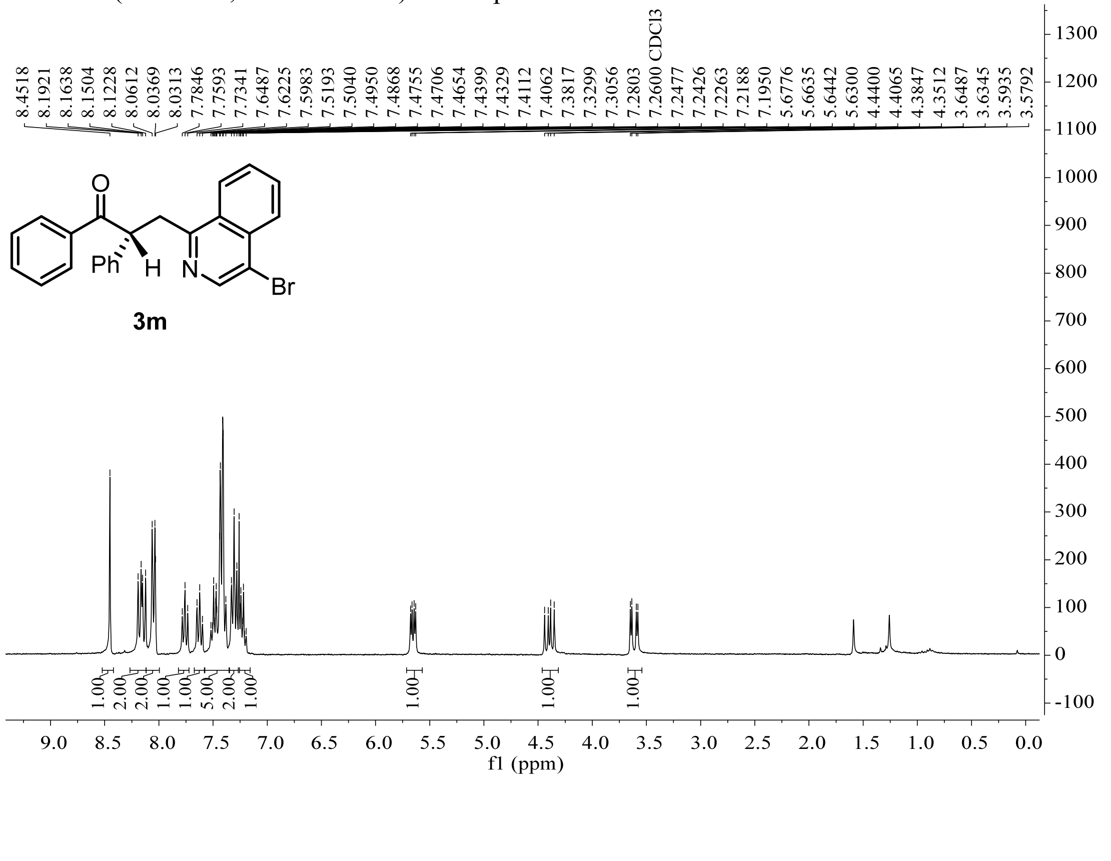

^{13}C NMR (75 MHz, Chloroform-*d*) of compound **3m**

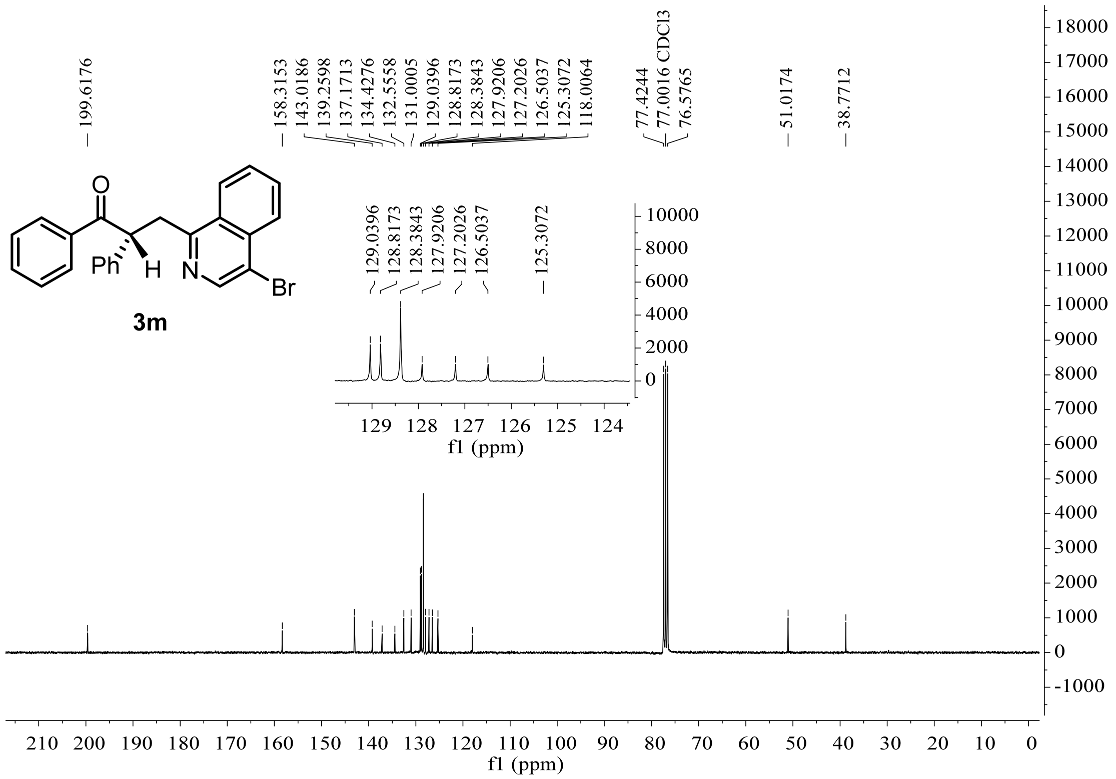

^{1}H NMR (400 MHz, Chloroform-d) of compound **3n**

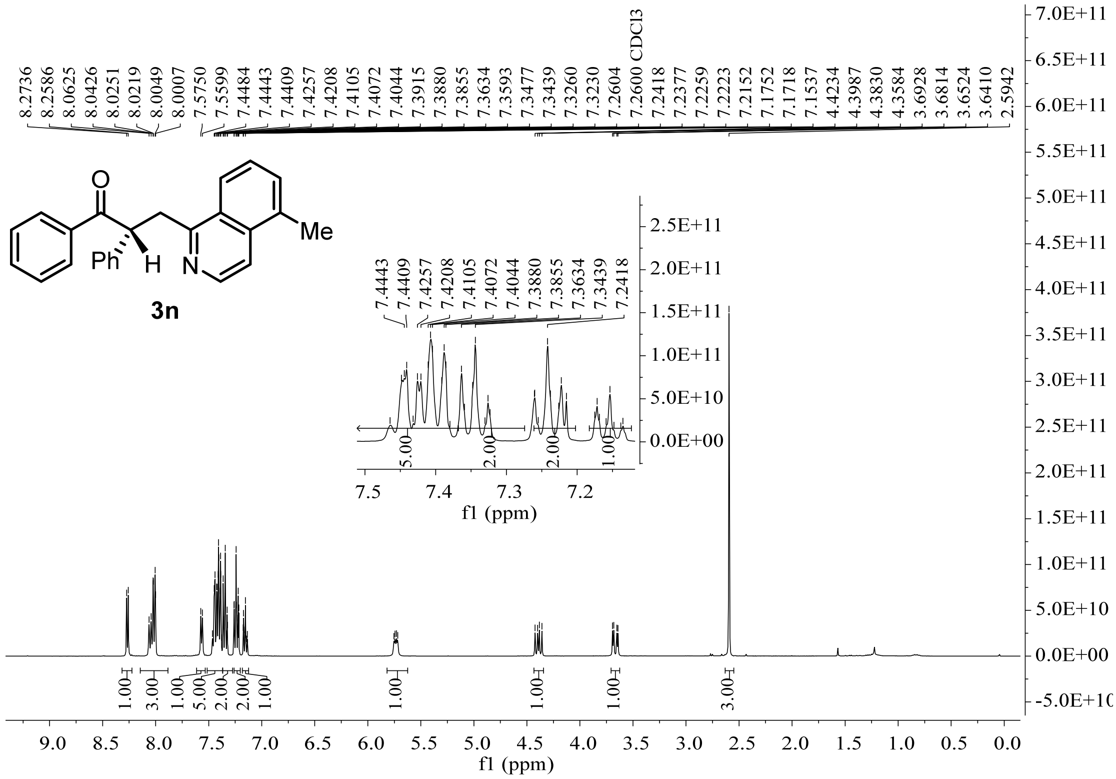

^{13}C NMR (101 MHz, Chloroform-*d*) of compound **3n**

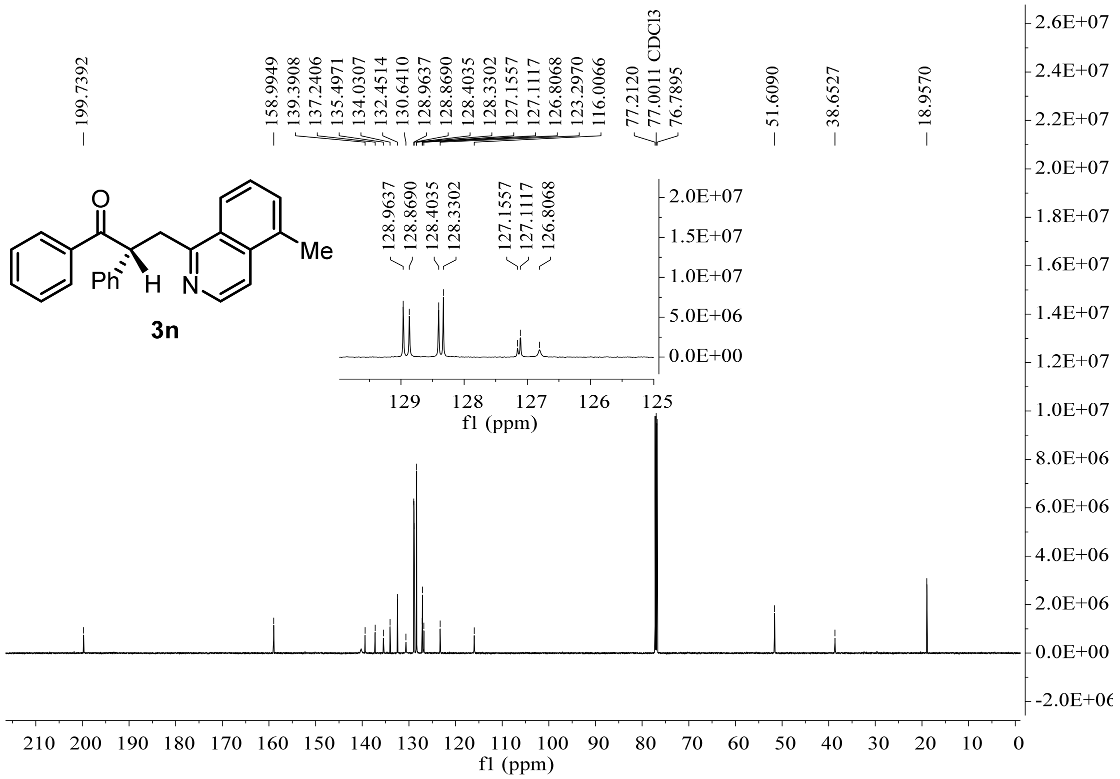

^{1}H NMR (300 MHz, Chloroform-d) of compound **3o**

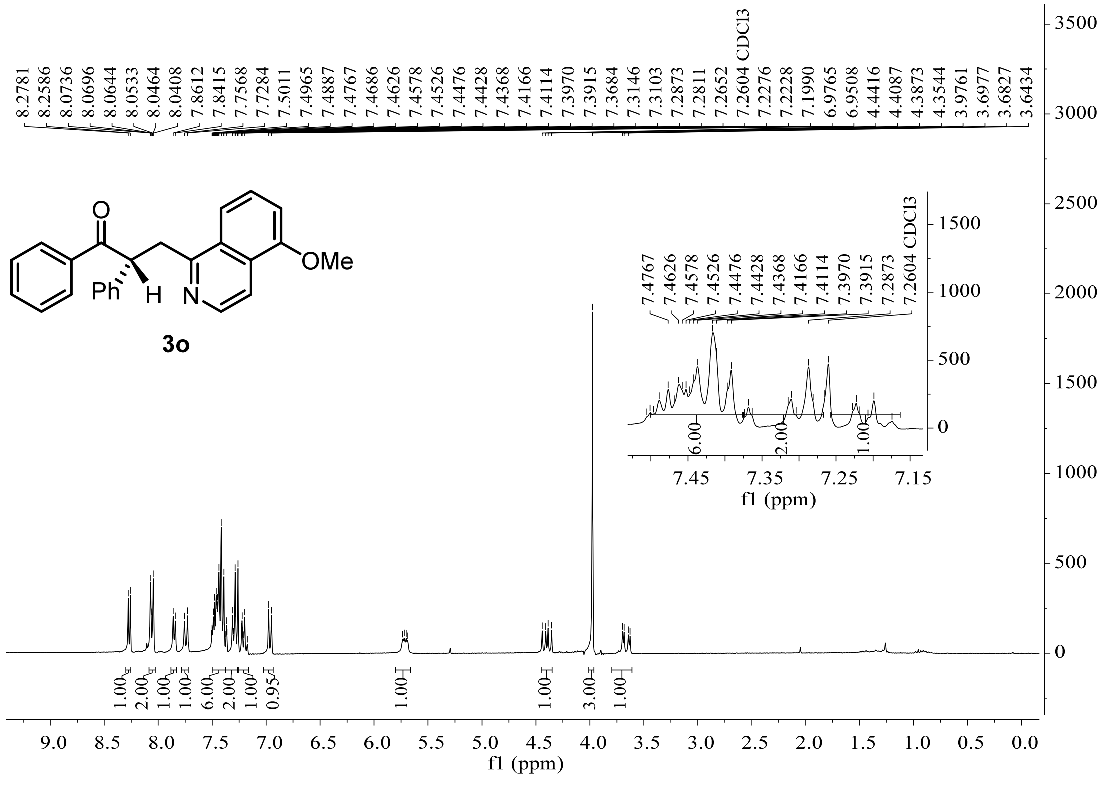

^{13}C NMR (75 MHz, Chloroform-*d*) of compound **3o**

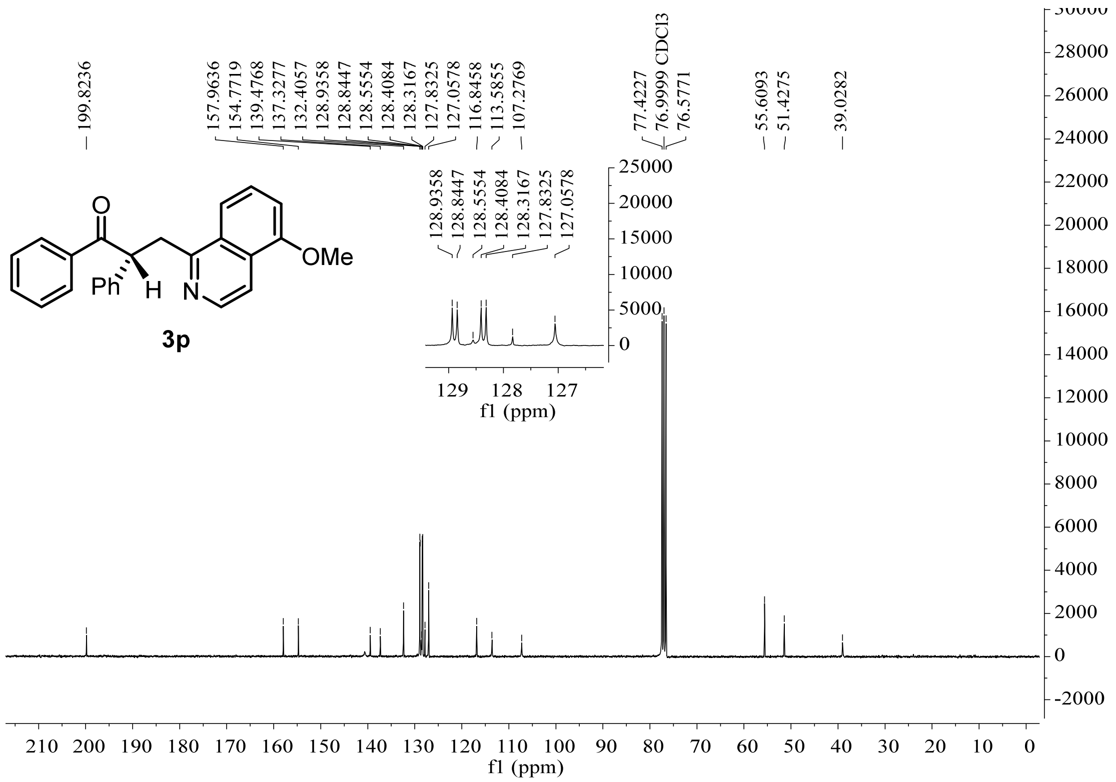

^{1}H NMR (300 MHz, Chloroform-d) of compound **3p**

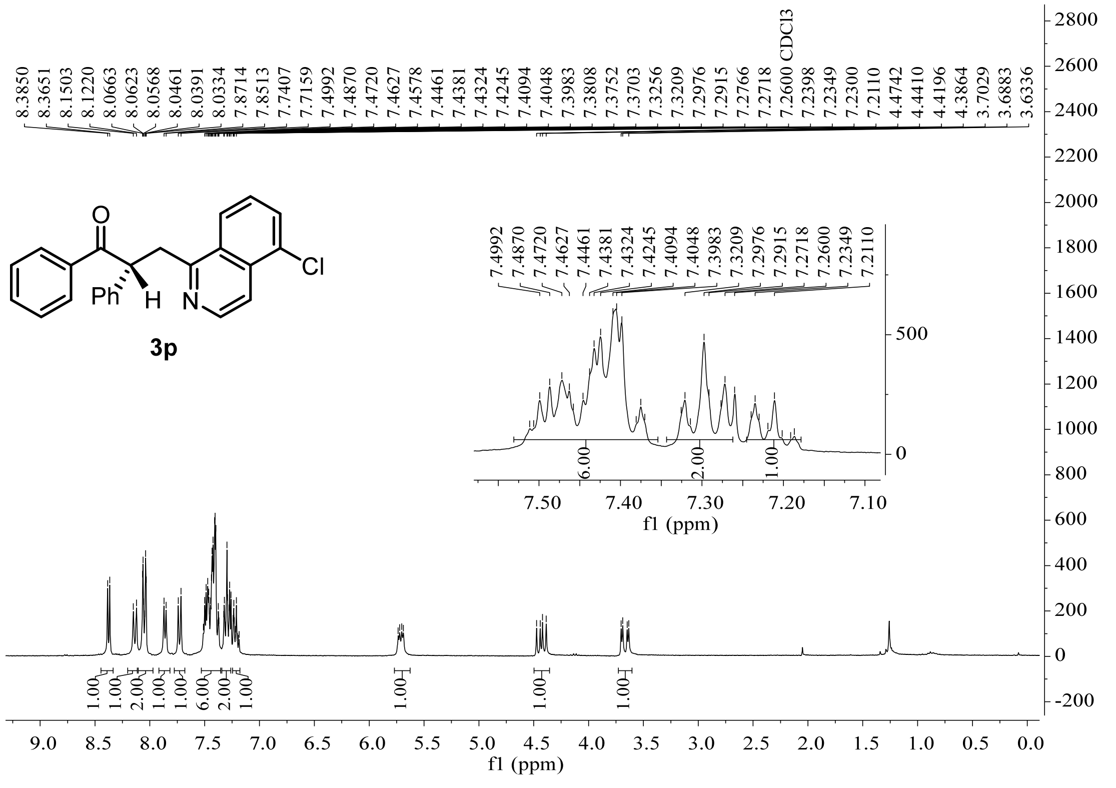

^{13}C NMR (75 MHz, Chloroform-*d*) of compound **3p**

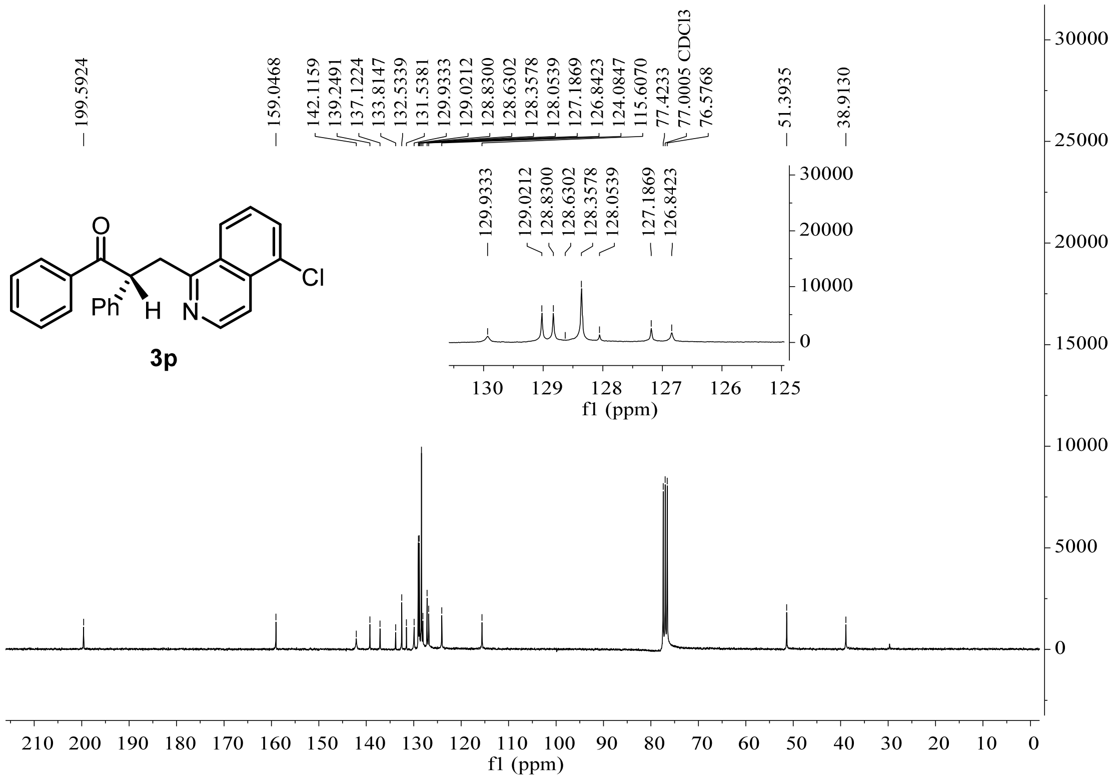

^{1}H NMR (300 MHz, Chloroform-d) of compound **3q**

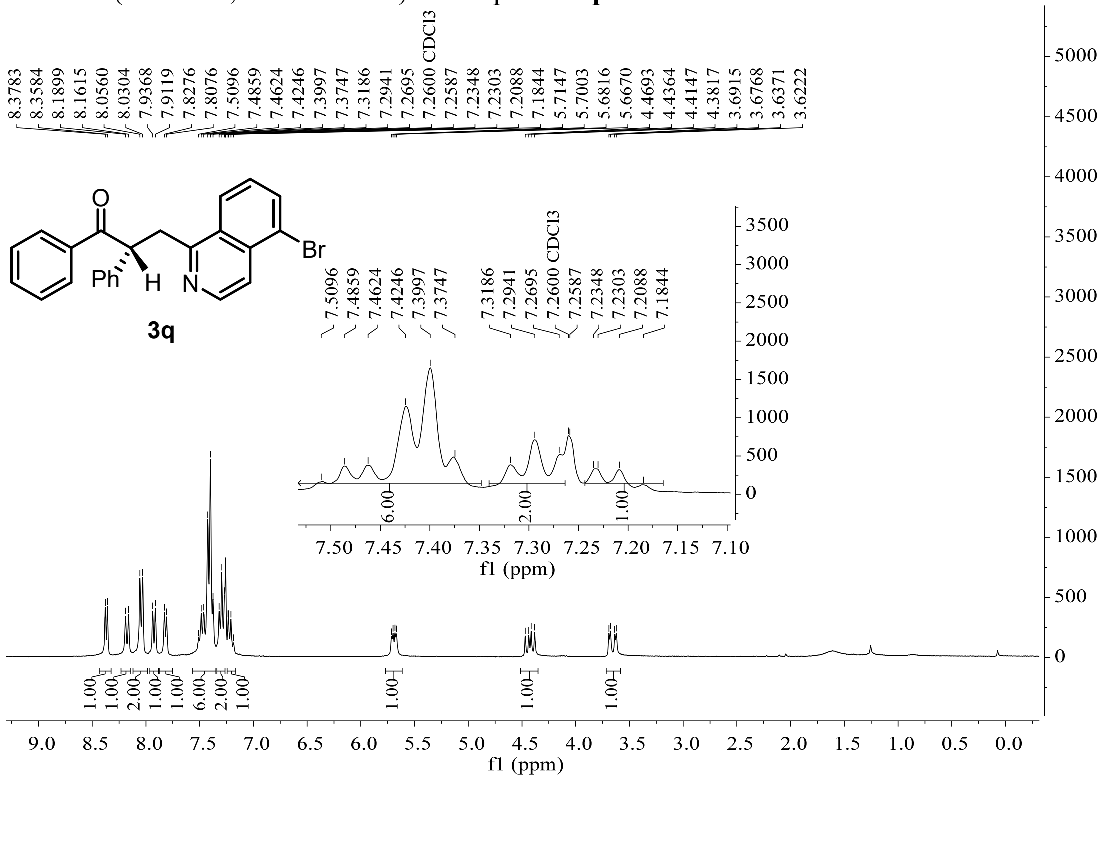

^{13}C NMR (75 MHz, Chloroform-*d*) of compound **3q**

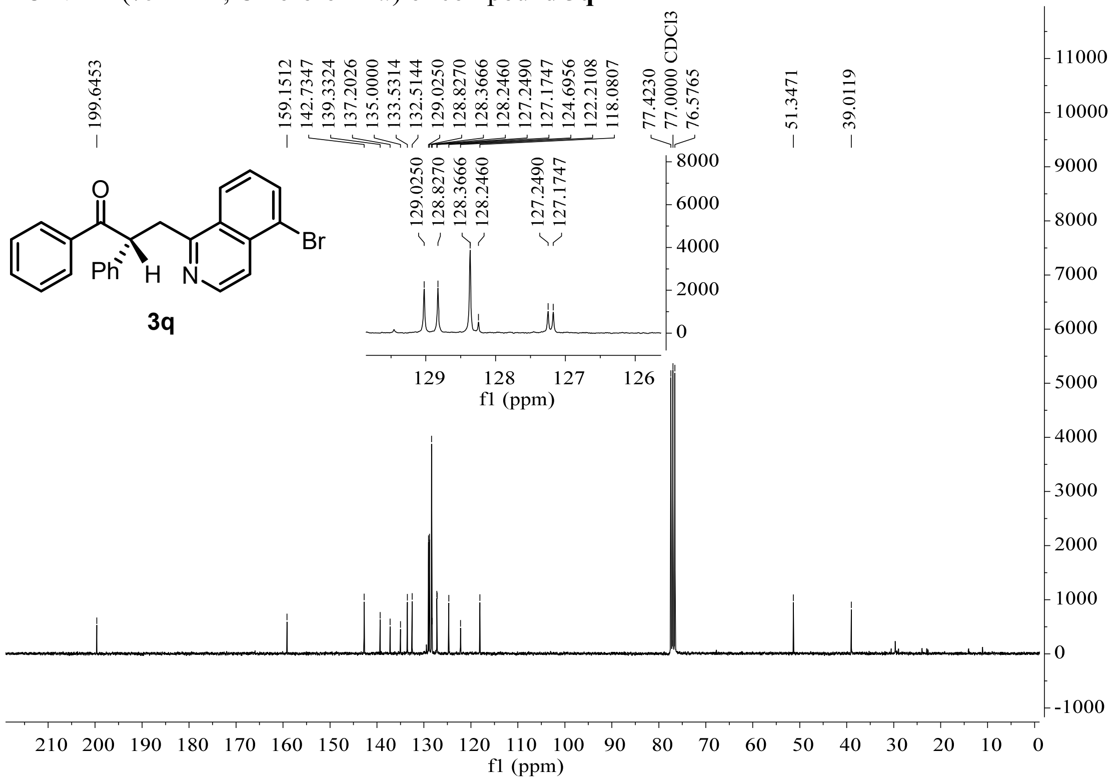

^{1}H NMR (300 MHz, Chloroform-d) of compound **3r**

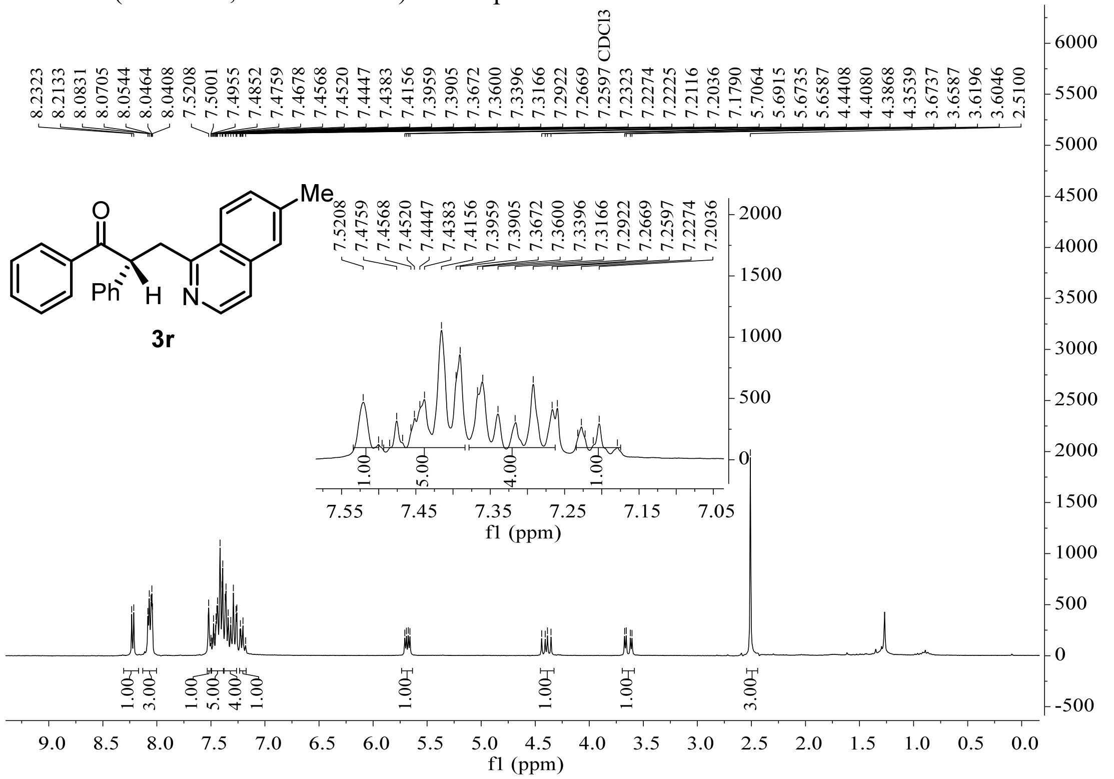

^{13}C NMR (75 MHz, Chloroform-*d*) of compound **3r**

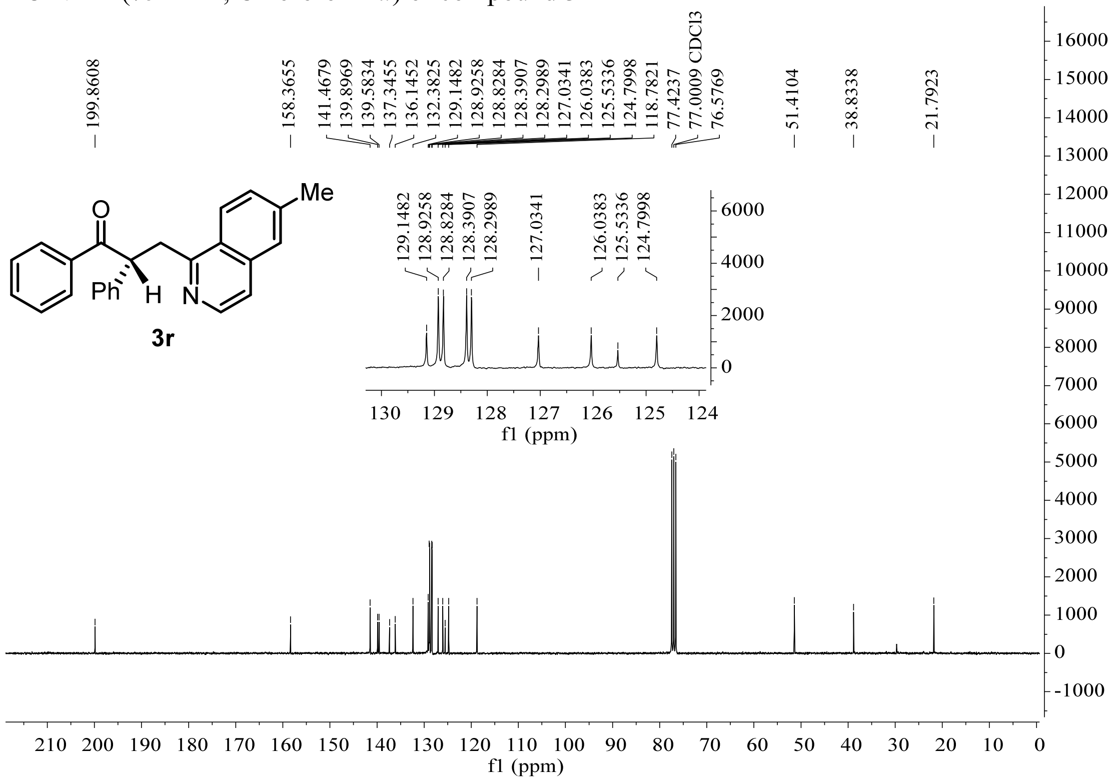

^{1}H NMR (300 MHz, Chloroform-d) of compound **3s**

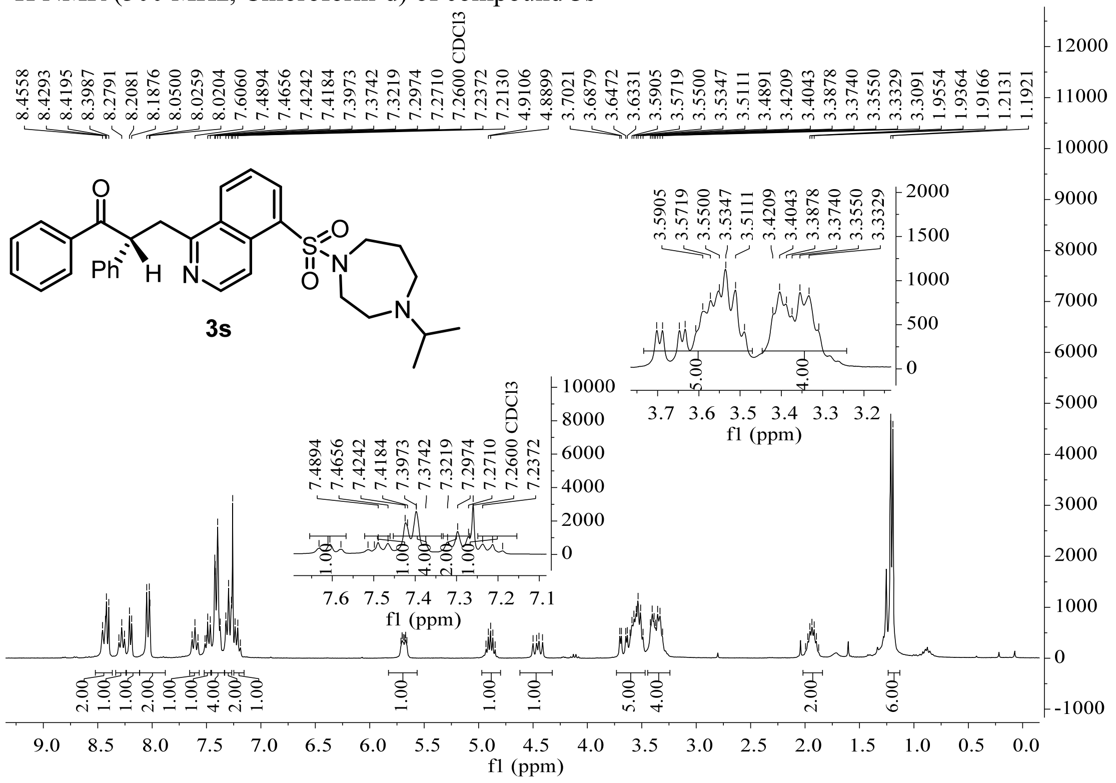

^{13}C NMR (75 MHz, Chloroform-*d*) of compound **3s**

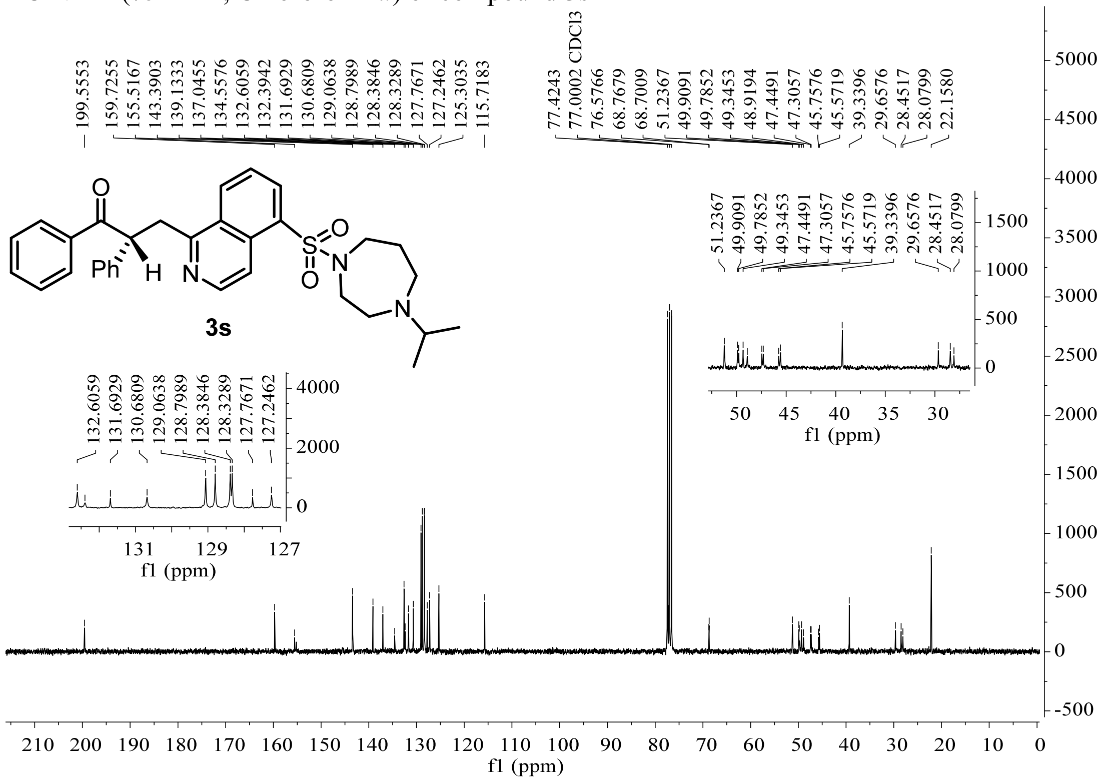

^{1}H NMR (300 MHz, Chloroform-d) of compound **3t**

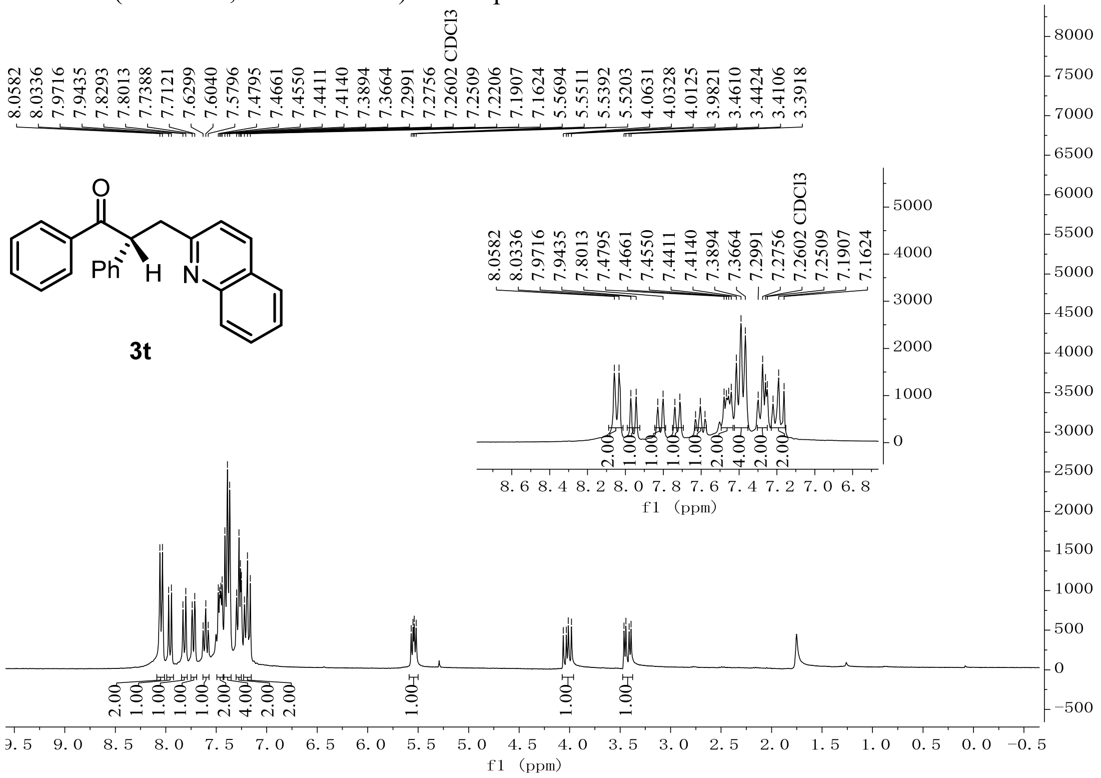

^{13}C NMR (75 MHz, Chloroform-*d*) of compound **3t**

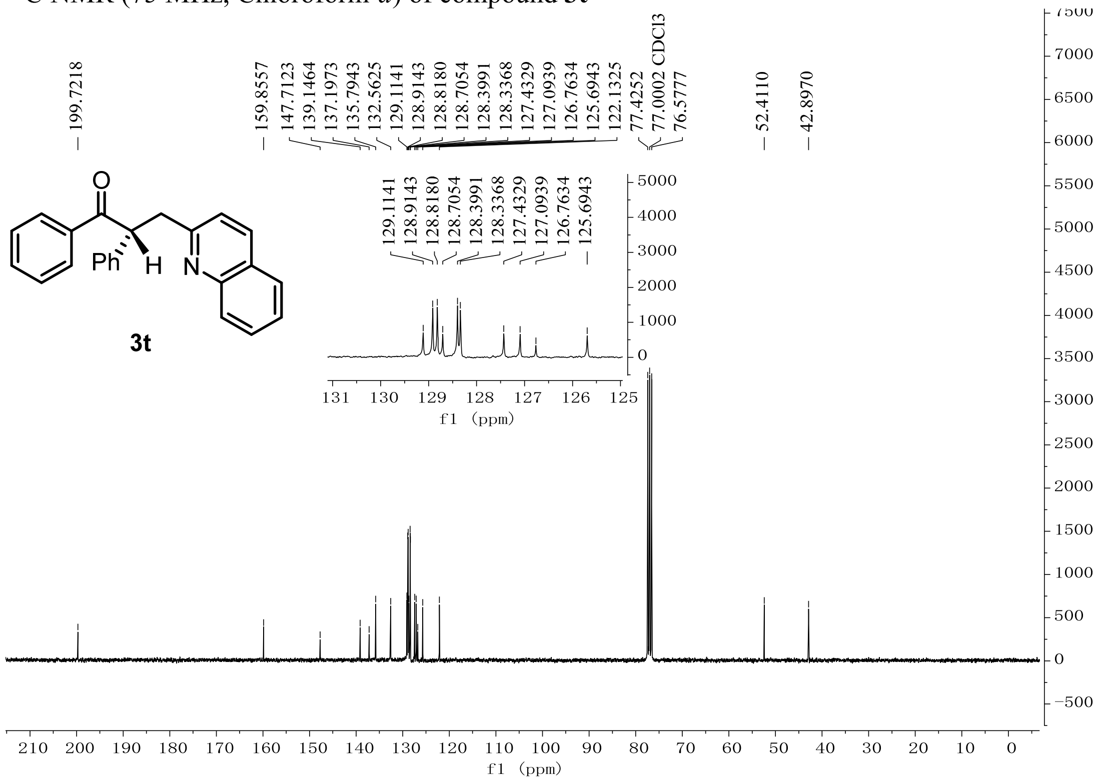

^{1}H NMR (400 MHz, Chloroform-d) of compound **3u**

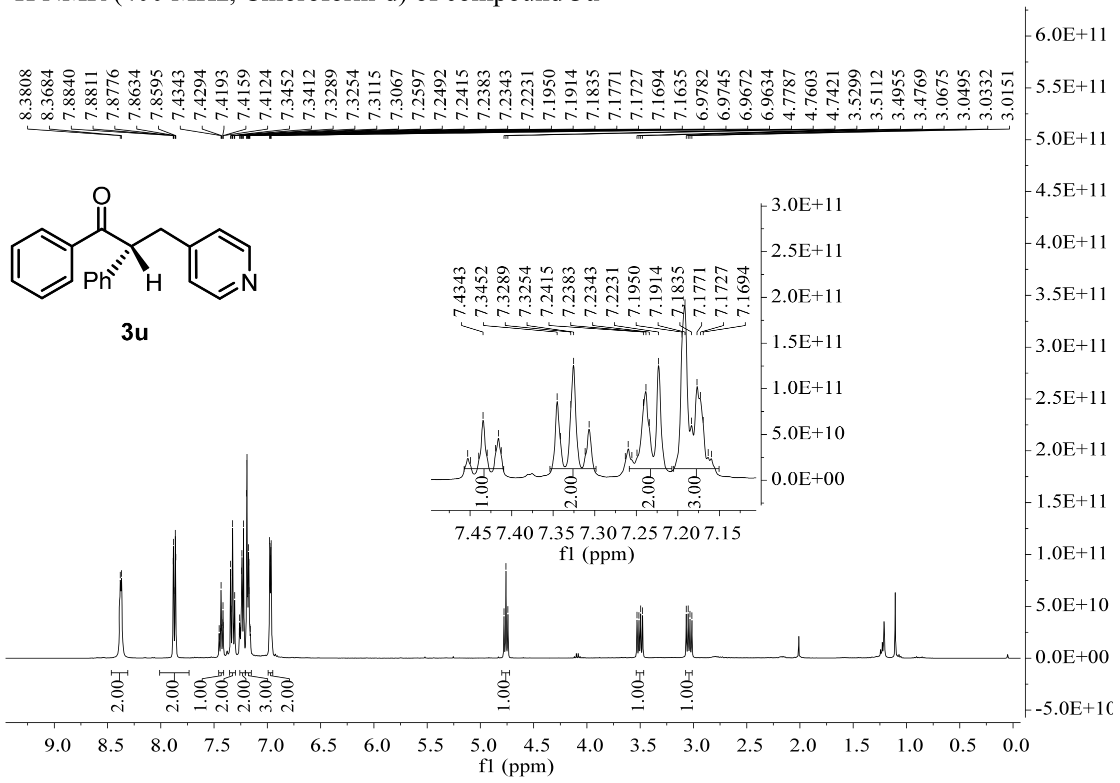

^{13}C NMR (101 MHz, Chloroform-*d*) of compound **3u**

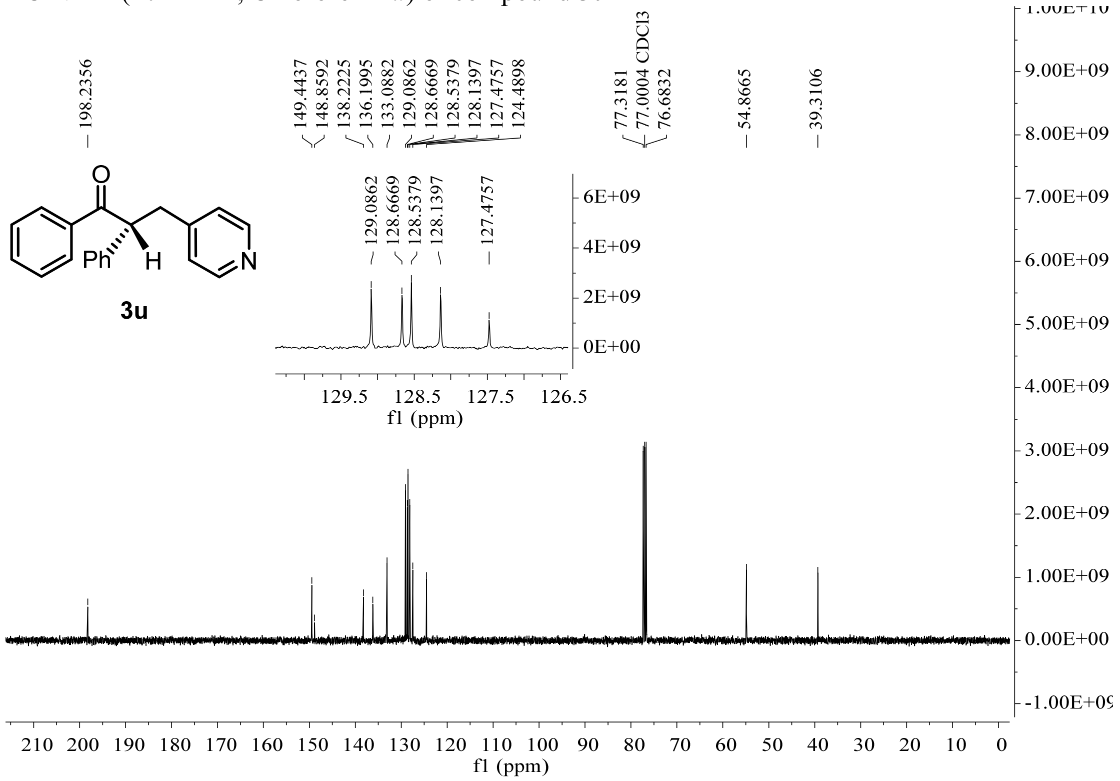

^{1}H NMR (400 MHz, Chloroform-d) of compound **3v**

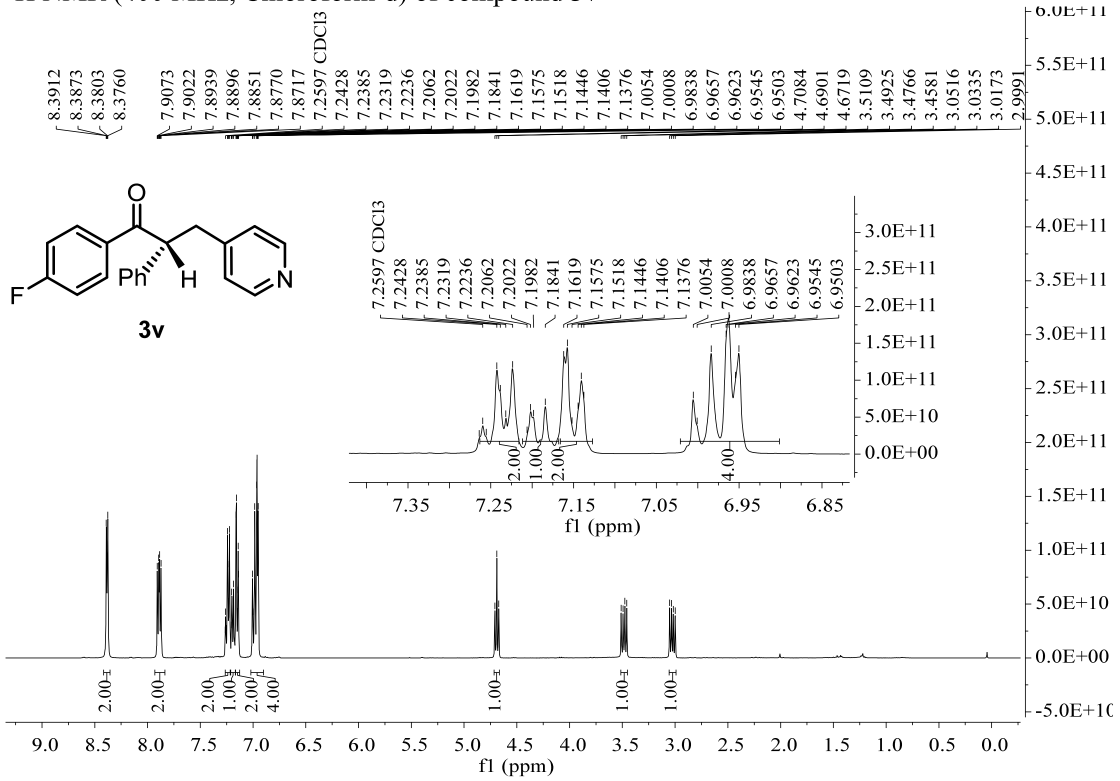

^{13}C NMR (101 MHz, Chloroform-*d*) of compound **3v**

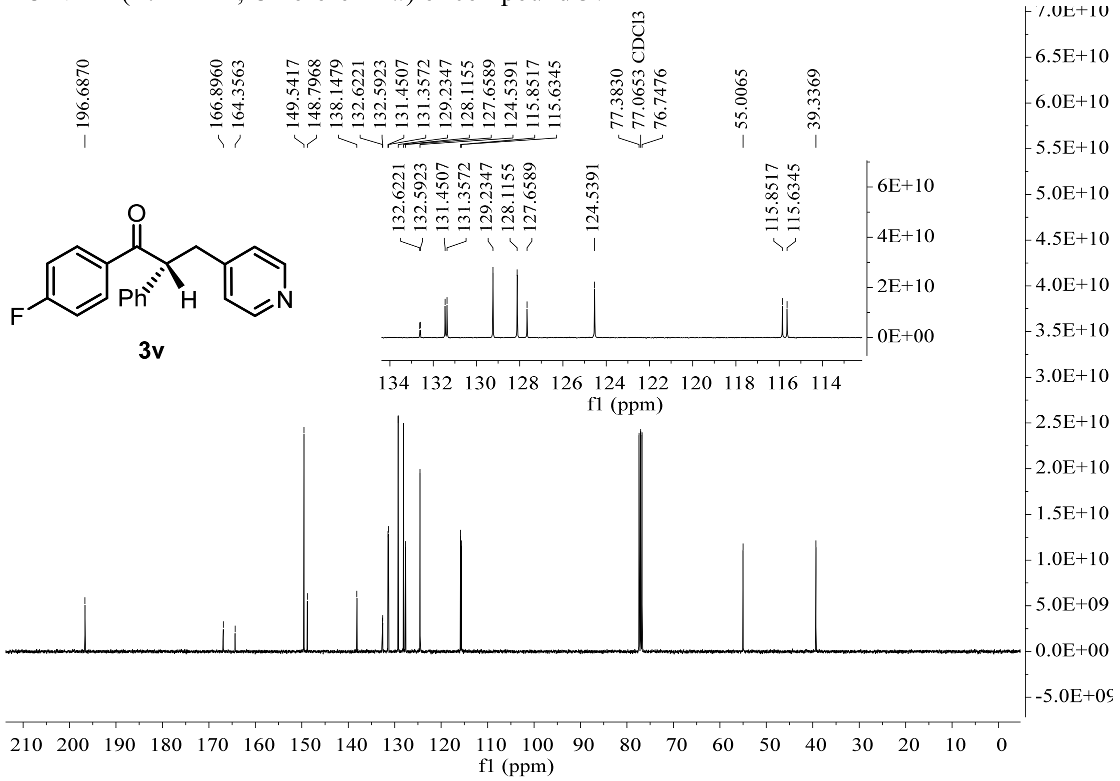

^{19}F NMR (376 MHz, Chloroform-*d*) of compound **3v**

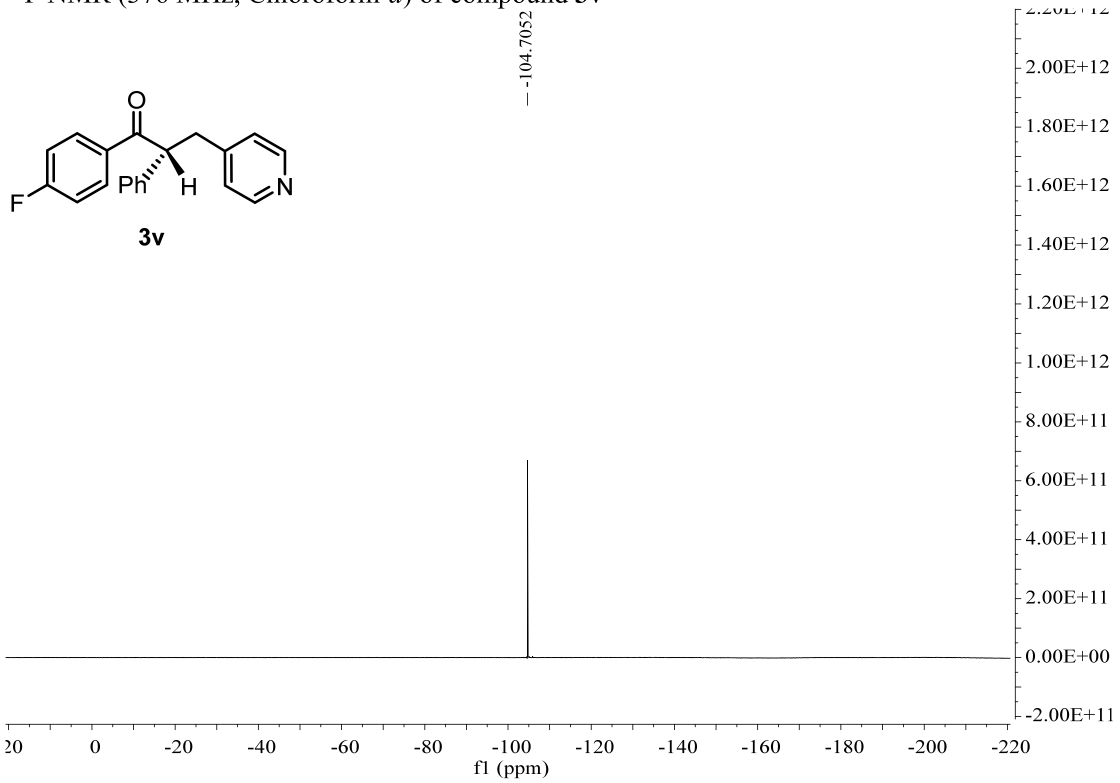

^{1}H NMR (400 MHz, Chloroform-d) of compound **3w**

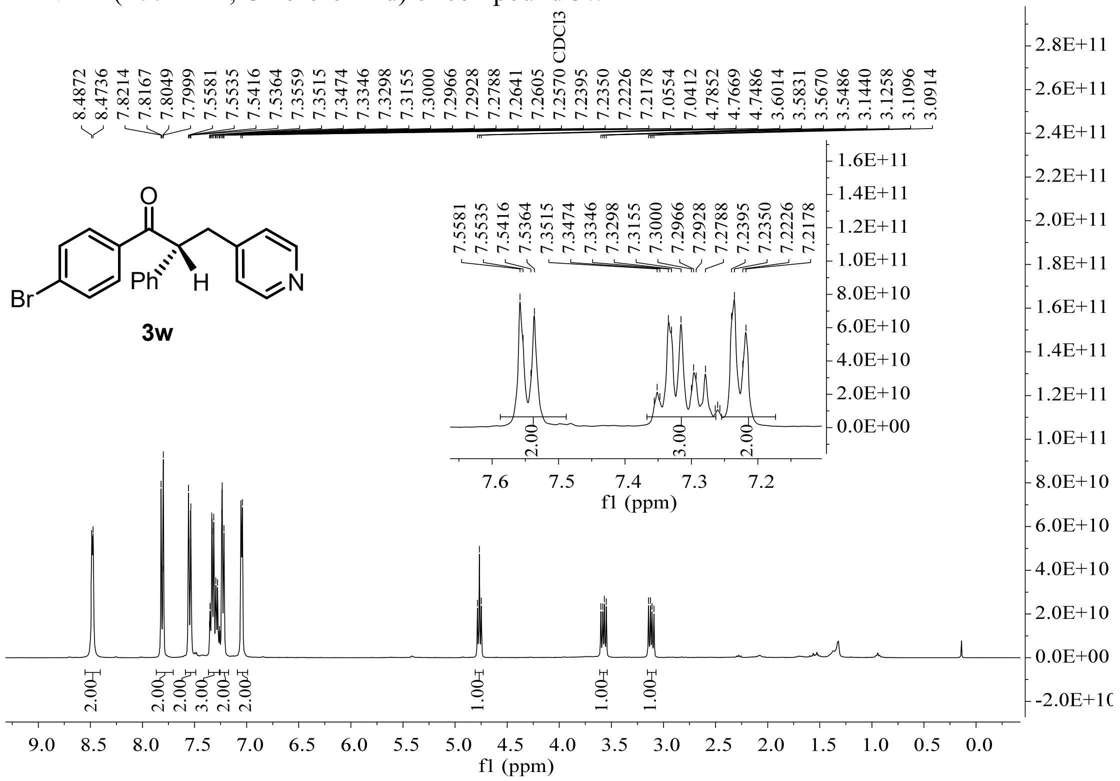

^{13}C NMR (101 MHz, Chloroform-*d*) of compound **3w**

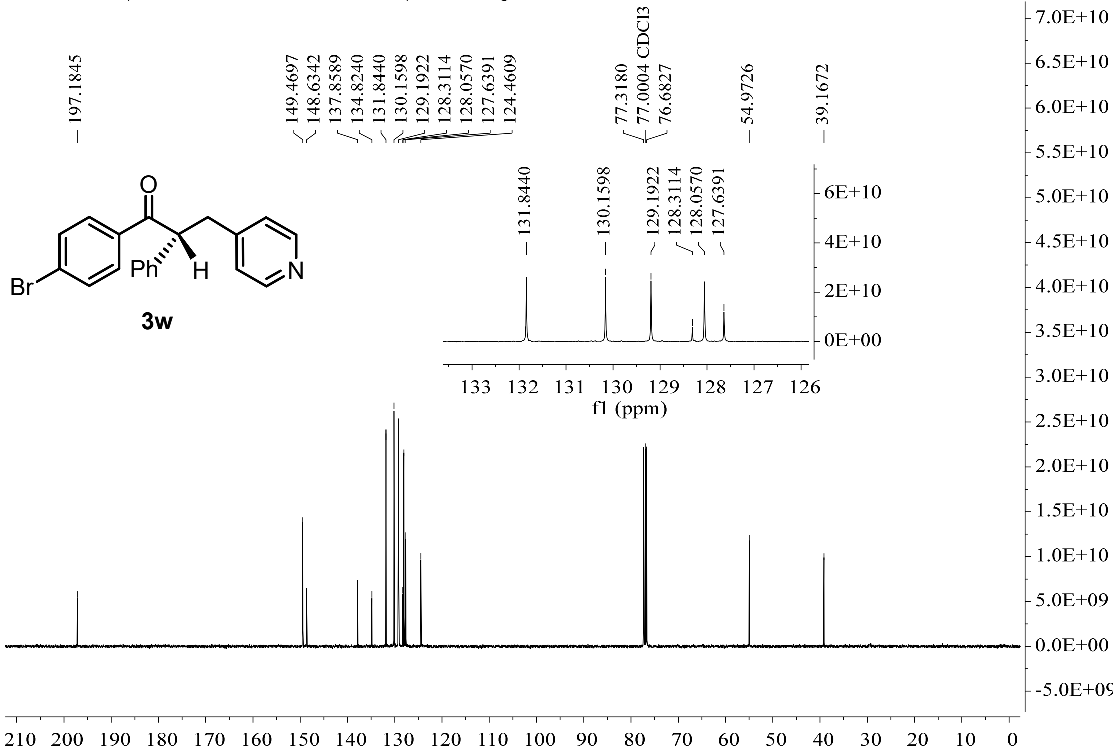

^{1}H NMR (400 MHz, Chloroform-d) of compound **3x**

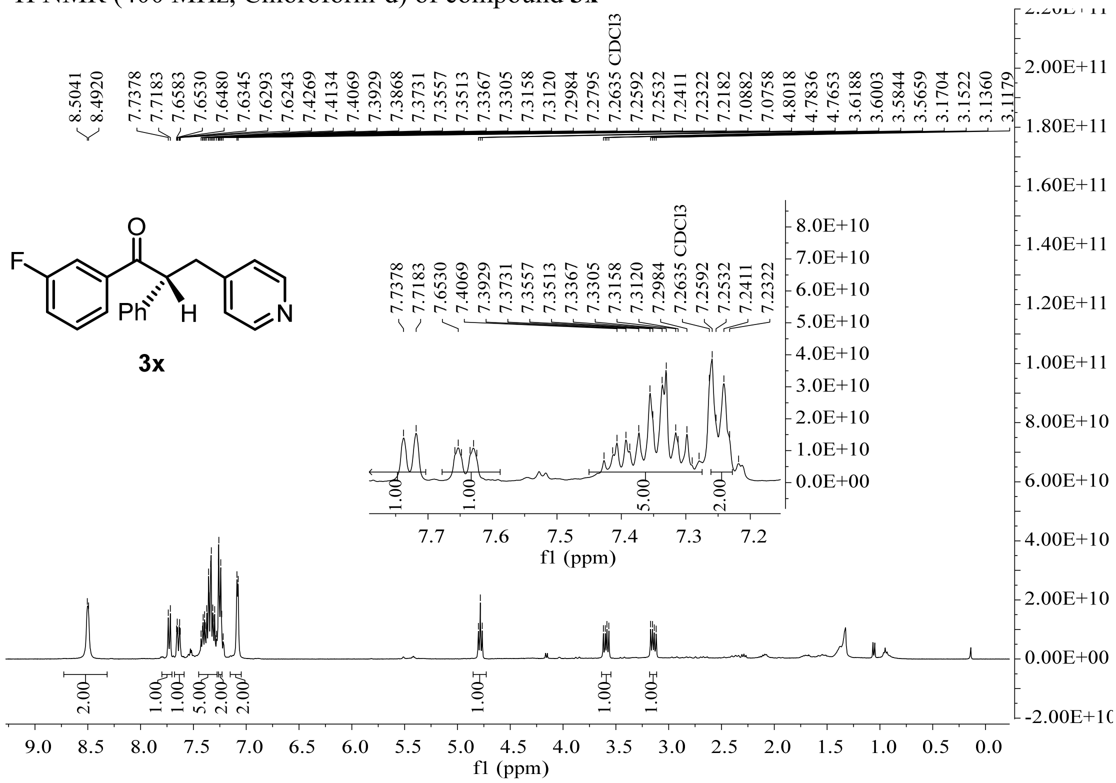

^{13}C NMR (101 MHz, Chloroform-*d*) of compound **3x**

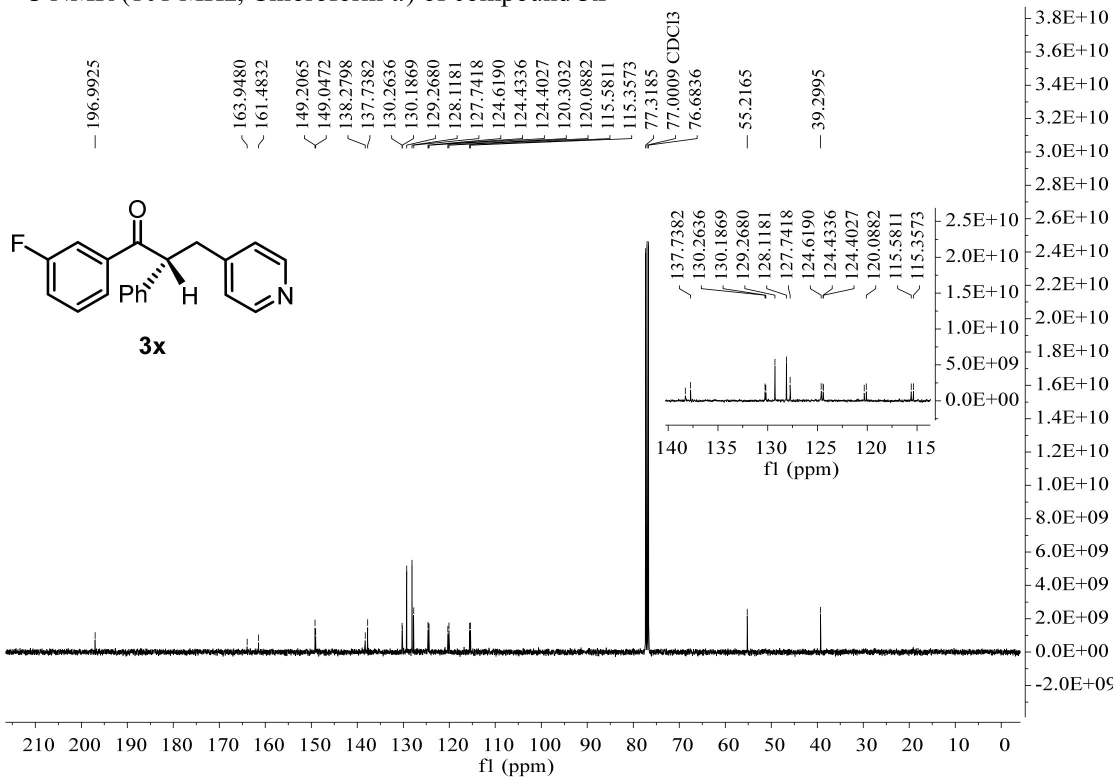

^{19}F NMR (376 MHz, Chloroform-d) of compound **3x**

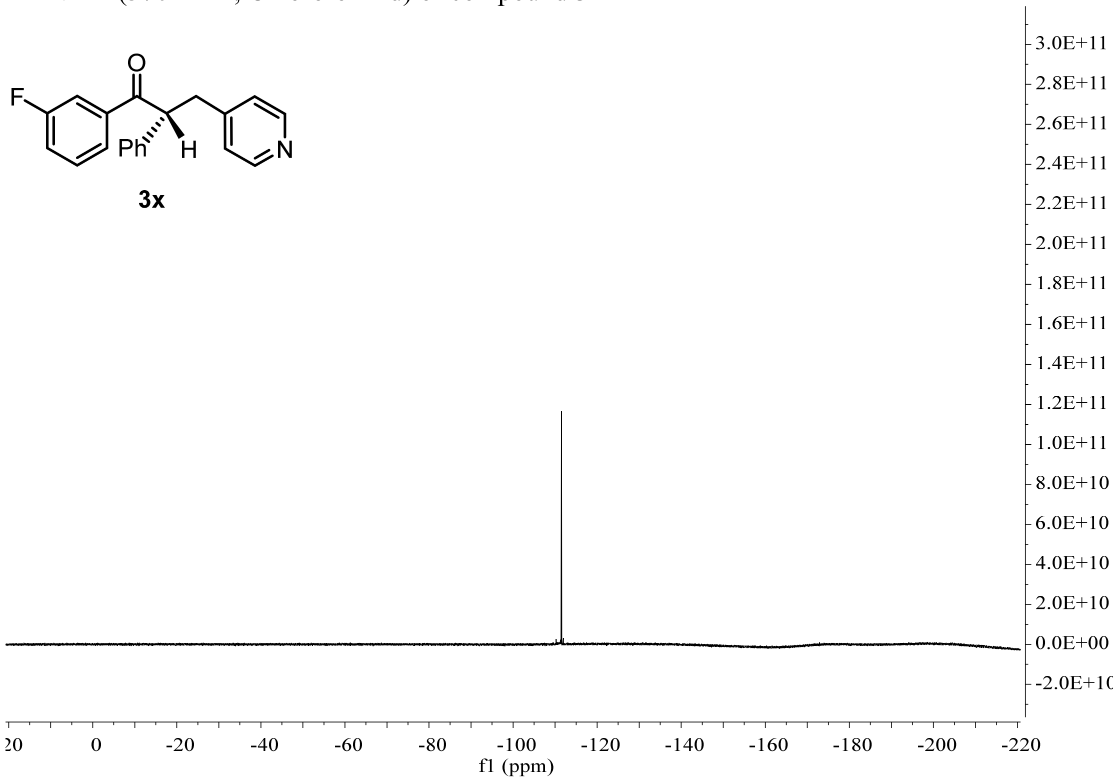

^{1}H NMR (400 MHz, Chloroform-d) of compound **3y**

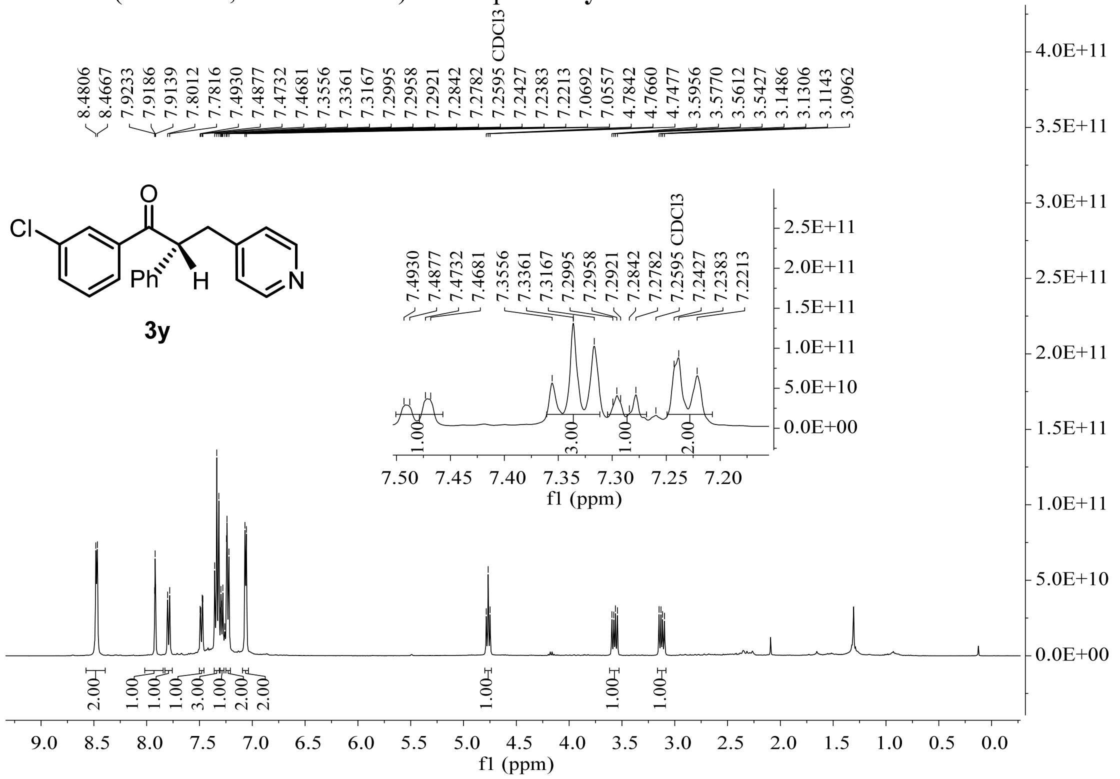

^{13}C NMR (101 MHz, Chloroform-*d*) of compound **3y**

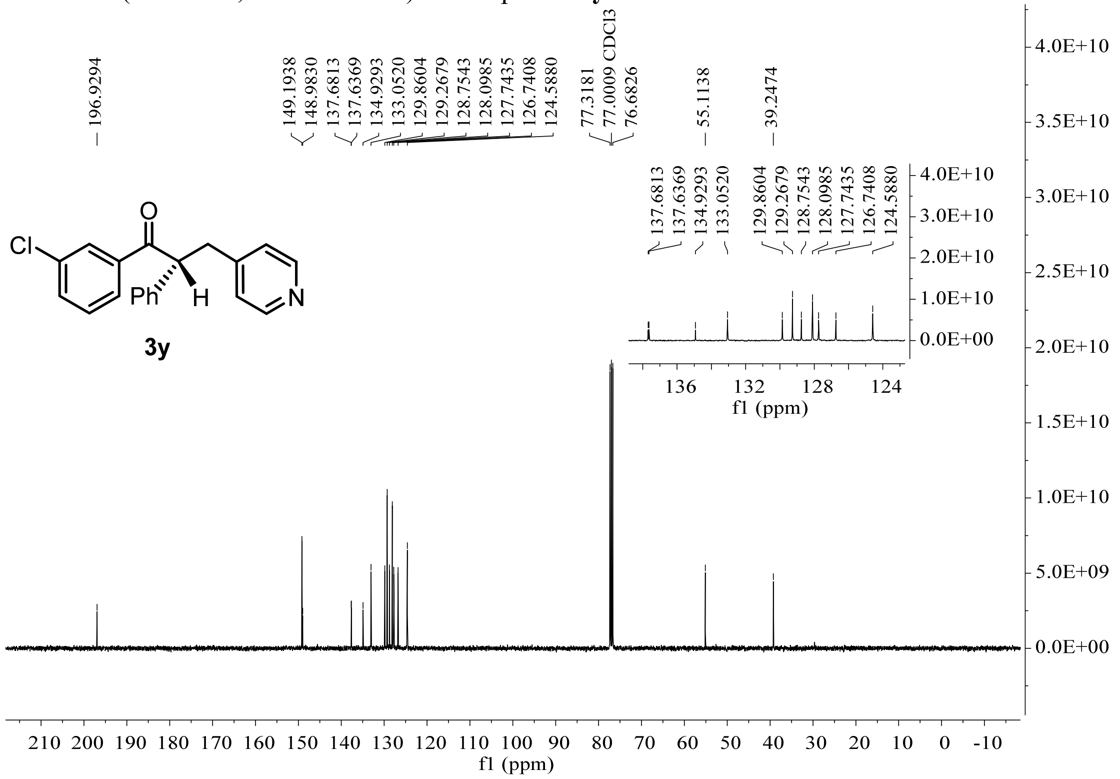

^{1}H NMR (400 MHz, Chloroform-d) of compound **3za**

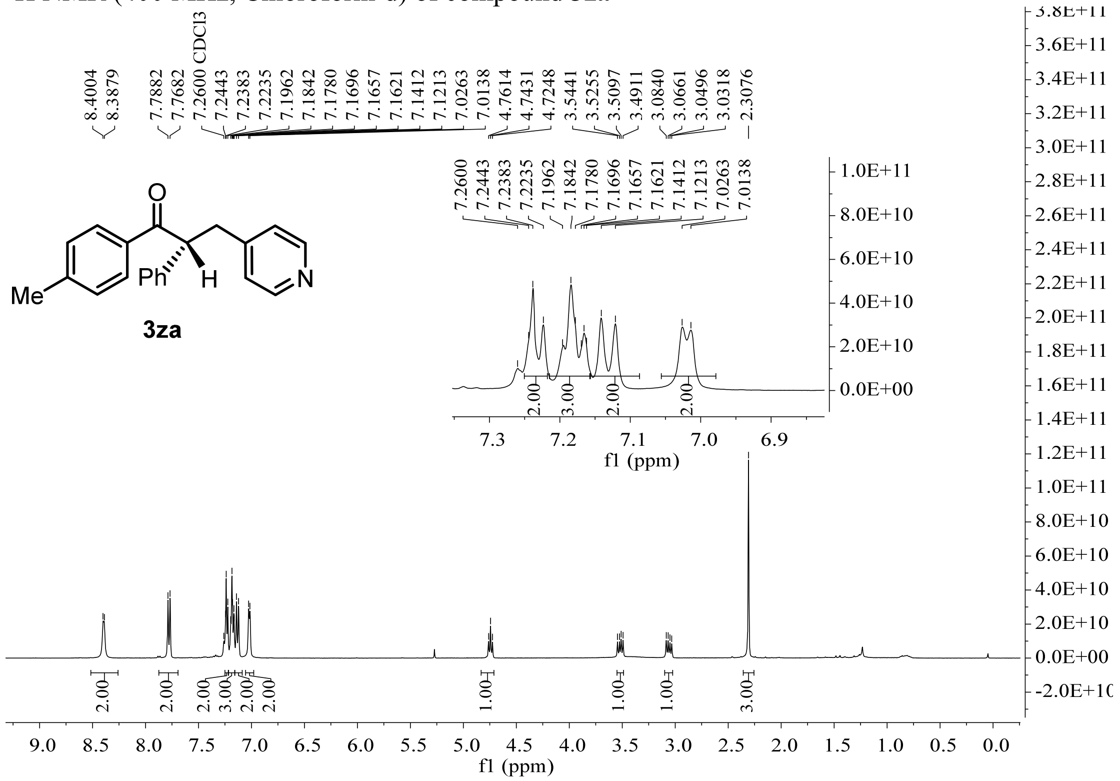

^{13}C NMR (101 MHz, Chloroform-*d*) of compound **3za**

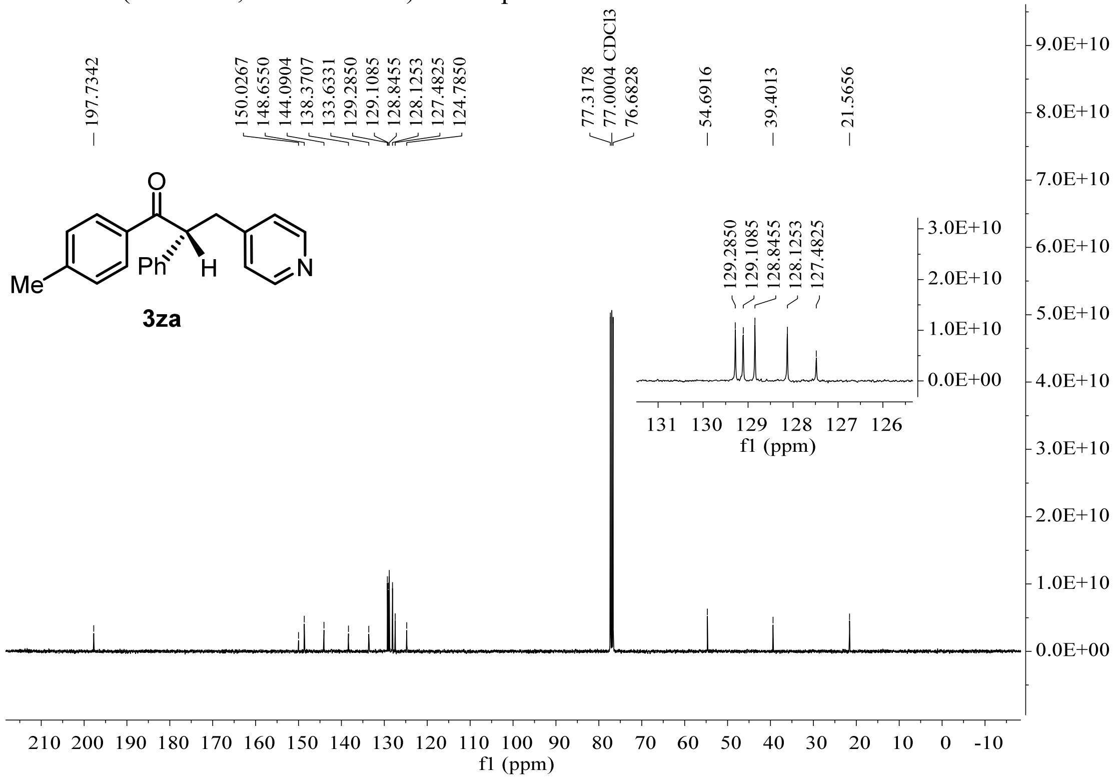

^{1}H NMR (400 MHz, Chloroform-d) of compound **3zb**

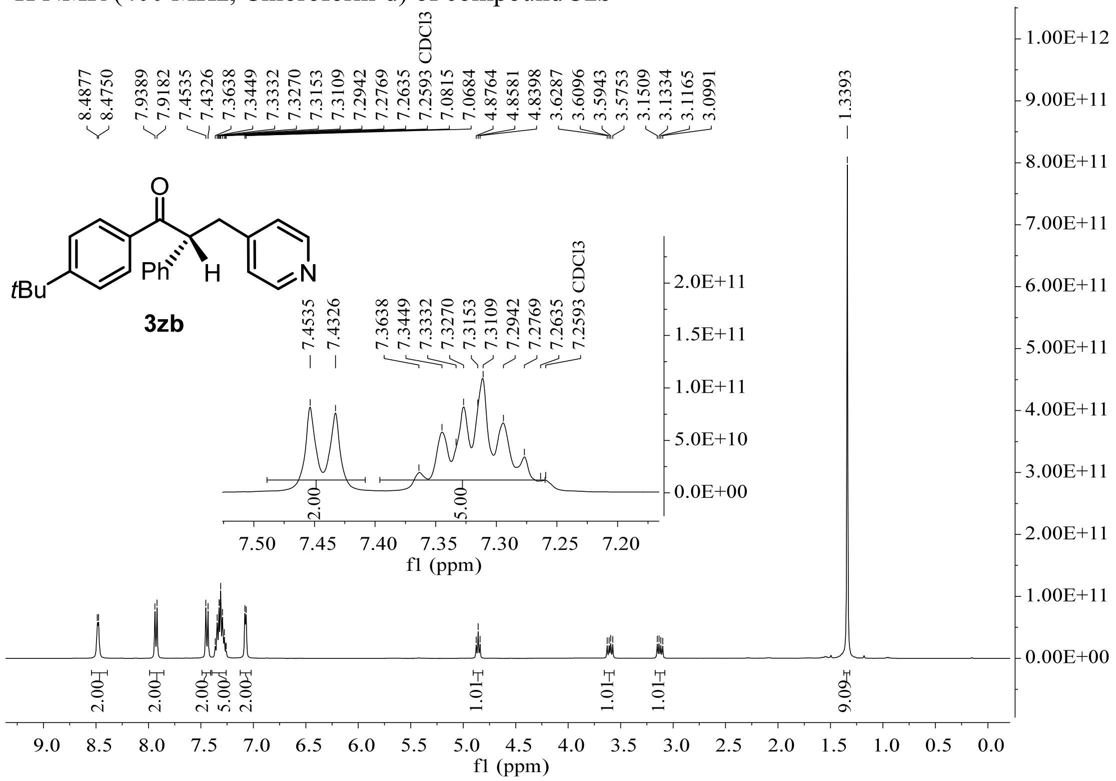

^{13}C NMR (101 MHz, Chloroform-*d*) of compound **3zb**

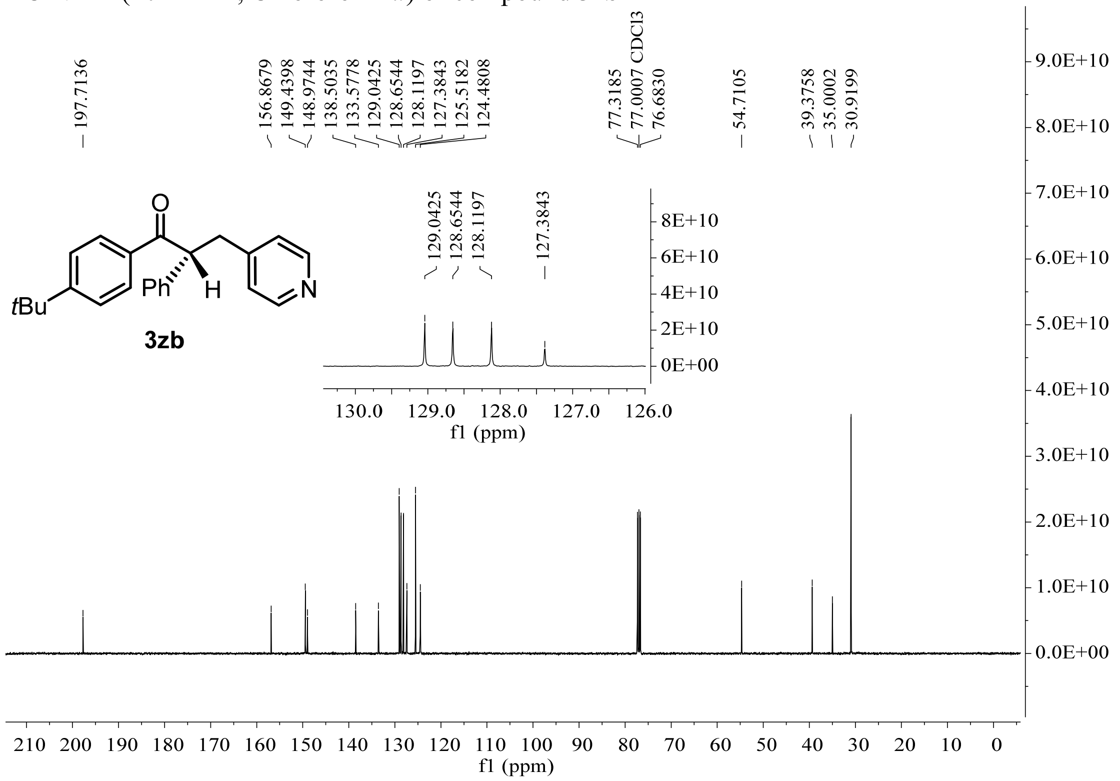

^{1}H NMR (300 MHz, Chloroform-d) of compound **3zc**

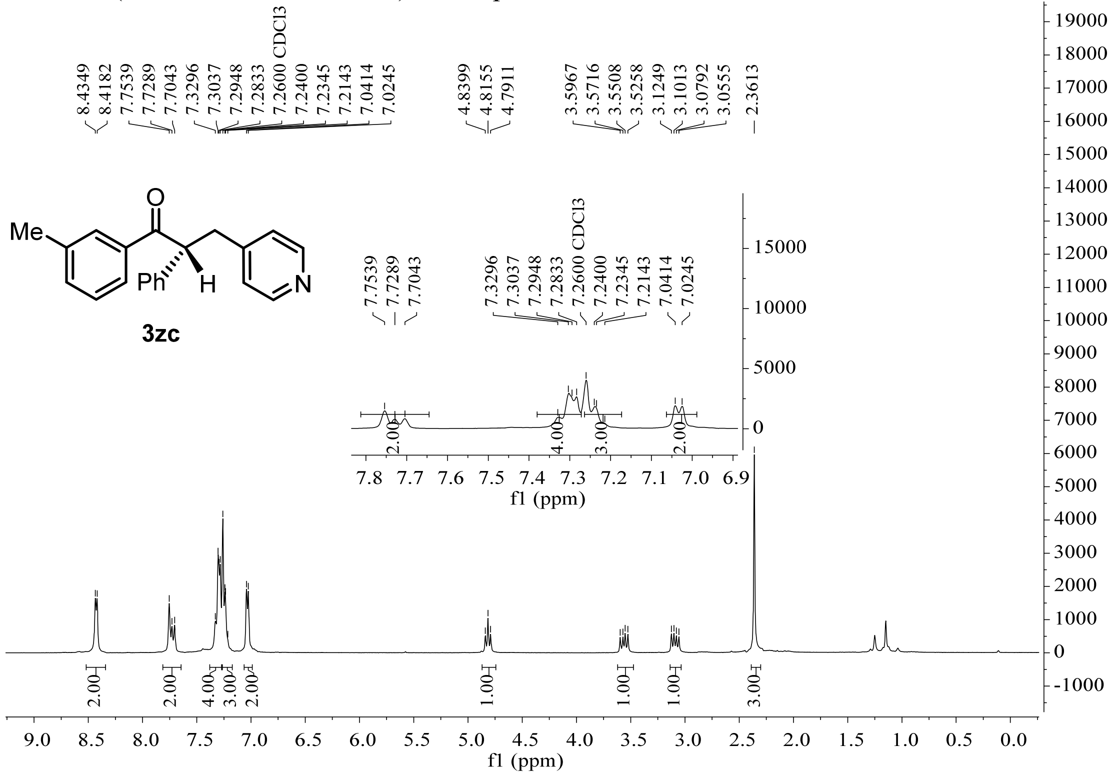

^{13}CNMR (75 MHz, Chloroform-d) of compound **3zc**

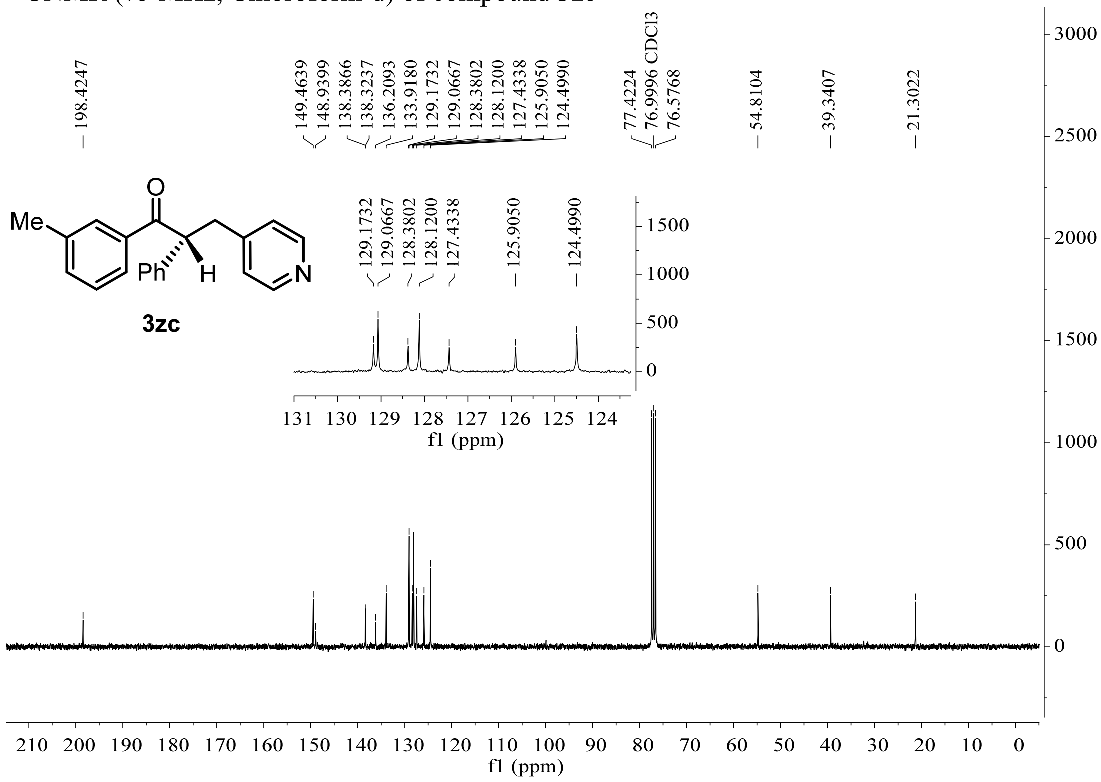

^{1}H NMR (400 MHz, Chloroform-d) of compound **3zd**

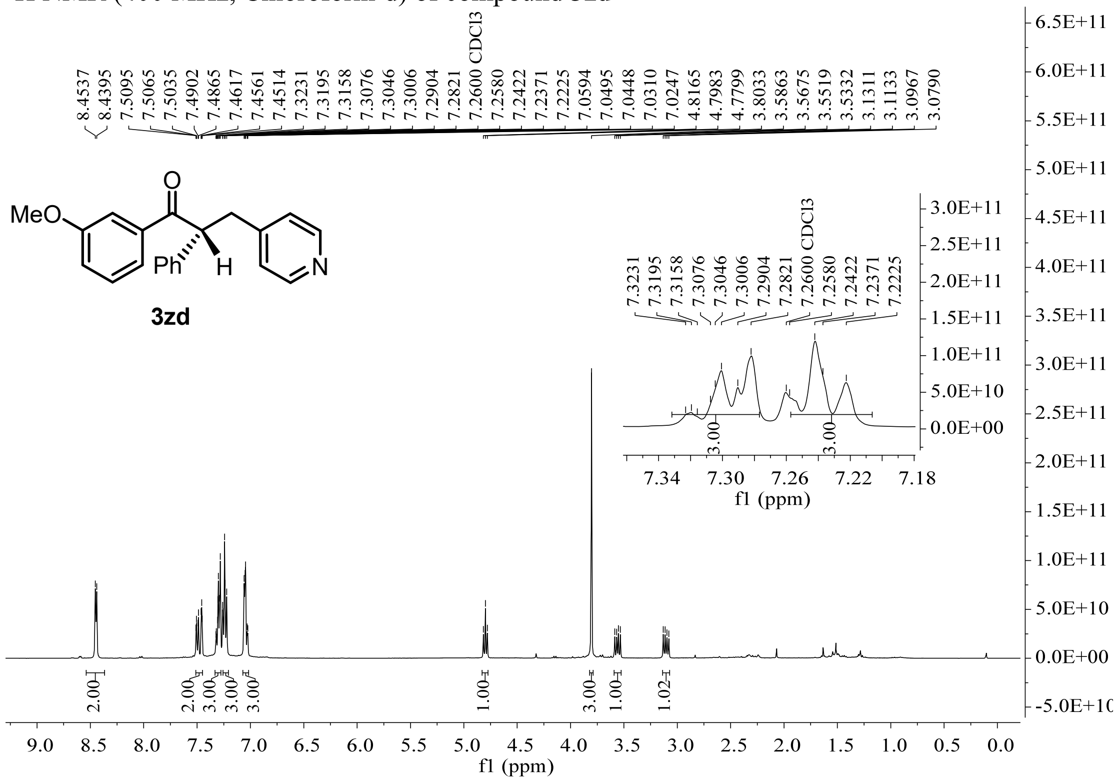

^{13}C NMR (101 MHz, Chloroform-*d*) of compound **3zd**

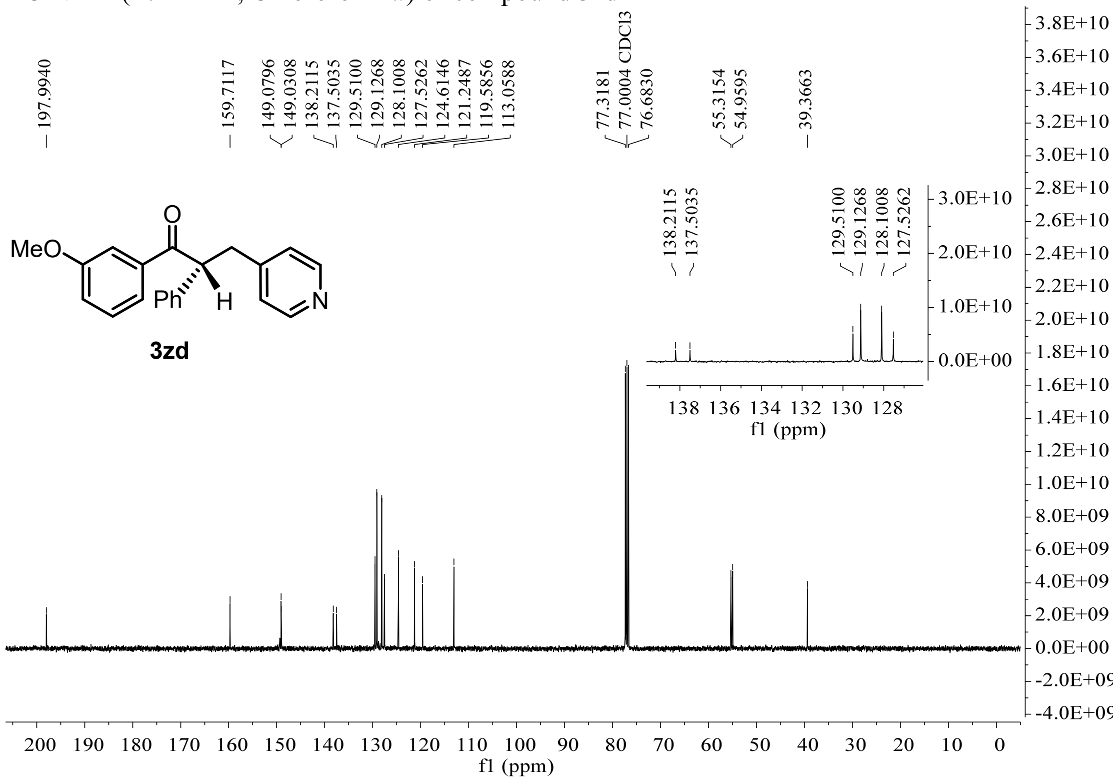

^{1}H NMR (400 MHz, Chloroform-d) of compound **3ze**

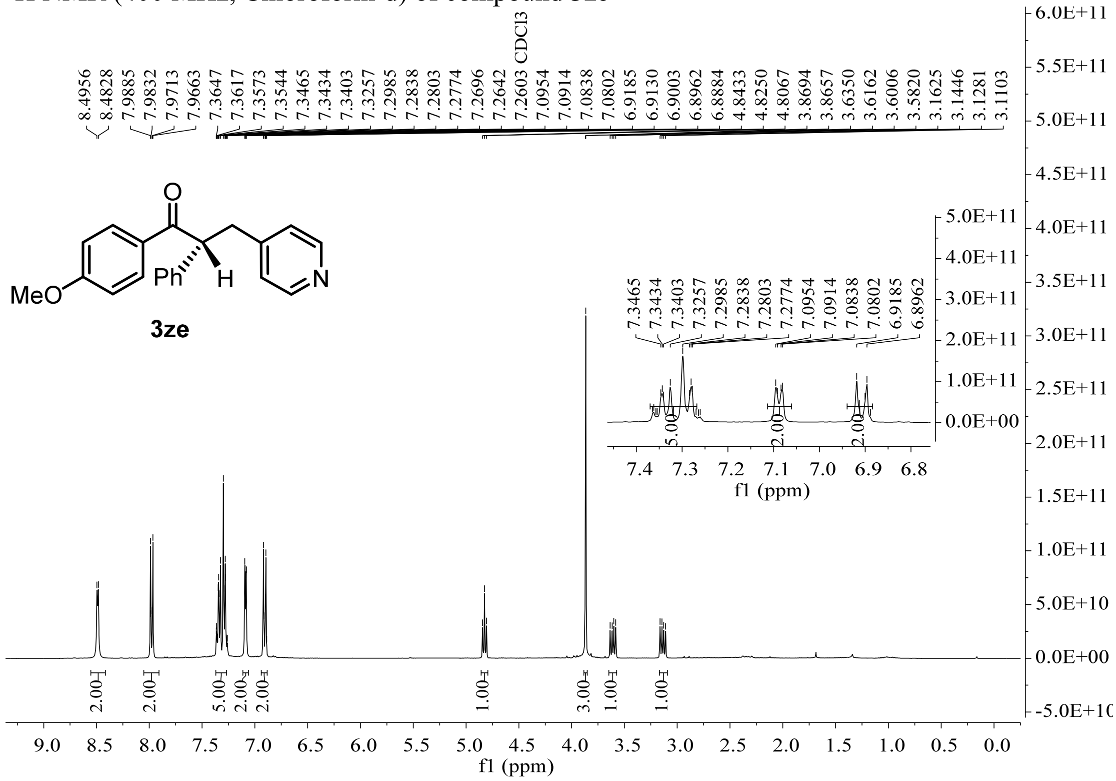

^{13}C NMR (101 MHz, Chloroform-*d*) of compound **3ze**

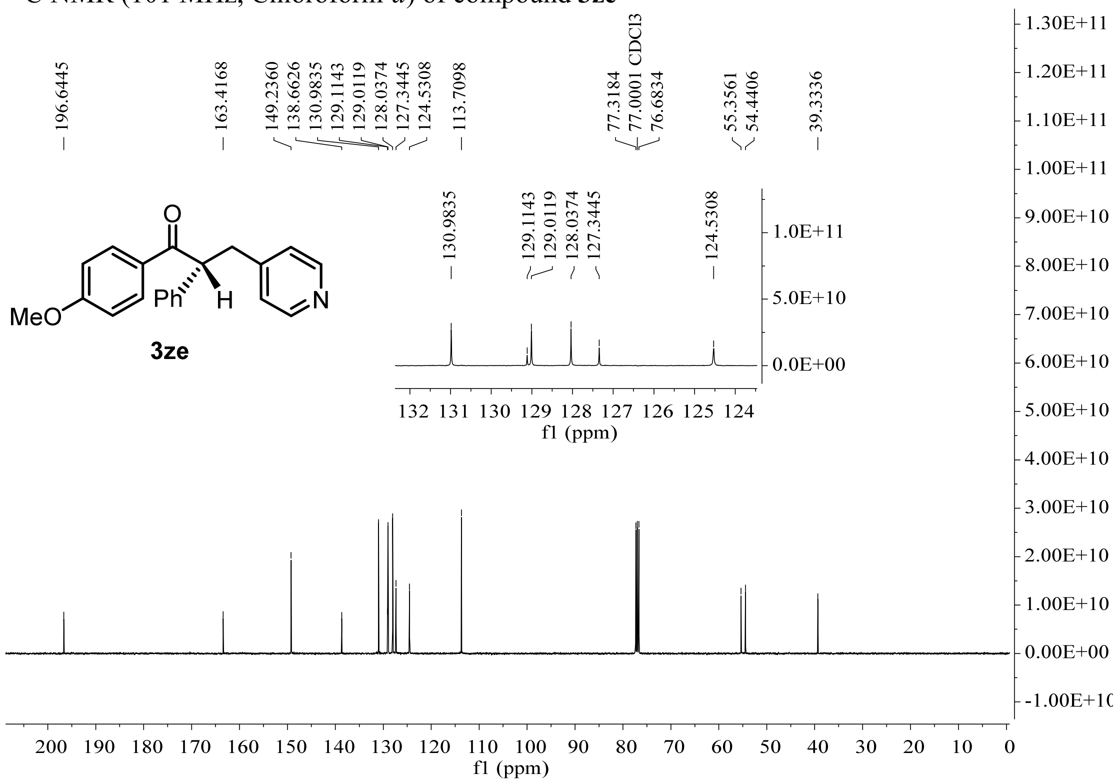

^{1}H NMR (400 MHz, Chloroform-d) of compound **3zf**

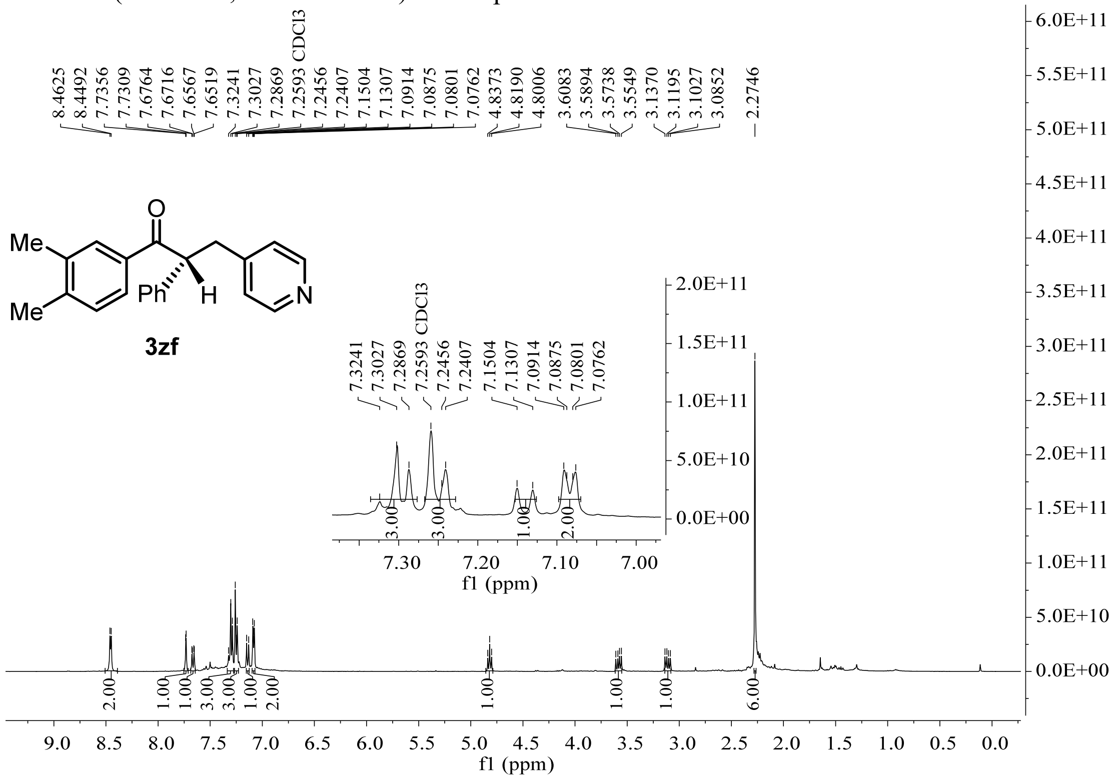

^{13}C NMR (101 MHz, Chloroform-*d*) of compound **3zf**

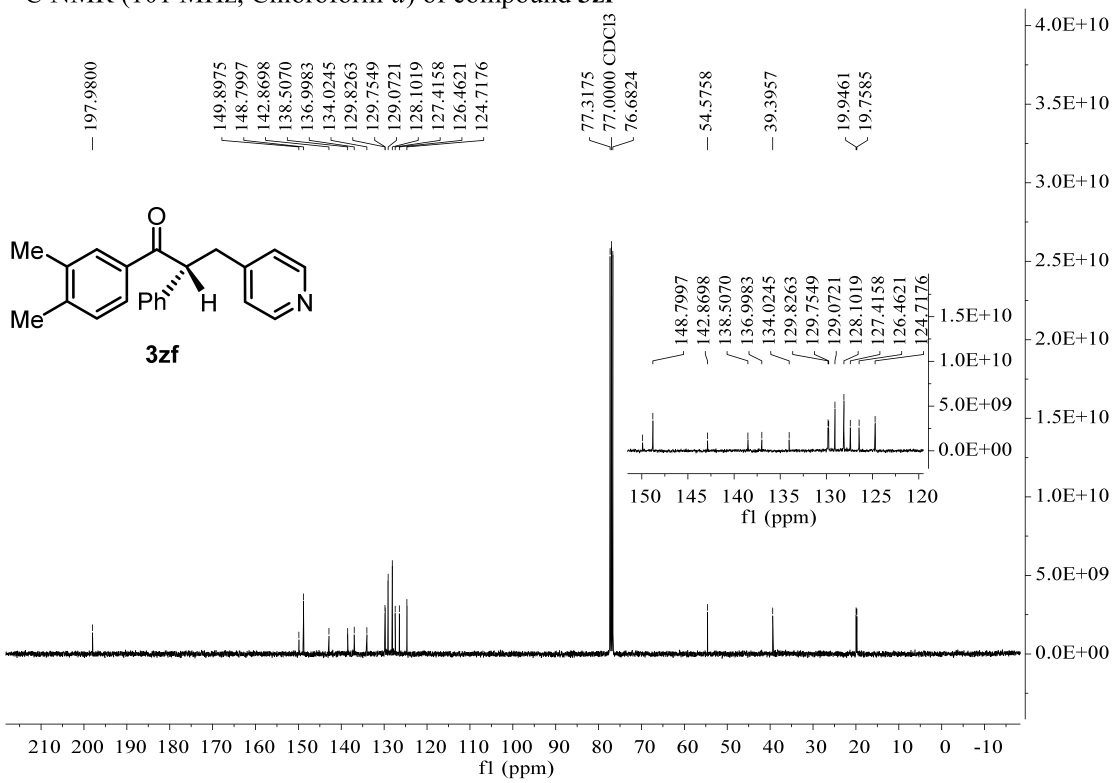

^{1}H NMR (400 MHz, Chloroform-d) of compound **3zg**

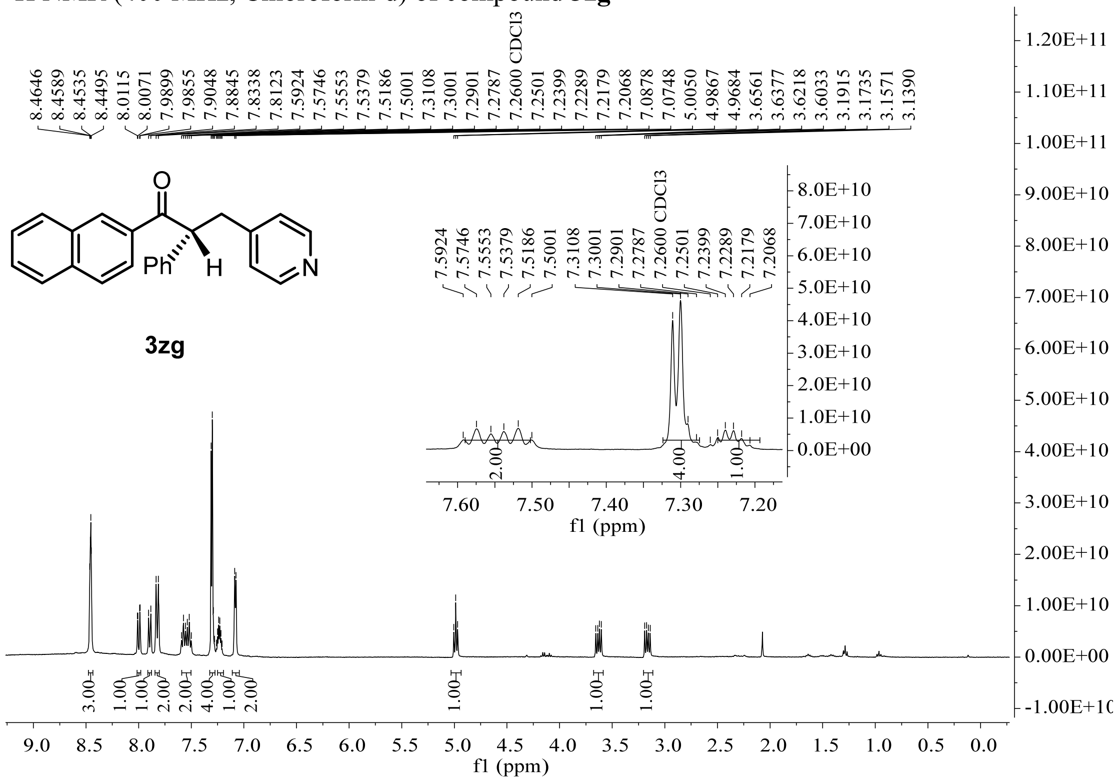

^{13}C NMR (101 MHz, Chloroform-*d*) of compound **3zg**

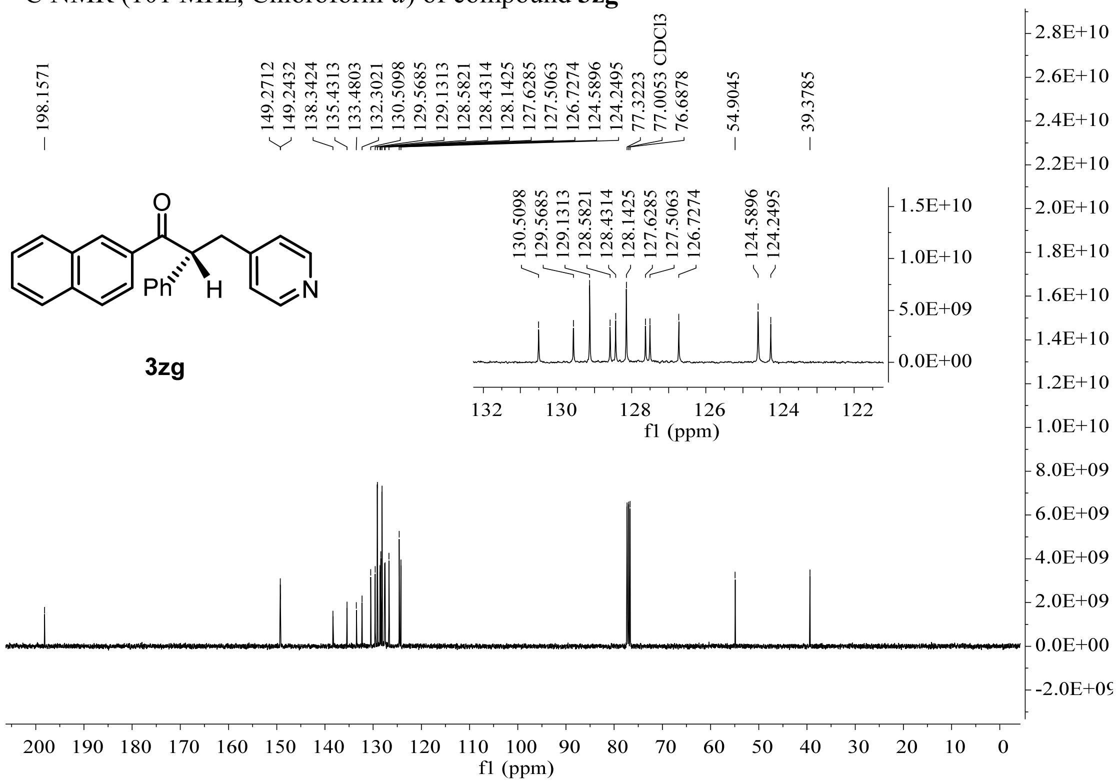

^{1}H NMR (400 MHz, Chloroform-d) of compound **3zh**

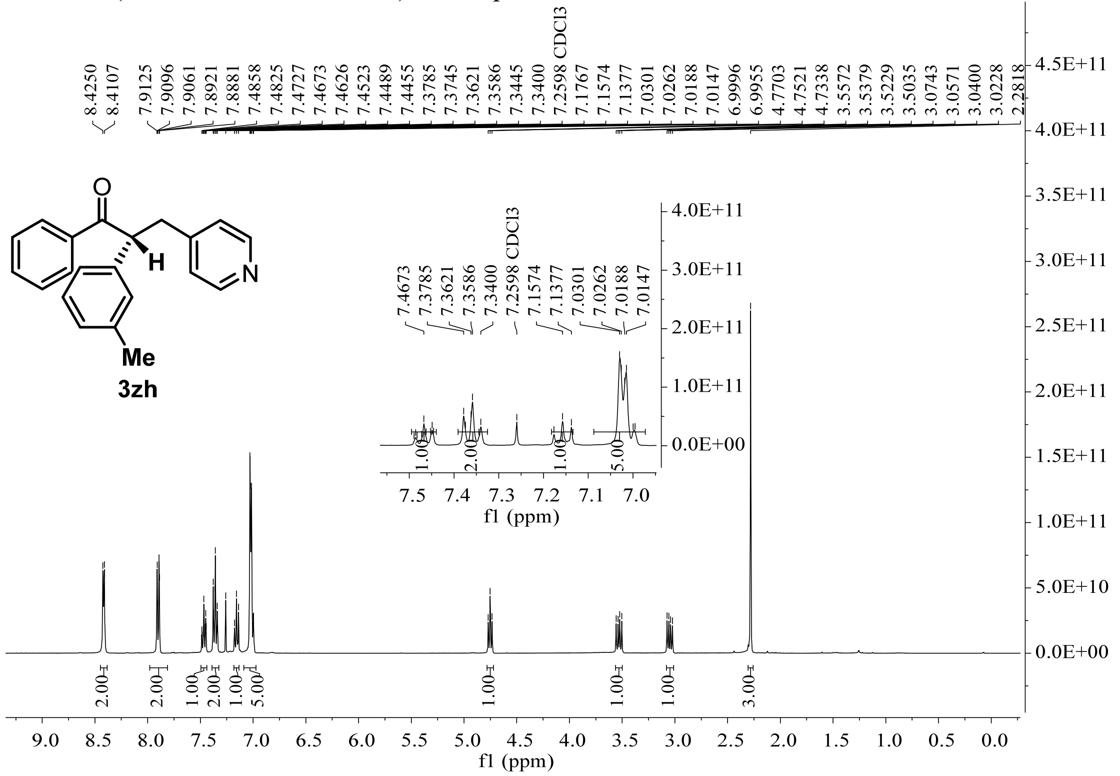

^{13}C NMR (101 MHz, Chloroform-*d*) of compound **3zh**

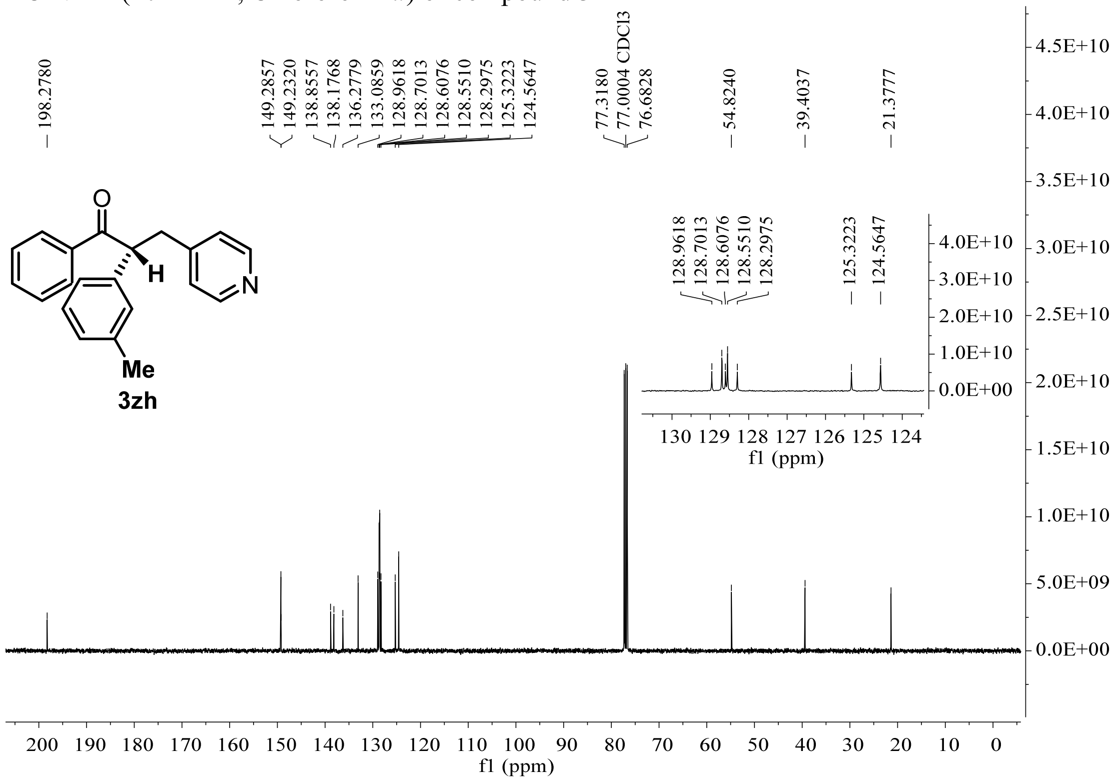

^{1}H NMR (400 MHz, Chloroform-d) of compound **3zi**

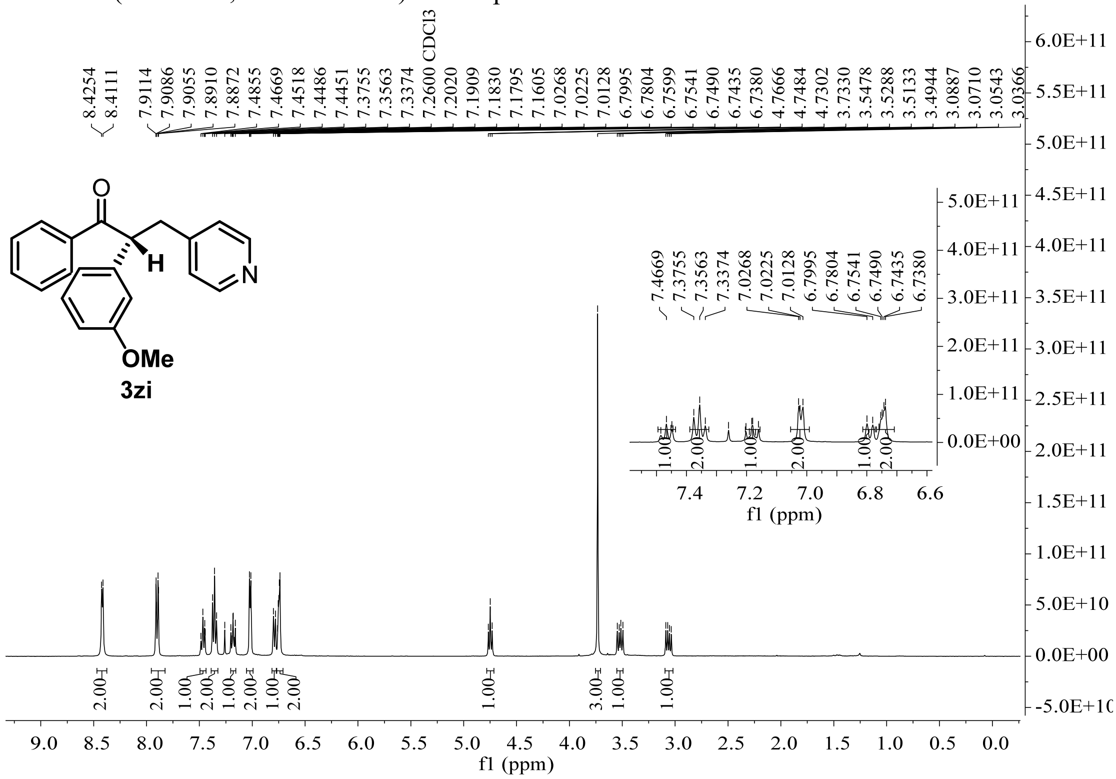

^{13}C NMR (101 MHz, Chloroform-*d*) of compound **3zi**

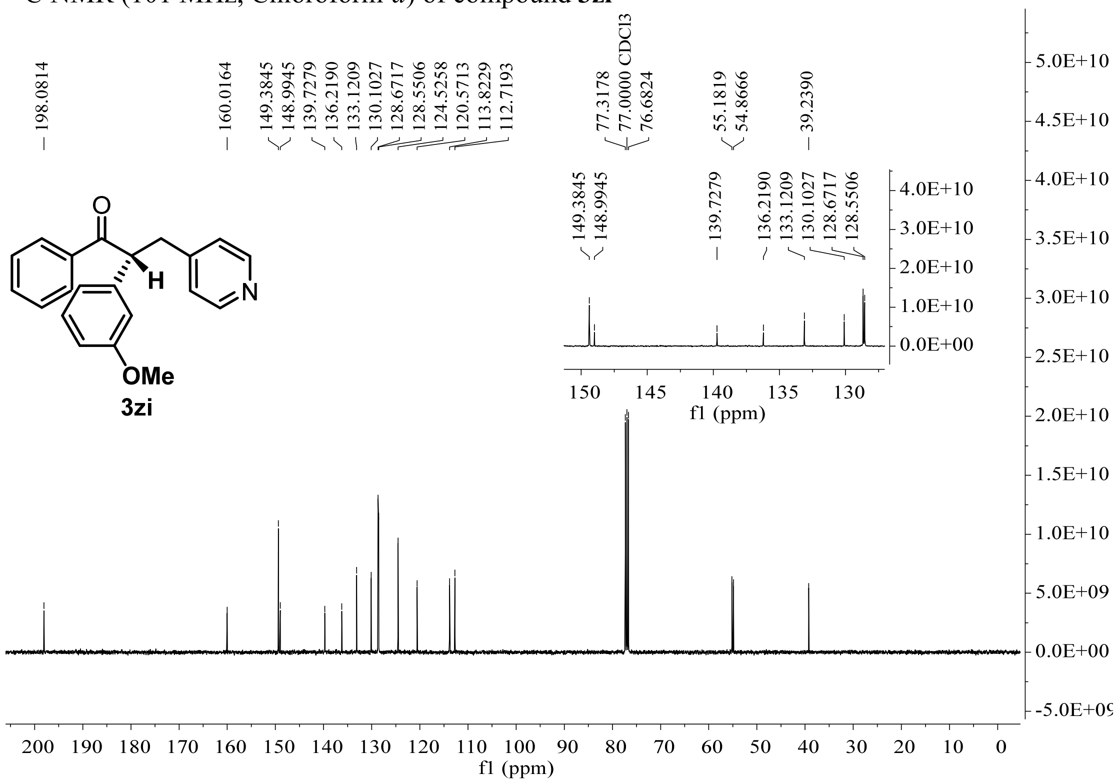

^{1}H NMR (400 MHz, Chloroform-d) of compound **3zj**

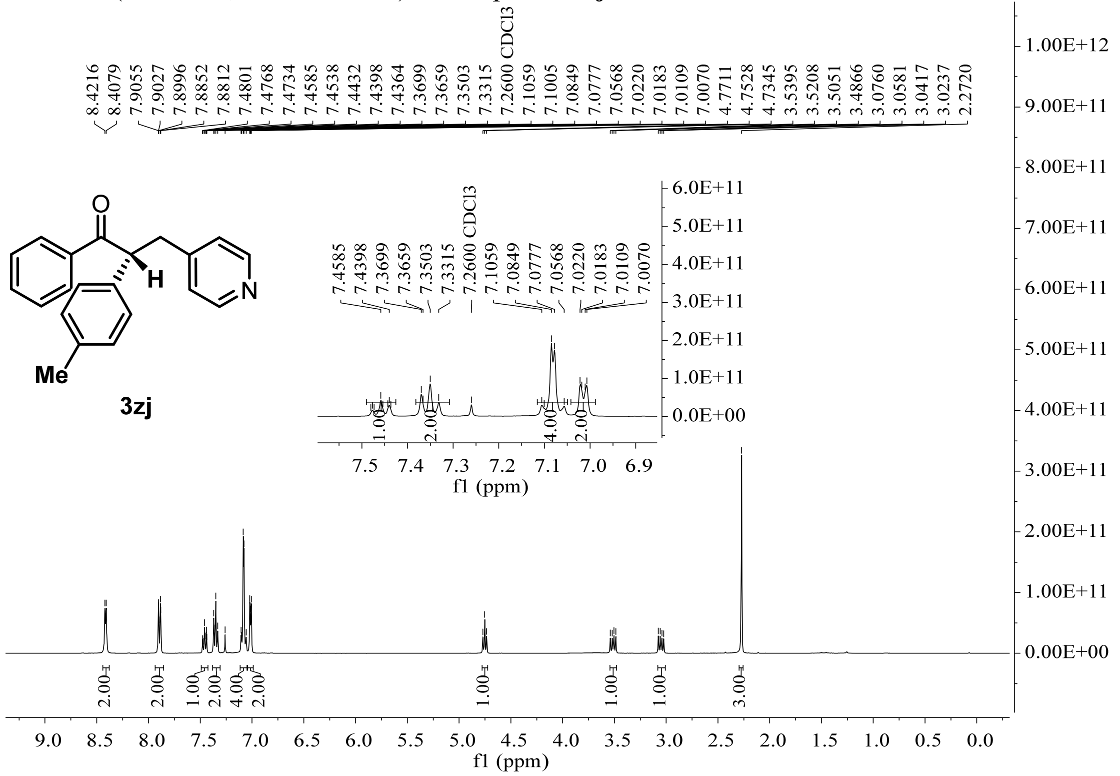

^{13}C NMR (101 MHz, Chloroform-*d*) of compound **3zj**

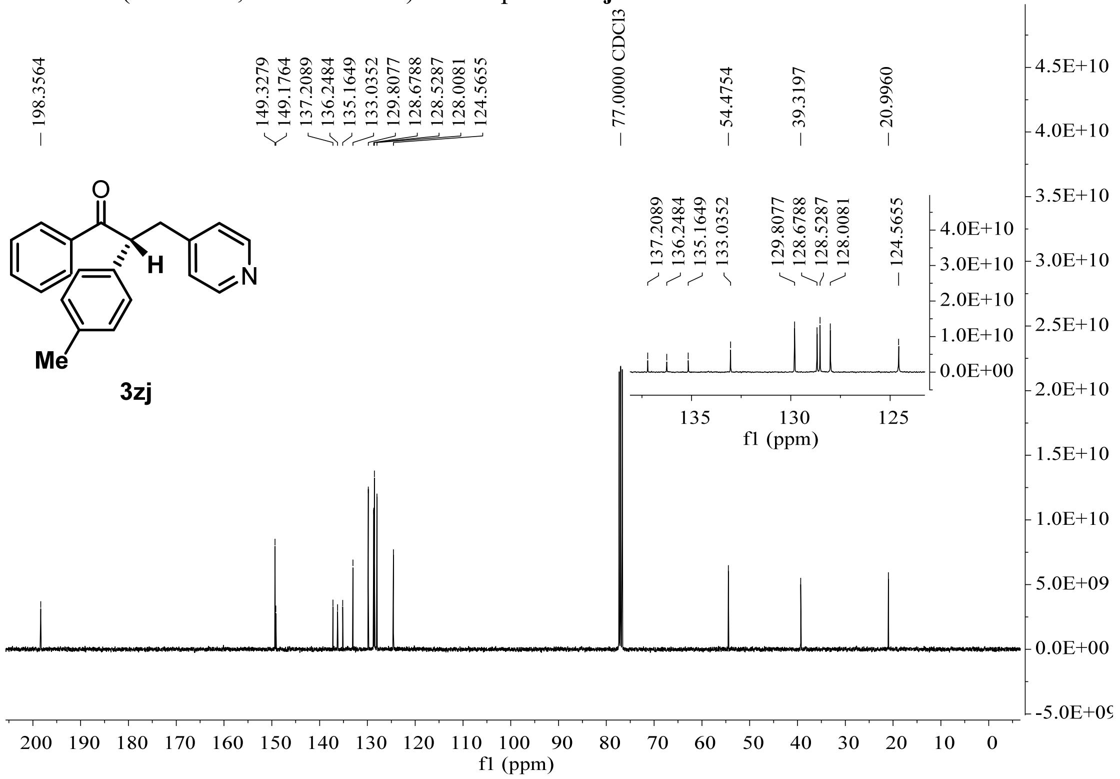

^{1}H NMR (300 MHz, Chloroform-d) of compound **6a**

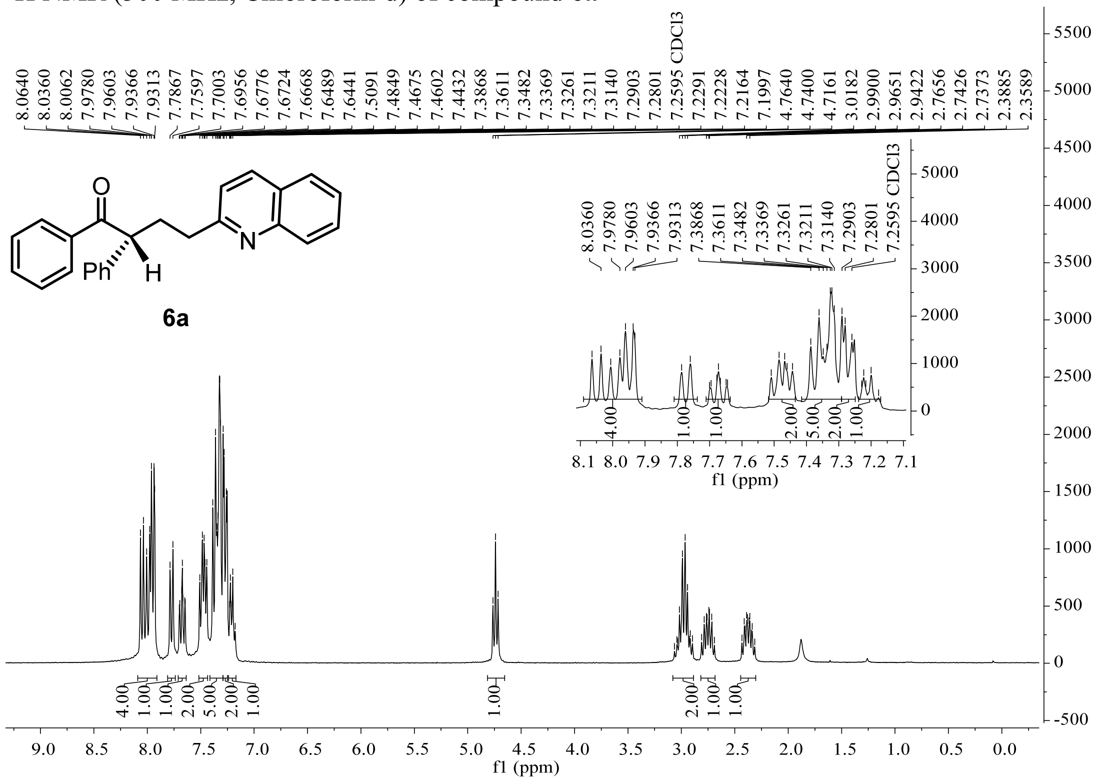

^{13}C NMR (75 MHz, Chloroform-*d*) of compound **6a**

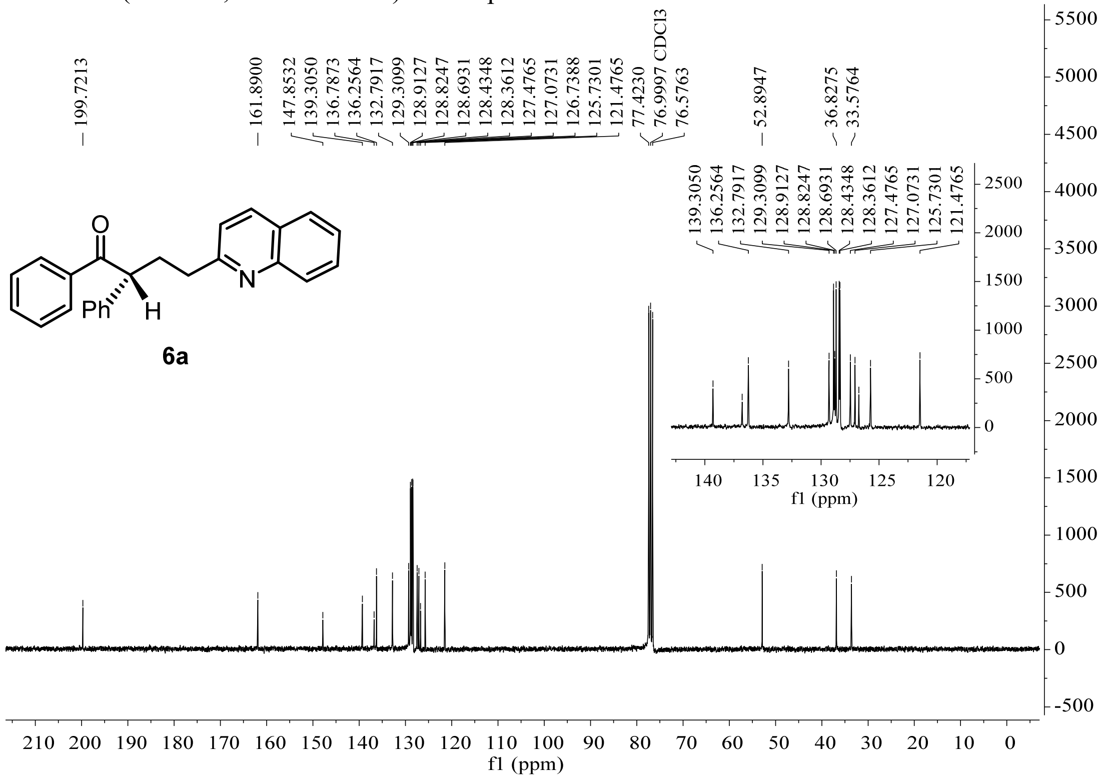

^{1}H NMR (300 MHz, Chloroform-d) of compound **6b**

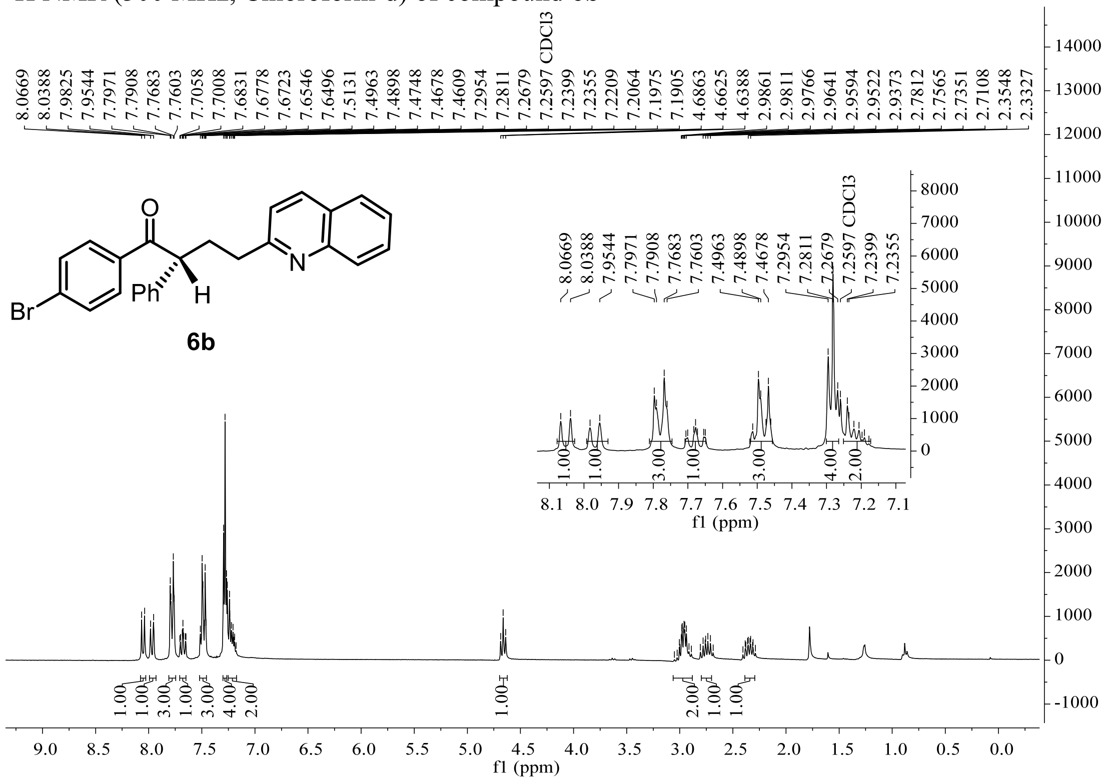

^{13}C NMR (75 MHz, Chloroform-*d*) of compound **6b**

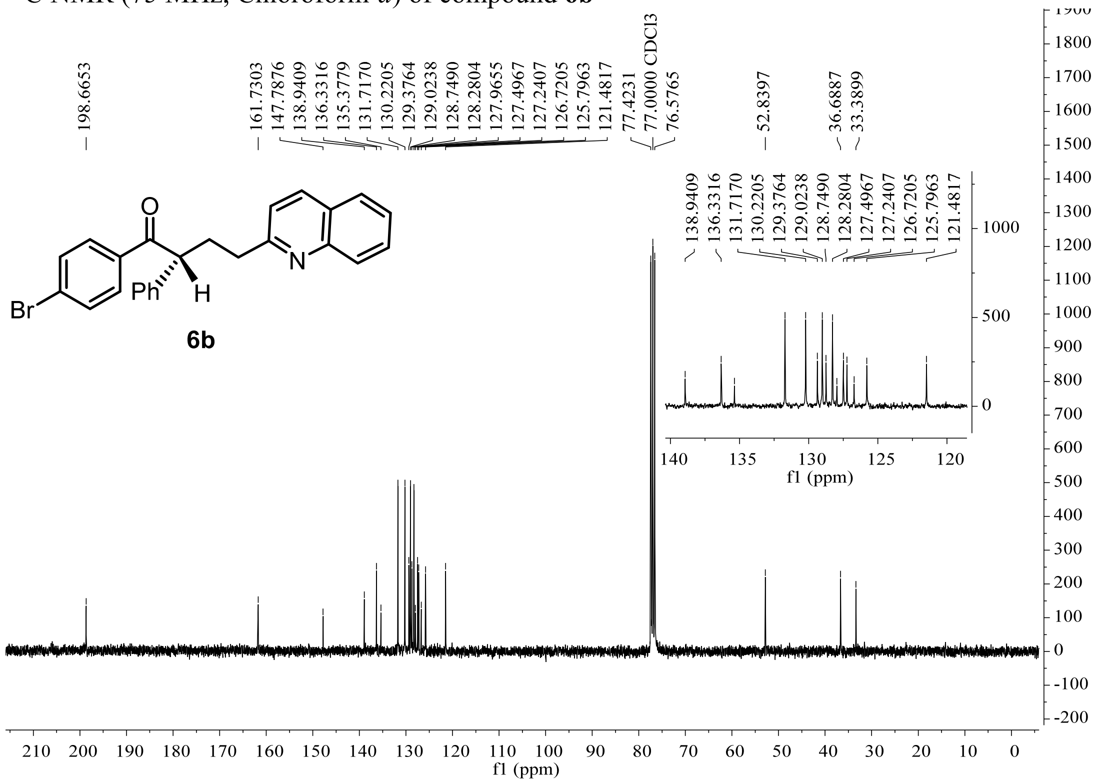

^{1}H NMR (300 MHz, Chloroform-d) of compound **6c**

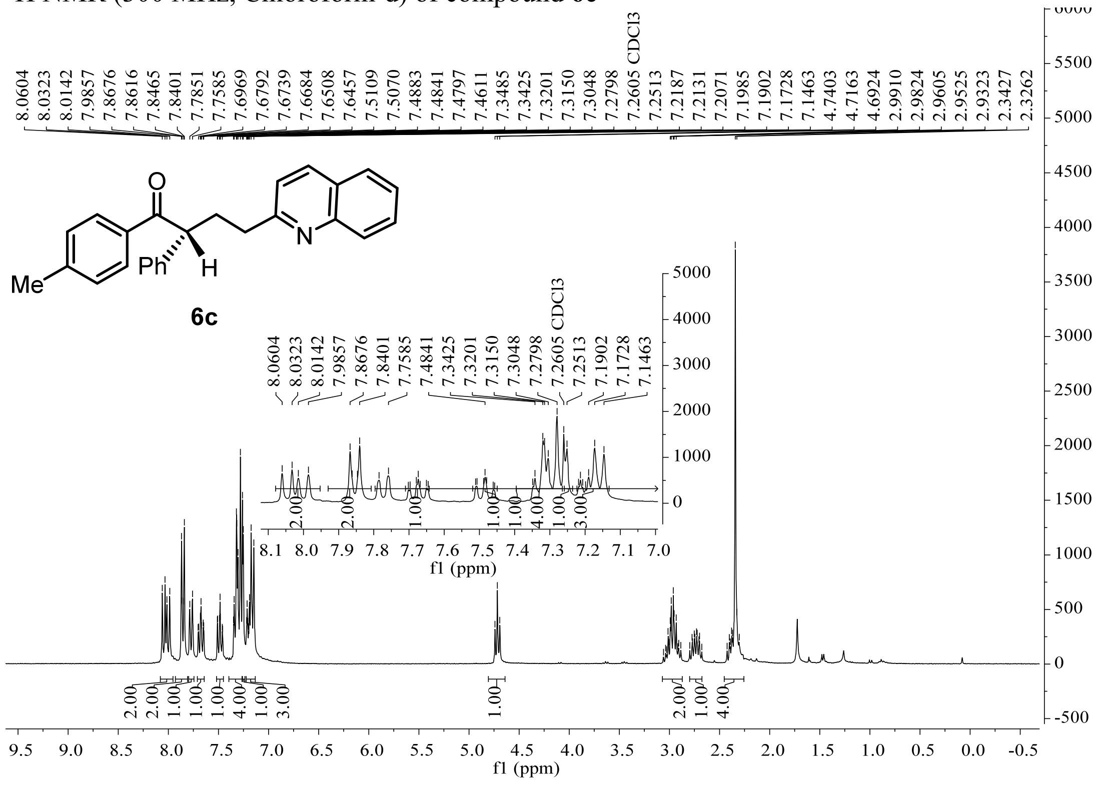

^{13}C NMR (75 MHz, Chloroform-*d*) of compound **6c**

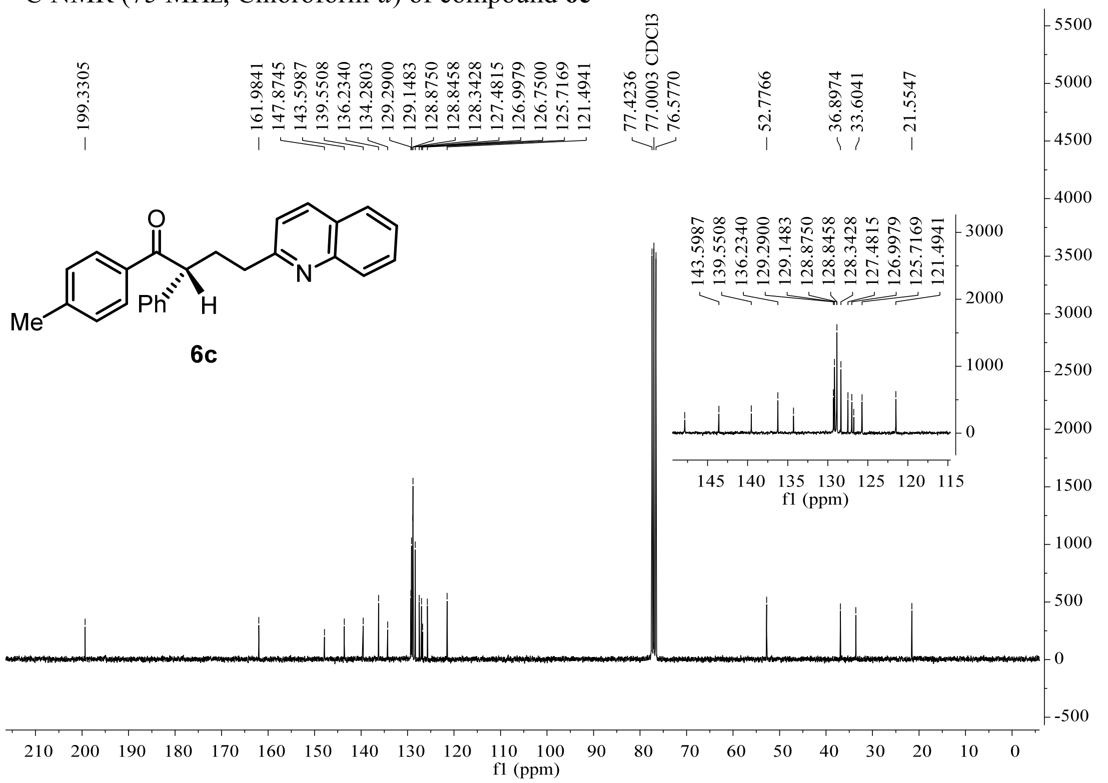

^{1}H NMR (300 MHz, Chloroform-d) of compound **6d**

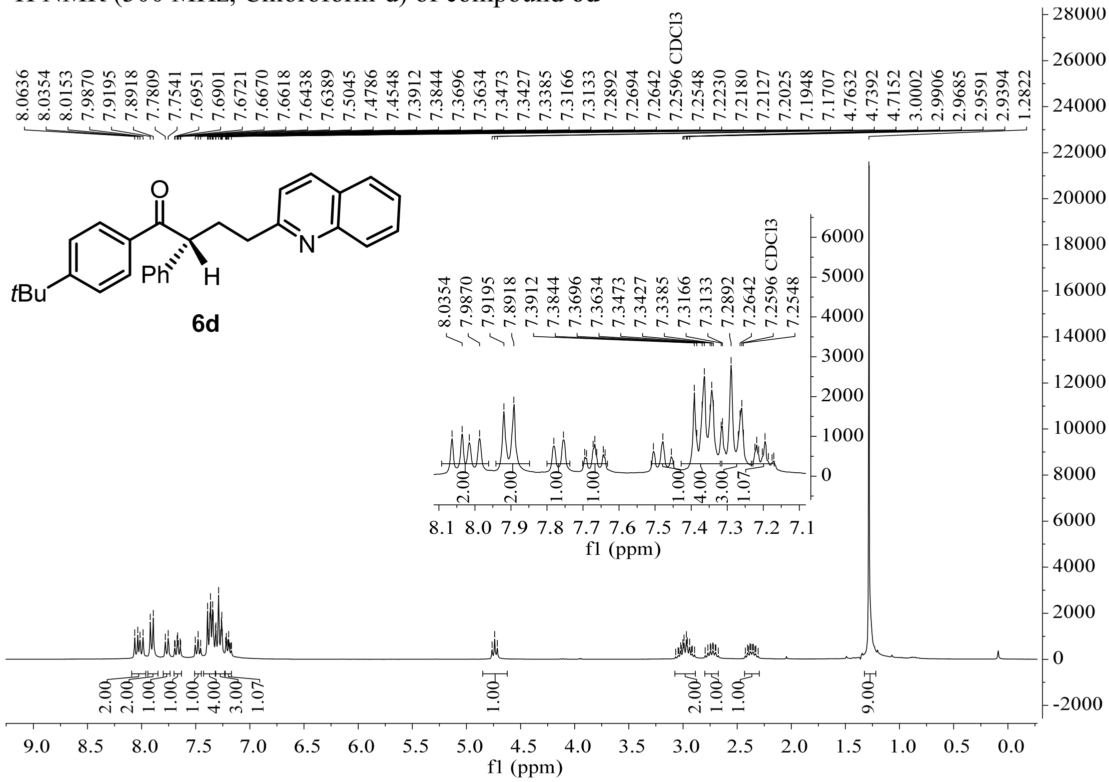

^{13}C NMR (75 MHz, Chloroform-*d*) of compound **6d**

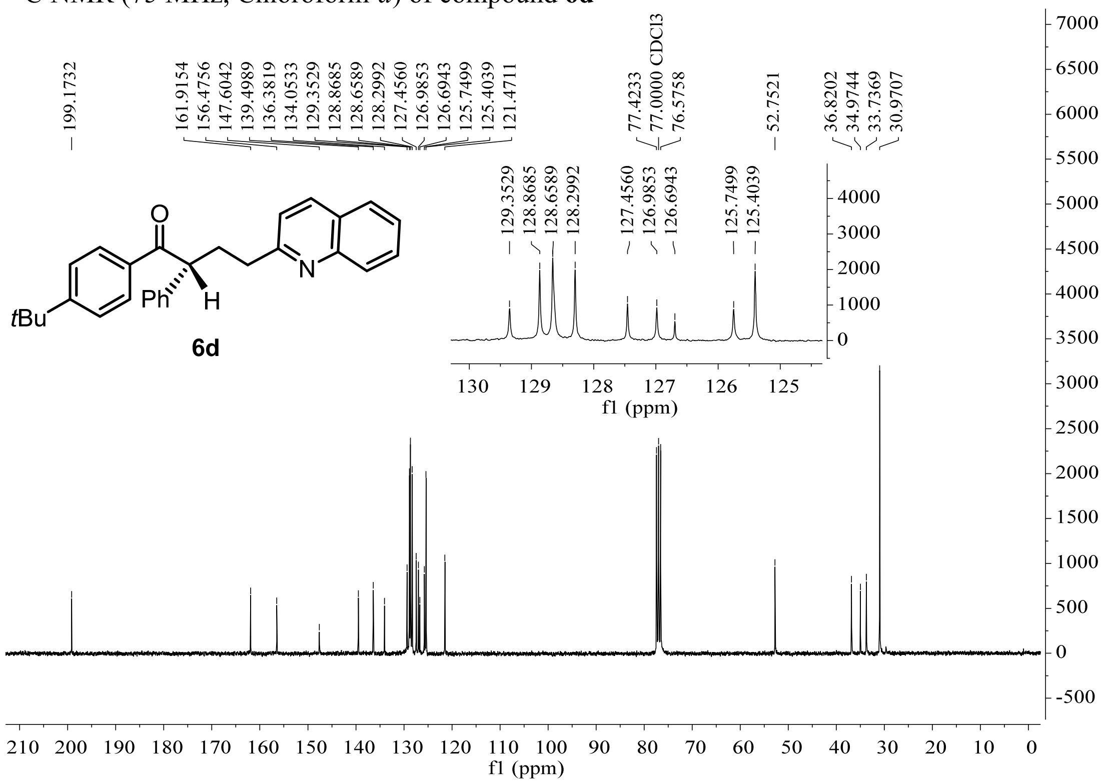

^{1}H NMR (300 MHz, Chloroform-d) of compound **6e**

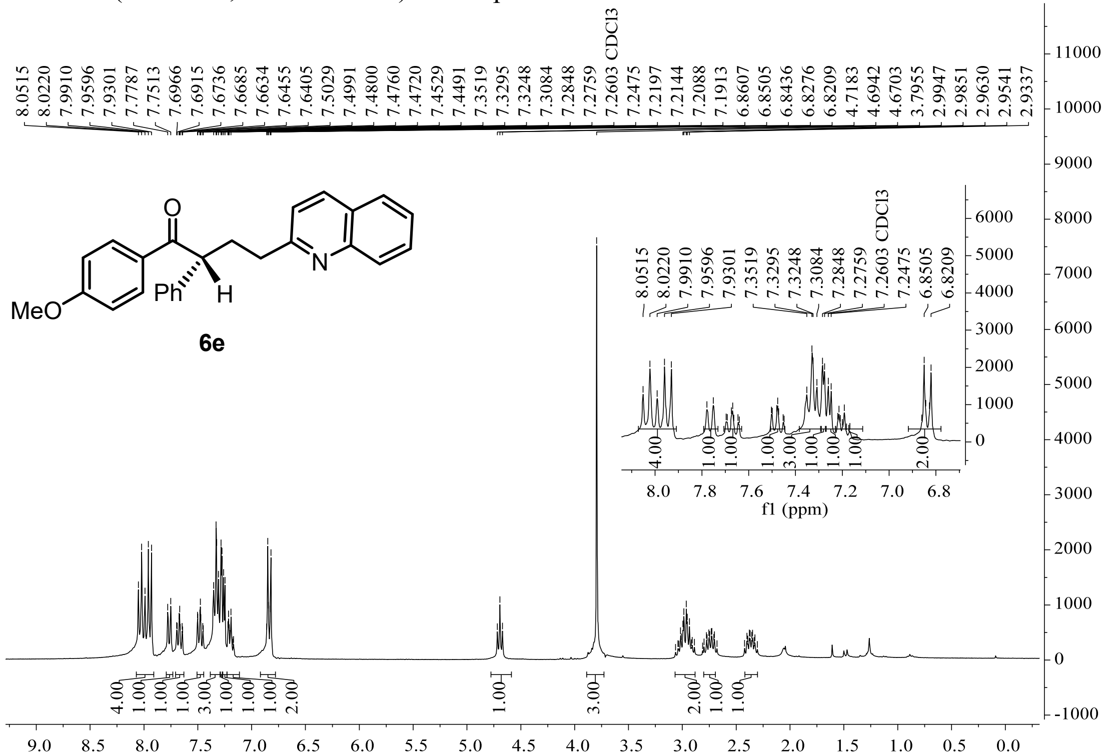

^{13}C NMR (75 MHz, Chloroform-*d*) of compound **6e**

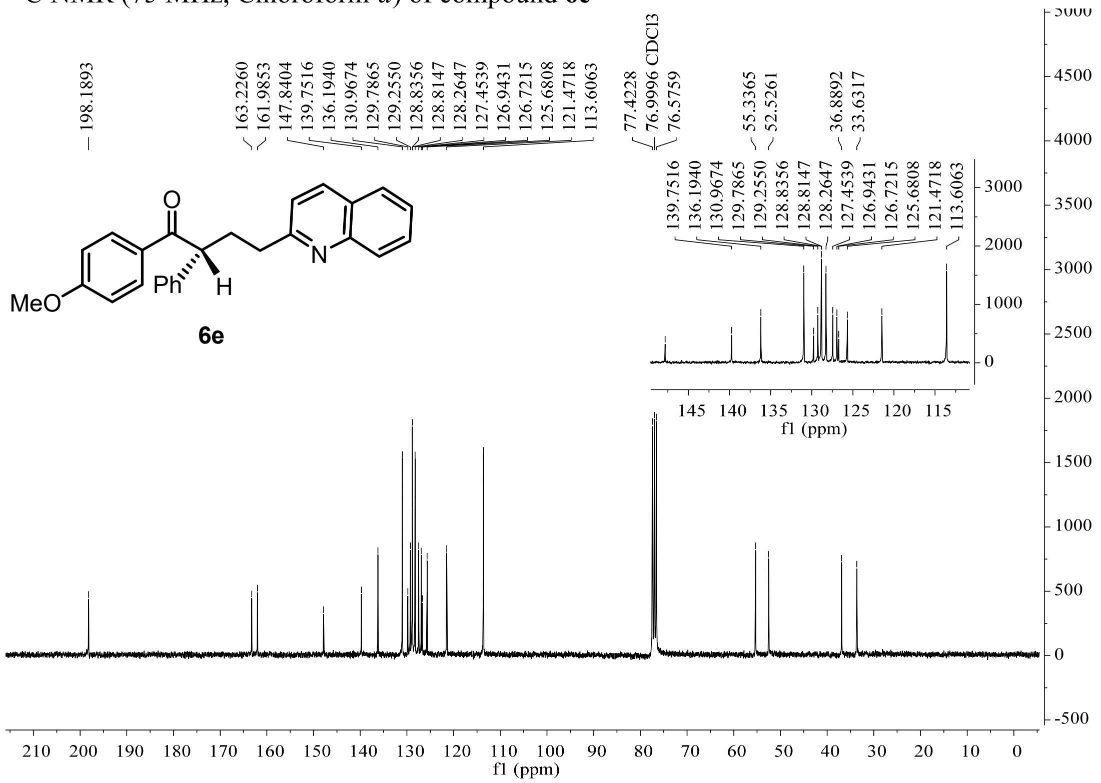

^{1}H NMR (300 MHz, Chloroform-d) of compound **6f**

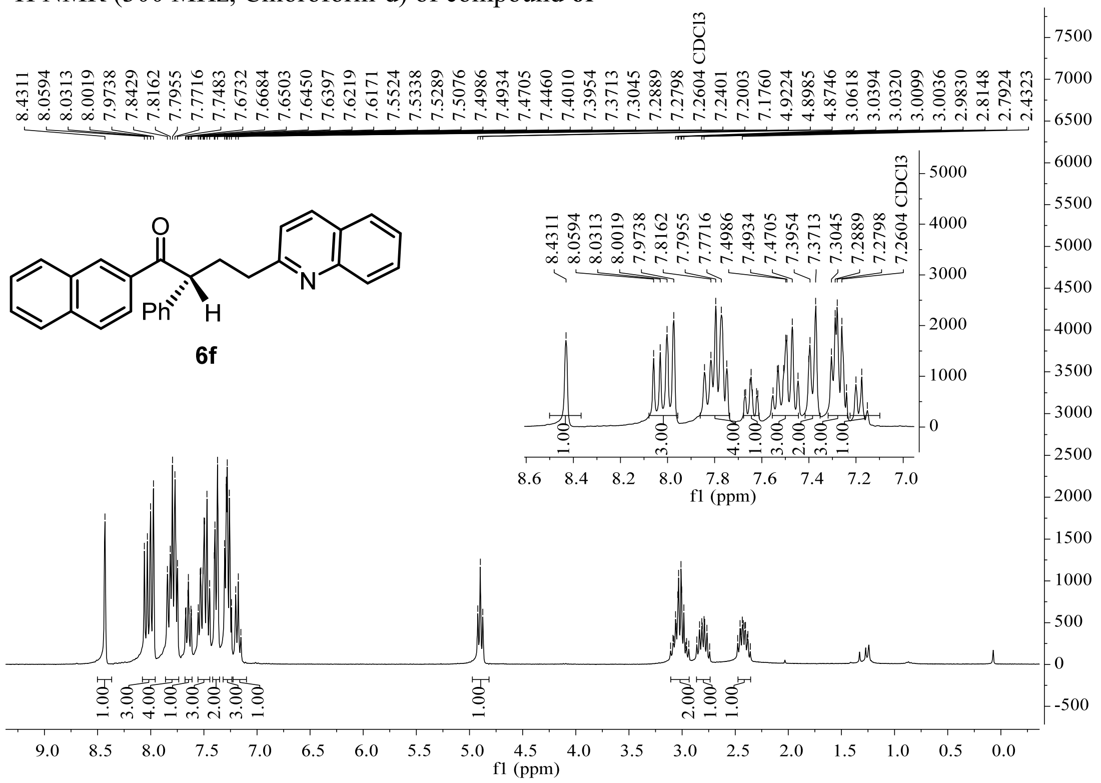

^{13}C NMR (75 MHz, Chloroform-*d*) of compound **6f**

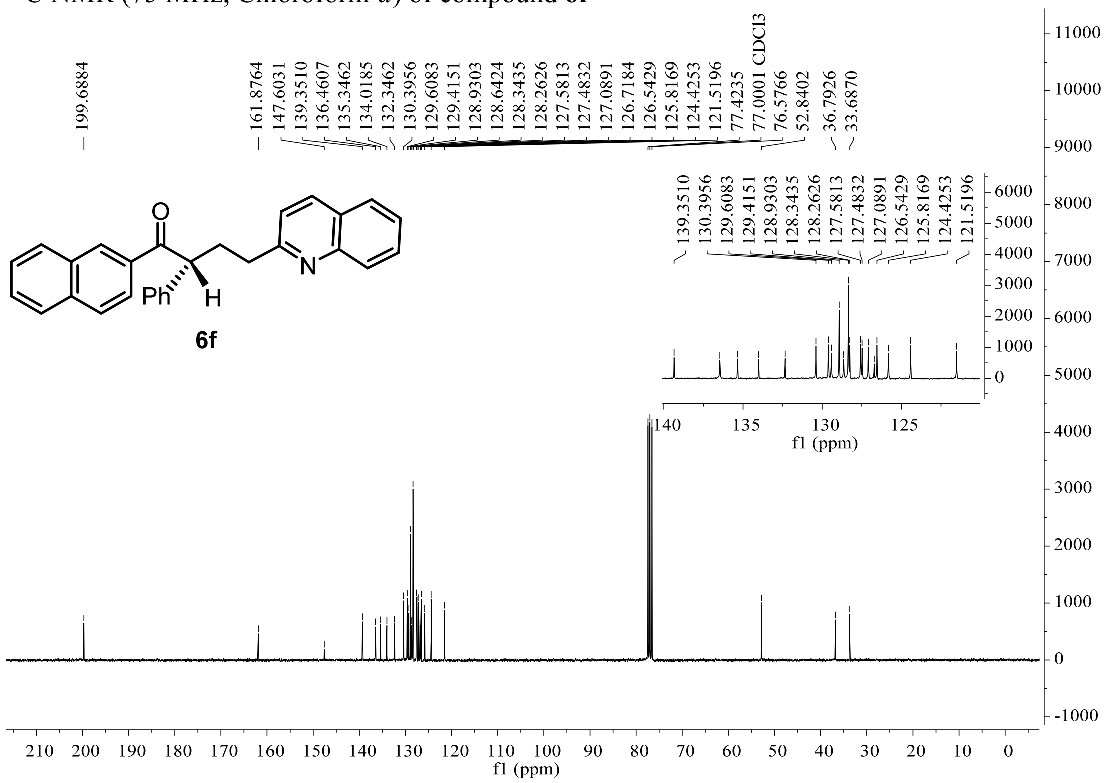

^{1}H NMR (300 MHz, Chloroform-d) of compound **6g**

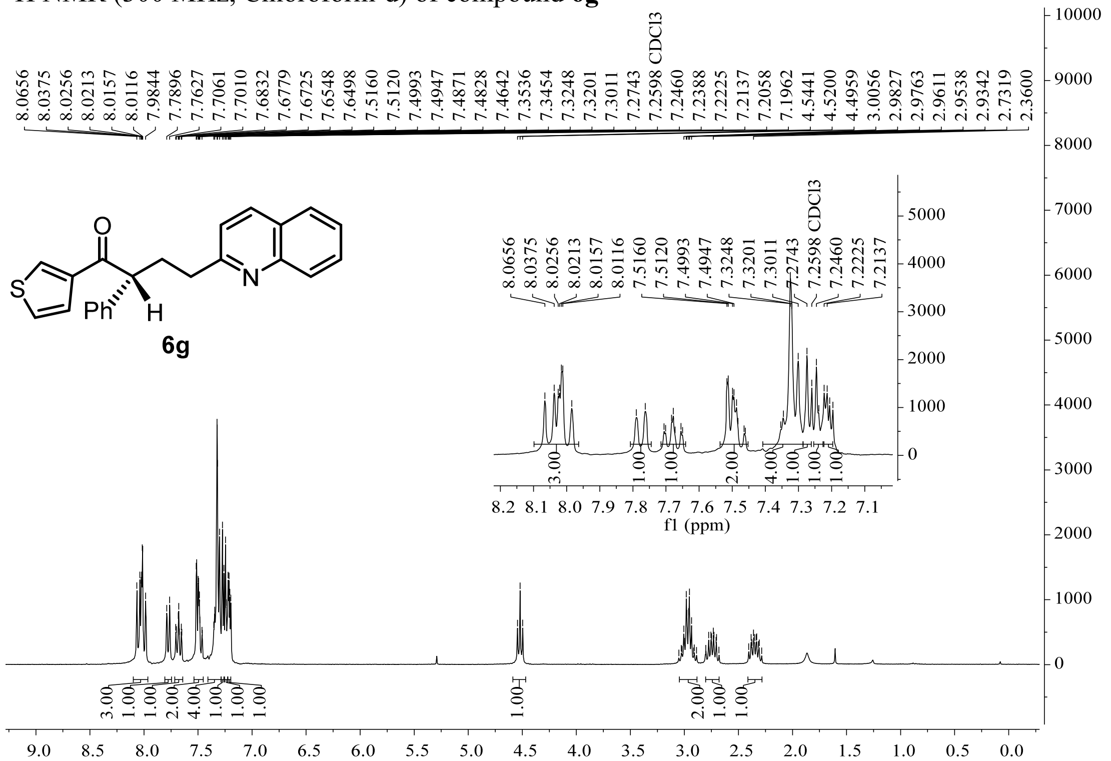

^{13}C NMR (75 MHz, Chloroform-*d*) of compound **6g**

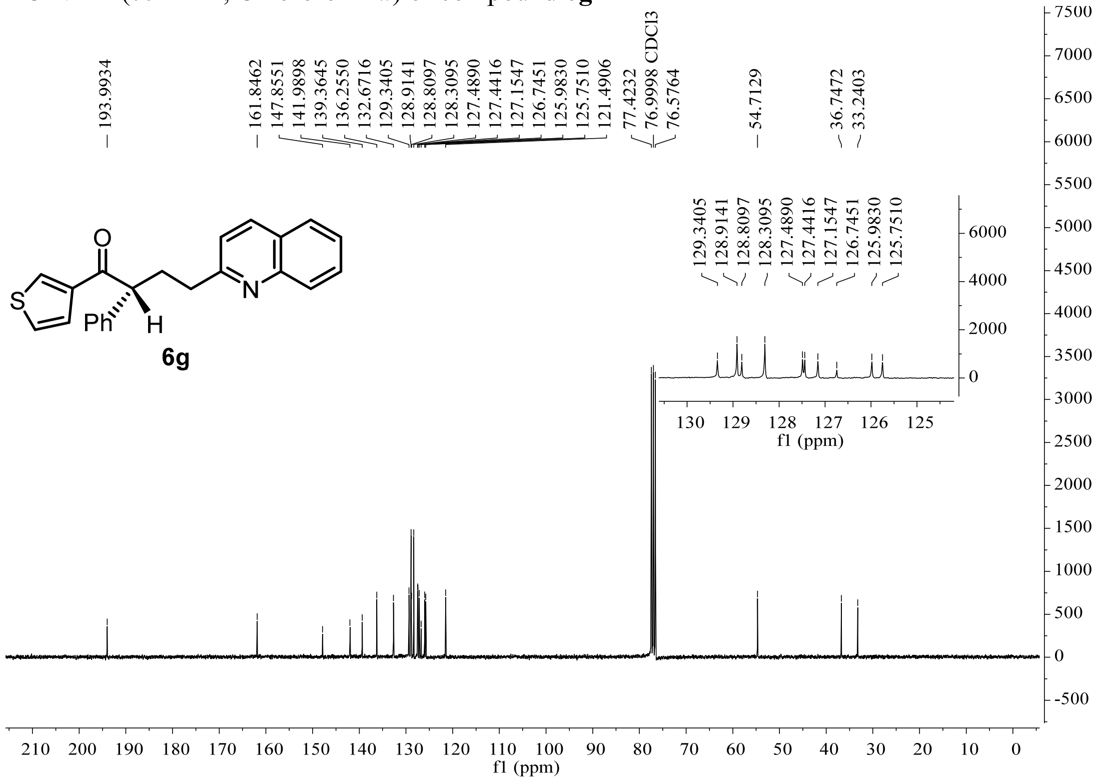

^{1}H NMR (300 MHz, Chloroform-d) of compound **6h**

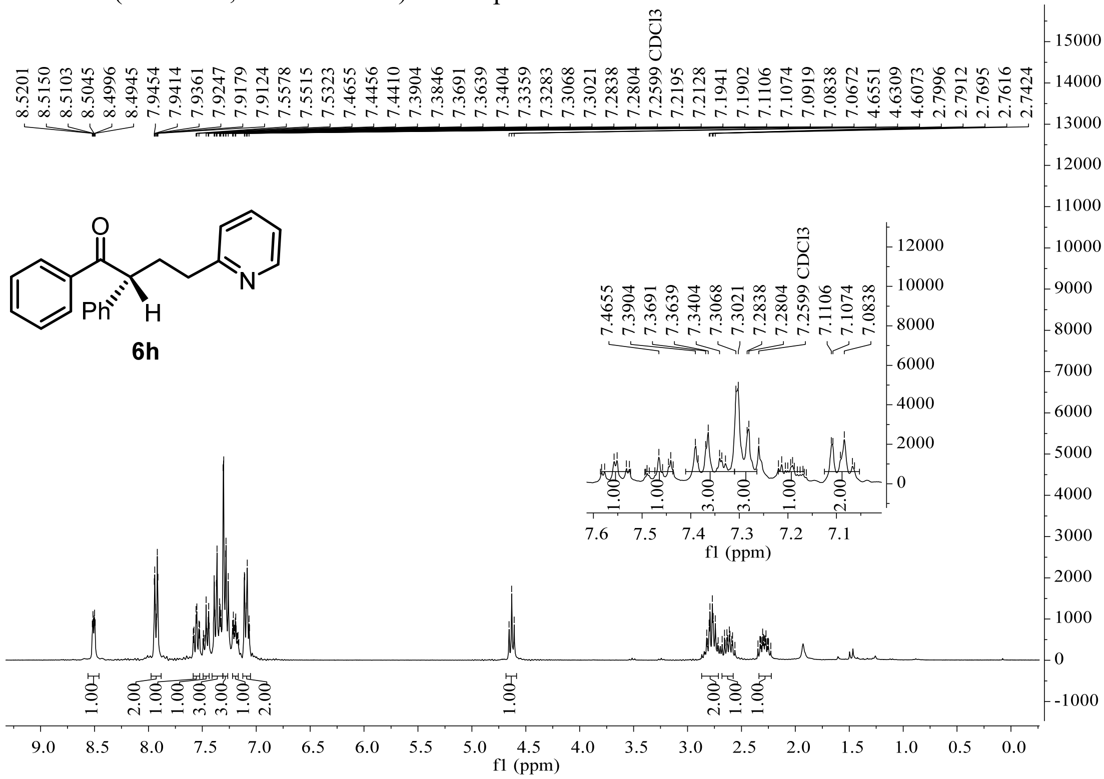

^{13}C NMR (75 MHz, Chloroform-*d*) of compound **6h**

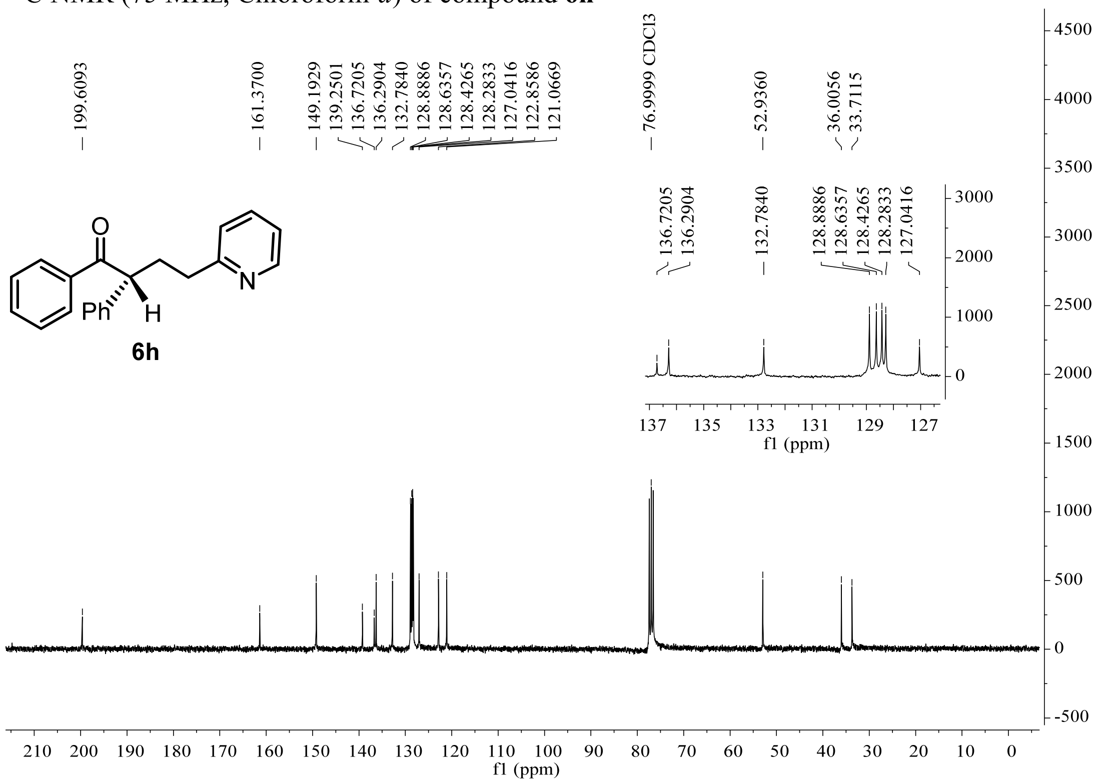

^{1}H NMR (300 MHz, Chloroform-d) of compound **6i**

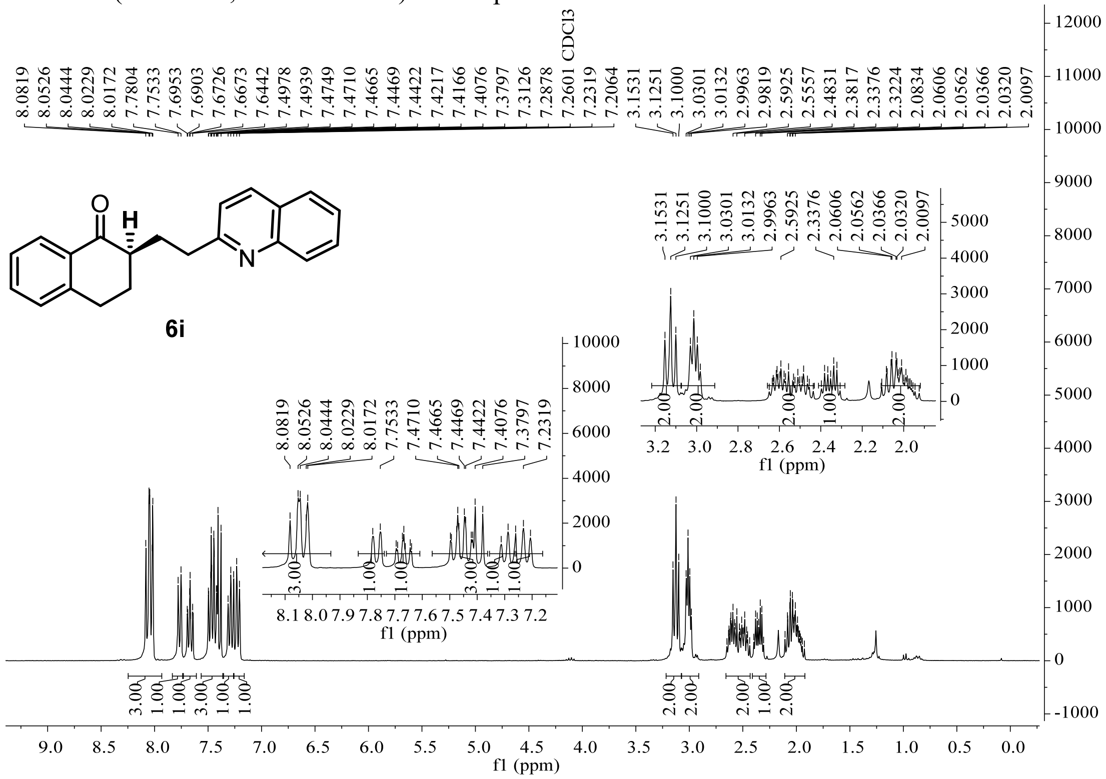

^{13}C NMR (75 MHz, Chloroform-*d*) of compound **6i**

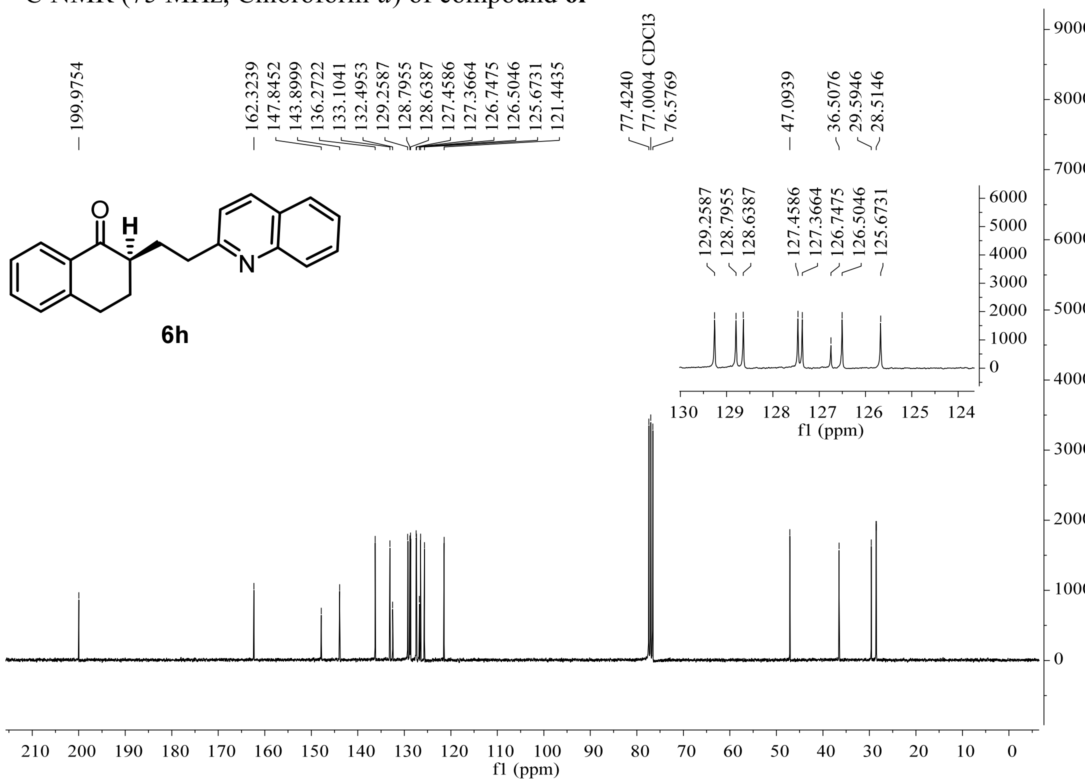

^{1}H NMR (300 MHz, Chloroform-d) of compound **7**

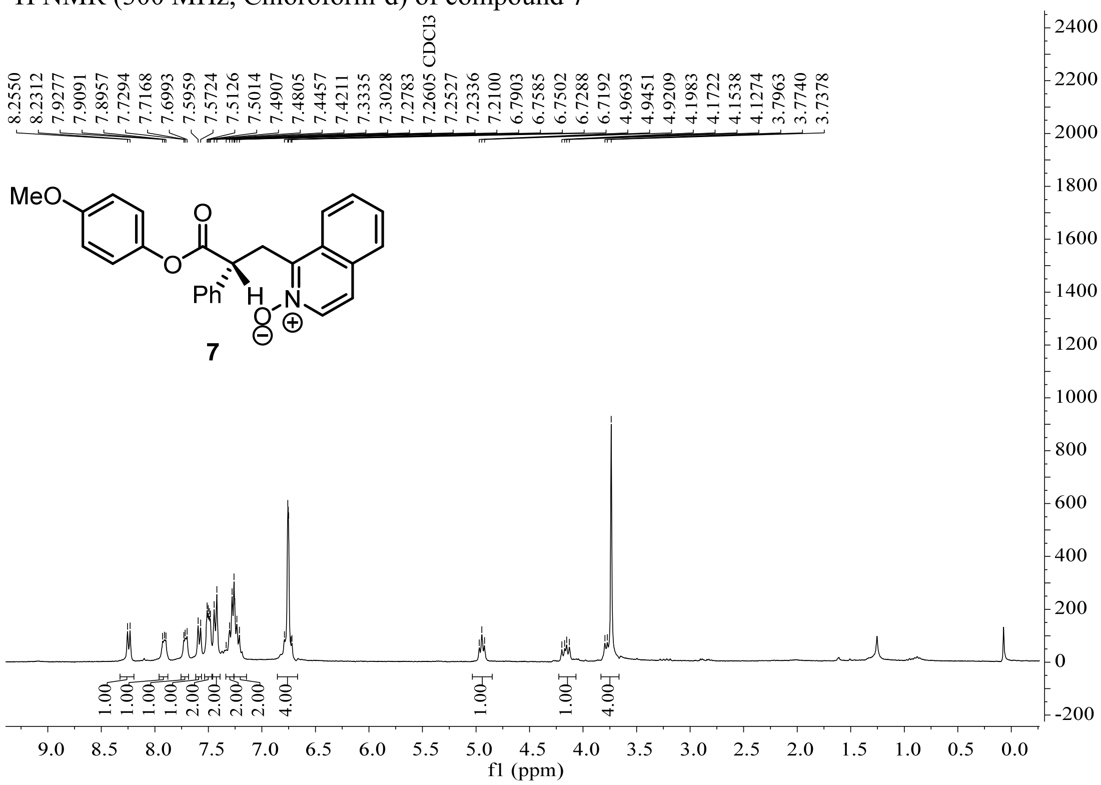

^{13}C NMR (75 MHz, Chloroform-*d*) of compound **7**

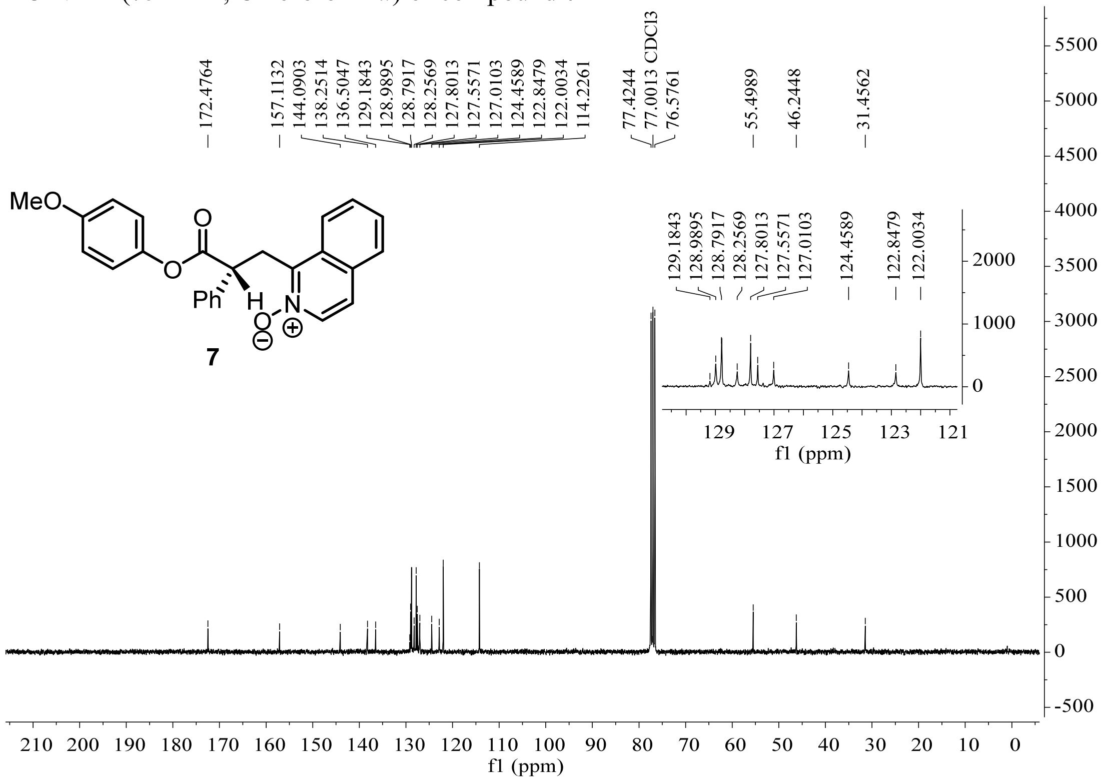

^{1}H NMR (300 MHz, Chloroform-d) of compound **8**

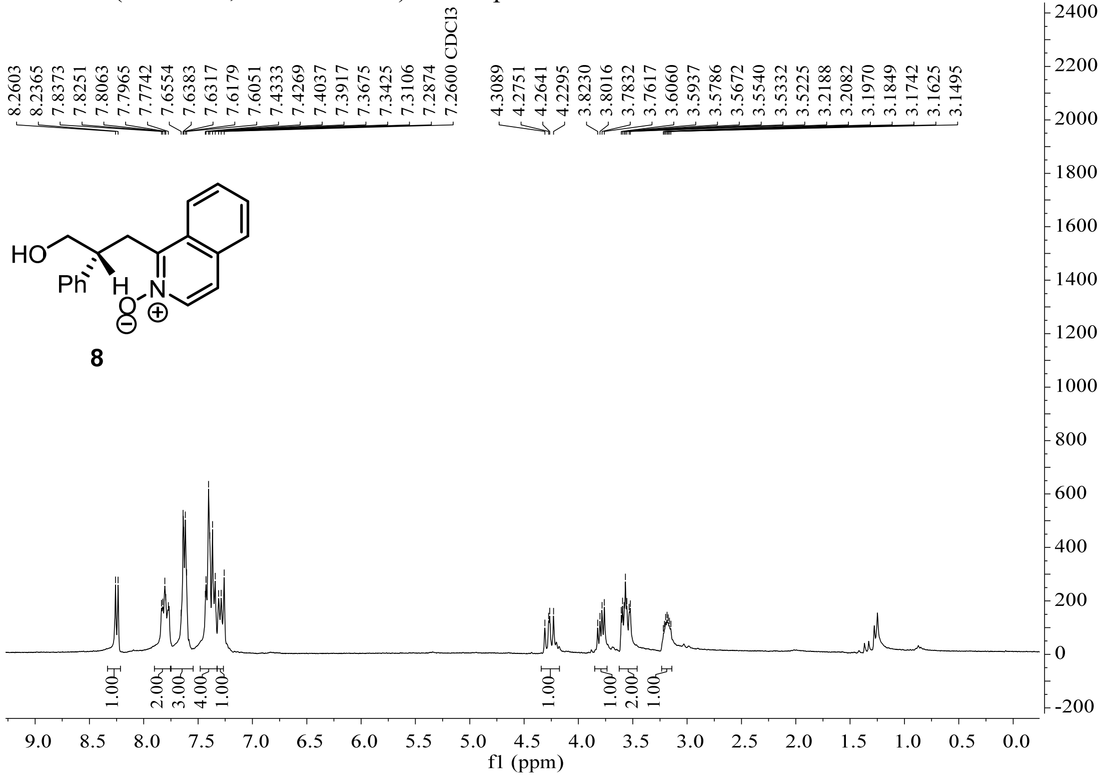

^{13}C NMR (75 MHz, Chloroform-*d*) of compound **8**

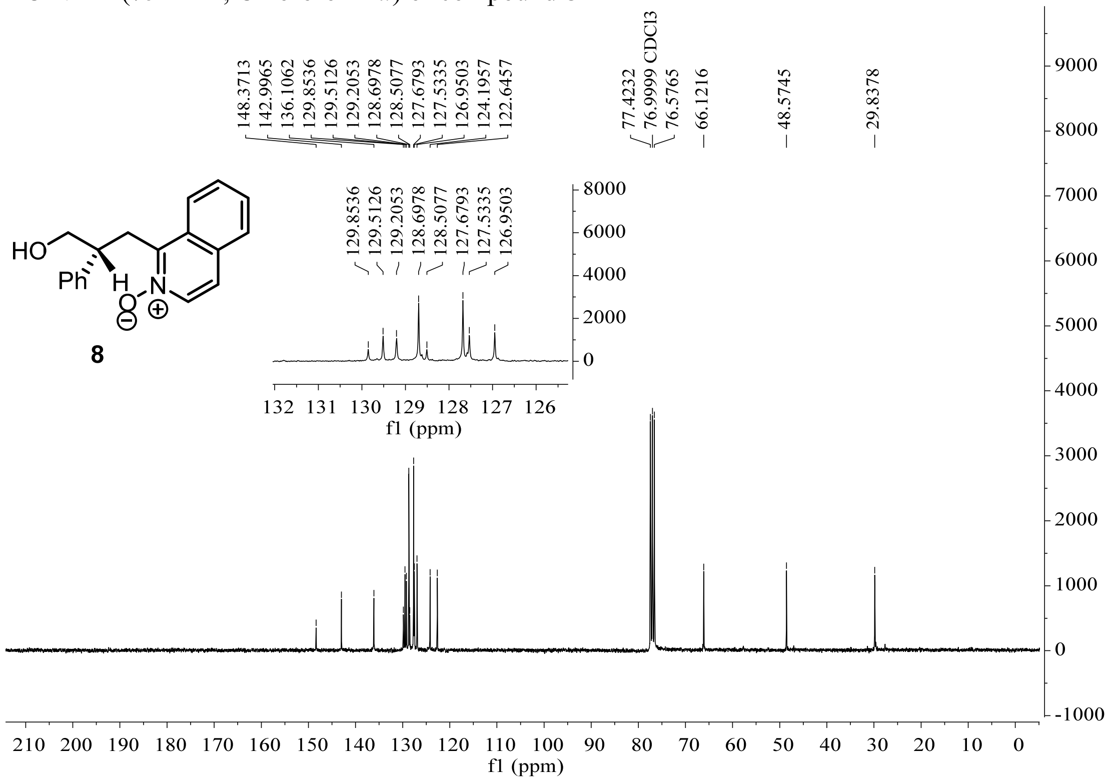

^{1}H NMR (300 MHz, Chloroform-d) of compound **9**

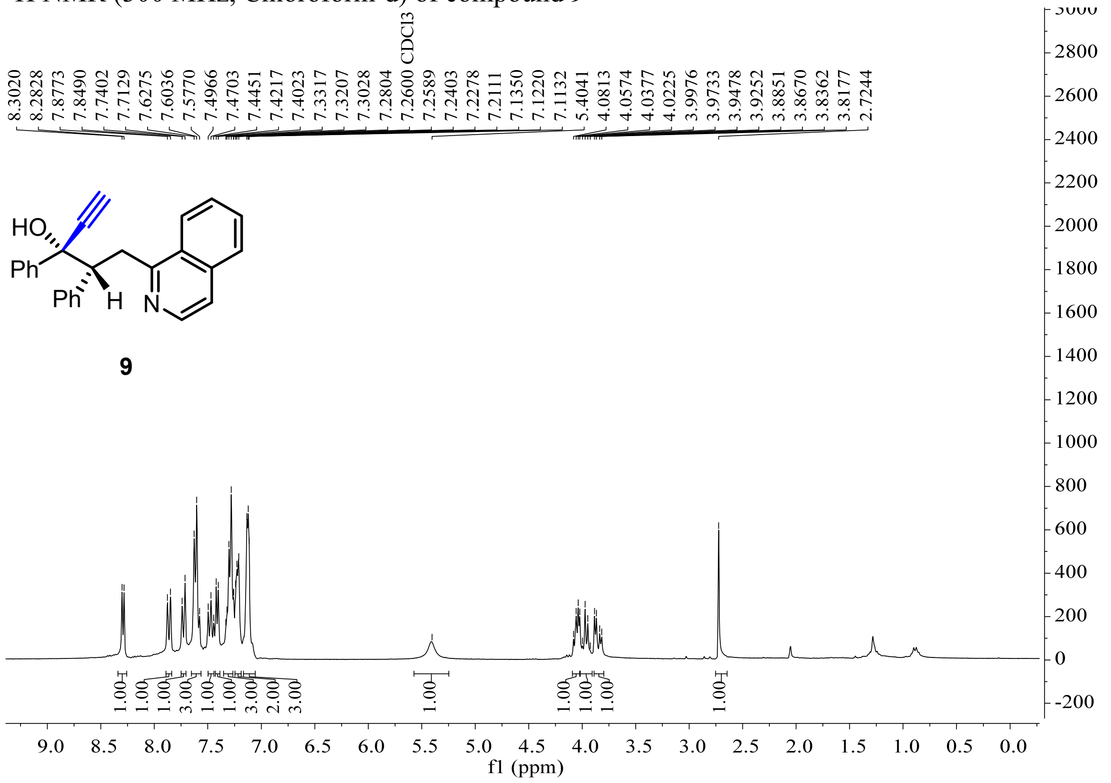

^{13}C NMR (75 MHz, Chloroform-*d*) of compound **9**

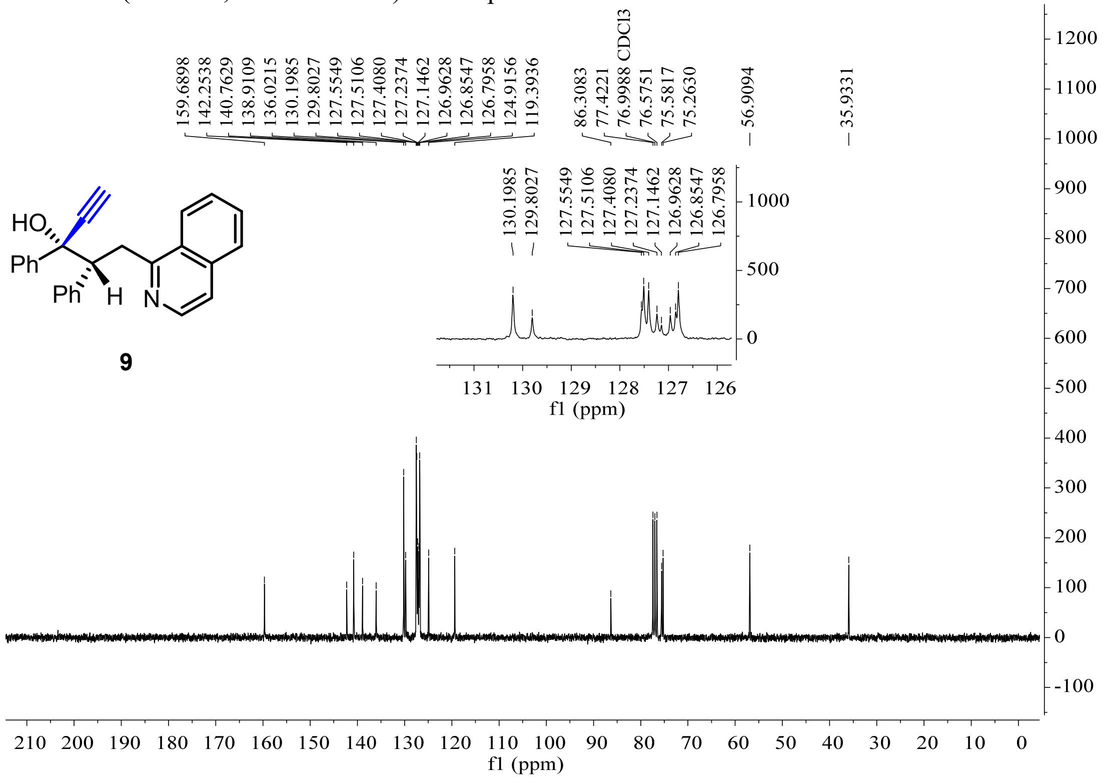

^{1}H NMR (300 MHz, Chloroform-d) of compound **10**

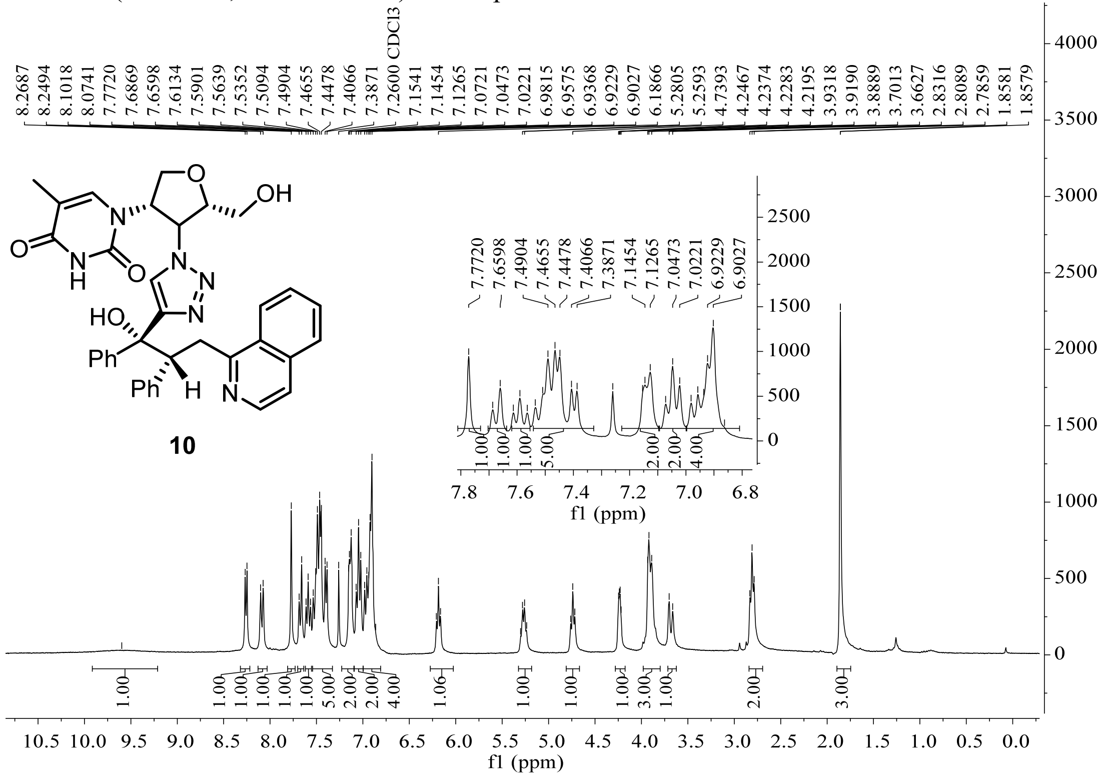

^{13}C NMR (75 MHz, Chloroform-*d*) of compound **10**

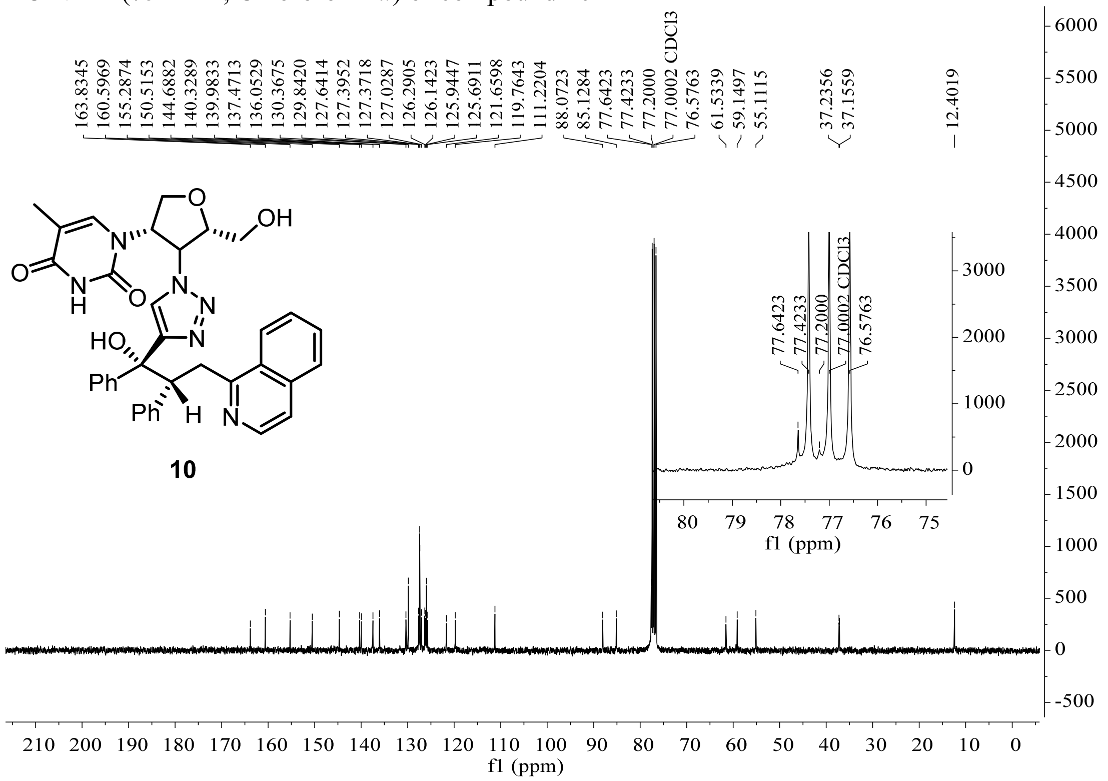

^{1}H NMR (300 MHz, Chloroform-d) of compound **12**

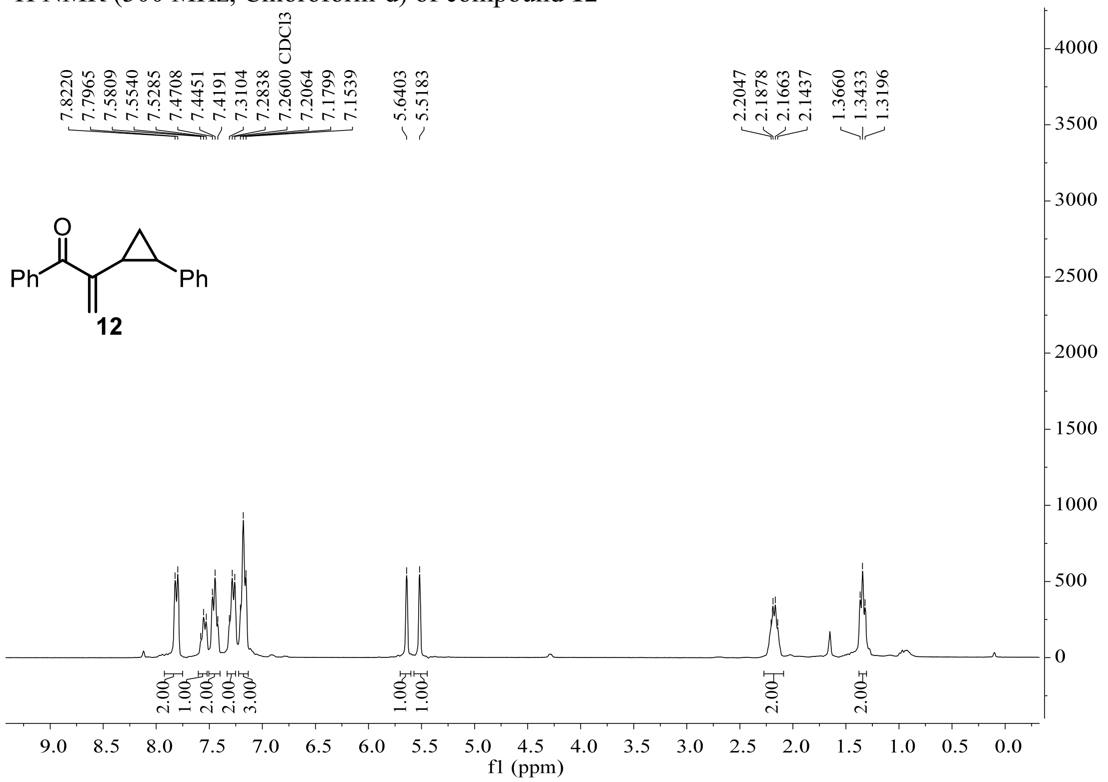

^{13}C NMR (75 MHz, Chloroform-*d*) of compound **12**

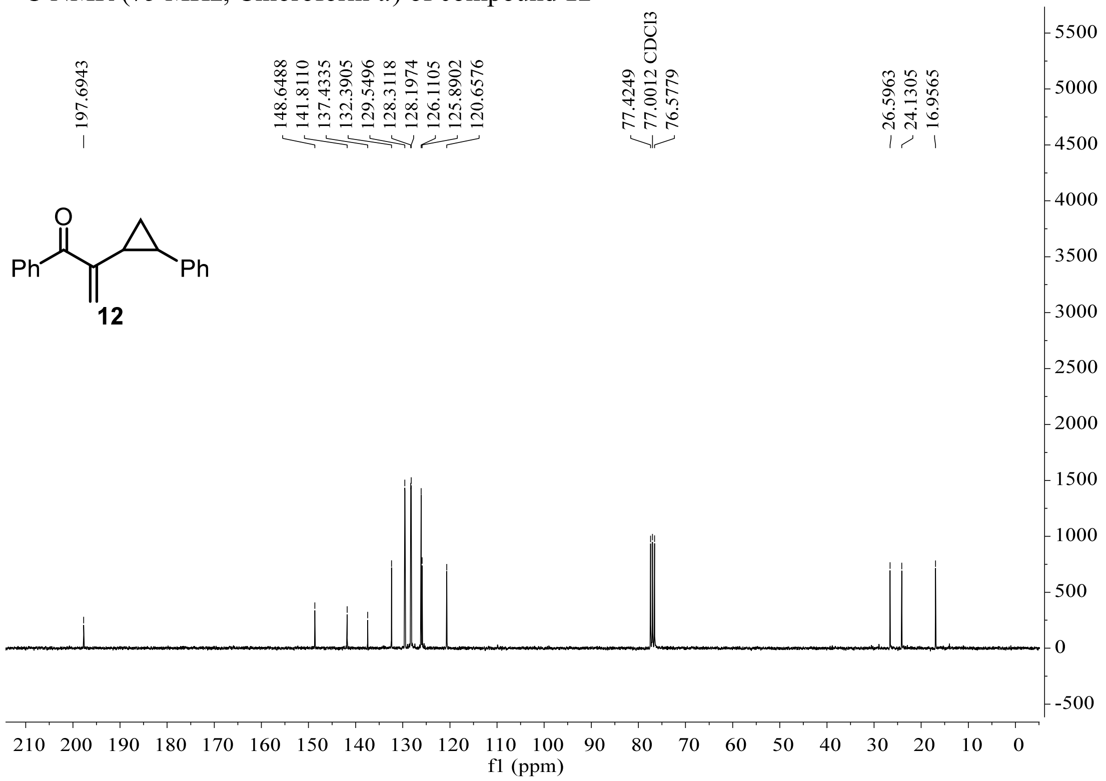

^{1}H NMR (300 MHz, Chloroform-d) of compound **13**

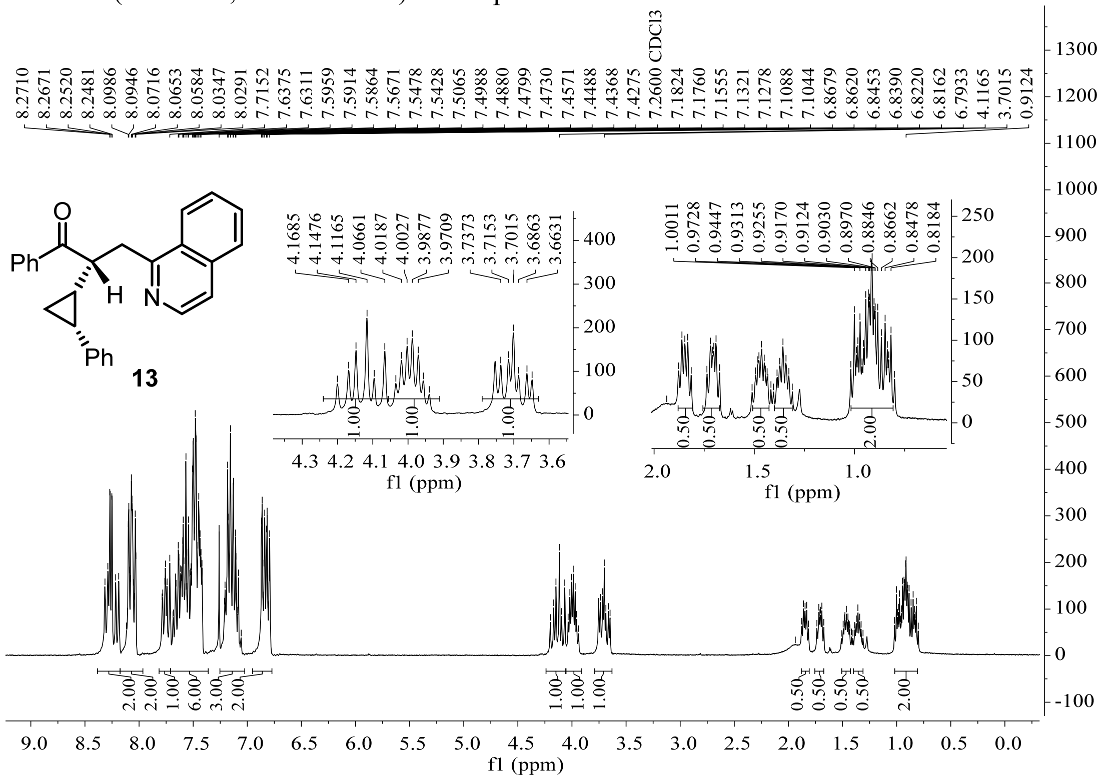

^{13}C NMR (75 MHz, Chloroform-*d*) of compound **13**

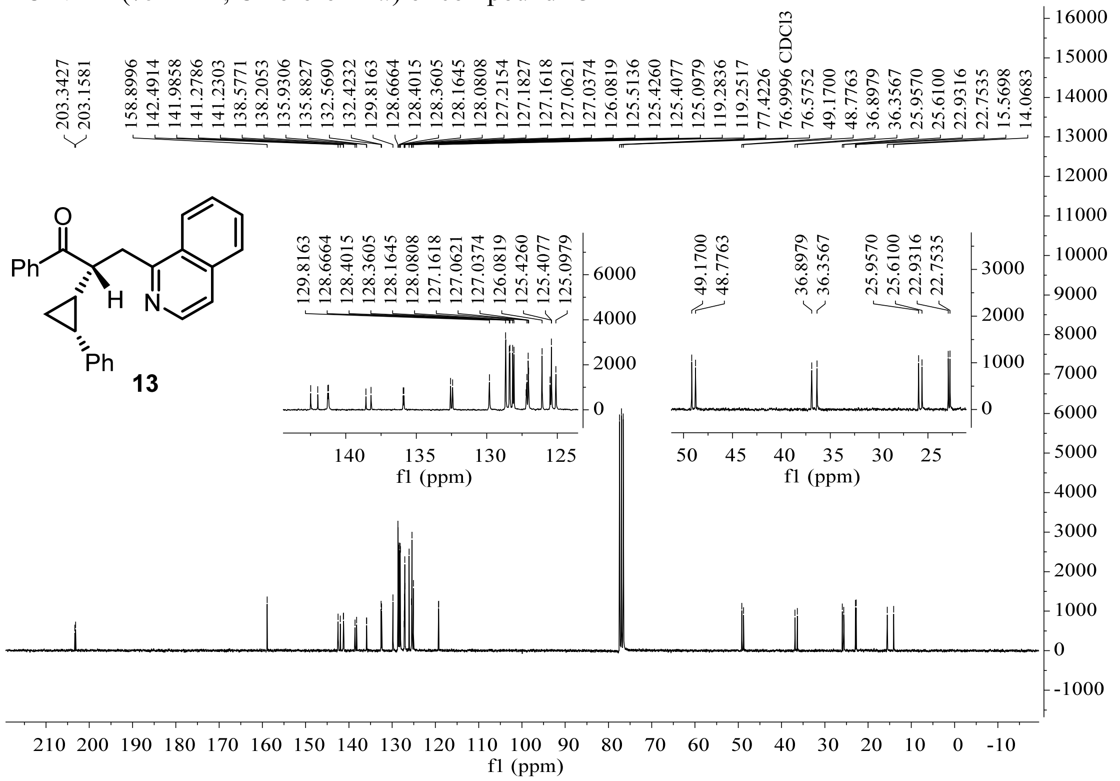

^{1}H NMR (300 MHz, Chloroform-d) of compound **3-phenylbut-3-en-2-one**

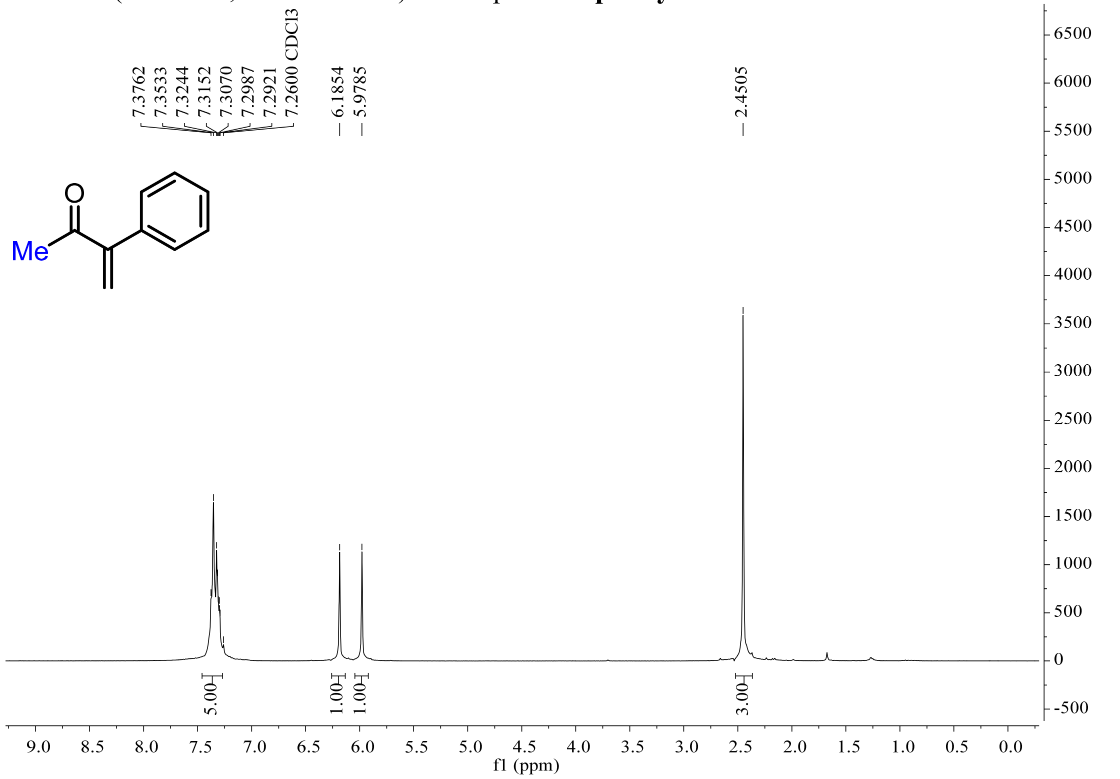

^{13}C NMR (75 MHz, Chloroform-*d*) of compound **3-phenylbut-3-en-2-one**

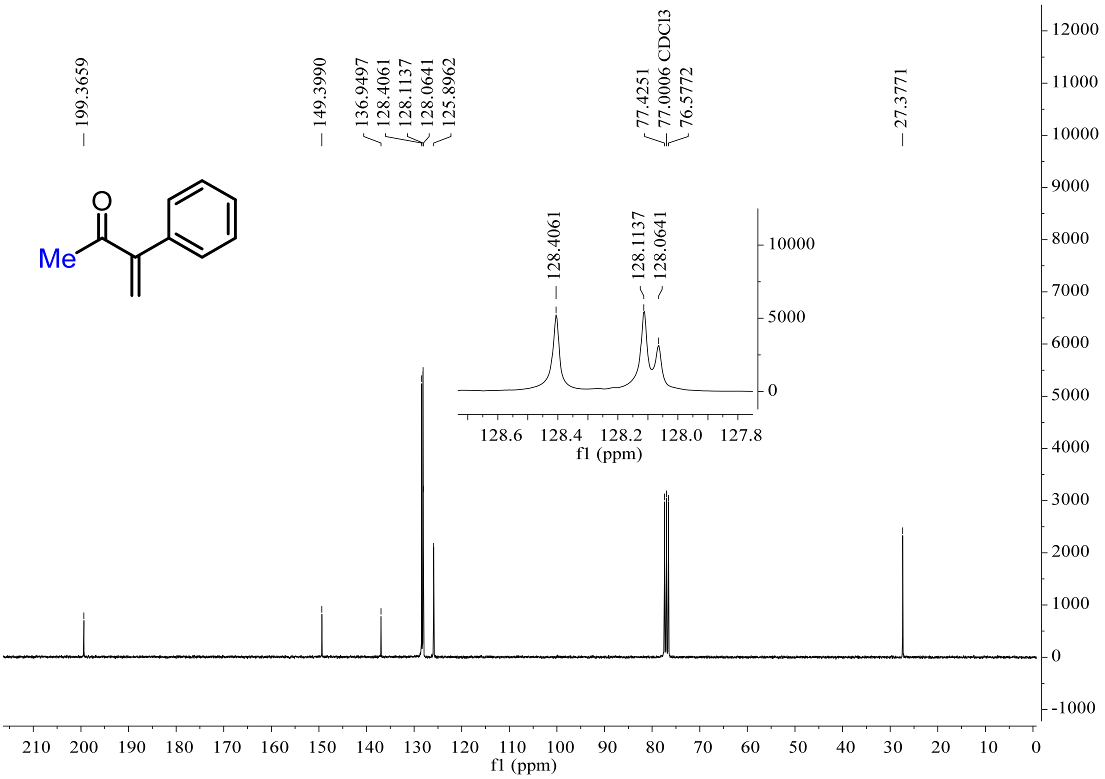

^{1}H NMR (300 MHz, Chloroform-d) of compound 1,4-dicyanobenzene

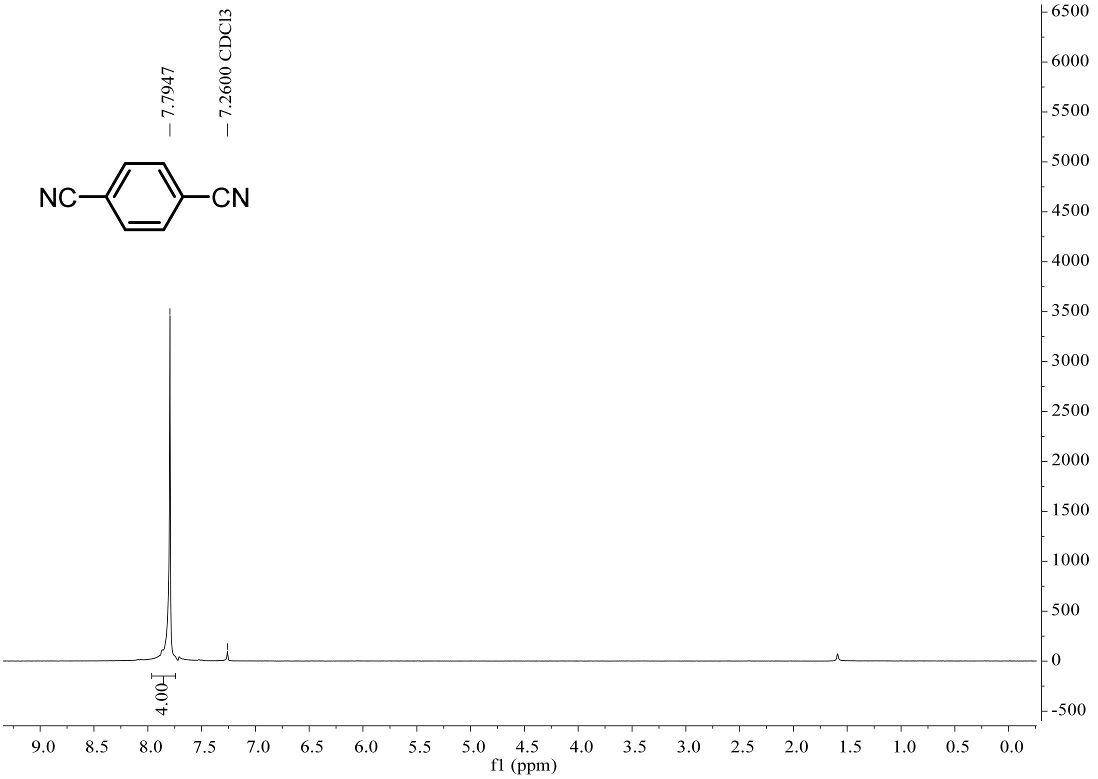

^{13}C NMR (75 MHz, Chloroform-*d*) of compound 1,4-dicyanobenzene

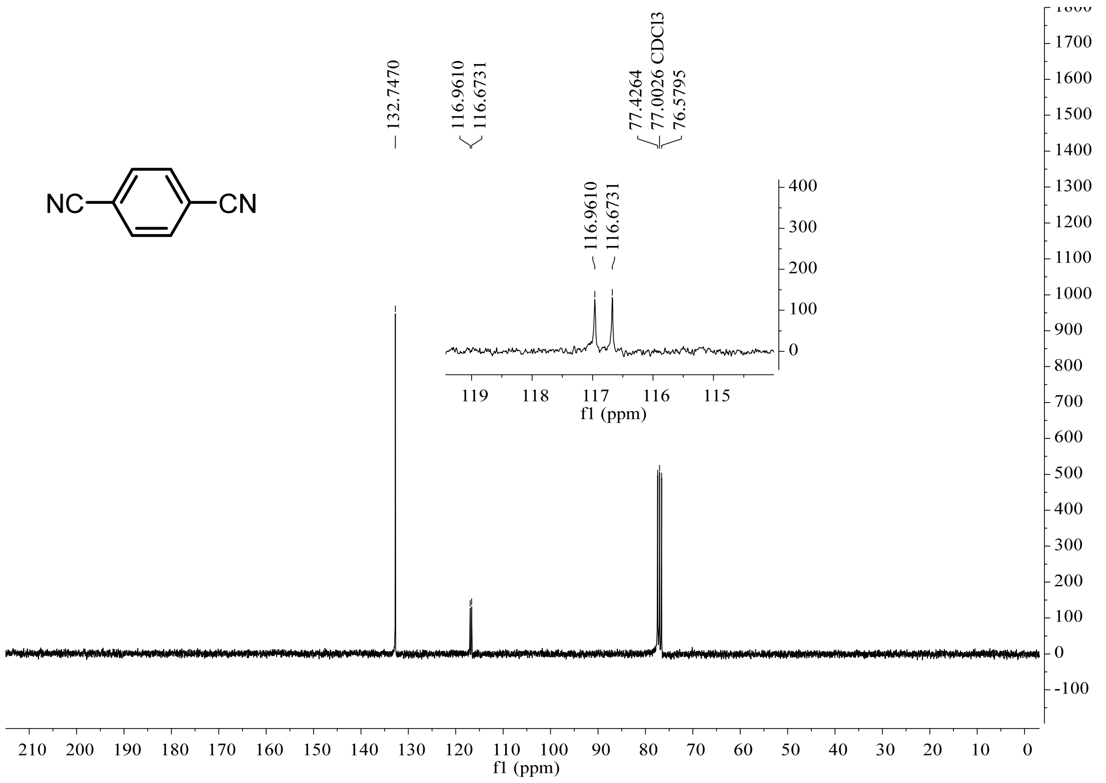

^{1}H NMR (300 MHz, Chloroform-d) of compound benzonitrile

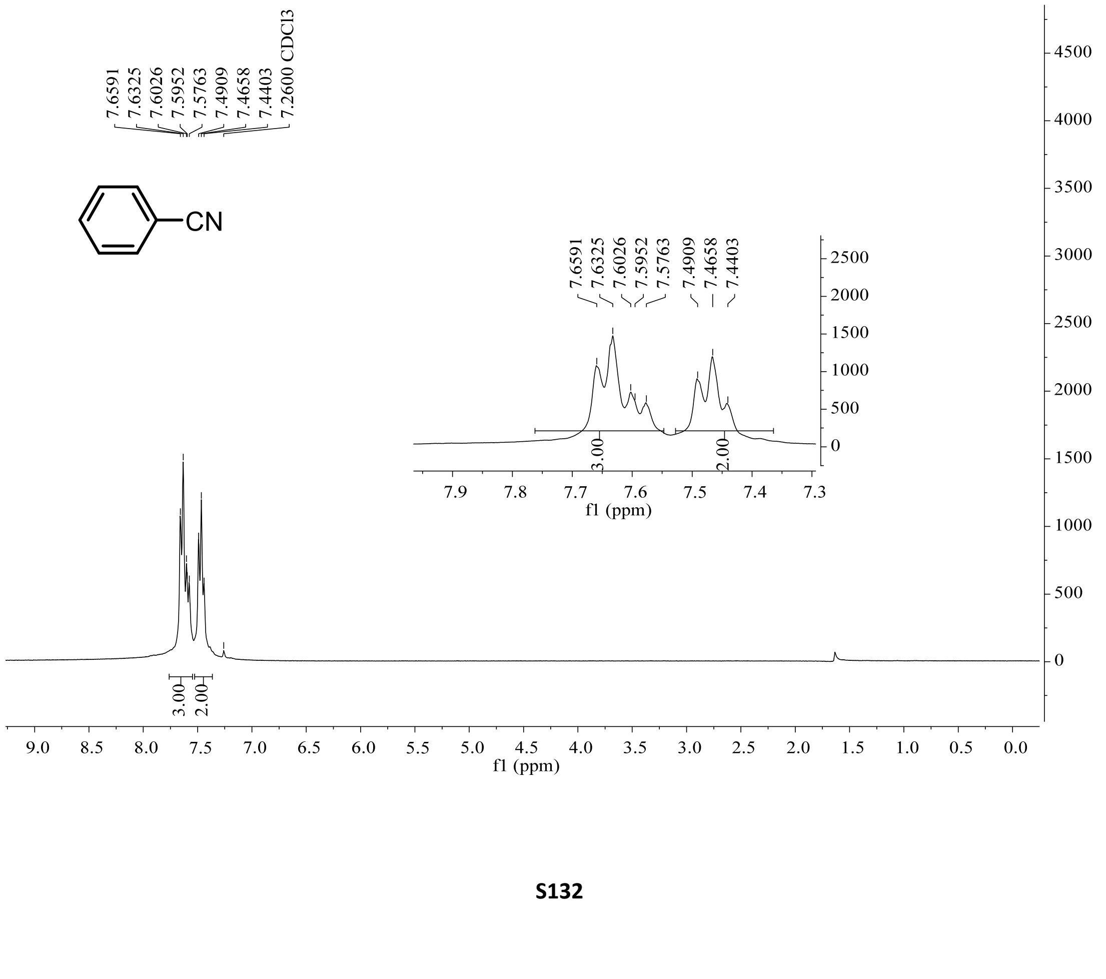

^{13}C NMR (75 MHz, Chloroform-*d*) of compound benzonitrile

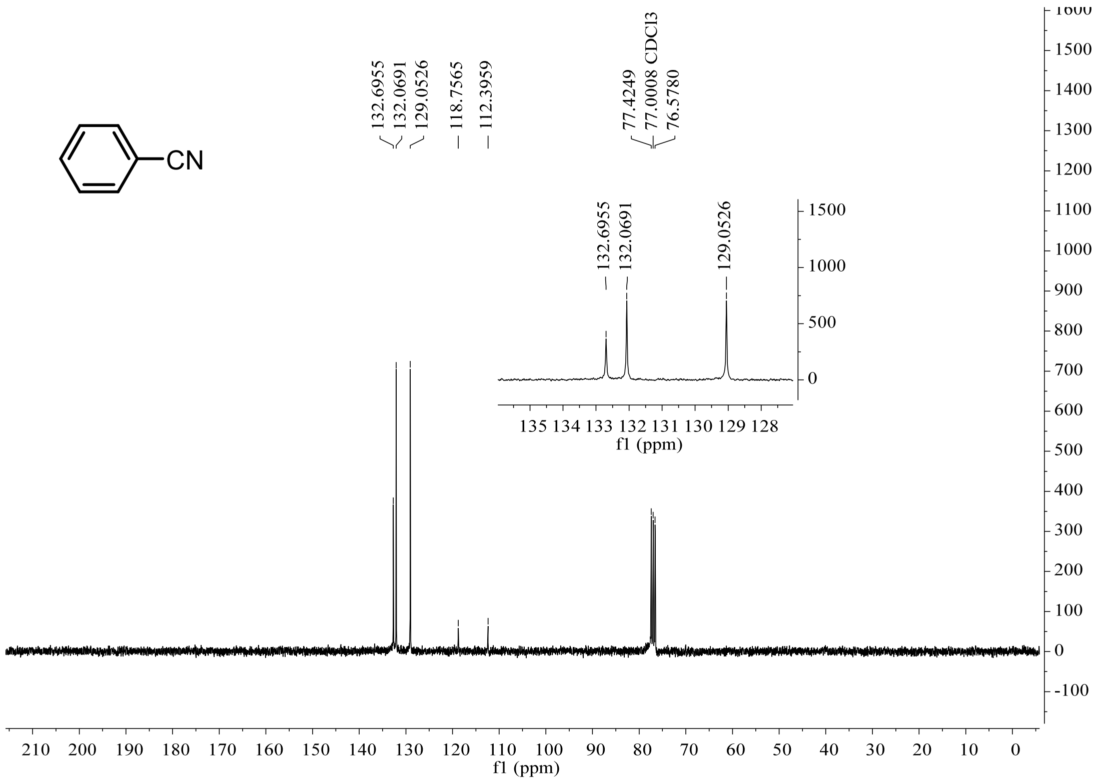

^{1}H NMR (300 MHz, Chloroform-d) of compound 4-cyanobenzyl bromide

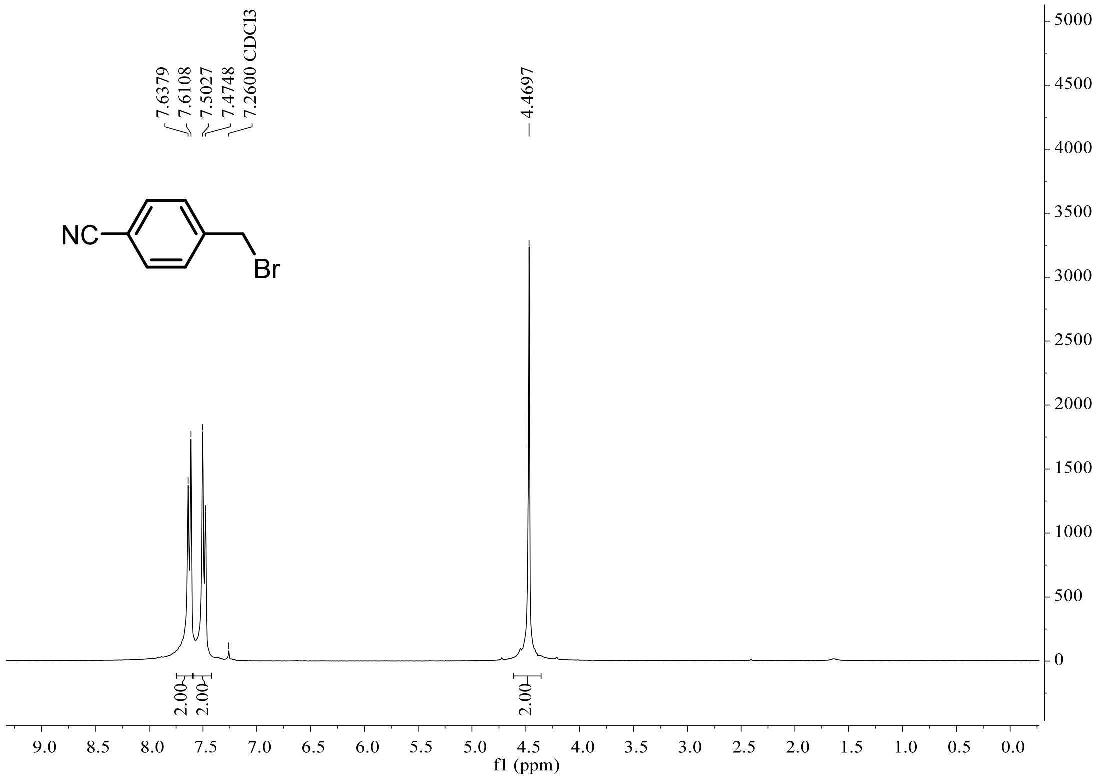

^{13}C NMR (75 MHz, Chloroform-*d*) of compound 4-cyanobenzyl bromide

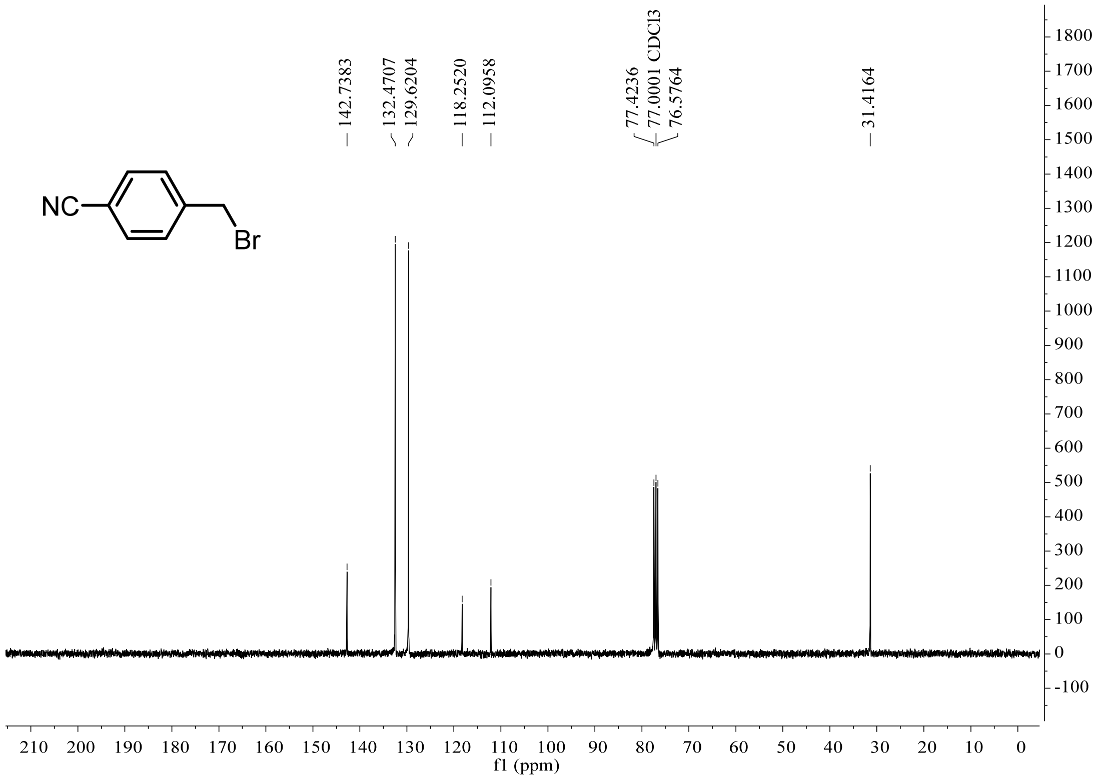

9. General information

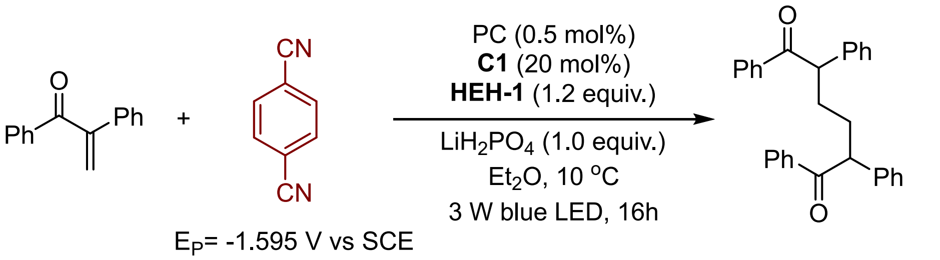

PC = DPZ, *fac*-Ir, 4DPAIPN

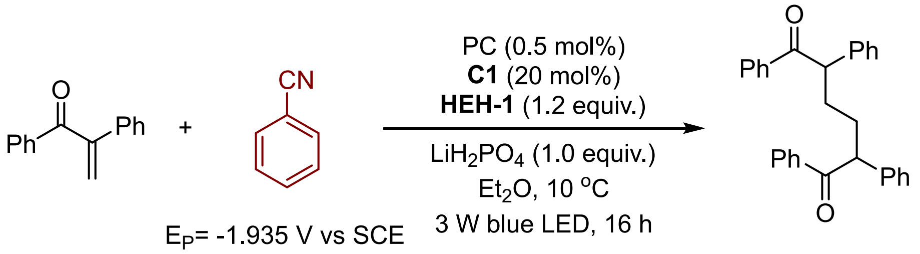

PC = DPZ, *fac*-Ir, 4DPAIPN

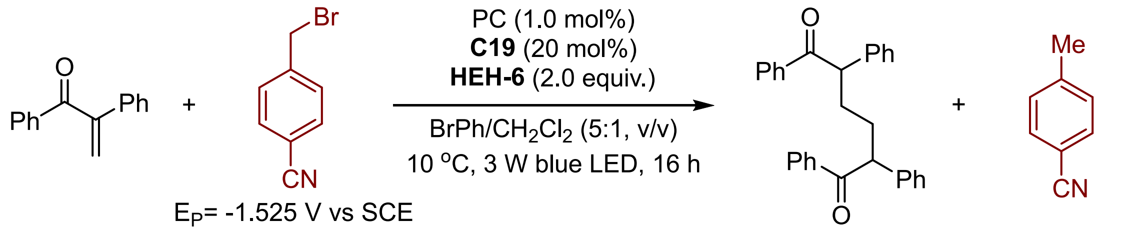

by-product GC-MS

PC = TDPZ, DPZ, *fac*-Ir, 4DPAIPN

Comments: Reductive homo-coupling product of enone was observed in three reactions. Also, in the third reaction, a reductive product from the bromide was detected. Correspondingly, the arene analogues are not compatible with the catalysis platform, presumably due to the unsuitable reactivity for radical coupling.
